# Cross-Dehydrogenative Coupling of Secondary Amines with Silanes Catalyzed by Agostic Iridium-NSi Species

**DOI:** 10.1021/acs.inorgchem.4c04512

**Published:** 2024-12-23

**Authors:** Marina Padilla, María Batuecas, Pilar García-Orduña, Israel Fernández, Francisco J. Fernández-Álvarez

**Affiliations:** † Departamento de Química Inorgánica, Instituto de Síntesis Química y Catálisis Homogénea (ISQCH), Facultad de Ciencias, Universidad de Zaragoza − CSIC, 50009 Zaragoza, Spain; ‡ Departamento de Química Orgánica I and Centro de Innovación en Química Avanzada, Facultad de Ciencias Químicas, Universidad Complutense de Madrid, Ciudad Universitaria, 28040 Madrid, Spain

## Abstract

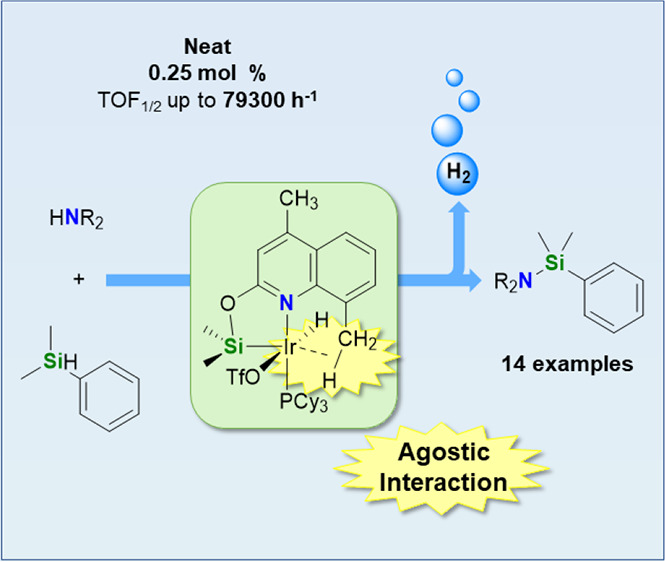

An active catalytic system for the cross-dehydrogenative coupling (CDC) of a wide range of secondary amines with silanes is reported. The iridium(III) derivatives [Ir(H)(X)(κ^2^-NSi^DMQ^)(L)] (NSi^DMQ^ = {4,8-dimethylquinoline-2-yloxy}dimethylsilyl; L = coe, X = Cl, **2**; L = coe, X = OTf, **3**; L = PCy_3_, X = Cl, **4**; L = PCy_3,_ X = OTf, **5**), which are stabilized by a weak yet noticeable Ir···H–C agostic interaction between the iridium and one of the C–H bonds of the 8-Me substituent of the NSi^DMQ^ ligand, have been prepared and fully characterized. These species have proven to be effective catalysts for the CDC of secondary amines with hydrosilanes. The best catalytic performance (TOF_1/2_ = 79,300 h^–1^) was obtained using **5** (0.25 mol %), *N*-methylaniline, and HSiMe_2_Ph. The catalytic activity of the species [Ir(H)(OTf)(κ^2^-NSi^Q^)(PCy_3_)] (**10**, NSi^Q^ = {quinoline-2-yloxy}dimethylsilyl) and [Ir(H)(OTf)(κ^2^-NSi^MQ^)(PCy_3_)] (**11**, NSi^MQ^ = {4-methylquinoline-2-yloxy}dimethylsilyl), related to **5** but lacking the 8-Me substituent, is markedly lower than that found for **5**. This fact highlights the crucial role of the 8-Me substituent of the NSi^DMQ^ ligand in enhancing the catalytic performance of these iridium complexes.

## Abstract 2

The synthesis and characterization of agostic iridium-NSi complexes, along with the study of their catalytic activity for the cross-dehydrogenative coupling of secondary aliphatic amines and aniline derivatives with silanes, are reported.

## Introduction

**Scheme 1:**
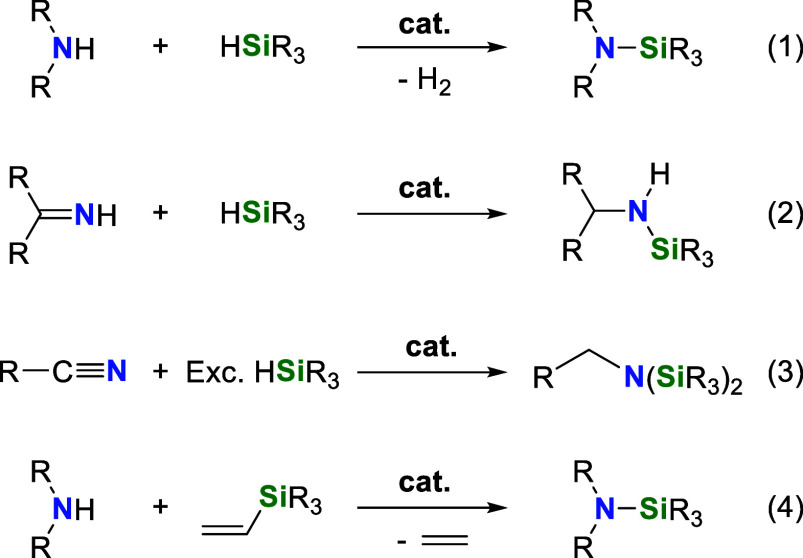
Examples of Catalytic Processes for the Synthesis of Silylamines

*N*-Silylamines (silazanes) are valuable substances in organic synthesis with a wide range of applications such as their utilization as bases, silylating reagents, ligands and precursors for Si/N polymers.^1^ They can be prepared by stoichiometric reactions like ammonolysis of chlorosilanes with amines or by the reaction of lithium amides with halosilanes.^1,2^ The main drawbacks of these methods (e.g., byproduct/waste formation) and the increasing demand for silazanes have promoted research oriented toward the development of catalytic methods for their preparation.^1a,3^ To date, several methodologies for the catalytic synthesis of silazanes are known, including cross-dehydrogenative coupling (CDC) of amines with hydrosilanes (eq 1 in Scheme 1),^4^ hydrosilylation of imines (eq 2 in Scheme 1),^5a^ or nitriles (eq 3 in Scheme 1)^5b^ and dealkenative *N*-silylation of amines with vinylsilanes (eq 4 in Scheme 1).^6^

**Scheme 2:**
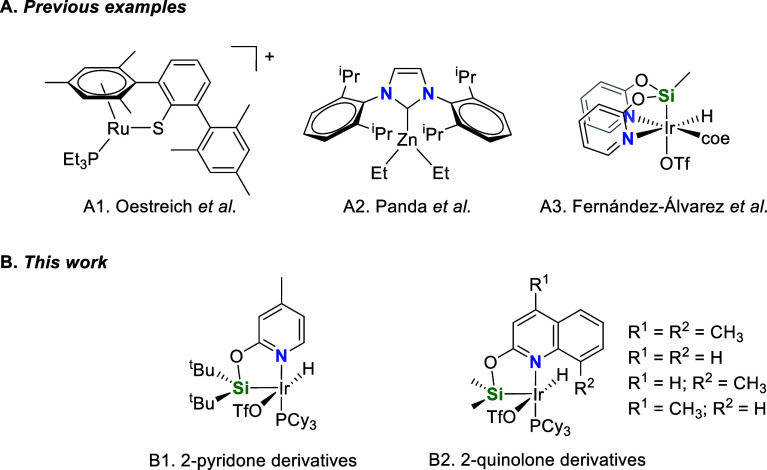
Examples of Homogeneous Catalysts Based on Metal Complexes Effective for the CDC of Amines with Hydrosilanes under Neat Conditions

The catalytic CDC of amines with hydrosilanes appears to be the most attractive synthetic route because it is a straightforward and highly atom-efficient method to produce silazanes with H_2_ as the only side-product. Examples of homogeneous catalysts active for the synthesis of silazanes by CDC of amines with hydrosilanes based on main group elements,^7−10^ lanthanides and actinides,^11^ and transition metals (TM)^12−22^ complexes have been reported. Among these, the Pt(II) cationic species reported by Conejero et al.^21^ stand out for their high activity. Few examples of homogeneous metal-based catalytic systems that operate under neat conditions have been described so far (Scheme 2).^7a,7b,8d,12,17a,20b^ Thus, the development of convenient catalytic systems that are active under neat conditions aiming for sustainable and eco-friendly synthetic approaches is highly desirable.

Our group has previously reported that the iridium(III) complex [Ir(H)(OTf)(*fac*-κ^3^-NSiN)(coe)] (NSiN = bis{pyridine-2-yloxy}methylsilyl) is an effective catalyst precursor for the CDC of secondary amines with hydrosilanes (A3 in Scheme 2).^20b^ However, its scope is limited to aliphatic amines having small substituents. The activity of Ir-(*fac*-κ^3^-NSiN) species as silylation catalysts is strongly conditioned by the steric hindrance and the rigidity of the NSiN ligands.^20b,23−25^ Indeed, we have found that iridium complexes with less hindered monoanionic bidentate κ^2^-NSi ligands^26^ are more active as hydrosilylation and/or silylation catalysts than the related Ir-NSiN species.^27−29^

Our interest in the chemistry of TM–complexes with multidentate organosilyl ancillary ligands lies in the fact that this type of ligands has a strong σ-donor character and the silyl group exhibits a strong *trans*-effect which facilitates the generation of electronically and coordinatively unsaturated species.^25,26^ Moreover, the TM–Si bond between the metal and the silicon atom of κ^3^-NSiN and κ^2^-NSi ligands is extraordinarily robust, stabilizing reaction intermediates.^25a,26^ The robustness of the TM–Si bond in these species has been attributed to the large contribution of the electrostatic component to the total bonding energy.^30^

These backgrounds motivated us to study the potential of 16-electron unsaturated Ir-(κ^2^-NSi) species as catalysts for the CDC of amines with hydrosilanes. Preliminary studies showed that the complex [Ir(H)(OTf)(κ^2^-NSi^tBu2^)(PCy_3_)] (NSi^tBu2^ = {4-methylpyridine-2-iloxy}ditertbutylsilyl) (B1 in Scheme 2),^30b^ recently described by our group, exhibits a moderate activity as catalyst for the CDC of *N*-methylaniline (1 mol %, 323 K, TOF_1/2_ = 530 h^–1^; TOF_1/2_ (h^–1^) = TOF (h^–1^) at 50% conversion, Figure S1) with HSiMe_2_Ph, which led us to design a more active catalyst. Therefore, we decided to design κ^2^-NSi ligands having less sterically hindered Ir–Si bonds, with methyl instead tertbutyl substituents at the silicon atom. However, when using (4-methylpyridine-2-iloxy)dimethylsilane as proligand, the coordination of two ligand units to the metal could not be avoided.^27^ In this regard, Huertos et al. have recently reported that the use of 8-(dimethylsilyl)quinoline and related compounds as proligands make it possible to prepare unsaturated rhodium(III) complexes with only one unit of the corresponding κ^2^-NSi ligand.^31^ Therefore, we decided to explore the potential of 2-quinolone derivatives, instead of 2-pyridone derivatives, as proligands for the preparation of Ir-(κ^2^-NSi) species (B2 in Scheme 2).

As a result of these studies, it has been possible to prepare unsaturated Ir-(κ^2^-NSi) species that are active catalysts for the catalytic CDC of secondary amines with hydrosilanes, affording a wide variety of *N*-silylamines under mild reaction conditions. Moreover, the use of (quinoline-2-yloxy)dimethylsilane derivatives as proligands has allowed us to compare the influence of the presence/absence of the 8-Me substituent, which enables Ir···H–C agostic interactions, on the catalytic activity of the resulting complexes.

## Results and Discussion

### Synthesis and Characterization of Ir-(κ^2^-NSi) Agostic Precursors

The reaction of 4,8-dimethyl-2-hydroxyquinoline with HSiMe_2_Cl and NEt_3_ in THF leads to the functionalized silane (4,8-dimethylquinoline-2-yloxy)dimethylsilane (**1**), which was isolated as a white solid in 74% yield. Compound **1** was characterized by means of NMR spectroscopy and used without further purification. The ^1^H NMR spectrum of **1** shows the Si–*H* proton as a septuplet resonance at δ 5.43 ppm (^3^*J*_HH_ = 2.9 Hz) coupled with the SiMe_2_ protons that appear as a doublet resonance at δ 0.53 ppm (Figure S15). The ^1^H–^29^Si HMBC NMR (C_6_D_6_) experiment shows a correlation between the resonance corresponding to the Si–*H* proton in the ^1^H NMR spectrum and a singlet resonance at δ 3.3 ppm in the ^29^Si NMR spectrum (Figure S19).

**Scheme 3:**
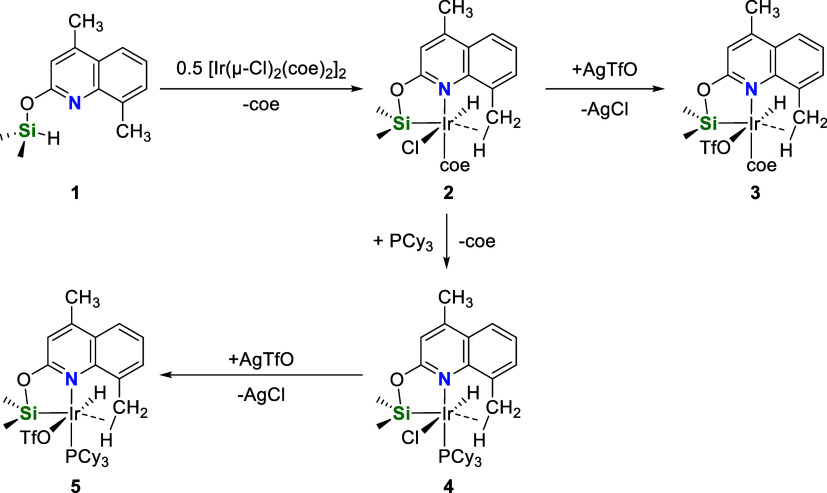
Synthesis of Complexes **2**, **3**, **4**, and **5**

The iridium(III) species [Ir(H)(Cl)(κ^2^-NSi^DMQ^)(coe)] (**2**) (NSi^DMQ^ = {4,8-dimethylquinoline-2-yloxy}dimethylsilyl; coe = *cis*-cyclooctene) was prepared by reaction of freshly prepared **1** with [{Ir(coe)_2_}_2_(μ-Cl)_2_] (ratio 2:1) in CH_2_Cl_2_ at room temperature (r.t.) (Scheme 3). Complex **2** was isolated in 78% yield and characterized by elemental analysis, high-resolution mass spectrometry (HR-MS) and NMR spectroscopy. The reaction of **2** with one equivalent of silver triflate gives the corresponding triflate derivative [Ir(H)(OTf)(κ^2^-NSi^DMQ^)(coe)] (**3**), which has been isolated as a pale-yellow solid in 90% yield. The most noticeable resonance in the ^1^H NMR spectra of **2** and **3** is due to the Ir–*H* moiety that appears as a singlet at δ −16.64 (**2**) ppm (Figure S20) and δ −25.78 (**3**) ppm (Figure S25). The ^29^Si chemical shift obtained by ^1^H–^29^Si HMBC NMR experiment appears at δ 32.8 ppm (**2**) and 32.2 ppm (**3**), low field shifted in comparison to the value of δ 3.3 ppm found for **1**, which confirms the presence of the Ir–Si bond (Figures S24 and S30). Complex **2** reacts with one equivalent of PCy_3_ in toluene to afford the corresponding species [Ir(H)(Cl)(κ^2^-NSi^DMQ^)(PCy_3_)] (**4**), which was isolated as a pale-green solid in 72% yield. Complex **4** reacts with one equivalent of silver triflate to give [Ir(H)(OTf)(κ^2^-NSi^DMQ^)(PCy_3_)] (**5**), which was isolated as a pale-brown solid in 93% yield (Scheme 3).

**Figure 1:**
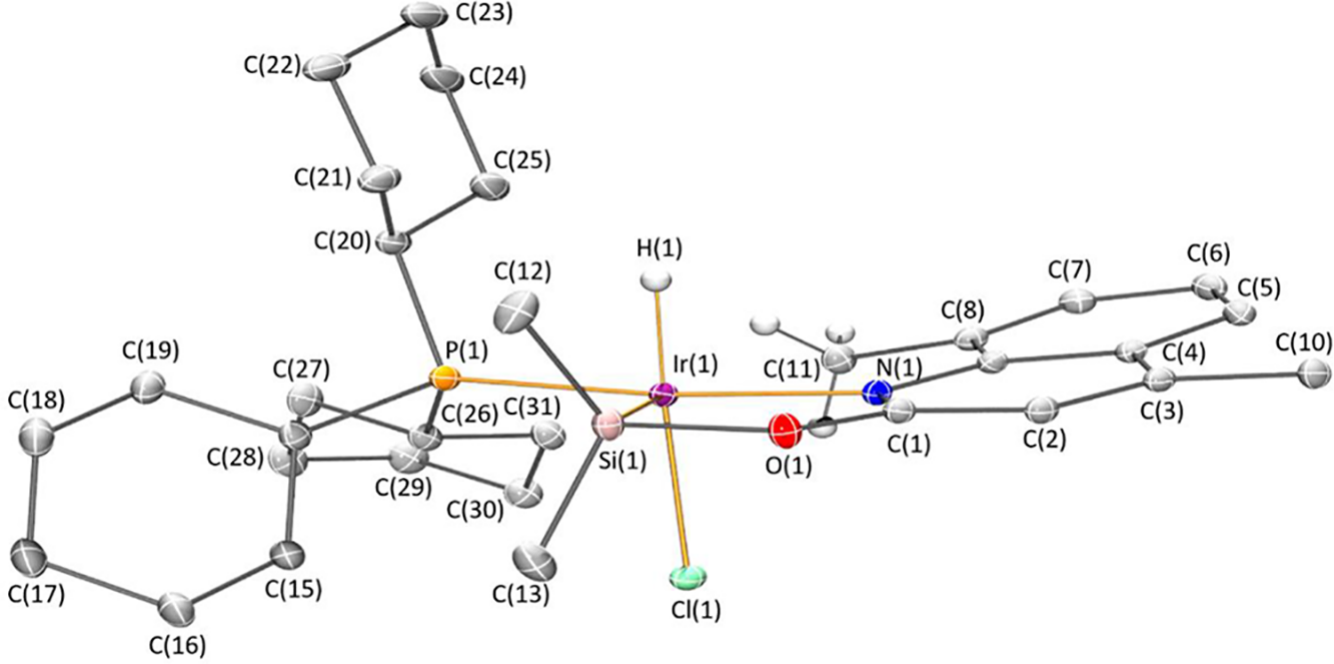
Molecular structure of **4**. Hydrogen atoms (except hydride and those of the methyl group involved in agostic interactions) are omitted for clarity.

**Figure 2:**
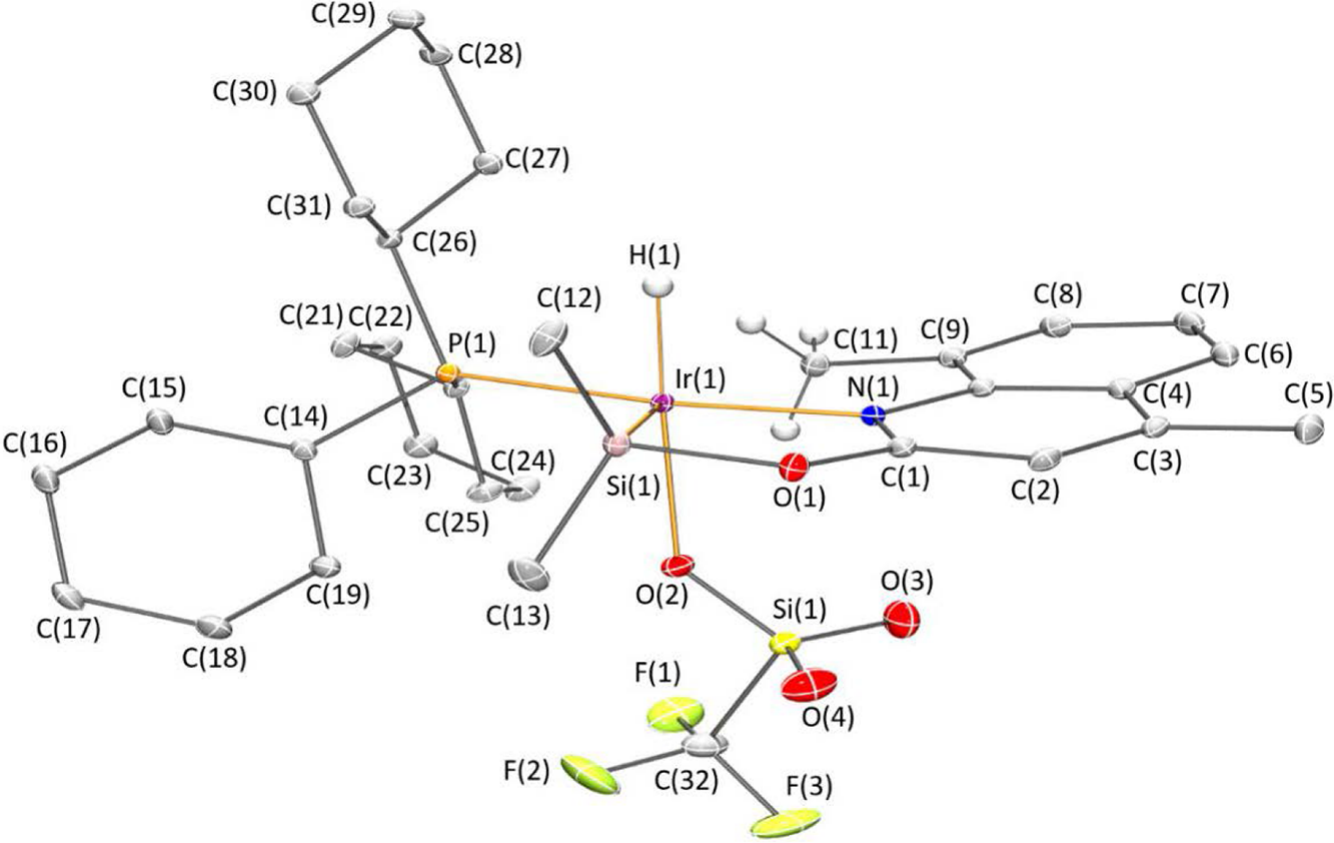
Molecular structure of **5**. Hydrogen atoms (except hydride and those of the methyl group involved in agostic interactions) are omitted for clarity.

Complexes **4** and **5** have been fully characterized by means of elemental analysis, HR-MS and multinuclear NMR spectroscopy, and in both cases, it has been possible to establish their solid-state structure by X-ray diffraction analysis (Figures 1 and 2). The most noticeable resonance in the ^1^H NMR spectra of **4** and **5** species is a doublet at δ −20.87 ppm (^2^*J*_HP_ = 18.8 Hz) (**4**) and −29.18 ppm (^2^*J*_HP_ = 19.2 Hz) (**5**) (Figures S31 and S37). The ^29^Si chemical shifts obtained by ^1^H–^29^Si HMBC NMR experiment δ 28.2 ppm (**4**) and 29.0 ppm (**5**) are high field shifted in comparison with those found for **2** (Figures S36 and S43) with a less electron-rich metal center as a consequence of the Ir(d_π_)-backbonding to the coe ligand. The first evidence for an Ir···H–C agostic interaction^32−35^ in **2**, **3**, **4** and **5** came from the ^1^H–^29^Si HMBC NMR experiments, which show a clear correlation between the silicon atom and the protons of the 8-Me substituent of the NSi^DMQ^ ligand. Moreover, as should be expected for a weak Ir···H–C agostic interaction,^32a,35,36^ the ^1^*J*_CH_ values found for the 8-Me 124.9 Hz (**2**), 123.2 Hz (**3**), 123.9 Hz (**4**) and 121.5 Hz (**5**) are lower than those found for the proligand **1** (127.2 Hz, Figure S68) and for the 4-Me in **2** (128.1 Hz, Figure S69), **3** (128.8 Hz, Figure S70), **4** (128.2 Hz, Figure S71) and **5** (128.2 Hz, Figure S72).

The solid-state structures of **4** and **5** (Figures 1 and 2) exhibit a square pyramidal geometry with the silicon atom at the apical position and the nitrogen atom *trans* located to the phosphorus atom, whose geometrical parameters are reported in Table 1. The Ir–Si bond distances 2.2529(4) Å (**4**) and 2.2687(5) Å (**5**) are slightly shorter than those reported for the related 2-pyridone derivatives [Ir(H)(X)(κ^2^-NSi^tBu2^)(PCy_3_)] (X = Cl; X = OTf), which are around 2.28 Å.^30b^ In agreement with the Ir···H–C agostic interaction observed by ^1^H–^29^Si HMBC and ^13^C NMR experiments, the solid-state structures of **4** and **5** show the presence of a close carbon atom, occupying the coordination vacancy located *trans* to the silicon atom. Observed geometrical parameters (d(Ir···H) 2.20(2) Å and 2.17(3) Å; and Ir···H–C angles 118.0(16)° and 112.5(18)° in **4** and **5**, respectively) are within the ranges typically defined for agostic interactions d(Ir···H) ≈ 1.8–2.3 Å and M···H–C ≈ 90–140°.^32a^

**Table 1:** Selected Bond Lengths (Å) and Angles (deg) for Complexes **4**, **5**, and **10**

	4	5	10
Ir(1)–X^a^	2.4556(4)	2.2533(14)	2.2263(11)
Ir(1)–P(1)	2.2766(4)	2.2839(5)	2.2715(3)
Ir(1)–Si(1)	2.2529(4)	2.2687(5)	2.2522(4)
Ir(1)–N(1)	2.1399(11)	2.1106(15)	2.1068(11)
Ir(1)–H(1)	1.50(3)	1.589(18)	1.544(15)
X–Ir(1)–P(1)	94.888(13)	96.91(4)	91.82(3)
X–Ir(1)–Si(1)	97.300(14)	95.40(4)	102.07(3)
X–Ir(1)–N(1)	90.95(3)	87.25(5)	92.52(4)
X–Ir(1)–H(1)	177.2(8)	177.0(12)	175.9(8)
P(1)–Ir(1)–Si(1)	98.483(14)	101.104(17)	103.837(13)
P(1)–Ir(1)–N(1)	173.77(3)	173.66(4)	171.96(3)
P(1)–Ir(1)–H(1)	87.5(9)	85.9(12)	86.5(8)
Si(1)–Ir(1)–N(1)	82.89(3)	83.20(4)	81.86(3)
Si(1)–Ir(1)–H(1)	83.9(9)	83.0(12)	81.9(8)
N(1)–Ir(1)–H(1)	86.7(9)	90.0(12)	88.7(8)

a **4**, X = Cl(1); **5**, X = O(2); **10**, X = O(2).

**Figure 3:**
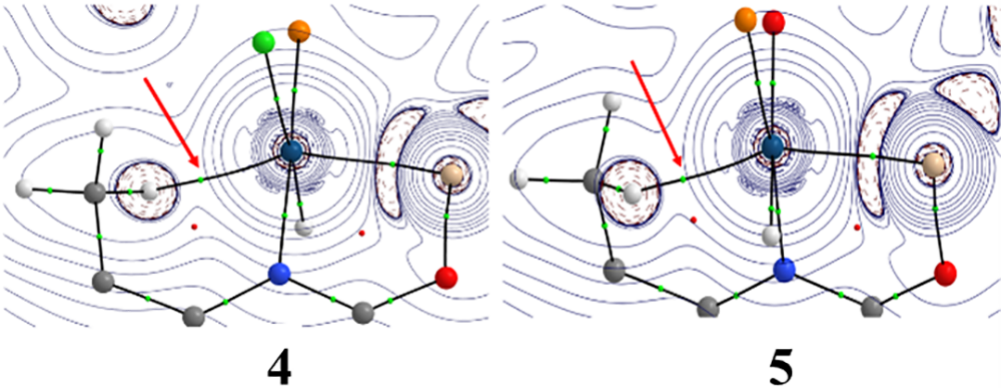
Contour line diagrams ∇^2^ρ(r) for complexes **4** (left) and **5** (right) in the Ir···H–C plane. Green and red spheres denote the located bond critical points (BCP) and ring critical points (RCP), respectively. The red arrow indicates the position of the Ir···H–C BCP in both complexes.

The formation of an agostic M···H–C interaction requires the proximity of a C–H bond to an unsaturated 14 or 16-electron TM–complex. Agostic M···H–C interactions typically involve the donation of electron density from the doubly occupied σ(C–H) molecular orbital to empty d orbitals of the metal, and often, also backbonding from d orbitals of the metal to the antibonding σ*(C–H) molecular orbital.^32−34^ Density Functional Theory (DFT) calculations at the dispersion corrected RI-BP86-D3/def2-TZVP level were carried out to gain more insight into the Ir···H–C agostic interaction in these complexes. The computed Ir···H bond distances (2.087 and 2.053 Å, for **4** and **5**, respectively) suggest that the interaction is somewhat stronger in **5**. In both species, the Quantum Theory of Atom in Molecules (QTAIM) method locates a bond critical point (BCP) between the H and Ir centers and a bond path (BP) running between both atoms, which confirms the occurrence of the proposed agostic interaction (Figure 3). Interestingly, all the QTAIM values computed at the BCP clearly indicate that the agostic interaction is stronger in **5** than in **4** (Table 2). For instance, the delocalization index (DI), which is directly related to the bond strength, is slightly higher in **5**. Moreover, the Natural Orbital Bond (NBO) method indicates that the agostic interaction in these complexes is established by the donation of electron density from the doubly occupied σ(C–H) bond of the 8-Me moiety to a vacant d atomic orbital of the metal. Not surprisingly, the associated second-order perturbation energy (Δ*E*^(2)^) is more stabilizing for **5** (−9.01 kcal mol^–1^) than for **4** (−6.99 kcal mol^–1^) therefore confirming a stronger interaction. This is very likely due to the higher electron-withdrawing ability of the triflate ligand as compared to chloride, which enhances the acceptor properties of the iridium center.

**Table 2:** Results of the QTAIM and NBO Analysis for **4** and **5**

	4	5
r(Ir···H)/Å^a^	2.087 (2.20(2))	2.053 (2.17(3))
**ρ**(r)/e Å^–3^	0.039	0.042
**∇**^2^**ρ**(r)/e Å^–5^	0.135	0.155
**ε**	0.286	0.37
DI	0.165	0.176
**ΔΕ**^**(2)**^**/σ(**C–H**)**→**δ(**Ir**)**/kcal mol^–1^	–6.99	–9.01

a Values within parentheses refer to the experimental bond distances.

Therefore, the Ir···H–C interaction in **4** and **5** can be described as a weak agostic interaction (Table 2). The ^1^*J*_CH_ coupling constant of the 8-Me substituent in the ^13^C NMR spectra of **4** and **5** is higher than expected for an agostic Ir···H–C interaction. This can be explained on the basis of the rotation of the 8-Me group, which could not be frozen out even at 193 K. As a result, the ^1^*J*_CH_ coupling constants observed for the 8-Me subtituent in the ^13^C NMR correspond to averaged values. This is due to the decreases in the ^1^*J*_CH_ coupling constant of the coordinated C–H bond while the ^1^*J*_CH_ coupling constants of the two uncoordinated C–H bonds increase.^36^

### Ir-NSi Catalyzed Cross-Dehydrocoupling of Amines with Hydrosilanes

Once the catalytic precursors **2**, **3**, **4** and **5** were characterized, we decided to explore their activity as catalysts for the CDC of amines with hydrosilanes. ^1^H NMR (C_6_D_6_) studies of the reaction of pyrrolidine with HSiMe_2_Ph were used as a benchmark to evaluate the activity of **2**, **3**, **4** and **5** (1 mol %) as catalysts for the CDC of secondary amines with tertiary silanes. The best catalytic performance was achieved when using complex **5**, with triflate and PCy_3_ ancillary ligands, as catalyst precursor (Table 3, Entry 4). The higher catalytic activity of the Ir–OTf species **3** and **5** compared to their Ir–Cl counterparts **2** and **4**, respectively (Table 3), suggests a noninnocent role for the triflate ligand. A similar behavior has been previously observed in hydrosilylation and silylation processes catalyzed by Ir-(κ^2^-NSi) species,^27b,29,30b^ which may be related to the ability of the triflate ligand to assist in the Si–H bond activation step.^37^ On the other hand, the superior activity of complex **5** compared to complex **3**, both Ir–OTf derivatives, can be attributed to the robustness of the Ir–PCy_3_ bond in **5**, which stabilizes active intermediates. In contrast, the lower activity of complex **3** is likely due to catalyst decomposition during the process, where both the dissociation of coe ligand and formation of coa (cycloctane) during the catalysis are observed.

**Table 3:**
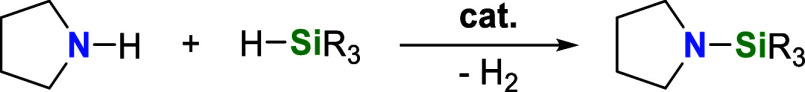
Comparison of the Activity of **2**, **3**, **4**, and **5** as Catalysts for the CDC of Pyrrolidine (0.3 mmol) with Different Hydrosilanes (0.3 mmol), and Influence of the Hydrosilane on the Activity of **5**^a^

entry	cat	hydrosilane	time (h)	conv (%)^b^
1	**2**	HSiMe_2_Ph	3	23
2	**3**	HSiMe_2_Ph	3	48
3	**4**	HSiMe_2_Ph	3	38
4	**5**	HSiMe_2_Ph	3	92
5	**5**	HSiMePh_2_	3	38
6	**5**	HSiMe(OSiMe_3_)_2_	3	10
7	**5**	HSiEt_3_	3	1

a Reactions in C_6_D_6_ (0.4 mL) at r.t. using 1 mol % catalyst loading.

b Conversion based on ^1^H NMR integration using hexamethylbenzene as an internal standard (IS).

The nature of the tertiary silane also has a clear influence on the activity (Table 3, Entries 4–7). Thus, ^1^H NMR (C_6_D_6_) studies of the **5** (1 mol %) catalyzed reaction of pyrrolidine with HSiEt_3_, HSiMe(OSiMe_3_)_2_, HSiMePh_2_ and HSiMe_2_Ph at r.t. after 3 h show a conversion of 1%, 10%, 38% and 92%, respectively. These results imply that the steric hindrance around the Si–H bond has a significant influence on the reaction performance.

**Figure 4:**
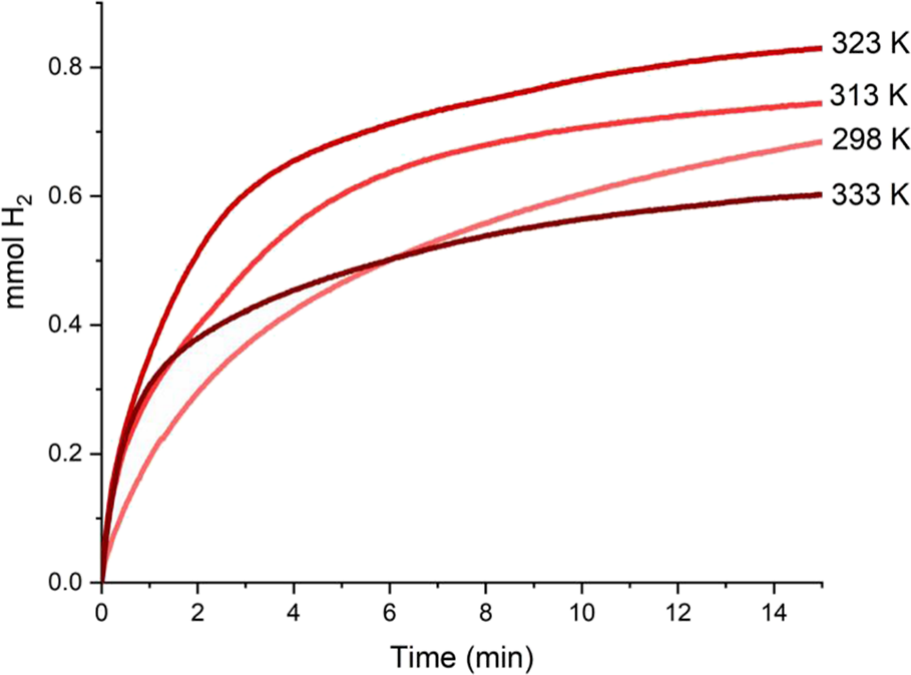
Time profile of H_2_ (mmol) generation from the **5**-catalyzed (1 mol %) reaction of pyrrolidine (1 mmol) with HSiMe_2_Ph (1 mmol) at different temperatures under neat conditions.

These findings encouraged us to study the potential of **5** as catalyst for the CDC of secondary amines with HSiMe_2_Ph, for which we decided to perform the reactions under neat conditions in a microreactor equipped with a pressure sensor.^38^ Initially, we explored the effect of the temperature on the **5**-catalyzed (1 mol %) CDC of pyrrolidine with HSiMe_2_Ph. These studies evidenced that increasing the temperature from 298 to 323 K produces a positive effect on the catalytic activity of the system based on **5** (Figure 4 and Table S4). However, at 333 K a decrease of the activity was observed after 2 min of reaction. This decrease in activity upon heating above 323 K might be ascribed to a catalyst deactivation process occurring above this temperature. It is worth mentioning that ^1^H NMR studies show that solutions of **5** in CD_2_Cl_2_ remain stable at 333 K for 24 h. Therefore, the decomposition of the catalyst must be attributed to the decomposition of catalytic intermediates.

**Scheme 4:**
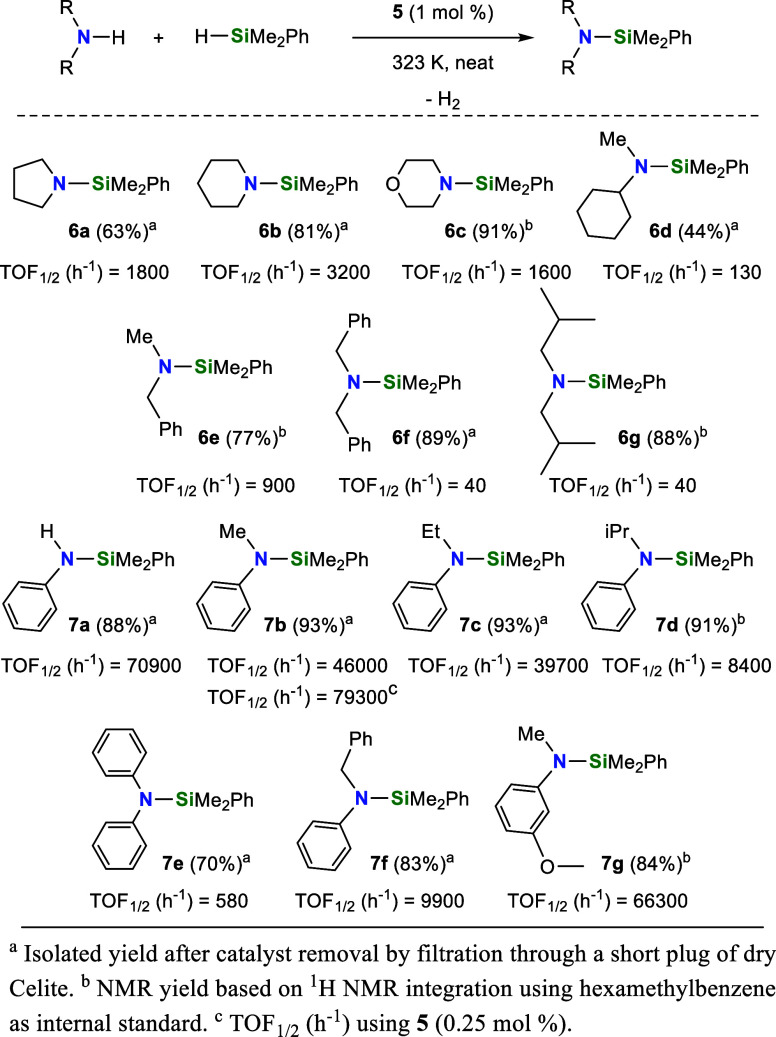
**5**-Catalyzed (1 mol %) CDC of Secondary Amines with HSiMe_2_Ph at 323 K under Neat Conditions, TOF_1/2_ (h^–1^) = TOF (h^–1^) at 50% Conversion

**Figure 5:**
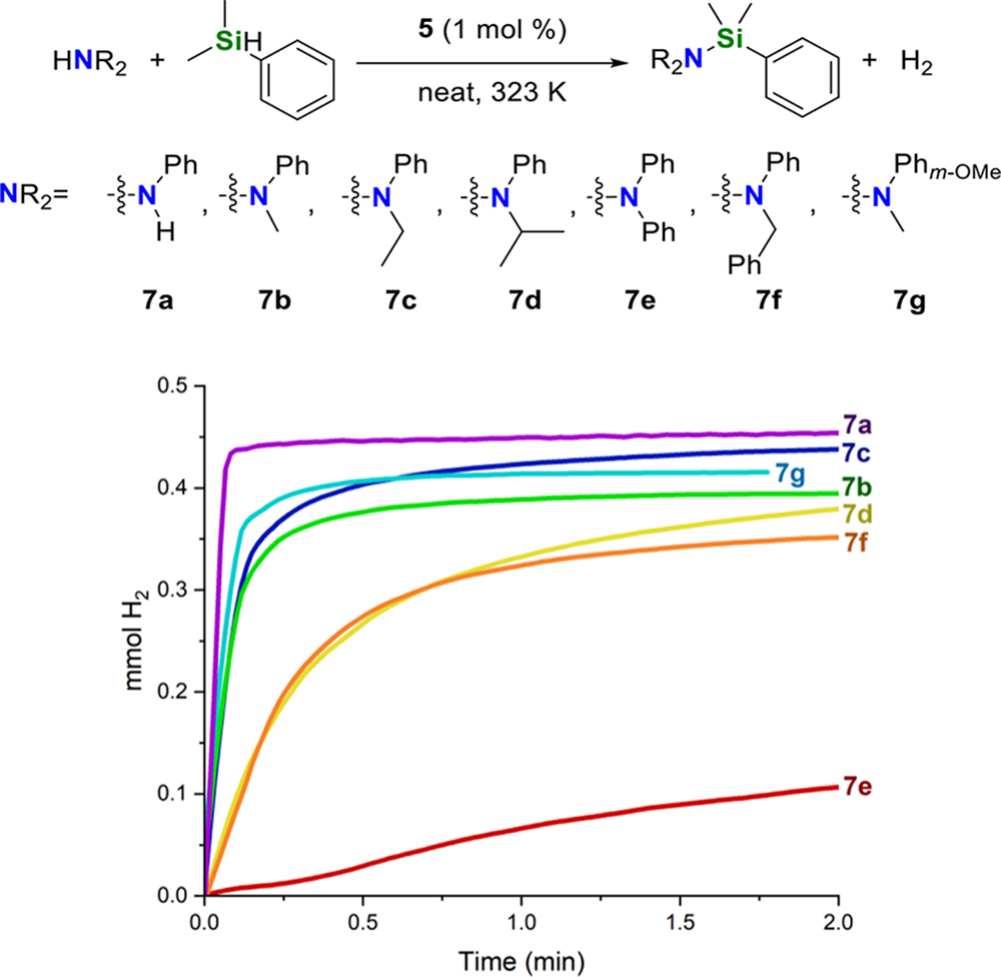
Time profile of H_2_ (mmol) generation from the **5-**catalyzed (1 mol %) reaction of different aniline derivatives (0.5 mmol) with HSiMe_2_Ph (0.5 mmol) at 323 K under neat conditions.

Once the best working temperature (323 K) was established, we decided to extend the scope of our study to different secondary amines. The results of these studies show that **5** (1 mol %) is an active catalyst in all the studied cases (Scheme 4). To our delight, we found that **5** exhibits higher catalytic activity for *N*-alkylaniline derivatives compared to aliphatic secondary amines (Scheme 4 and Figure 5). The lower TOF values found for *N*-benzylaniline, *N*-isopropylaniline and diphenylamine in comparison with *N*-methylaniline prove that the activity of **5** depends on the steric hindrance of the N–H bond. Moreover, the higher activity found for aniline, *N*-ethylaniline and 3-methoxy-*N*-methylaniline in comparison with *N*-methylaniline indicates that the electronic richness of the nitrogen atom also plays a role in the catalytic activity of **5** (Scheme 4 and Figure 5).

On the other hand, since the **5**-catalyzed reaction of aniline with HSiMe_2_Ph is selective toward the formation of **7a**, we decided to explore the selectivity of this system with the primary silane H_3_SiPh. **5** was also an active catalyst for the CDC reaction of *N*-methylaniline with H_3_SiPh (TOF_1/2_ = 15,400 h^–1^; TOF_final_ = 500 h^–1^; Figure S10). However, in this case, the reaction is not selective, yielding a mixture of H_2_PhSi(NMePh) (**7h-1**) (80.6%) and HPhSi(NMePh)_2_ (**7h-2**) (19.4%) (Figures S139–S142).

These results demonstrate that the Ir-(κ^2^-NSi) catalytic precursors described in this work are more active and versatile catalysts than the Ir-(κ^3^-NSiN) species previously described by our group.^20b^ However, their activity in the case of aliphatic amines is lower than that reported for cationic Pt(II) species.^21^ Examples of catalytic systems that similarly to **5** promote the CDC of amines with hydrosilanes, where aniline derivatives undergo silylation very efficiently, are also known (Table 4).^4a,17,40^ It is worth mentioning that aniline derivatives are typically less reactive than aliphatic amines due to differences in basicity and nucleophilicity between aromatic and aliphatic amines. This behavior can be attributed to several factors, the most prominent being: (i) in anilines, the nitrogen lone pair is partially delocalized into the aromatic ring through resonance, reducing the electron density on the nitrogen atom and thus making it less nucleophilic; and (ii) because of this electron delocalization, the nitrogen in anilines is also less basic compared to aliphatic amines.^39^

**Table 4:** Comparison of the Activity of **5** and Other Compounds as Catalysts for the CDC of *N*-methylaniline with HSiMe_2_Ph

entry	cat.	mol % cat.	solvent	*T* (K)	TO*F*_1/2_ (h^–1^)	TOF_final_ (h^–1^)	ref
1	**5**	1	neat	323	46,000	3900	this work
2	**5**	0.25	neat	323	79,300	6800	this work
3	**10**	1	neat	323	740	520	this work
4	**11**	1	neat	323	1690	960	this work
5	[Ru–S]	1	Hex.	r.t.		1100^c^	(17a)
6	[Ru_3_(CO)_12_]^a^	1	Tol.	353		25^d^	(17b)
7	[RhCl(PPh_3_)_3_]^b^	0.2	neat	323		123^d^	(40)

a With HSiEt_3_.

b With H_2_SiEt_2_.

c TOF at 5 min.

d TOF at 4 h.

**Figure 6:**
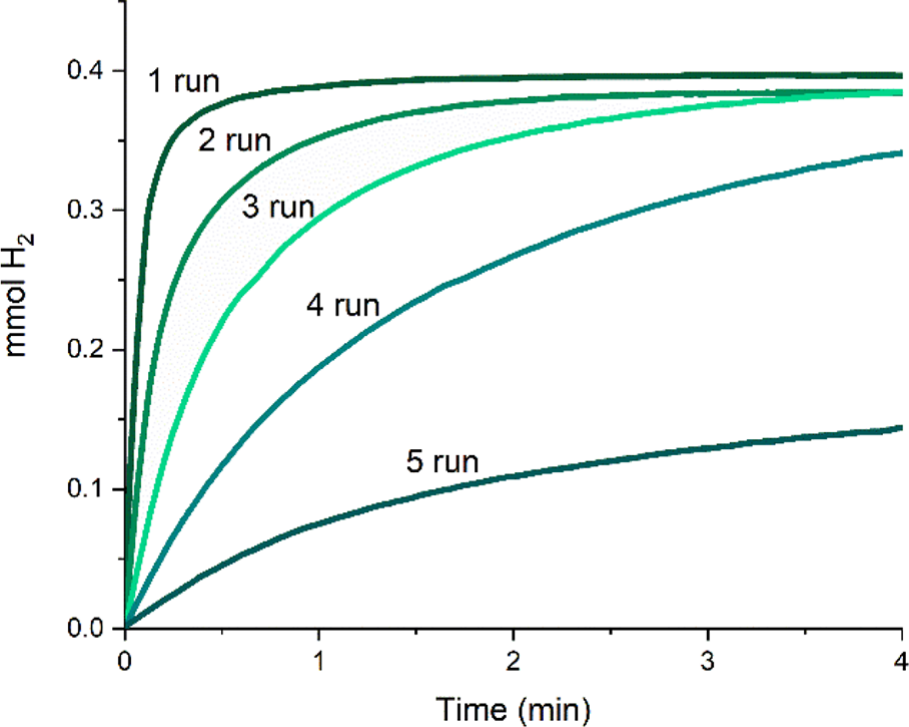
Time profile of H_2_ (mmol) generation from the **5-**catalyzed (1.0 mol %) reaction of *N*-methylaniline (0.5 mmol) with HSiMe_2_Ph (0.5 mmol) at 323 K under neat conditions.

It is remarkable that by reducing the catalyst loading of **5** up to 0.25 mol % an enhancement of the activity was observed (TOF_1/2_ = 79,300 h^–1^, Table 4, Entry 2). This result shows that **5** operates efficiently at low catalyst loading. In addition, reusability studies under these conditions, 0.25 mol % of **5**, show that after reaction completion (1 run), the catalyst can be reused up to 4 more runs reaching full conversion in all cases (Figure 6 and Table S10). Even though longer reaction times are needed in each run, this is likely attributable to partial catalyst decomposition after each run, as it has been proven that dilution does not affect the catalyst performance (Figure S9).

Furthermore, to explore the applicability and utility of this method, we provide an example of reaction scale-up by running the reaction on a gram scale. Thus, the reaction of *N*-methylaniline (4 mmol) with HSiMe_2_Ph (4 mmol) and 0.25 mol % of **5** gave **7b**, after 10 min, in 93% isolated yield demonstrating the reproducibility and scalability of this catalytic process.

Labeling studies show that there is no observable kinetic isotopic effect (KIE) when the **5-**catalyzed CDC reactions were performed using DSiMe_2_Ph and/or *N*-methylaniline-*d*_1_, evidencing that neither the Si–H nor N–H bond activations are the rate-limiting steps of the catalytic process (Figures S11 and S12). The above-mentioned absence of KIE and the results from the catalytic studies (Scheme 4), which show that not only the steric hindrance of the N–H bond, but also electronic factors play a relevant role in the catalytic performance, suggest that the coordination of the amine to the iridium is a key step of the process.

**Scheme 5:**
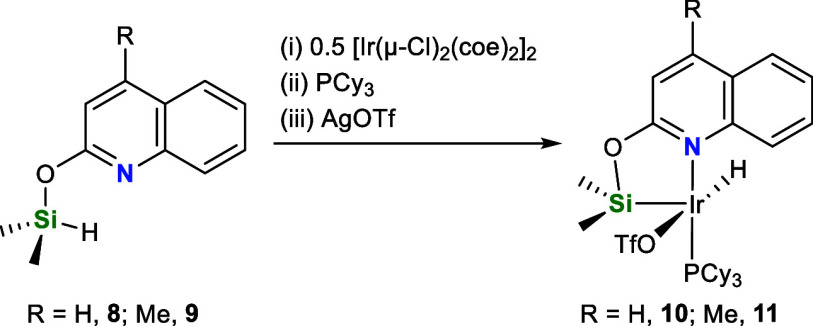
Synthesis of Complexes **10** and **11**

At this point, a question arises: does the occurrence of the Ir···H–C agostic interaction in **5** plays indeed a role in its high activity? To unveil this question the proligands (quinoline-2-yloxy)dimethylsilane (**8**) and (4-methylquinoline-2-yloxy)dimethylsilane (**9**) lacking a 8-Me substituent were synthesized and used, following a similar synthetic procedure to that described for **5**, to prepare the species [Ir(H)(OTf)(κ^2^-NSi^Q^)(PCy_3_)] (**10**, NSi^Q^ = {quinoline-2-yloxy}dimethylsilyl) and [Ir(H)(OTf)(κ^2^-NSi^MQ^)(PCy_3_)] (**11**, NSi^MQ^ = {4-methylquinoline-2-yloxy}dimethylsilyl), which strongly resemble **5** but lack any Ir···H–C agostic interaction (Scheme 5).

**Figure 7:**
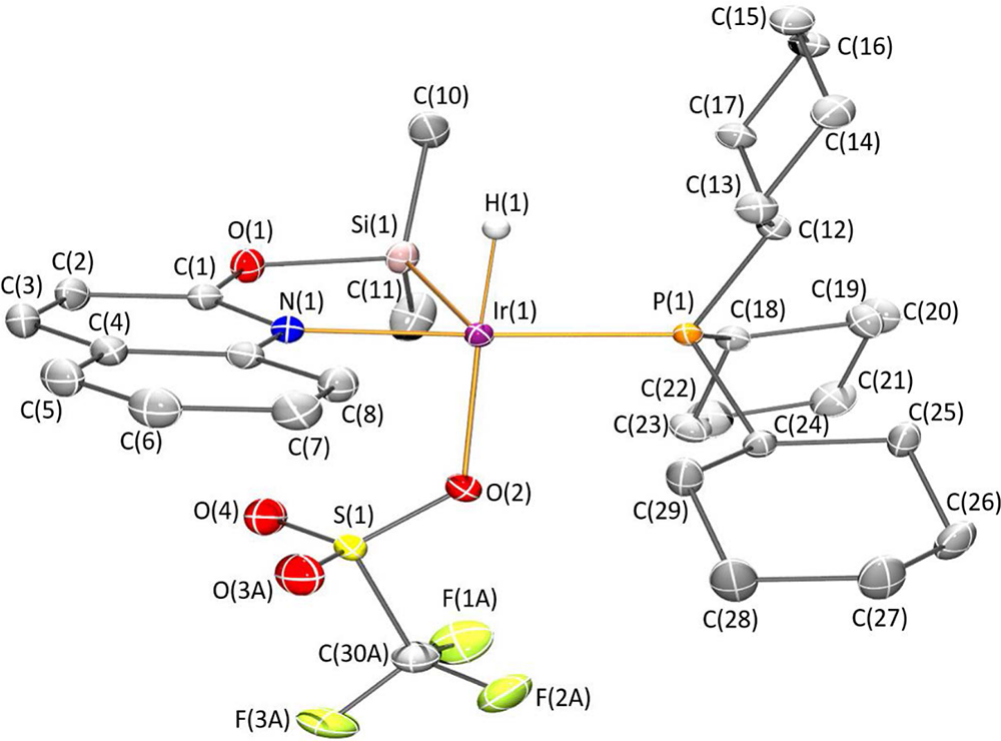
Molecular structure of **10**. Hydrogen atoms (except hydride) and minor components of disordered triflate are omitted for clarity.

Complexes **10** and **11** have been isolated as yellow and dark-green solids in 41% and 91% yield, respectively. Both complexes have been characterized by NMR spectroscopy and HR-MS, and in the case of **10** it has been possible to determine its solid-state structure by X-ray diffraction. The solid-state structure of **10** (Figure 7) exhibits a square pyramidal geometry with the silicon atom at the apical position and the nitrogen atom *trans* located to the phosphorus atom, whose geometrical parameters are reported in Table 1. The Ir–Si bond distance in **10** (2.2522(4) Å) compares to that found for **4** and **5** (Table 1). The ^1^H NMR spectrum of **10** and **11** shows the Ir–H resonance as a doublet centered at δ −28.03 (^2^*J*_HP_ = 17.8 Hz) and −27.88 ppm (^2^*J*_HP_ = 17.7 Hz), respectively, slightly low field shifted in comparison to **5**. The ^29^Si resonance in **10** and **11** is observed, in both cases, at δ 26.9 ppm slightly high field shifted in comparison to **5** (δ 29.0 ppm).

**Figure 8:**
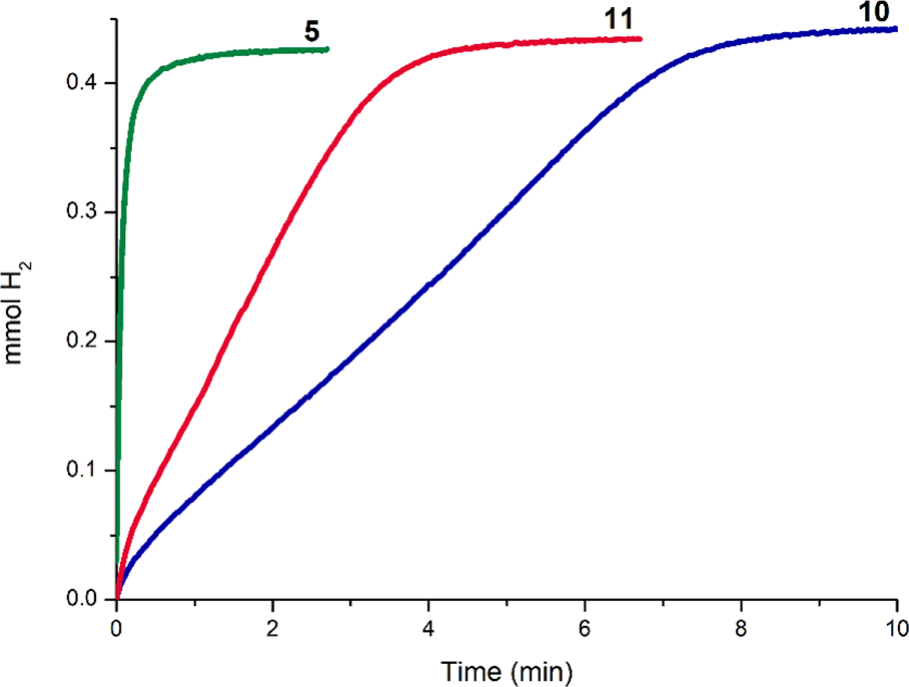
Time profile of H_2_ (mmol) generation from the **5**-, **10**-, and **11**-catalyzed (1 mol %) reaction of *N*-methylaniline (0.5 mmol) with HSiMe_2_Ph (0.5 mmol) at 323 K under neat conditions.

Studies on the **10**- and **11**-catalyzed (1 mol %) CDC of *N*-methylaniline with HSiMe_2_Ph evidenced that the systems based on **10** and **11** exhibit a comparatively lower activity than **5** (Table 4, Entries 3 and 4, and Figure 8). In the case of the reaction of HSiMe_2_Ph with pyrrolidine, the activity of the system based on **5**, with an 8-Me substituent, is also higher than that of the systems based on **10** and **11** though not as pronounced (Figure S14). These results suggest that the presence of the 8-Me substituent plays a crucial role in the **5**-catalyzed reaction of *N*-methylaniline with HSiMe_2_Ph. Moreover, the higher activity of **11** in comparison to **10** shows that the presence of the 4-Me also influences the catalytic activity. Further studies are currently being performed in our laboratories to elucidate the reaction mechanism involved in this transformation. Reusability studies show that the catalytic system formed by **10** (or **11**) and HSiMe_2_Ph can be reused for the silylation of *N*-methylaniline at least five cycles. However, a noticeable decrease in activity is observed after each use (Figures S6 and S7).

## Conclusions

The iridium(III) derivatives [Ir(H)(X)(κ^2^-NSi^DMQ^)(L)] (NSi^DMQ^ = {4,8-dimethylquinoline-2-yloxy}dimethylsilane; L = coe, X = Cl, **2**; L = coe, X = OTf, **3**; L = PCy_3_, X = Cl, **4**; L = PCy_3_, X = OTf, **5**) and the species [Ir(H)(OTf)(κ^2^-NSi^Q^)(PCy_3_)] (**10**, NSi^Q^ = {quinoline-2-yloxy}dimethylsilyl) and [Ir(H)(OTf)(κ^2^-NSi^MQ^)(PCy_3_)] (**11**, NSi^MQ^ = {4-methylquinoline-2-yloxy}dimethylsilyl) have been prepared and fully characterized. Complexes **2**, **3**, **4** and **5** are stabilized by a weak yet noticeable Ir···H–C agostic interaction between the iridium and one of the C–H bonds of the 8-Me substituent of the NSi^DMQ^ ligand. All these complexes have shown to be active as catalyst precursors for the CDC of amines with hydrosilanes under neat conditions, with the activity of **5** being considerably greater than that shown by **2**, **3** or **4**. The greater catalytic activity observed for the Ir–OTf derivatives **3** and **5** compared to their Ir–Cl counterparts **2** and **4**, respectively, suggests a noninnocent role for the triflate ligand.

Complex **5** is a versatile and highly active catalyst precursor for the CDC of secondary aliphatic amines with HSiMe_2_Ph. Moreover, its activity is comparable to that of the most active catalysts reported for this transformation and can be applied also to the much more challenging *N*-alkylaniline derivatives. Furthermore, **5** operates at low catalyst loading (0.25 mol %) under neat and mild reaction conditions, can be reused and applied in gram-scale reactions. These results confirm that the presence of the 8-Me substituent plays a role in the catalytic activity of **5** in comparison to the related species **10** and **11**.

## Experimental Section

### General Information

All manipulations were performed with rigorous exclusion of air at an argon/vacuo manifold using standard Schlenk-tube or glovebox techniques. Solvents were dried by the usual procedures and distilled under argon prior to use or obtained oxygen- and water-free from a Solvent Purification System (Innovative Technologies). ^1^H, ^13^C{^1^H}, ^13^C APT, ^13^C, ^1^H–^13^C HSQC, ^1^H–^13^C HMBC, ^31^P{^1^H}, ^31^P, ^1^H–^29^Si HMBC, ^1^H–^29^Si HMQC and ^19^F NMR spectra were recorded on a Bruker ARX, Bruker Avance 300 MHz and Bruker Avance 400 MHz instrument. Coupling constants *J* are given in hertz (Hz) (multiplicity: s = singlet, d = doublet, dd = double doublet, ddd = doublet of doublets of doublets, psd = pseudo doublet, sept = septuplet, m = multiplet, br = broad signal). The “Brief Guide to the Nomenclature of Organic Chemistry” was followed for signal assignment.^41^ [{Ir(coe)_2_}_2_(μ-Cl)_2_]^42^ was prepared following the reported methodology. The secondary amines and hydrosilanes were purchased from commercial sources and dried on 4 Å molecular sieves prior to use.

**Caution!** In some cases, **5**-catalyzed reactions of amines with hydrosilanes were found to vigorously generate hydrogen. Therefore, appropriate precautions must be taken.

### Synthesis of 4,8-Dimethylquinoline-2-oxy-dimethylsilane (**1**)

NEt_3_ (201 μL, 1.44 mmol) was added to a THF (10 mL) solution of 4,8-dimethyl-2-hydroxyquinoline (200 mg, 1.15 mmol). The resulting mixture was stirred at room temperature (r.t.) for 2 h. After that, the solution was cooled to 0 °C, prior to the slow addition (via cannula) of a THF (3 mL) solution of HSiMe_2_Cl (141 μL, 1.27 mmol). After that, the reaction mixture was warmed to r.t. and stirred at 60 °C for 20 h. The resulting suspension was cooled to r.t. The THF solution was filtered, and the off-white residue extracted with hexane (3 × 5 mL). The THF and hexane fractions were mixed and brought to dryness to give a white solid, which was extracted with hexane (1 × 2 mL) to give **1** as a white solid in 74% yield (197 mg, 0.85 mmol). ^1^H NMR (300 MHz, 298 K, C_6_D_6_): δ 7.48 (psd, ^3^*J*_HH_ = 8.3 Hz, 1H, *H*^5^), 7.34 (psd, ^3^*J*_HH_ = 7.0 Hz, 1H, *H*^7^), 7.15 (dd, ^3^*J*_HH_ = 8.2 Hz, ^3^*J*_HH_ = 7.4 Hz, 1H, *H*^6^), 6.67 (m, 1H, *H*^3^), 5.43 (sept, ^3^*J*_HH_ = 2.9 Hz, 1H, Si–*H*), 2.78 (s, 3H, 8-C*H*_3_), 2.09 (d, ^4^*J*_HH_ = 1.0 Hz, 3H, 4-C*H*_3_), 0.53 (d, ^3^*J*_HH_ = 2.9 Hz, 6H, Si-(C*H*_3_)_2_). ^13^C{^1^H} NMR (75 MHz, 298 K, C_6_D_6_): δ 160.3 (s, *C*^ipso-2^), 148.2 (s, *C*^ipso-8a^), 146.2 (s, *C*^ipso-4^), 135.9 (s, *C*^ipso-8^), 130.0 (s, *C*^7^), 125.5 (s, *C*^ipso4a^), 123.7 (s, *C*^6^), 121.9 (s, *C*^5^), 114.1 (s, *C*^3^), 18.7 (s, 4-*C*H_3_) 18.6 (s, 8-*C*H_3_), −1.1 (s, 2C, Si-(*C*H_3_)_2_). ^29^Si from ^1^H–^29^Si HMBC NMR (60 MHz, 298 K, C_6_D_6_): δ 3.3 (s).

### Synthesis of [Ir(H)(Cl)(κ^2^-NSi^DMQ^)(coe)] (**2**)

To a mixture of **1** (266.4 mg, 1.15 mmol) and [{Ir(coe)_2_}_2_(μ-Cl)_2_] (516.0 mg, 0.58 mmol) dichloromethane (10 mL) was added. The resulting solution was stirred at r.t. for 2 h. After this time, the mixture was brought to dryness to give a pale-brown residue, which was washed with hexane (3 × 5 mL) to afford **2** as a pale-yellow solid in 78% yield (510.5 mg, 0.89 mmol). ^1^H NMR (300 MHz, 298 K, CD_2_Cl_2_): δ 7.81 (dd, ^3^*J*_HH_ = 8.2 Hz, ^4^*J*_HH_ = 1.4 Hz, 1H, *H*^5^), 7.48 (dd, ^3^*J*_HH_ = 7.1 Hz, ^4^*J*_HH_ = 1.4 Hz, 1H, *H*^7^), 7.36 (dd, ^3^*J*_HH_ = 8.2 Hz, ^3^*J*_HH_ = 7.1 Hz, 1H, *H*^6^), 7.02 (m, 1H, *H*^3^), 4.15 (m, 1H, C*H*-coe), 3.72 (m, 1H, C*H*-coe), 2.81 (m, 1H, C*H*_*2*_-coe), 2.74 (s, 3H, 8-C*H*_3_), 2.67 (s, 3H, 4-C*H*_3_), 2.49 (m, 1H, C*H*_*2*_-coe), 1.95–1.25 (overlapping signals, 10H, C*H*_*2*_-coe), 0.53 (s, 3H, Si–C*H*_3_), 0.49 (s, 3H, Si–C*H*_3_), −16.64 (s, 1H, Ir–*H*). ^13^C{^1^H} NMR (75 MHz, 298 K, CD_2_Cl_2_): δ 165.0 (s, *C*^ipso-2^), 151.5 (s, *C*^ipso-8a^), 145.5 (s, *C*^ipso-4^), 134.7 (s, *C*^ipso-8^), 132.5 (s, *C*^7^), 126.7 (s, *C*^ipso-4a^), 124.8 (s, *C*^6^), 124.2 (s, *C*^5^), 114.2 (s, *C*^3^), 65.6 (s, *C*H-coe), 62.5 (s, *C*H-coe), 33.6, 33.3, 32.3, 29.0, 27.1, and 26.7 (s, 6× *C*H_2_-coe), 20.0 (s, 4-*C*H_3_), 17.7 (s, 8-*C*H_3_), 7.6 (s, Si-*C*H_3_), 2.2 (s, Si-*C*H_3_). ^29^Si from ^1^H–^29^Si HMBC NMR (60 MHz, 298 K, CD_2_Cl_2_): δ 32.8 (s). Anal. Calcd for C_21_H_31_ClIrNOSi: C, 44.31; H, 5.49; N, 2.46, found C, 44.32; H, 5.29; N, 2.61. HRMS (ESI^+^, *m*/*z*): calcd for C_21_H_31_IrNOSi, [M – Cl]^+^ = 534.1804; found = 534.1777.

### Synthesis of [Ir(H)(OTf)(κ^2^-NSi^DMQ^)(coe)] (**3**)

To a solution of **2** (292.5 mg, 0.50 mmol) and AgTfO (128.6 mg, 0.50 mmol) dichloromethane (10 mL) was added in the absence of light. After 15 h of stirring at r.t., the obtained suspension was filtered through Celite with a cannula. The solution is brought to dryness to obtain **3** as a pale-yellow solid in 90% yield (307 mg, 0.45 mmol). ^1^H NMR (300 MHz, 298 K, CD_2_Cl_2_): δ 7.86 (dd, ^3^*J*_HH_ = 8.3 Hz, ^4^*J*_HH_ = 1.5 Hz, 1H, *H*^5^), 7.56 (ddd, ^3^*J*_HH_ = 7.2 Hz, ^4^*J*_HH_ = 1.6 Hz, ^4^*J*_HH_ = 0.9 Hz, 1H, *H*^7^), 7.41 (dd, ^3^*J*_HH_ = 8.2 Hz, ^3^*J*_HH_ = 7.2 Hz, 1H, *H*^6^), 7.07 (d, ^4^*J*_HH_ = 1.1 Hz, 1H, *H*^3^), 4.54 (m, 1H, C*H*-coe), 4.15 (m, 1H, C*H*-coe), 3.00 (s, 3H, 8-C*H*_3_), 2.70 (s, 3H, 4-C*H*_3_), 2.55–1.24 (m, 12H, C*H*_*2*_-coe), 0.67 (s, 3H, Si–C*H*_3_), 0.60 (s, 3H, Si–C*H*_3_), −25.78 (s, 1H, Ir–*H*). ^13^C{^1^H} NMR (75 MHz, 298 K, CD_2_Cl_2_): δ 166.0 (s, *C*^ipso-2^), 152.9 (s, *C*^ipso-8a^), 145.5 (s, *C*^ipso-4^), 134.7 (s, *C*^ipso-8^), 131.4 (s, *C*^7^), 126.6 (s, *C*^ipso-4a^), 125.0 (s, *C*^6^), 124.4 (s, *C*^5^), 114.1 (s, *C*^3^), 73.7 (s, *C*H-coe), 70.9 (s, *C*H-coe), 33.0, 32.8, 31.7, 28.6, 26.9, and 26.6 (s, 6x *C*H_2_-coe), 20.1 (s, 4-*C*H_3_), 19.1 (s, 8-*C*H_3_), 7.4 (s, Si-*C*H_3_), 1.4 (s, Si-*C*H_3_). ^29^Si from ^1^H–^29^Si HMBC NMR (60 MHz, 298 K, CD_2_Cl_2_): δ 32.2 (s). ^19^F NMR (282 MHz, 298 K, CD_2_Cl_2_): −79.3 (s, OTf). Anal. Calcd for C_22_H_31_F_3_IrNO_4_SSi: C, 38.70; H, 4.58; N, 2.05, found C, 38.78; H, 4.48; N, 2.15. HRMS (ESI^+^, *m*/*z*): calcd for C_21_H_31_IrNOSi, [M-OTf]^+^ = 534.1804; found = 534.1851.

### Synthesis of [Ir(H)(Cl)(κ^2^-NSi^DMQ^)(PCy_3_)] (**4**)

To a solution of **2** (490 mg, 0.84 mmol) in toluene (12 mL) was added a toluene (5 mL) solution of PCy_3_ (258.7 mg, 0.58 mmol). After 4 days of stirring at r.t., the reaction mixture was brought to dryness and washed with cold hexane (3 × 5 mL) to obtain **4** as a pale-green solid in 72% yield (446 mg, 0.60 mmol). ^1^H NMR (300 MHz, 298 K, CD_2_Cl_2_): δ 7.75 (dd, ^3^*J*_HH_ = 8.2 Hz, ^4^*J*_HH_ = 1.6 Hz, 1H, *H*^5^), 7.46 (dd, ^3^*J*_HH_ = 7.2 Hz, ^4^*J*_HH_ = 1.6 Hz, 1H, *H*^7^), 7.33 (dd, ^3^*J*_HH_ = 8.2 Hz, ^4^*J*_HH_ = 7.2 Hz, 1H, *H*^6^), 6.96 (m, 1H, *H*^3^), 2.86 (s, 3H, 8-C*H*_3_), 2.63 (d, ^4^*J*_HH_ = 1.1 Hz, 3H, 4-C*H*_3_), 2.15 (m, 6H, 3× C*H*_2_-PCy_3_), 1.94 (m, 3H, C*H*-PCy_3_), 1.85 (m, 6H, 3× C*H*_2_-PCy_3_), 1.73 (m, 2H, C*H*_2_-PCy_3_), 1.54 (m, 6H, 3× C*H*_2_-PCy_3_), 1.29 (m, 10H, 5× C*H*_2_-PCy_3_), 0.68 (s, 3H, Si–C*H*_3_), 0.62 (s, 3H, Si–C*H*_3_), −20.87 (d, ^2^*J*_HP_ = 18.8 Hz, 1H, Ir–*H*). ^13^C{^1^H} NMR (75 MHz, 298 K, CD_2_Cl_2_): δ 163.9 (s, *C*^ipso-2^), 149.7 (s, *C*^ipso-8a^), 146.2 (s, *C*^ipso-4^), 133.8 (s, *C*^ipso-8^), 133.5 (s, *C*^7^), 126.8 (s, *C*^ipso-4a^), 124.2 (s, *C*^6^), 123.8 (s, *C*^5^), 114.5 (s, *C*^3^), 37.8 (d, ^2^*J*_CP_ = 29.9 Hz, 3C, *C*H-PCy_3_), 30.2 (br, 3C, *C*H_2_-PCy_3_), 30.1 (d, ^4^*J*_CP_ = 2.7 Hz, 3C, *C*H_2_-PCy_3_), 28.3 (br, 3C, *C*H_2_-PCy_3_), 27.2 (br, 6C, *C*H_2_-PCy_3_), 19.7 (s, 4-*C*H_3_), 18.3 (s, 8-*C*H_3_), 11.1 (s, Si-*C*H_3_), 4.9 (s, Si-*C*H_3_). ^29^Si from the ^1^H–^29^Si HMBC NMR (60 MHz, 298 K, CD_2_Cl_2_): δ 28.2 (s). ^31^P{^1^H} NMR (121 MHz, 298 K, CD_2_Cl_2_): δ 14.1 (s, *P*Cy_3_). Anal. Calcd for C_31_H_50_ClIrNOPSi: C, 50.35; H, 6.82; N, 1.89, found C, 50.26; H, 6.98; N, 2.09. HRMS (ESI^+^, *m*/*z*): calcd for C_31_H_50_IrNOPSi, [M – Cl]^+^ = 704.3029; found = 704.3169.

### Synthesis of [Ir(H)(OTf)(κ^2^-NSi^DMQ^)(PCy_3_)] (**5**)

To a mixture of **4** (385 mg, 0.52 mmol) and AgTfO (133.7 mg, 0.52 mmol) dichloromethane (10 mL) was added in the absence of light. After 15 h of stirring at r.t., the obtained suspension was filtered through Celite with a cannula. The solution is brought to dryness to obtain **5** as a pale-brown solid in 93% yield (409 mg, 0.48 mmol). ^1^H NMR (300 MHz, 298 K, CD_2_Cl_2_): δ 7.80 (dd, ^3^*J*_HH_ = 8.2 Hz, ^4^*J*_HH_ = 1.5 Hz, 1H, *H*^5^), 7.54 (dd, ^3^*J*_HH_ = 7.2 Hz, ^4^*J*_HH_ = 1.5 Hz, 1H, *H*^7^), 7.37 (dd, ^3^*J*_HH_ = 8.2 Hz, ^3^*J*_HH_ = 7.2 Hz, 1H, *H*^6^), 7.02 (m, 1H, *H*^3^), 3.02 (s, 3H, 8-C*H*_3_), 2.66 (d, ^4^*J*_HH_ = 0.6 Hz, 3H, 4-C*H*_3_), 2.08–1.20 (overlapping signals, 33H, C*H* and C*H*_*2*_-PCy_3_), 0.77 (s, Si–C*H*_3_), 0.65 (s, Si–C*H*_3_), −29.18 (d, ^2^*J*_HP_ = 19.2 Hz, 1H, Ir–*H*). ^13^C{^1^H} NMR (75 MHz, 298 K, CD_2_Cl_2_): δ 165.2 (s, *C*^ipso-2^), 150.9 (s, *C*^ipso-8a^), 147.0 (s, *C*^ipso-4^), 134.0 (s, *C*^ipso-8^), 132.6 (s, *C*^7^), 126.6 (s, *C*^ipso-4a^), 124.3 (s, *C*^6^), 124.1 (s, *C*^5^), 114.3 (s, *C*^3^), 38.1 (d, ^2^*J*_CP_ = 29.8 Hz, 3C, *C*H-PCy_3_), 30.1 (br, 3C, *C*H_2_-PCy_3_), 29.7 (br, 3C, *C*H_2_-PCy_3_), 28.3 (d, ^4^*J*_CP_ = 5.7 Hz, 3C, *C*H_2_-PCy_3_), 28.1 (d, ^4^*J*_CP_ = 5.2 Hz, 3C, *C*H_2_-PCy_3_), 27.0 (br, 3C, *C*H_2_-PCy_3_), 19.9 (s, 4-*C*H_3_), 17.8 (s, 8-*C*H_3_), 10.4 (s, Si-*C*H_3_), 3.7 (s, Si-*C*H_3_). ^29^Si from ^1^H–^29^Si HMBC NMR (60 MHz, 298 K, CD_2_Cl_2_): δ 29.0 (s). ^19^F NMR (282 MHz, 298 K, CD_2_Cl_2_): δ −79.3 (s, OTf). ^31^P{^1^H} NMR (121 MHz, 298 K, CD_2_Cl_2_): δ 15.4 (s, *P*Cy_3_). Anal. Calcd for C_32_H_50_F_3_IrNO_4_PSSi: C, 45.05; H, 5.91; N, 1.64, found C, 44.76; H, 5.57; N, 1.70. HRMS (ESI^+^, *m*/*z*): calcd for C_32_H_50_IrNOPSi, [M-OTf]^+^ = 704.3029; found = 704.3069.

### Synthesis of Quinoline-2-oxy-dimethylsilane (**8**)

NEt_3_ (480 μL, 3.44 mmol) was added to a THF (10 mL) solution of quinolin-2-ol (400 mg, 2.76 mmol). The resulting mixture was stirred at r.t. for 2 h. After that, the solution was cooled to 0 °C, prior to the slow addition (via cannula) of a THF (3 mL) solution of HSiMe_2_Cl (286 μL, 3.03 mmol). After that, the reaction mixture was warmed to r.t. and stirred at 60 °C for 20 h. The resulting suspension was cooled to r.t. The THF solution was filtered, and the pale-brown residue was extracted with hexane (3 × 5 mL). The THF and hexane fractions were mixed and brought to dryness to give a brown oil, which was extracted with hexane (1 × 2 mL) to give **8** as a brown oil in 59% yield (330 mg, 1.62 mmol). ^1^H NMR (300 MHz, 298 K, C_6_D_6_): δ 7.90 (dd, ^3^*J*_HH_ = 9.2 Hz, ^4^*J*_HH_ = 1.1 Hz, 1H, *H*^8^), 7.43 (d, ^3^*J*_HH_ = 8.7 Hz, 1H, *H*^3^), 7.32 (overlapped m, 2H, *H*^5^ and *H*^7^), 7.08 (ddd, ^3^*J*_HH_ = 8.0 Hz, ^3^*J*_HH_ = 7.0 Hz, ^4^*J*_HH_ = 1.1 Hz, 1H, *H*^6^), 6.74 (d, ^3^*J*_HH_ = 8.7 Hz, 1H, *H*^4^), 5.44 (sept, ^3^*J*_HH_ = 2.9 Hz, 1H, Si–*H*), 0.48 (d, ^3^*J*_HH_ = 2.9 Hz, 6H, Si-(C*H*_3_)_2_). ^13^C{^1^H} NMR (75 MHz, 298 K, C_6_D_6_): δ 161.5 (s, *C*^ipso-2^), 147.3 (s, *C*^ipso-8a^), 139.6 (s, *C*^3^), 129.7 and 127.6 (both s, *C*^5^ and *C*^7^), 127.8 (s, *C*^8^), 125.5 (s, *C*^ipso-4a^), 124.3 (s, *C*^6^), 114.4 (s, *C*^4^), −1.0 (s, 2C, Si-(*C*H_3_)_2_). ^29^Si from the ^1^H–^29^Si HMQC NMR (60 MHz, 298 K, C_6_D_6_): δ 4.0 (s).

### Synthesis of 4-Methylquinoline-2-oxy-dimethylsilane (**9**)

NEt_3_ (220 μL, 1.57 mmol) was added to a THF (10 mL) solution of 4-methylquinolin-2-ol (200 mg, 1.26 mmol). The resulting mixture was stirred at r.t. for 2 h. After that, the solution was cooled to 0 °C, prior to the slow addition (via cannula) of a THF (3 mL) solution of HSiMe_2_Cl (156 μL, 1.38 mmol). After that, the reaction mixture was warmed to r.t. and stirred at 60 °C for 20 h. The resulting suspension was cooled to r.t. The THF solution was filtered and brought to dryness to give a brown oil, which was extracted with toluene (1 × 2 mL) to give **9** as a brown oil in 67% yield (183 mg, 0.84 mmol). ^1^H NMR (300 MHz, 298 K, C_6_D_6_): δ 7.97 (ddd, ^3^*J*_HH_ = 8.3 Hz, ^4^*J*_HH_ = 1.3 Hz, ^4^*J*_HH_ = 0.6 Hz, 1H, *H*^5^), 7.52 (dd, ^3^*J*_HH_ = 8.3 Hz, ^4^*J*_HH_ = 1.5 Hz, 1H, *H*^8^), 7.36 (ddd, ^3^*J*_HH_ = 8.3 Hz, ^3^*J*_HH_ = 6.9 Hz, ^4^*J*_HH_ = 1.5 Hz, 1H, *H*^6^), 7.13 (ddd, ^3^*J*_HH_ = 8.3 Hz, ^3^*J*_HH_ = 6.9 Hz, ^4^*J*_HH_ = 1.3 Hz, 1H, *H*^7^), 6.63 (q, ^4^*J*_HH_ = 1.1 Hz, 1H, *H*^3^), 5.48 (sept, ^3^*J*_HH_ = 2.9 Hz, 1H, Si–*H*), 2.06 (d, ^3^*J*_HH_ = 2.9 Hz, 3H, 4-C*H*_*3*_), 0.52 (d, ^3^*J*_HH_ = 2.9 Hz, 6H, Si(C*H*_3_)_2_). ^13^C{^1^H} NMR (75 MHz, 298 K, C_6_D_6_): δ 161.4 (s, *C*^ipso-2^), 147.7 (s, *C*^ipso-8a^), 147.4 (s, *C*^ipso-4^), 129.5 (s, *C*^6^), 128.4 (s, *C*^5^), 125.7 (s, *C*^ipso-4a^), 124.0 (s, *C*^7^), 123.8 (s, *C*^8^), 114.6 (s, *C*^3^), 18.3 (s, 4-*C*H_3_), −0.9 (s, 2C, Si-(*C*H_3_)_2_). ^29^Si from the ^1^H–^29^Si HMQC NMR (60 MHz, 298 K, C_6_D_6_): δ 3.6 (s).

### Synthesis of [Ir(H)(OTf)(κ^2^-NSi^Q^)(PCy_3_)] (**10**)

To a mixture of freshly prepared **8** (100 mg, 0.46 mmol) and [{Ir(coe)_2_}_2_(μ-Cl)_2_] (206 mg, 0.23 mmol) CH_2_Cl_2_ (10 mL) was added. The resulting solution was stirred for 30 min at r.t. to afford a yellow precipitate. The CH_2_Cl_2_ solution was filtered off and the solid residue was washed with cold hexane (2 × 3 mL) and dried in vacuo. To the resulting solid, a toluene (10 mL) solution of PCy_3_ (127 mg, 0.46 mmol) was added, and the mixture was stirred at r.t. for 2 days. After that, the solution was brought to dryness and washed with cold hexane (3 × 5 mL). To the resulting green light solid, AgTfO (112 mg, 0.46 mmol) and CH_2_Cl_2_ (10 mL) were added in the absence of light. After 15 h of stirring at r.t., the obtained suspension was filtered through Celite with cannula. The CH_2_Cl_2_ solution is brought to dryness to obtain **10** as a yellow solid in 41% yield (157 mg, 0.19 mmol). ^1^H NMR (300 MHz, 298 K, CD_2_Cl_2_): δ 8.16 (d, ^3^*J*_HH_ = 8.9 Hz, 1H, *H*^3^), 7.94 (dd, ^3^*J*_HH_ = 8.5 Hz, ^4^*J*_HH_ = 1.1 Hz, 1H, *H*^8^), 7.86 (dd, ^3^*J*_HH_ = 8.0 Hz, ^4^*J*_HH_ = 1.5 Hz, 1H, *H*^5^), 7.68 (ddd, ^3^*J*_HH_ = 8.5 Hz, ^3^*J*_HH_ = 7.0 Hz, ^4^*J*_HH_ = 1.5 Hz, 1H, *H*^7^), 7.47 (ddd, ^3^*J*_HH_ = 8.0 Hz, ^3^*J*_HH_ = 7.0 Hz, ^4^*J*_HH_ = 1.1 Hz, 1H, *H*^6^), 7.09 (d, ^3^*J*_HH_ = 8.9 Hz, 1H, *H*^4^), 2.19–1.28 (overlapping signals, 33H, C*H*- and C*H*_2_-PCy_3_), 0.87 (s, 3H, Si–C*H*_3_), 0.67 (s, 3H, Si–C*H*_3_), −28.03 (d, ^2^*J*_HP_ = 17.8 Hz, 1H, Ir–*H*). ^13^C{^1^H} NMR (75 MHz, 298 K, CD_2_Cl_2_): δ 164.5 (s, *C*^ipso-2^), 147.0 (s, *C*^ipso-8a^), 141.4 (s, *C*^3^), 130.3 (s, *C*^7^), 128.8 (s, *C*^5^), 125.7 (s, *C*^ipso-4a^), 125.5 (s, *C*^6^), 121.8 (s, *C*^8^), 114.0 (s, *C*^4^), 35.9 (d, ^2^*J*_CP_ = 30 Hz, 3C, *C*H-PCy_3_), 30.5 (br, 3C, *C*H_2_-PCy_3_), 30.3 (d, ^4^*J*_CP_ = 2.3 Hz, 3C, *C*H_2_-PCy_3_), 27.9 (d, ^4^*J*_CP_ = 2.0 Hz, 3C, *C*H_2_-PCy_3_), 27.8 (br, 3C, *C*H_2_-PCy_3_), 26.9 (br, 3C, *C*H_2_-PCy_3_), 10.6 (s, Si-*C*H_3_), 4.2 (s, Si-*C*H_3_). ^29^Si from the ^1^H–^29^Si HMQC NMR (60 MHz, 298 K, CD_2_Cl_2_): δ 26.9 (s). ^19^F NMR (282 MHz, 298 K, CD_2_Cl_2_): δ −78.69 (s, OTf). ^31^P NMR (121 MHz, 298 K, CD_2_Cl_2_): δ 18.9 (d, ^2^*J*_PH_ = 13.0 Hz, *P*Cy_3_). Anal. Calcd for C_30_H_46_F_3_IrNO_4_PSSi: C, 43.67; H, 5.62; N, 1.70, found C, 44.01; H, 5.77; N, 1.72. HRMS (ESI^+^, *m*/*z*): calcd for C_29_H_46_IrNOPSi, [M-OTf]^+^ = 676.2716; found = 676.2725.

### Synthesis of [Ir(H)(OTf)(κ^2^-NSi^MQ^)(PCy_3_)] (**11**)

To a mixture of freshly prepared **9** (100 mg, 0.46 mmol) and [{Ir(coe)_2_}_2_(μ-Cl)_2_] (206 mg, 0.23 mmol) CH_2_Cl_2_ (10 mL) was added. The resulting solution was stirred for 30 min at r.t. to afford a yellow precipitate. The CH_2_Cl_2_ solution was filtered off and the solid residue was washed with cold hexane (2 × 3 mL) and dried in vacuo. To the resulting solid, a toluene (10 mL) solution of PCy_3_ (127 mg, 0.46 mmol) was added, and the mixture was stirred at r.t. for 24 h. After that, the solution was brought to dryness and washed with cold hexane (3 × 5 mL). To the resulting green light solid, AgTfO (112 mg, 0.46 mmol) and CH_2_Cl_2_ (10 mL) were added in the absence of light. After 15 h of stirring at r.t., the obtained suspension was filtered through Celite with cannula. The CH_2_Cl_2_ solution is brought to dryness to obtain **11** as a dark green solid in 91% yield (359 mg, 0.42 mmol). ^1^H NMR (300 MHz, 298 K, CD_2_Cl_2_): δ 8.01 (dd, ^3^*J*_HH_ = 8.3 Hz, ^4^*J*_HH_ = 1.5 Hz, 1H, *H*^5^), 7.94 (m, 1H, *H*^8^), 7.69 (ddd, ^3^*J*_HH_ = 8.3 Hz, ^3^*J*_HH_ = 7.0 Hz, ^4^*J*_HH_ = 1.1 Hz, 1H, *H*^6^), 7.51 (ddd, ^3^*J*_HH_ = 8.2 Hz, ^3^*J*_HH_ = 7.0 Hz, ^4^*J*_HH_ = 1.5 Hz, 1H, *H*^7^), 6.98 (m, 1H, *H*^3^), 2.66 (d, ^4^*J*_HH_ = 1.1 Hz, 3H, 4-C*H*_3_), 2.19–1.27 (overlapping signals, 33H, C*H* and C*H*_*2*_-PCy_3_), 0.86 (s, 3H, Si–C*H*_3_), 0.67 (s, 3H, Si–C*H*_3_), −27.88 (d, ^2^*J*_HP_ = 17.7 Hz, 1H, Ir–*H*). ^13^C{^1^H} NMR (75 MHz, 298 K, CD_2_Cl_2_): δ 164.0 (s, *C*^ipso-2^), 150.6 (s, *C*^ipso-8a^), 146.8 (s, *C*^ipso-4^), 130.0 (s, *C*^6^), 126.0 (s, *C*^ipso-4a^), 125.1 (s, *C*^5^), 125.0 (s, *C*^7^), 122.2 (s, *C*^8^), 114.1 (s, *C*^3^), 35.8 (d, ^2^*J*_CP_ = 30.1 Hz, 3C, *C*H-PCy_3_), 30.5 (br, 3C, *C*H_2_-PCy_3_), 30.4 (d, ^4^*J*_CP_ = 2.3 Hz, 3C, *C*H_2_-PCy_3_), 27.9 (d, ^4^*J*_CP_ = 2.1 Hz, 3C, *C*H_2_-PCy_3_), 27.8 (br, 3C, *C*H_2_-PCy_3_), 26.9 (br, 3C, *C*H_2_-PCy_3_), 19.4 (s, 4-*C*H_3_), 10.5 (s, Si-*C*H_3_), 4.1 (s, Si-*C*H_3_). ^29^Si from the ^1^H–^29^Si HMQC NMR (60 MHz, 298 K, CD_2_Cl_2_): δ 26.9 (s). ^19^F NMR (282 MHz, 298 K, CD_2_Cl_2_): δ −78.5 (s, OTf). ^31^P{^1^H} NMR (121 MHz, 298 K, CD_2_Cl_2_): δ 18.7 (s, *P*Cy_3_). HRMS (ESI^+^, *m*/*z*): calcd. for C_30_H_48_IrNOPSi, [M-OTf]^+^ = 690.2872; found = 690.2863.

### Catalytic CDC Reaction of Pyrrolidine with HSiMe_2_Ph at the NMR Scale

Under an argon atmosphere, a NMR tube was charged with 1 mol % of the corresponding Ir precursor (**2**, 1.7 mg; **3**, 2.1 mg; **4**, 2.2 mg; **5**, 2.5 mg; **10**, 2.5 mg; **11**, 2.5 mg; 0.003 mmol), 16.7 mol % of hexamethylbenzene (8.0 mg, 0.05 mmol) as internal standard (IS) and 0.4 mL of benzene-*d*_6_. Then, pyrrolidine (24 μL, 0.3 mmol) and HSiMe_2_Ph (45 μL, 0.3 mmol) were added at room temperature (r.t.) and the resulting mixture was frozen at 0 °C. The reaction was allowed to warm to r.t. and monitored by ^1^H NMR spectroscopy (Figures S73–S78).

### **5**-Catalyzed CDC Reaction of Pyrrolidine with Different Hydrosilanes at the NMR Scale

Under an argon atmosphere, a NMR tube was charged with 1 mol % of **5** (2.5 mg, 0.003 mmol) and 16.7 mol % of hexamethylbenzene (8.0 mg, 0.05 mmol) as IS and dissolved in 0.4 mL of benzene-*d*_*6*_. Then, pyrrolidine (24 μL, 0.3 mmol) and 0.3 mmol of the corresponding hydrosilane (HSiMe_2_Ph, 45 μL; HSiMePh_2_, 58 μL; HSiEt_3_, 47 μL; and HSiMe(SiOMe_3_)_2_, 80 μL) were added at r.t. and the resulting mixture was frozen at 0 °C. The reaction was allowed to warm to r.t. and monitored by ^1^H NMR spectroscopy (Figures S79–S82).

### **5**-Catalyzed CDC Reaction of Pyrrolidine with HSiMe_2_Ph at Different Temperatures

Catalytic reactions were carried out on a microreactor (man on the moon series X102 Kit)^38^ with a total volume of 16.2 mL. Under an argon atmosphere, the reactor was filled with pyrrolidine (82 μL, 1 mmol) and **5** (8.5 mg, 0.01 mmol). The reactor was then closed and put in an external oil bath preheated at the corresponding temperature. Once the temperature and pressure of the system were stabilized, HSiMe_2_Ph (153 μL, 1 mmol) was injected with a microsyringe. Afterward, the increase in pressure in the reactor was measured until the system stopped generating hydrogen. Then, the reactor was connected to a Schlenk line, and under an argon atmosphere, hexane (2 mL) was added to the reaction crude. The solution was filtered through Celite using a cannula. The solvent was removed to dryness, and the oily product was characterized by NMR spectroscopy and high-resolution mass spectrometry (HR-MS).

### **5**-Catalyzed CDC Reaction of Aliphatic Amines with HSiMe_2_Ph

Catalytic reactions were carried out on a microreactor (man on the moon series X102 Kit)^38^ with a total volume of 16.2 mL. Under an argon atmosphere, the reactor was filled with 1 mmol of the corresponding amine (pyrrolidine, 82 μL; piperidine, 99 μL; morpholine, 86 μL; *N*-methylcyclohexylamine, 130 μL; *N*-methylbenzylamine, 129 μL; dibenzylamine, 103 μL; diisobutylamine, 175 μL) and **5** (8.5 mg, 0.01 mmol). The reactor was then closed and put in an external oil bath preheated at 323 K. Once the temperature and pressure of the system were stabilized, HSiMe_2_Ph (153 μL, 1 mmol) was injected with a microsyringe. Afterward, the increase in pressure in the reactor was measured until the system stopped generating hydrogen. Then, the reactor was connected to a Schlenk line, and under an argon atmosphere, hexane (2 mL) was added to the reaction crude. The solution was filtered through Celite using a cannula. The solvent was removed to dryness, and the oily product was characterized by NMR spectroscopy and HR-MS.

### **5**-Catalyzed CDC Reaction of Aniline Derivatives with HSiMe_2_Ph

Catalytic reactions were carried out on a microreactor (man on the moon series X102 Kit)^38^ with a total volume of 16.2 mL. Under an argon atmosphere, the reactor was filled with 0.5 mmol of the different anilines (aniline, 46 μL; *N*-methylaniline, 54 μL; *N*-ethylaniline, 63 μL; *N*-isopropylaniline, 72 μL; diphenylamine, 84.6 mg; *N*-benzylaniline, 83 μL; 3-methoxy-*N*-methylaniline, 65 μL) and **5** (4.2 mg, 0.005 mmol). The reactor was then closed and put in an external oil bath preheated at 323 K. Once the temperature and pressure of the system were stabilized, HSiMe_2_Ph (76 μL, 0.5 mmol) was injected with a microsyringe. Afterward, the increase in pressure in the reactor was measured until the system stopped generating hydrogen. Then, the reactor was connected to a Schlenk line, and under an argon atmosphere, hexane (2 mL) was added to the reaction crude. The solution was filtered through Celite using a cannula. The solvent was removed to dryness, and the oily product was characterized by NMR spectroscopy and HR-MS.

### Single-Crystal Structure Determination

Single crystals suitable for X-ray diffraction were obtained by slow diffusion of hexane (5 mL) into saturated solutions of **4**, **5** and **10** in dichloromethane (1 mL) at 273 K. Single crystal X-ray diffraction data of compounds **4**, **5** and **10** were collected on a D8 VENTURE Bruker diffractometer, using Mo κ_α_ radiation (λ = 0.71073 Å). Single crystals were mounted on a MiTeGen support and cooled to 100(2) K with open-flow nitrogen gas. Data were collected using ω and ϕ scans with narrow frames strategies. Diffracted intensities were integrated and corrected from absorption effects with SAINT^43^ and SABABS^44^ programs included in APEX4 package.^45^ Crystal structures were solved and refined using SHELXS^46^ and SHELXL^47^ included in Olex2 program.^48^ Special refinement details have been reported below. Hydrogen atoms of methyl group (C11) of complexes **4** and **5** have been included in the model in observed positions and freely refined, as they are involved in analyzed agostic interaction.

### Crystal Data of **4**

C_31_H_50_ClIrNOPSi·1/2(C_6_H_14_); *M*_r_ = 782.51; yellow prism 0.110 × 0.110 × 0.140 mm^3^; Monoclinic *C*2/*c; a* = 26.8425(8) Å, *b* = 19.7227(6) Å; *c* = 15.0096(4) Å, β = 118.6330(10)°; *V* = 6974.4(4) Å^3^; *Z* = 8; *D*_c_ = 1.490 g/cm^3^; μ = 4.012 mm^–1^; min and max. absorption correction factors: 0.6776 and 0.7468; 2θ_max_ = 68.590°; 85,956 reflections measured, 12,719 unique; *R*_int_ = 0.0325; number of data/restraint/parameters: 12,719/0/353; *R*_1_ = 0.0188 [11,417 reflections, *I* > 2σ(*I*)], w*R*(*F*^2^) = 0.0434 (all data); largest difference peak: 1.60 e Å^–3^. Hydride ligand has been included in the model in observed position and freely refined. The solvent content has been taken into account with a solvent mask.

### Crystal Data of **5**

C_32_H_46_F_3_IrNO_4_PSSi; *M*_r_ = 853.05; yellow plate 0.020 × 0.050 × 0.090 mm^3^; Monoclinic *P*2_1_/*c; a* = 9.3616(4) Å, *b* = 21.0805(10) Å; *c* = 17.4388(8) Å, β = 90.448(2)°; *V* = 3441.4(3) Å^3^; *Z* = 4; *D*_*c*_ = 1.646 g/cm^3^; μ = 4.075 mm^–1^; min and max. absorption correction factors: 0.6881 and 0.7457; 2θ_max_ = 56.616°; 205,013 reflections measured, 8566 unique; *R*_int_ = 0.0438; number of data/restraint/parameters: 8566/1/416; *R*_1_ = 0.0169 [8026 reflections, *I* > 2σ(*I*)], w*R*(*F*^2^) = 0.0410 (all data); largest difference peak: 0.898 e Å^–3^. Hydride ligand has been included in the model in observed position and refined with a restraint in Ir–H bond length.

### Crystal Data of **10**

C_30_H_30_F_3_IrNO_4_PSSi; *M*_r_ = 825.00; yellow prism 0.075 × 0.094 × 0.122 mm^3^; Monoclinic *P*2_1_/*c; a* = 9.6067(4) Å, *b* = 20.1261(8) Å; *c* = 17.4573(8) Å, β = 94.5750(10)°; *V* = 3364.5(2) Å^3^; *Z* = 4; *D*_*c*_ = 1.629 g/cm^3^; μ = 4.165 mm^–1^; min and max. absorption correction factors: 0.6636 and 0.7457; 2θ_max_ = 56.606°; 140,086 reflections measured, 8354 unique; *R*_int_ = 0.0331; number of data/restraint/parameters: 8354/2/410; *R*_1_ = 0.0132 [8144 reflections, *I* > 2σ(*I*)], w*R*(*F*^2^) = 0.0300 (all data); largest difference peak: 0.586 e Å^–3^. Hydride ligand has been included in the model in observed position and refined with a restraint in Ir–H bond length. Most of the atoms of triflate ligand have been found to be disordered.

### Computational Details

Geometry optimizations of the molecules were performed without symmetry constraints using the Gaussian03^49^ optimizer together with Turbomole 7.01^50^ energies and gradients at the BP86^51^/def2-TZVP^52^ level of theory using the D3 dispersion correction suggested by Grimme et al.^53^ and the resolution-of-identity (RI) approximation.^54^ This level is denoted RI-BP86-D3/def2-TZVP. All species were characterized by frequency calculations and have positive definite Hessian matrices confirming that they are minima on the potential energy surface. All AIM results described in this work correspond to calculations performed at the BP86-D3/6-31G(d)/WTBS (for Ir) level on the optimized geometries obtained at the RI-BP86-D3/def2-TZVP level. The WTBS (well-tempered basis sets)^55^ have been recommended for AIM calculations involving transition metals.^56^ The topology of the electron density was conducted using the AIMAll program package.^57^

## Supporting Information Available

Experimental details, NMR spectra, and theoretical calculations data (PDF)

## Abbreviations

TOF: turnover frequency
TOF_1/2_ (h^–1^): TOF (h^–1^) at 50% conversion
