# Supplementary material for: Cross-Dehydrogenative Coupling of Secondary Amines with Silanes Catalyzed by Agostic Iridium-NSi Species
Source: Inorg Chem. 2024 Dec 23;64(1):255–67. doi: 10.1021/acs.inorgchem.4c04512 (PMC11734117; doi:10.1021/acs.inorgchem.4c04512)
Supplement: Supplementary file 1 — ic4c04512_si_001.pdf [file ic4c04512_si_001.pdf]

Supporting Information

Cross-Dehydrogenative Coupling of Secondary Amines with Silanes Catalyzed by Agostic Iridium-NSi Species

Marina Padilla,[a] María Batuecas,[a] Pilar García-Orduña,[a] Israel Fernández*,[b] and Francisco J. Fernández-Álvarez*,[a]

[a] Departamento de Química Inorgánica, Facultad de Ciencias – Instituto de Síntesis Química y Catálisis Homogénea (ISQCH), Universidad de Zaragoza–CSIC, Campus Plaza San Francisco, 50009, Zaragoza, Spain.

[b] Departamento de Química Orgánica I and Centro de Innovación en Química Avanzada, Facultad de Ciencias Químicas, Universidad Complutense de Madrid, Ciudad Universitaria, 28040, Madrid, Spain.

Corresponding Author:

F.J.F.-A.: paco@unizar.es

I.F.: israel@quim.ucm.es

Table of contents

1. General information S3

2. Reaction conditions optimization... S4

2.1. Screening of Iridium catalyst precursors S4

2.2. Screening of hydrosilanes... S6

2.3. Study of reaction temperature influence.. S7

3. Amine scope.. S9

4. Reusability of **5**, **10** and **11** S12

5. Experiment at gram scale S17

6. Experiment with phenylsilane... S18

7. D-labeling studies... S20

8. Catalytic activity of **5**, **10** and **11** under neat conditions..................................... S22

9. Characterization of silylamines... S25

10. References... S31

11. NMR spectra S32

11.1. NMR spectra of proligands and Ir-complexes... S32

11.2. ^{13}C NMR spectra of **1**, **2**, **3**, **4** and **5** S59

11.3. NMR spectra of reaction conditions optimization..................................... S62

11.4. NMR spectra of silylamines S69

11.5. NMR spectra of experiment at gram scale S99

12. Cartesian coordinates... S100

1. General information

All manipulations were performed with rigorous exclusion of air at an argon/vacuo manifold using standard Schlenk-tube or glovebox techniques. Solvents were dried by the usual procedures and distilled under argon prior to use or obtained oxygen- and water-free from a Solvent Purification System (Innovative Technologies). ^{1}H, $^{13}C\{^{1}H\}$, ^{13}C APT, ^{13}C, ^{1}H-^{13}C HSQC, ^{1}H-^{13}C HMBC, $^{31}P\{^{1}H\}$, ^{31}P, ^{1}H-^{29}Si HMBC, ^{1}H-^{29}Si HMQC and ^{19}F NMR spectra were recorded on a Bruker ARX, Bruker Avance 300 MHz and Bruker Avance 400 MHz instrument. Coupling constants *J* are given in hertz (Hz) (multiplicity: s = singlet, d = doublet, dd = double doublet, ddd= doublet of doublets of doublets, psd = pseudo doublet, hept = heptuplet, m = multiplet, br = broad signal). The "Brief Guide to the Nomenclature of Organic Chemistry" was followed for signal assignment.[S1] $[\{Ir(coe)_2\}_2(\mu\text{-}Cl)_2]$[S2] was prepared following the reported methodology. The secondary amines and hydrosilanes were purchased from commercial sources and dried on 4Å molecular sieves prior to use.

Turnover Number (TON) and Turnover Frequency (TOF) determination.[S3]

H_2 pressure is the pressure measured by the microreactor. $P_{H2} = P_{measured}$

Amount of H_2 formed was calculated with the Ideal Gas Law. $n_{H2} = \frac{P_{H2} \cdot V}{R \cdot T}$

Total volume (V) = 0.0162 L; R constant = 0.082 atm L mol^{-1} K^{-1}

$$TON = \frac{n_{H2}}{n_{cat}}; \; TOF = \frac{TON}{t}$$

2. Reaction conditions optimization

2.1. Screening of Iridium catalyst precursors

*Comparison between **B1**, **5**, **10** and **11** as catalysts for the CDC reaction at NMR scale*

Catalytic reactions were carried out on a microreactor (man on the moon ™ series X102 Kit)[S4] with a total volume of 16.2 mL. Under an argon atmosphere, the reactor was filled with *N*-methylaniline (54 μL, 0.5 mmol) and the corresponding catalyst precursor (**B1**, 4.3 mg; **5**, 4.2 mg; **10**, 4.1 mg; **11**, 4.2 mg; 0.005 mmol) (Scheme S1). The reactor was then closed and put in an external oil bath preheated at 323 K. Once the temperature and pressure of the system were stabilized, $HSiMe_2Ph$ (76 μL, 0.5 mmol) was injected with a microsyringe. The results are shown in Figure S1 and Table S1.

Scheme S1. **B1**, **5**, **10** and **11**-catalyzed (1 mol %) reaction of *N*-methylaniline with $HSiMe_2Ph$ at 323 K under neat conditions.

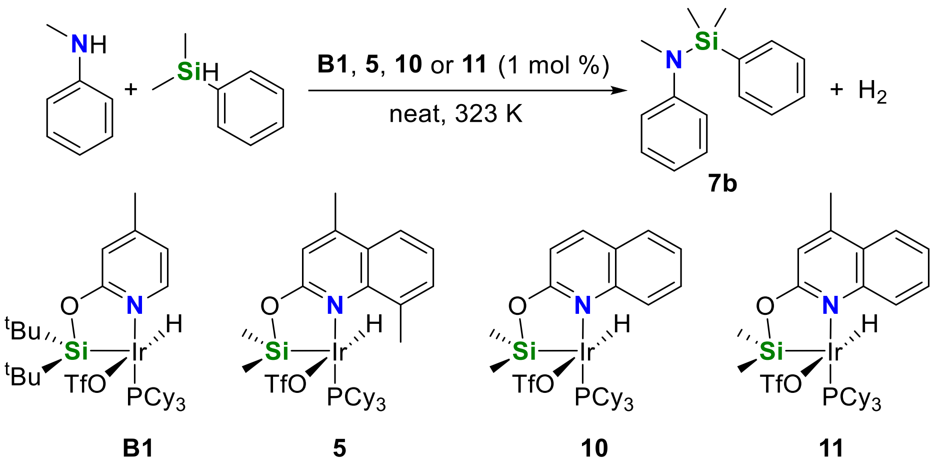

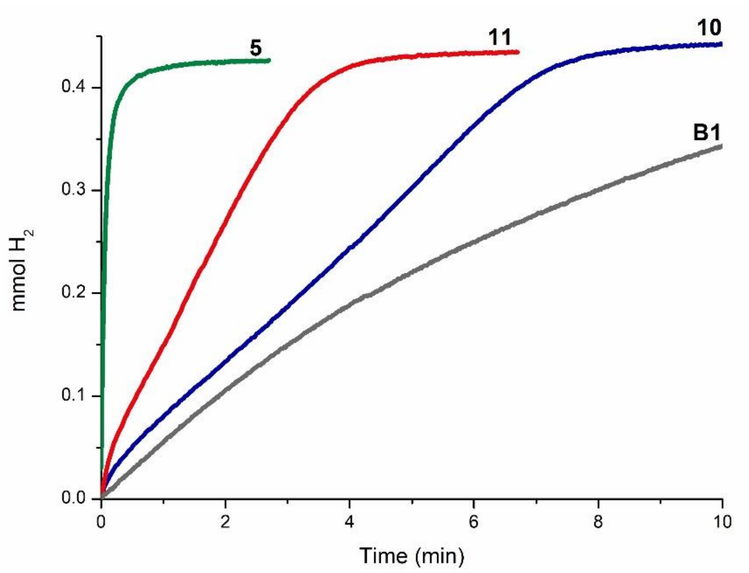

Figure S1. Time profile of H_2 (mmol) generation from the **B1**, **5**, **10** and **11**-catalyzed (1 mol %) reaction of *N*-methylaniline (0.5 mmol) with $HSiMe_2Ph$ (0.5 mmol) at 323 K under neat conditions.

Table S1. TOF of the **B1**, **5**, **10** and **11**-catalyzed (1 mol %) reaction of *N*-methylaniline (0.5 mmol) with $HSiMe_2Ph$ (0.5 mmol) at 323 K under neat conditions.

Cat.	$TOF_{0.2min}$ (h^{-1})	$TOF_{1/2}$ (h^{-1})
**B1**	690	530
**5**	20350	46000
**10**	1700	740
**11**	3200	1690

*Comparison between **2**, **3**, **4**, **5**, **10** and **11** as catalysts for the CDC reaction at NMR scale*

Under an argon atmosphere, a NMR tube was charged with 1 mol % of the corresponding Ir complex (**2**, 1.7 mg; **3**, 2.1 mg; **4**, 2.2 mg; **5**, 2.5 mg; **10**, 2.5 mg; **11**, 2.5 mg; 0.003 mmol) and 16.7 mol % of hexamethylbenzene (8.0 mg, 0.05 mmol) as internal standard (IS) and dissolved in 0.4 mL of benzene-d_6. Then, pyrrolidine (24 µL, 0.3 mmol) and $HSiMe_2Ph$ (45 µL, 0.3 mmol) were added at room temperature (r.t.) and the resulting mixture was frozen at 0 ºC (Scheme S2). The reaction was allowed to warm to r.t. and monitored by ^{1}H NMR spectroscopy (Figures S73-S78). The results are shown in Table S2.

Scheme S2. Catalytic reaction of pyrrolidine with $HSiMe_2Ph$ using different Ir catalyst precursors (1 mol %) in C_6D_6 at r.t.

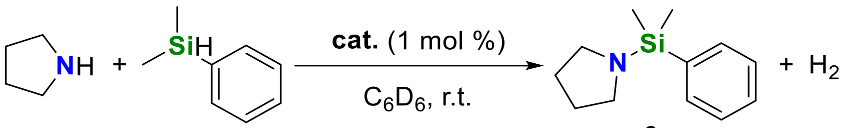

Table S2. Results of the catalytic reaction of pyrrolidine with $HSiMe_2Ph$ using different Ir catalyst precursors (1 mol %) in C_6D_6 at r.t. using hexamethylbenzene as IS.

Cat.	Time (h)	**6a** (mol %)[a]
**2**	0.5	8
	3	23
**3**	0.5	16
	3	48
**4**	0.5	25
	3	38
**5**	0.5	75
	3	92
**10**	0.5	26
	3	47
**11**	0.5	28
	3	54

[a]Obtained by ^{1}H NMR integration

2.2. Screening of hydrosilanes

Under an argon atmosphere, a NMR tube was charged with 1 mol % of **5** (2.5 mg, 0.003 mmol) and 16.7 mol % of hexamethylbenzene (8.0 mg, 0.05 mmol) as IS and dissolved in 0.4 mL of benzene-*d*6. Then, pyrrolidine (24 µL, 0.3 mmol) and 0.3 mmol of the corresponding hydrosilane ($HSiMe_2Ph$, 45 µL; $HSiMePh_2$, 58 µL; $HSiEt_3$, 47 µL; and $HSiMe(SiOMe_3)_2$, 80 µL) were added at r.t. and the resulting mixture was frozen at 0 ºC (Scheme S3). The reaction was allowed to warm to r.t. and monitored by ^{1}H NMR spectroscopy (Figures S79-S82). The results are shown in Table S3.

Scheme S3. **5**-catalyzed (1 mol %) reaction of pyrrolidine with different hydrosilanes in C_6D_6 at r.t.

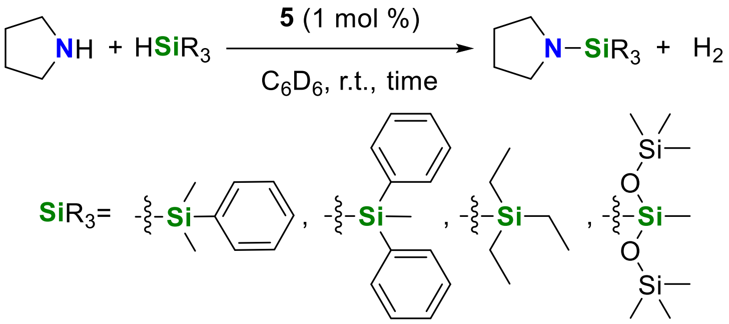

Table S3. Results of the **5**-catalyzed (1 mol %) reaction of pyrrolidine with different hydrosilanes in C_6D_6 at r.t. using hexamethylbenzene as IS.

Hydrosilane	Time (h)	% Silylamine[a]
**$HSiEt_3$**	0.5	<1
	3	<1
**$HSiMe(SiOMe_3)_2$**	0.5	<1
	3	10
**$HSiMePh_2$**	0.5	19
	3	38
**$HSiMe_2Ph$**	0.5	75
	3	92

[a]Obtained by ^{1}H NMR integration

2.3. Study of reaction temperature influence

Catalytic reactions were carried out on a microreactor (man on the moon ™ series X102 Kit)[S4] with a total volume of 16.2 mL. Under an argon atmosphere, the reactor was filled with pyrrolidine (82 µL, 1 mmol) and **5** (8.5 mg, 0.01 mmol). The reactor was then closed and put in an external oil bath preheated at the corresponding temperature. Once the temperature and pressure of the system were stabilized, $HSiMe_2Ph$ (153 µL, 1 mmol) was injected with a microsyringe (Scheme S4). The results are shown in Figure S2 and Table S4.

Scheme S4. 5-catalyzed (1 mol %) reaction of pyrrolidine with $HSiMe_2Ph$ at different temperatures under neat conditions.

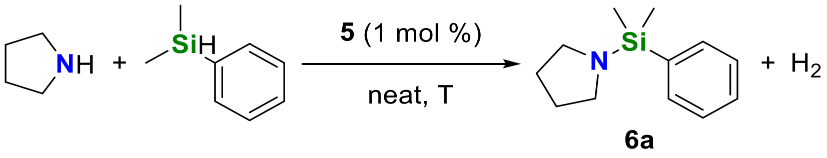

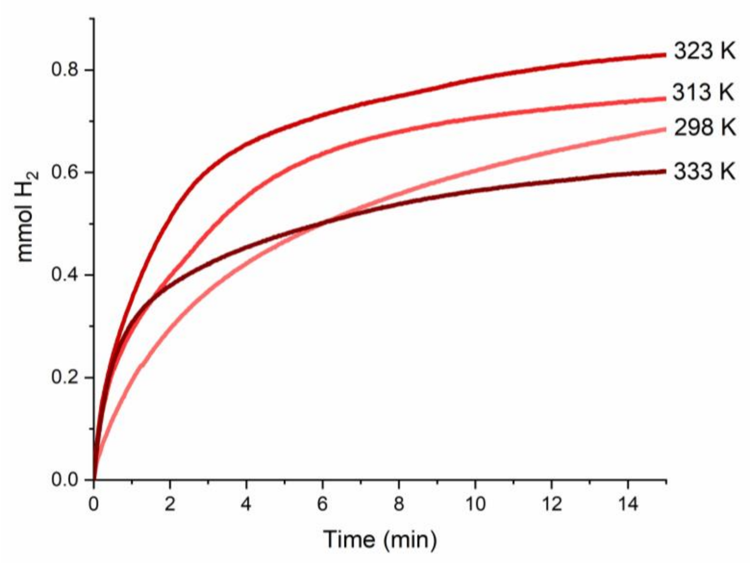

Figure S2. Time profile of H_2 (mmol) generation from the **5**-catalyzed (1 mol %) reaction of pyrrolidine (1 mmol) with $HSiMe_2Ph$ (1 mmol) at different temperatures under neat conditions.

Table S4. TOF of the **5**-catalyzed (1 mol %) reaction of pyrrolidine with $HSiMe_2Ph$ at different temperatures under neat conditions.

Temperature (K)	TOF_{2min} (h^{-1})	$TOF_{1/2}$ (h^{-1})
**298**	890	610
**313**	1190	1160
**323**	1540	1790
**333**	1140	1800

3. Amine scope

Catalytic reactions were carried out on a microreactor (man on the moon ™ series X102 Kit)[S4] with a total volume of 16.2 mL. Under an argon atmosphere, the reactor was filled with 1 mmol of the corresponding amine (*N*-methylaniline, 108 µL; pyrrolidine, 82 µL; piperidine, 99 µL; morpholine, 86 µL; *N*-methylcyclohexylamine, 130 µL; *N*-methylbenzylamine, 129 µL; dibenzylamine, 193 µL; diisobutylamine, 175 µL) and **5** (8.5 mg, 0.01 mmol). The reactor was then closed and put in an external oil bath preheated at 323 K. Once the temperature and pressure of the system were stabilized, $HSiMe_2Ph$ (153 µL, 1 mmol) was injected with a microsyringe (Scheme S5). The results are shown in Figure S3 and Table S5.

Scheme S5. 5-catalyzed (1 mol %) reaction of different amines with $HSiMe_2Ph$ at 323K under neat conditions.

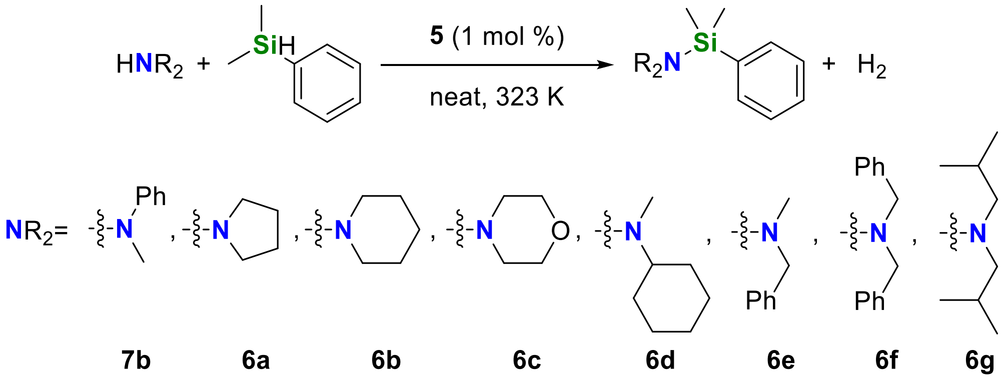

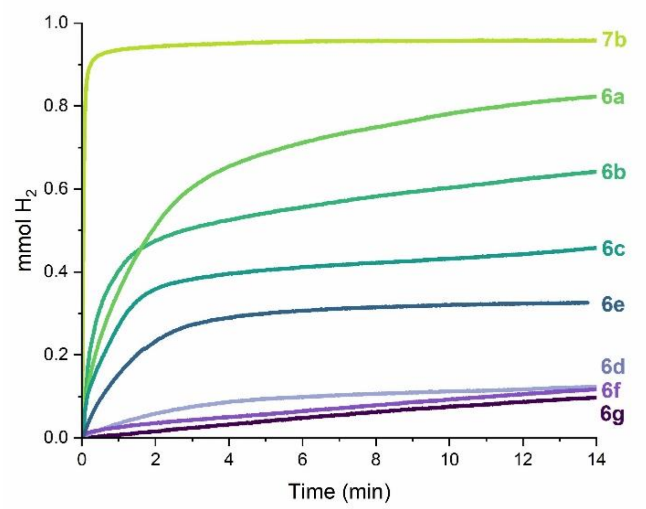

Figure S3. Time profile of H_2 (mmol) generation from the **5**-catalyzed (1 mol %) reaction of different amines (1 mmol) with $HSiMe_2Ph$ (1 mmol) at 323 K under neat conditions.

Table S5. TOF of the **5**-catalyzed (1 mol %) reaction of different amines with $HSiMe_2Ph$ at 323 K under neat conditions.

Amine	TOF_{2min} (h^{-1})	$TOF_{1/2}$ (h^{-1})
***N*-methylaniline (7b)**	2830	84800
**Pyrrolidine (6a)**	1540	1800
**Piperidine (6b)**	1430	3200
**Morpholine (6c)**	1100	1600
***N*-methylcyclohexylamine (6d)**	180	130
***N*-methylbenzylamine (6e)**	700	900
**Dibenzylamine (6f)**	110	40
**Diisobutylamine (6g)**	50	40

Catalytic reactions were carried out on a microreactor (man on the moon ™ series X102 Kit)[S4] with a total volume of 16.2 mL. Under an argon atmosphere, the reactor was filled with 0.5 mmol of the different anilines (aniline, 46 µL; *N*-methylaniline, 54 µL; *N*-ethylaniline, 63 µL; *N*-isopropylaniline, 72 µL; diphenylamine, 84.6 mg; *N*-benzylaniline, 83 µL; 3-methoxy-*N*-methylaniline, 65 µL) and **5** (4.2 mg, 0.005 mmol). The reactor was then closed and put in an external oil bath preheated at 323 K. Once the temperature and pressure of the system were stabilized, $HSiMe_2Ph$ (76 µL, 0.5 mmol) was injected with a microsyringe (Scheme S6). The results are shown in Figure S4 and Table S6.

Scheme S6. **5**-catalyzed (1 mol %) reaction of different anilines derivatives with $HSiMe_2Ph$ at 323 K under neat conditions.

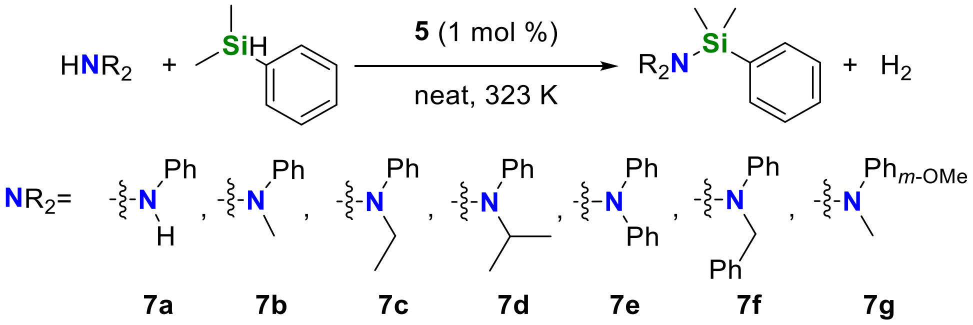

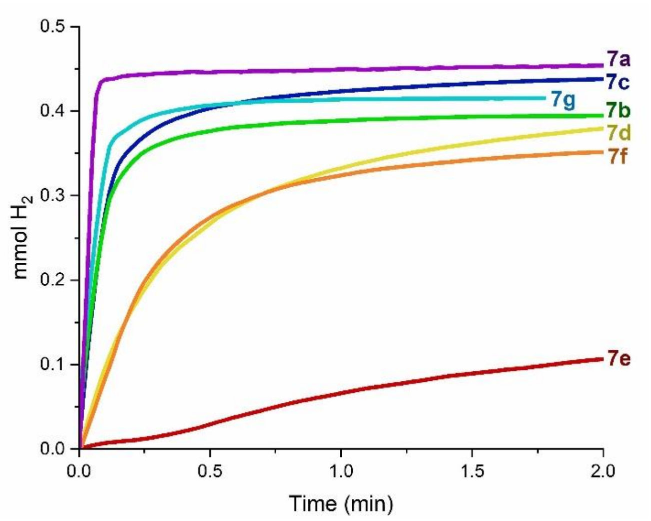

Figure S4. Time profile of H_2 (mmol) generation from the **5**-catalyzed (1 mol %) reaction of different anilines derivatives (0.5 mmol) with $HSiMe_2Ph$ (0.5 mmol) at 323 K under neat conditions.

Table S6. TOF of the **5**-catalyzed (1 mol %) reaction of different anilines derivatives with $HSiMe_2Ph$ at 323 K under neat conditions.

Aniline derivatives	$TOF_{0.2min}$ (h^{-1})	$TOF_{1/2}$ (h^{-1})
**Aniline (7a)**	26500	70900
***N*-methylaniline (7b)**	20350	46000
***N*-ethylaniline (7c)**	21300	39700
***N*-isopropylaniline (7d)**	9800	8400
**Diphenylamine (7e)**	700	580
***N*-benzylaniline (7f)**	10100	9900
**3-methoxy-*N*-methylaniline (7g)**	22900	66300

4. Reusability of 5, 10 and 11

Reusability experiment

Catalytic reactions were carried out on a microreactor (man on the moon ™ series X102 Kit)[S4] with a total volume of 16.2 mL. Under an argon atmosphere, the reactor was filled with *N*-methylaniline (54 μL, 0.5 mmol) and the corresponding catalyst precursor (**5**, 4.2 mg; **10**, 4.1 mg; **11**, 4.2 mg; 0.005 mmol) (Scheme S7). The reactor was then closed and put in an external oil bath preheated at 323 K. Once the temperature and pressure of the system were stabilized, $HSiMe_2Ph$ (76 μL, 0.5 mmol) was injected with a microsyringe (Scheme S7). When the reaction was finished, another charge of reagents was injected to the microreactor. The recharge process was repeated four times. The results are shown in Figures S5-S7 and Tables S7-S9.

Scheme S7. **5**, **10** and **11**-catalyzed (1 mol %) reaction of *N*-methylaniline with $HSiMe_2Ph$ at 323 K under neat conditions.

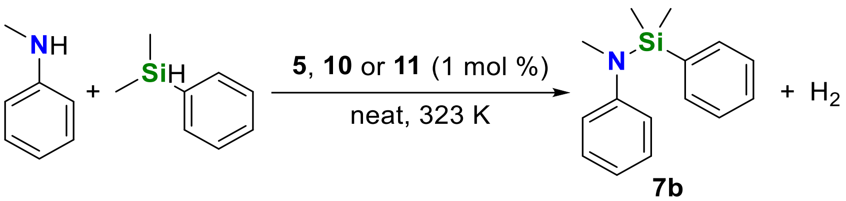

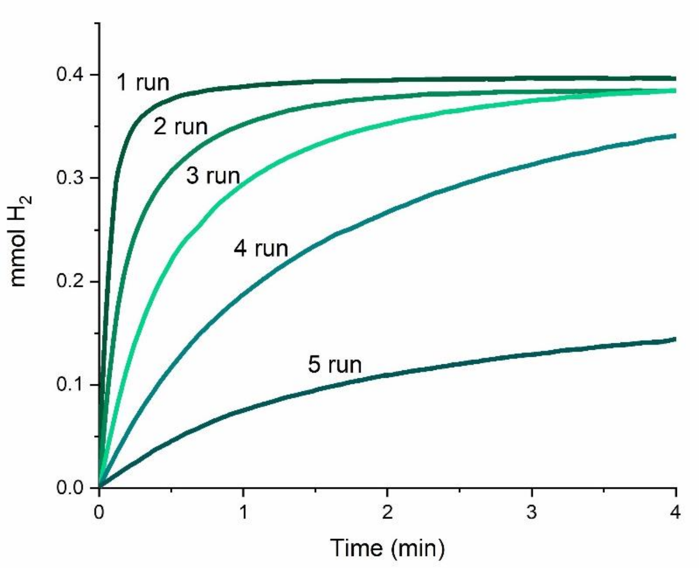

Figure S5. Time profile of H_2 (mmol) generation from the **5**-catalyzed (1 mol %) reaction of *N*-methylaniline (0.5 mmol) with $HSiMe_2Ph$ (0.5 mmol) at 323 K under neat conditions.

Table S7. TOF of the **5**-catalyzed (1 mol %) reaction of *N*-methylaniline with $HSiMe_2Ph$ at 323 K under neat conditions.

Run	$TOF_{0.2min}$ (h^{-1})	$TOF_{1/2}$ (h^{-1})
**1**	20350	41300
**2**	13350	15300
**3**	7200	5900
**4**	3250	2150
**5**	750	50

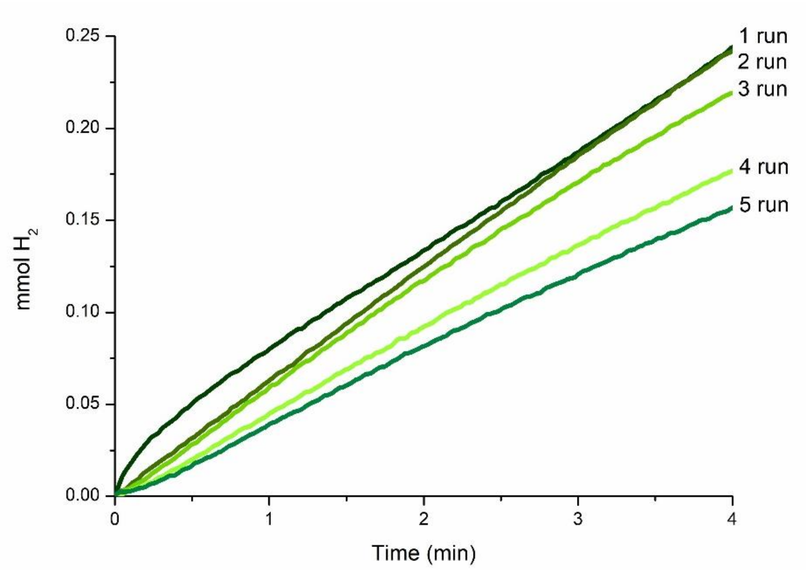

Figure S6. Time profile of H_2 (mmol) generation from the **10**-catalyzed (1 mol %) reaction of *N*-methylaniline (0.5 mmol) with $HSiMe_2Ph$ (0.5 mmol) at 323 K under neat conditions.

Table S8. TOF of the **10**-catalyzed (1 mol %) reaction of *N*-methylaniline with $HSiMe_2Ph$ at 323 K under neat conditions.

Run	$TOF_{0.2min}$ (h^{-1})	$TOF_{1/2}$ (h^{-1})
**1**	1700	740
**2**	800	730
**3**	580	660
**4**	400	510
**5**	330	440

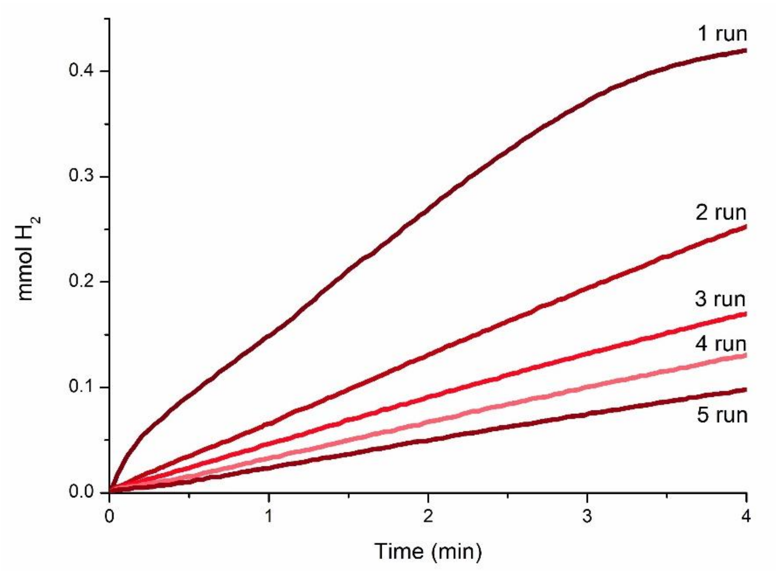

Figure S7. Time profile of H_2 (mmol) generation from the **11**-catalyzed (1 mol %) reaction of *N*-methylaniline (0.5 mmol) with $HSiMe_2Ph$ (0.5 mmol) at 323 K under neat conditions.

Table S9. TOF of the **11**-catalyzed (1 mol %) reaction of *N*-methylaniline with $HSiMe_2Ph$ at 323 K under neat conditions.

Run	$TOF_{0.2min}$ (h^{-1})	$TOF_{1/2}$ (h^{-1})
**1**	3200	1690
**2**	950	770
**3**	620	490
**4**	430	350
**5**	290	250

Reusability experiment using 0.25 mol % of ***5***

Catalytic reactions were carried out on a microreactor (man on the moon ™ series X102 Kit)[S4] with a total volume of 16.2 mL. Under an argon atmosphere, the reactor was filled with *N*-methylaniline (54 µL, 0.5 mmol) and **5** (1.1 mg, 0.00125 mmol). The reactor was then closed and put in an external oil bath preheated at 323 K. Once the temperature and pressure of the system were stabilized, $HSiMe_2Ph$ (76 µL, 0.5 mmol) was injected with a microsyringe (Scheme S8). When the reaction was finished, another charge of reagents was injected to the microreactor. The recharge process was repeated four times. The results are shown in Figure S8 and Table S10.

Scheme S8. **5**-catalyzed (0.25 mol %) reaction of *N*-methylaniline with $HSiMe_2Ph$ at 323 K under neat conditions.

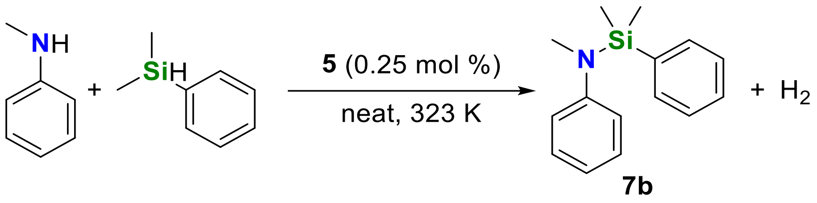

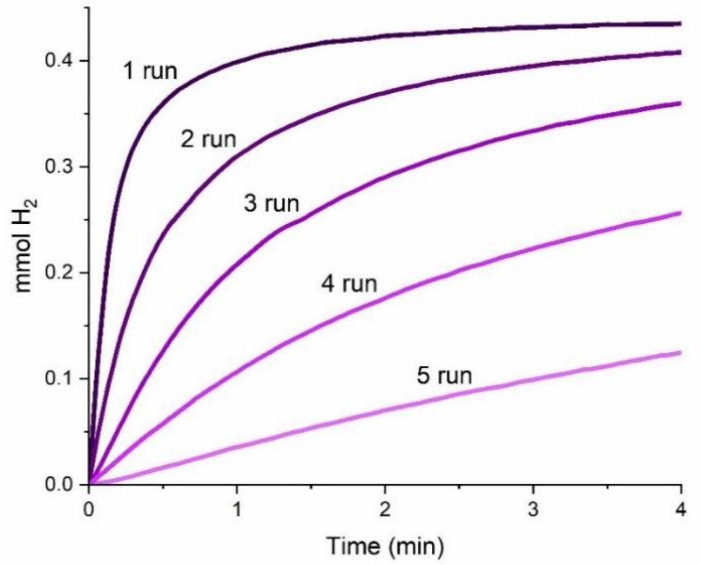

Figure S8. Time profile of H_2 (mmol) generation from the **5**-catalyzed (0.25 mol %) reaction of *N*-methylaniline (0.5 mmol) with $HSiMe_2Ph$ (0.5 mmol) at 323 K under neat conditions.

Table S10. TOF of the **5**-catalyzed (0.25 mol %) reaction of *N*-methylaniline with $HSiMe_2Ph$ at 323 K under neat conditions.

Run	$TOF_{0.2min}$ (h^{-1})	$TOF_{1/2}$ (h^{-1})
**1**	65900	79300
**2**	29500	23700
**3**	12300	9600
**4**	5800	3600
**5**	1200	1100

Dilution experiment

Catalytic reactions were carried out on a microreactor (man on the moon ™ series X102 Kit)[S4] with a total volume of 16.2 mL. Under an argon atmosphere, the reactor was filled with *N*-methylaniline (54 µL, 0.5 mmol), **5** (4.2 mg, 0.005 mmol) and *N*,1,1-trimethyl-*N*,1-diphenylsilanamine (**7b**, 485 mg, 2 mmol) as solvent. The reactor was then closed and put in an external oil bath preheated at 323 K. Once the temperature and pressure of the system were stabilized, $HSiMe_2Ph$ (76 µL, 0.5 mmol) was injected with a microsyringe (Scheme S9). The results of this experiment were compared with those obtained from a reusability experiment (described in the previous section) using 0.005 mmol of **5** (Figure S9).

Scheme S9. **5**-catalyzed (1 mol %) reaction of *N*-methylaniline with $HSiMe_2Ph$ in the presence of **7b** as solvent at 323 K.

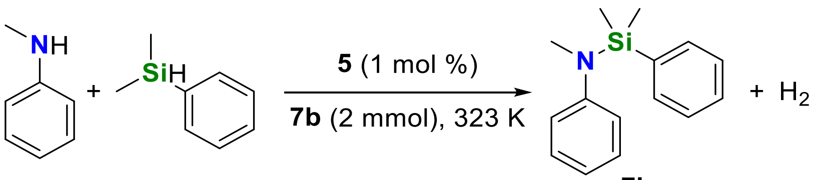

7b

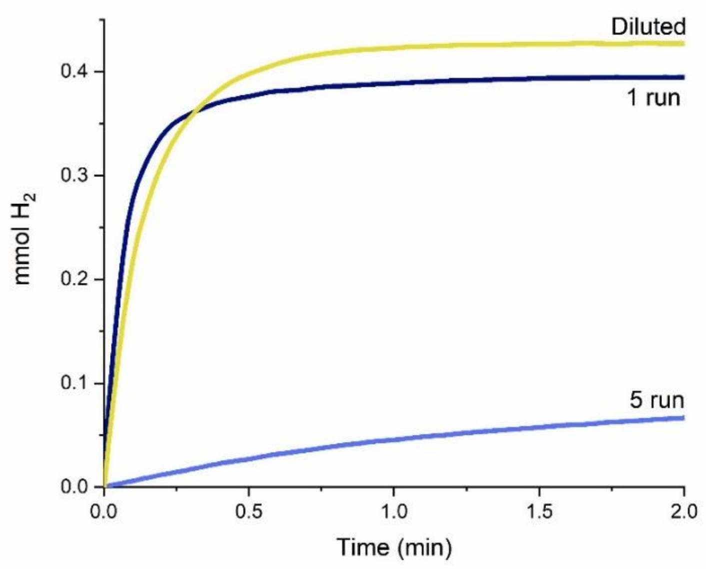

Figure S9. Comparison of time profile of H_2 (mmol) generation from the **5**-catalyzed (0.005 mmol) reaction of *N*-methylaniline (0.5 mmol) with $HSiMe_2Ph$ (0.5 mmol) in 1 run, 5 run and diluted reaction at 323 K.

5. Experiment at gram scale

Under an argon atmosphere, a Schlenk tube was filled with **5** (8.5 mg, 0.01 mmol) and *N*-methylaniline (0.43 mL, 4 mmol) and $HSiMe_2Ph$ (0.61 mL, 4 mmol) were added with a syringe. The Schlenk tube was equipped with a bubbler and the reaction mixture was stirred at r.t. for 10 min. After that, when the H_2 bubbling is finished, hexane (2 mL) was added to the reaction crude and the solution was filtered through celite with a cannula. The resulting hexane solution was brought to dryness to give **7b** as an orange oil in 93% yield (0.90 g, 3.7 mmol). Figure S143 shows the ^1H NMR spectrum of the obtained product.

6. Experiment with phenylsilane

Catalytic reaction was carried out on a microreactor (man on the moon ™ series X102 Kit)[S4] with a total volume of 16.2 mL. Under an argon atmosphere, the reactor was filled with *N*-methylaniline (54 µL, 0.5 mmol) and **5** (4.2 mg, 0.005 mmol) (Scheme S10). The reactor was then closed and put in an external oil bath preheated at 323 K. Once the temperature and pressure of the system were stabilized, H_3SiPh (62 µL, 0.5 mmol) was injected with a microsyringe. The results are shown in Figure S10 and Figures S139-S142.

Scheme S10. **5**-catalyzed (1 mol %) reaction of *N*-methylaniline with H_3SiPh at 323 K under neat conditions.

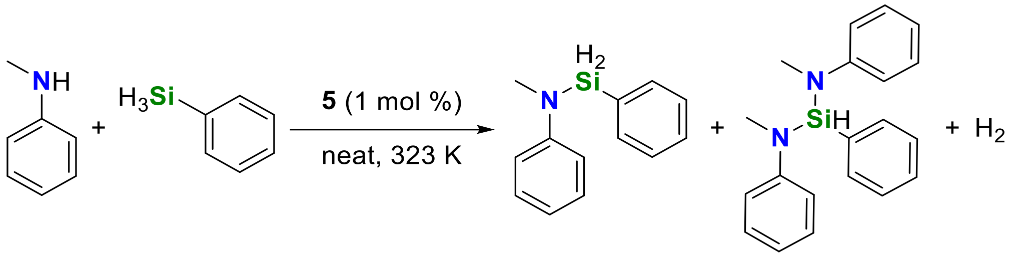

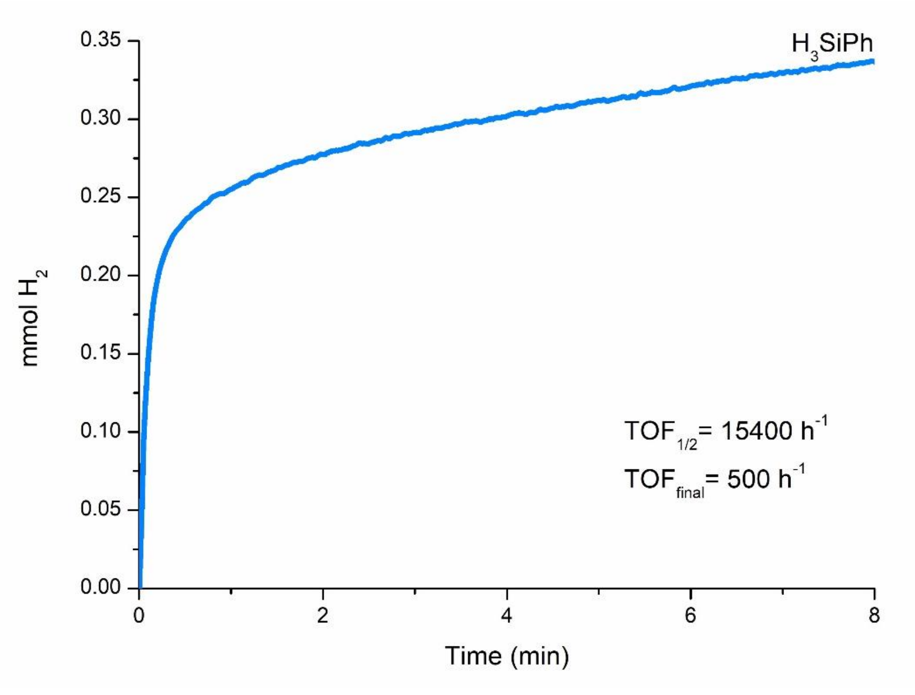

Figure S10. Time profile of H_2 (mmol) generation from the **5**-catalyzed (1 mol %) reaction of *N*-methylaniline (0.5 mmol) with H_3SiPh (0.5 mmol) at 323 K under neat conditions.

*NMR data obtained for **7h-1** and **7h-2***

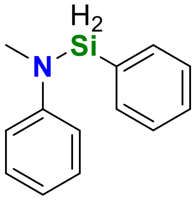

***N*-methyl-*N*,1-diphenylsilanamine (7h-1)**. ^{1}H NMR (300 MHz, 298 K, C_6D_6): δ 7.48 (m, 2H, C*H*-Ar), 7.11 (m, 5H, C*H*-Ar), 6.93 (m, 2H, C*H*-Ar), 6.78 (m, 1H, C*H*-Ar), 5.25 (s, 2H, Si-*H*), 2.72 (s, 3H, N-C*H*$_3$). ^{13}C{^{1}H} NMR (75 MHz, 298 K, C_6D_6): δ 149.8 (s, C^{ipso}), 134.7 (s, 2C, *C*H-Ar), 134.5 (s, C^{ipso}), 132.8 (s, *C*H-Ar), 129.4 (s, 2C, *C*H-Ar), 128.6 (s, 2C, *C*H-Ar), 119.2 (s, *C*H-Ar), 116.0 (s, 2C, *C*H-Ar), 36.5 (s, N-*C*H$_3$). ^{29}Si from the ^{1}H-^{29}Si HMQC NMR (60 MHz, 298 K, C_6D_6): δ –25.7 (s).

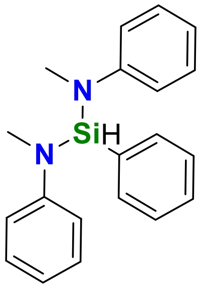

***N,N*'-dimethyl-*N,N'*,1-triphenylsilanediamine (7h-2)**. ^{1}H NMR (300 MHz, 298 K, C_6D_6): δ 7.48 - 6.74 (overlapping signals, 15H, C*H*-Ar), 5.80 (s, 1H, Si-*H*), 2.69 (s, 3H, N-C*H*$_3$). ^{13}C{^{1}H} NMR (75 MHz, 298 K, C_6D_6): δ 150.1 (s, C^{ipso}), 136.1, 135.2, 130.7, 129.4, 128.8 (s, *C*H-Ar), 133.5 (s, 2C, C^{ipso}), 119.8 (s, *C*H-Ar), 116.7 (s, *C*H-Ar), 33.9 (s, N-*C*H$_3$). ^{29}Si from the ^{1}H-^{29}Si HMQC NMR (60 MHz, 298 K, C_6D_6): δ –23.3 (s).

7. D-labeling studies

Comparison between N-methylaniline and N-methylaniline-d_1 under neat conditions

Catalytic reactions were carried out on a microreactor (man on the moon ™ series X102 Kit)[S4] with a total volume of 16.2 mL. Under an argon atmosphere, the reactor was filled with *N*-methylaniline-d_1[S5] (54 µL, 0.5 mmol) or *N*-methylaniline (54 µL, 0.5 mmol), and **5** (4.2 mg, 0.005 mmol). The reactor was then closed and put in an external oil bath preheated at 323 K. Once the temperature and pressure of the system were stabilized, $HSiMe_2Ph$ (76 µL, 0.5 mmol) was injected with a microsyringe (Scheme S11). The results are shown in Figure S11.

Scheme S11. **5**-catalyzed (1 mol %) reaction of *N*-methylaniline-d_1 with $HSiMe_2Ph$ at 323 K under neat conditions.

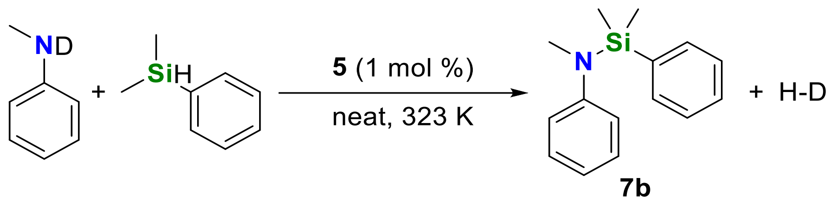

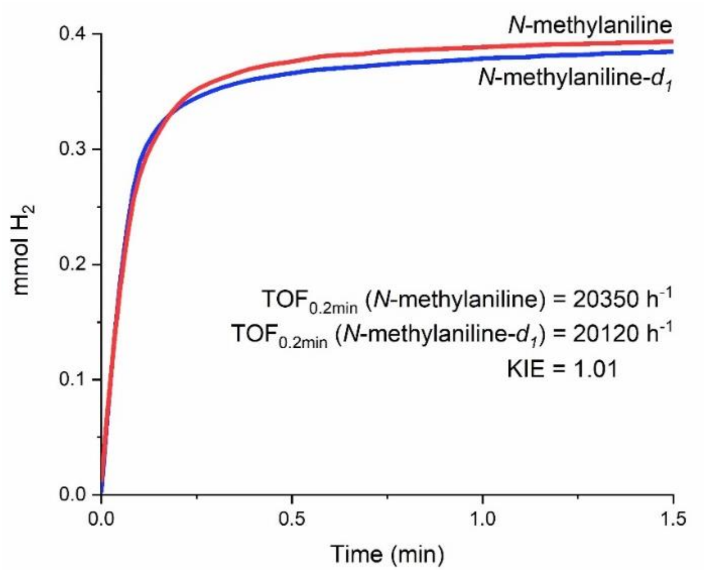

Figure S11. Comparison of time profile of H_2 (mmol) generation from the **5**-catalyzed (1 mol %) reaction of *N*-methylaniline and *N*-methylaniline-d_1 (0.5 mmol) with $HSiMe_2Ph$ (0.5 mmol) at 323 K under neat conditions.

Comparison between $HSiMe_2Ph$ and $DSiMe_2Ph$ under neat conditions

Catalytic reactions were carried out on a microreactor (man on the moon ™ series X102 Kit)[S4] with a total volume of 16.2 mL. Under an argon atmosphere, the reactor was filled with *N*-methylaniline (54 µL, 0.5 mmol) and **5** (4.2 mg, 0.005 mmol). The reactor was then closed and put in an external oil bath preheated at 323 K. Once the temperature and pressure of the system were stabilized, $DSiMe_2Ph$[S6] (76 µL, 0.5 mmol) or $HSiMe_2Ph$ (76 µL, 0.5 mmol) was injected with a microsyringe (Scheme S12). The results are shown in Figure S12.

Scheme S12. **5**-catalyzed (1 mol %) reaction of *N*-methylaniline with $DSiMe_2Ph$ at 323 K under neat conditions.

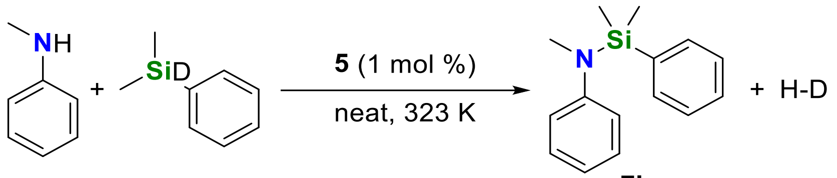

7b

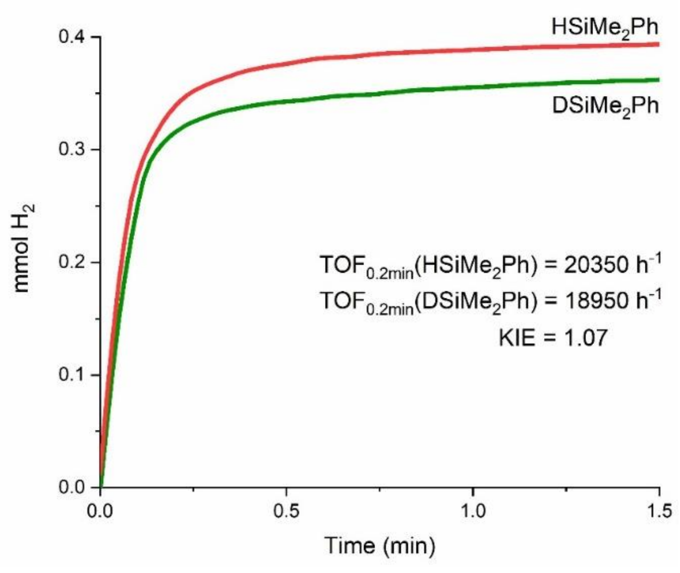

Figure S12. Comparison of time profile of H_2 (mmol) generation from the **5**-catalyzed (1 mol %) reaction of *N*-methylaniline (0.5 mmol) with $HSiMe_2Ph$ and $DSiMe_2Ph$ (0.5 mmol) at 323 K under neat conditions.

8. Catalytic activity of 5, 10 and 11 under neat conditions

Reaction of N-methylaniline with $HSiMe_2Ph$

Catalytic reactions were carried out on a microreactor (man on the moon ™ series X102 Kit)[S4] with a total volume of 16.2 mL. Under an argon atmosphere, the reactor was filled with *N*-methylaniline (54 µL, 0.5 mmol) and the corresponding catalyst precursor (**5**, 4.2 mg; **10**, 4.1 mg; **11**, 4.2 mg; 0.005 mmol). The reactor was then closed and put in an external oil bath preheated at 323 K. Once the temperature and pressure of the system were stabilized, $HSiMe_2Ph$ (76 µL, 0.5 mmol) was injected with a microsyringe (Scheme S13). The results are shown in Figure S13 and Table S11.

Scheme S13. 5, **10** and **11**-catalyzed (1 mol %) reaction of *N*-methylaniline with $HSiMe_2Ph$ at 323 K under neat conditions.

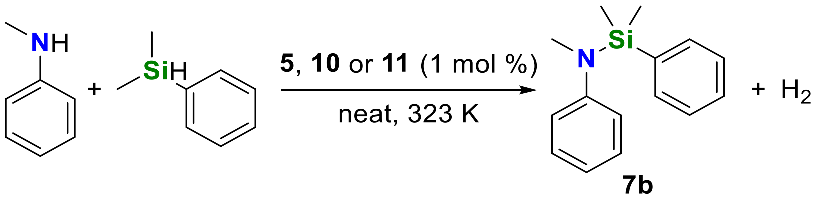

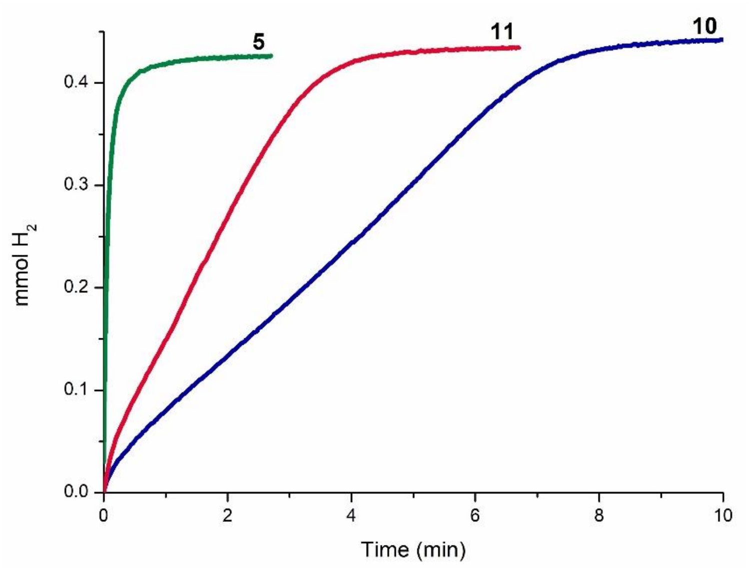

Figure S13. Time profile of H_2 (mmol) generation from the **5**, **10** and **11**-catalyzed (1 mol %) reaction of *N*-methylaniline (0.5 mmol) with $HSiMe_2Ph$ (0.5 mmol) at 323 K under neat conditions.

Table S11. TOF of the **5**, **10** and **11**-catalyzed (1 mol %) reaction of *N*-methylaniline (0.5 mmol) with $HSiMe_2Ph$ (0.5 mmol) at 323 K under neat conditions.

Cat.	$TOF_{0.2min}$ (h^{-1})	$TOF_{1/2}$ (h^{-1})	TOF_{final} (h^{-1})
**5**	20350	46000	3900
**10**	1700	740	520
**11**	3200	1690	960

Reaction of pyrrolidine with $HSiMe_2Ph$

Catalytic reactions were carried out on a microreactor (man on the moon ™ series X102 Kit)[S4] with a total volume of 16.2 mL. Under an argon atmosphere, the reactor was filled with pyrrolidine (82 µL, 1 mmol) and the corresponding catalyst precursor (**5**, 8.5 mg; **10**, 8.3 mg; **11**, 8.4 mg; 0.01 mmol). The reactor was then closed and put in an external water bath at 298 K. Once the temperature and pressure of the system were stabilized, $HSiMe_2Ph$ (153 µL, 1 mmol) was injected with a microsyringe (Scheme S14). The results are shown in Figure S14 and Table S12.

Scheme S14. 5, **10** and **11**-catalyzed (1 mol %) reaction of pyrrolidine with $HSiMe_2Ph$ at r.t. under neat conditions.

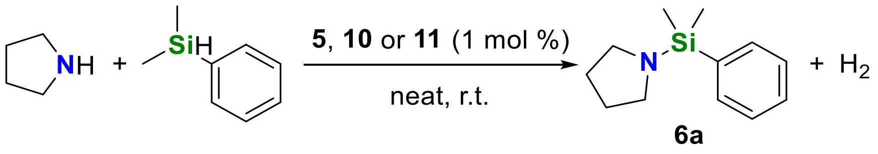

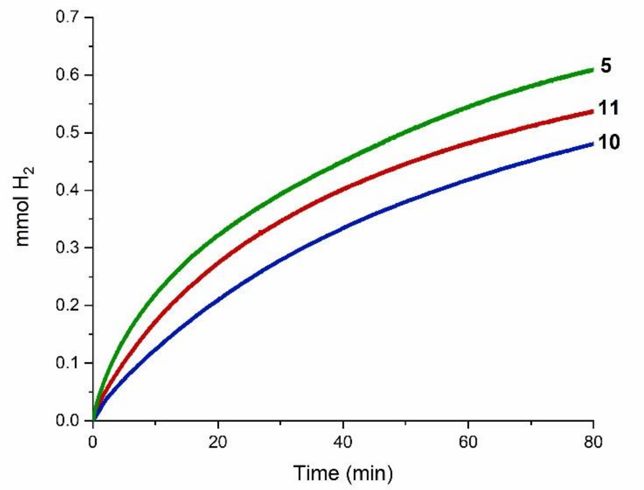

Figure S14. Time profile of H_2 (mmol) generation from the **5**, **10** and **11**-catalyzed (1 mol %) reaction of pyrrolidine (1 mmol) with $HSiMe_2Ph$ (1 mmol) at r.t. under neat conditions.

Table S12. TOF of the **5**, **10** and **11**-catalyzed (1 mol %) reaction of pyrrolidine (1 mmol) with $HSiMe_2Ph$ (1 mmol) at r.t under neat conditions.

Cat.	TOF_{2min} (h^{-1})	$TOF_{1/2}$ (h^{-1})
**5**	220	100
**10**	110	50
**11**	150	80

9. Characterization of silylamines

General procedure for the CDC reaction under neat conditions

Catalytic reactions were carried out on a microreactor (man on the moon ™ series X102 Kit)[S4] with a total volume of 16.2 mL. Under an argon atmosphere, the reactor was filled with the corresponding amount of each amine (0.5 mmol) and **5** (4.2 mg, 0.005 mmol). The reactor was then closed and put in an external oil bath preheated at 323 K, and when the temperature and pressure of the system were stabilized, the corresponding amount of $HSiMe_2Ph$ (0.5 mmol) was injected with a microsyringe (Scheme S15). Once the reaction has finished, the reactor was opened and connected to a Schlenk line. Then, under argon atmosphere, hexane (2 mL) was added and the solution was filtered through celite with a cannula to remove the residual catalyst. The solution is brought to dryness and the oily product was characterized by NMR spectroscopies and high resolution mass spectrometry (HR-MS).

The "Brief Guide to the Nomenclature of Organic Chemistry" was followed for signal assignment,[S1] and when was possible the silylamines were also characterized by comparison with reported data (**6a**,[S7] **6b**,[S7,S8] **6c**,[S7,S9] **6d**,[S7] **6g**,[S7,S9] **7a**,[S9] **7b**,[S10] **7f**,[S11]).

For silylamines with a purity higher than 95% isolated yields are reported. For silylamines with a purity minor than 95%, NMR yields based on ^1H NMR integration using hexamethylbenzene as IS are reported.

Scheme S15. Synthesis of silylamines (**6a** to **7g**).

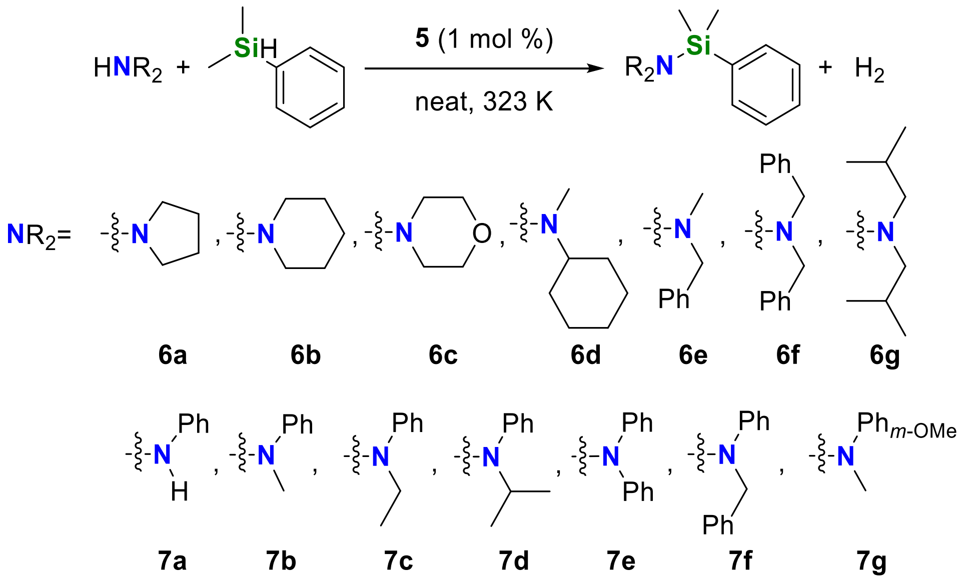

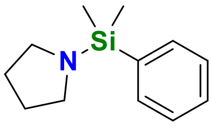

1-(dimethyl(phenyl)silyl)pyrrolidine (6a). ^{1}H NMR (300 MHz, 298 K, C_6D_6): δ 7.55 (m, 2H, Si-*o*-(C_6H_5)), 7.26 - 7.23 (overlapping signals, 3H, Si-*m,p*-(C_6H_5)), 2.92 (m, 4H, CH_2-2 and CH_2-5), 1.56 (m, 4H, CH_2-3 and CH_2-4), 0.34 (s, 6H, Si-(CH_3)$_2$). ^{13}C{^{1}H} (75 MHz, 298 K, C_6D_6): δ 140.2 (s, C^{ipso}), 134.0 - 128.1 (5C, *C*H-Ar), 47.4 (s, 2C, C^2 and C^5), 27.2 (s, 2C, C^3 and C^4), -2.1 (s, 2C, Si-(*C*H$_3$)$_2$). ^{29}Si from the ^{1}H-^{29}Si HMBC NMR (60 MHz, 298 K, C_6D_6): δ –5.0 (s). HRMS (ESI$^+$, *m/z*): calcd. for $C_{12}H_{20}NSi$, $[M+H]^+$ = 206.1365; found = 206.1367. **6a** was obtained as a yellow oil in a 63% isolated yield (65.2 mg, 0.32 mmol).

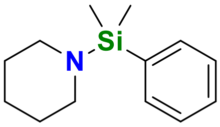

1-(dimethyl(phenyl)silyl)piperidine (6b). ^{1}H NMR (300 MHz, 298 K, C_6D_6): δ 7.57 (m, 2H, Si-*o*-(C_6H_5)), 7.26 - 7.23 (overlapping signals, 3H, Si-*m,p*-(C_6H_5)), 2.81 (m, 4H, CH_2-2 and CH_2-6), 1.48 (m, 4H, CH_2-3 and CH_2-5), 1.34 (m, 2H, CH_2-4), 0.29 (s, 6H, Si-(CH_3)$_2$). ^{13}C{^{1}H} (75 MHz, 298 K, C_6D_6): δ 140.1 (s, C^{ipso}), 134.2 - 128.1 (5C, C*H*-Ar), 47.0 (s, 2C, C^2 and C^6), 28.2 (s, 2C, C^3 and C^5), 26.0 (s, C^4), –1.8 (s, 2C, Si-(*C*H$_3$)$_2$). ^{29}Si from the ^{1}H-^{29}Si HMQC NMR (60 MHz, 298 K, C_6D_6): δ –2.8 (s). HRMS (MALDI, *m/z*): calcd. for $C_{11}H_{17}NNaSi$, $[M-2CH_3+H_2O+Na]^+$ = 230.097; found = 230.034. **6b** was obtained as an orange oil in 81% isolated yield (88.4 mg, 0.40 mmol).

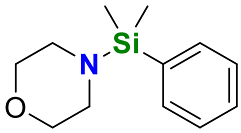

4-(dimethyl(phenyl)silyl)morpholine (6c). ^{1}H NMR (300 MHz, 298 K, C_6D_6): δ 7.49 (m, 2H, Si-*o*-(C_6H_5)), 7.25 - 7.19 (overlapping signals, 3H, Si-*m,p*-(C_6H_5)), 3.41 (m, 4H, CH_2-3 and CH_2-5), 2.69 (m, 4H, CH_2-2 and CH_2-6), 0.21 (s, 6H, Si-(CH_3)$_2$). ^{13}C{^{1}H} (75 MHz, 298 K, C_6D_6): δ 139.1 (s, C^{ipso}), 134.2 - 129.5 (5C, C*H*-Ar), 68.6 (s, 2C, C^3 and C^5), 46.1 (s, 2C, C^2 and C^6), -2.3 (s, 2C, Si-(*C*H$_3$)$_2$). ^{29}Si from the ^{1}H-^{29}Si HMQC NMR (60 MHz, 298 K, C_6D_6): δ –1.7 (s). HRMS (ESI$^+$, *m/z*): calcd. for $C_{12}H_{20}NOSi$, $[M+H]^+$ = 222.1314; found = 222.1311. **6c** was obtained as an air sensitive yellow oil with a conversion of 91% (105.0 mg, 0.47 mmol).

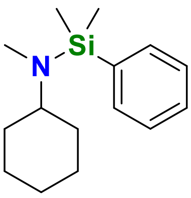

***N*-cyclohexyl-*N*,1,1-trimethyl-1-phenylsilanamine (6d)**. ^{1}H NMR (300 MHz, 298 K, C_6D_6): δ 7.57 (m, 2H, Si-*o*-(C_6H_5)), 7.24 - 7.19 (overlapping signals, 3H, Si-*m,p*-(C_6H_5)), 2.73 (s, 1H, N-C*H*), 2.38 (s, 3H, N-CH_3), 1.77 - 0.90 (overlapping signals, 10H, CH_2-Cy) 0.32 (s, 6H, Si-(CH_3)$_2$). ^{13}C{^{1}H} NMR (75 MHz, 298 K, C_6D_6): δ 139.9 (s, C^{ipso}), 133.3 - 128.1 (5C, *C*H-Ar), 58.6 (s, N-*C*H), 33.5 (s, N-*C*H$_3$), 33.2 - 25.2 (5C, C*H*$_2$-Cy), –1.0 (s, 2C, Si-

($CH_3)_2$). ^{29}Si from the ^{1}H-^{29}Si HMQC NMR (60 MHz, 298 K, C_6D_6): δ –2.3 (s). HRMS (ESI$^+$, *m/z*): calcd. for $C_{15}H_{26}NSi$, $[M+H]^+$ = 248.1835; found = 248.1832. **6d** was obtained as a brown oil in 44% isolated yield (54.9 mg, 0.22 mmol).

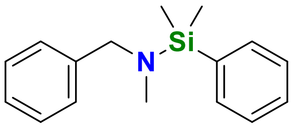

***N*-benzyl-*N*,1,1-trimethyl-1-phenylsilanamine (6e).** ^{1}H NMR (300 MHz, 298 K, C_6D_6): δ 7.53 (m, 2H, Si-*o*-(C_6H_5)), 7.25 - 7.07 (overlapping signals, 8H, CH_2-(C_6H_5) and Si-(C_6H_5)), 3.82 (s, 2H, N-CH_2), 2.31 (s, 3H, N-CH_3), 0.32 (s, 6H, Si-($CH_3)_2$). ^{13}C{^{1}H} NMR (75 MHz, 298 K, C_6D_6): δ 141.5 (s, C^{ipso}), 139.8 (s, C^{ipso}), 134.1 - 126.9 (10C, *C*H-Ar), 54.8 (s, N-CH_2), 34.8 (s, N-CH_3), –1.7 (s, 2C, Si-($CH_3)_2$). ^{29}Si from the ^{1}H-^{29}Si HMQC NMR (60 MHz, 298 K, C_6D_6): δ 0.3 (s). HRMS (ESI$^+$, *m/z*): calcd. for $C_{16}H_{21}NNaSi$, $[M+Na]^+$ = 278.1341; found = 278.0862; calcd. for $C_{14}H_{17}NSi$, $[M-2CH_3+2H]^+$ = 227.1130; found = 227.1548. **6e** was obtained as an air sensitive yellow oil with a conversion of 77% (123.4 mg, 0.48 mmol).

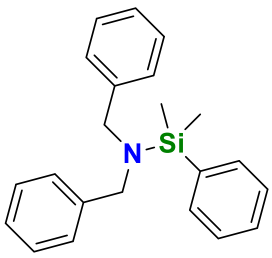

***N*,*N*-dibenzyl-1,1-dimethyl-1-phenylsilanamine (6f).** ^{1}H NMR (300 MHz, 298 K, C_6D_6): δ 7.57 (m, 2H, Si-*o*-(C_6H_5)), 7.27 - 7.08 (overlapping signals, 13H, CH_2-(C_6H_5) and Si-(C_6H_5)), 3.56 (s, 4H, 2 x N-CH_2), 0.32 (s, 6H, Si-($CH_3)_2$). ^{13}C{^{1}H} NMR (75 MHz, 298 K, C_6D_6): δ 141.1 (s, 2C, C^{ipso}), 139.9 (s, C^{ipso}), 133.4 - 127.1 (15C, *C*H-Ar), 53.4 (s, 2C, N-CH_2), 1.0 (s, 2C, Si-($CH_3)_2$). ^{29}Si from the ^{1}H-^{29}Si HMQC NMR (60 MHz, 298 K, C_6D_6): δ 0.2 (s). HRMS (ESI$^+$, *m/z*): calcd. for $C_{22}H_{26}NSi$, $[M+H]^+$ = 332.1835; found = 332.1832. **6f** was obtained as a brilliant yellow oil in 89% isolated yield (148 mg, 0.45 mmol).

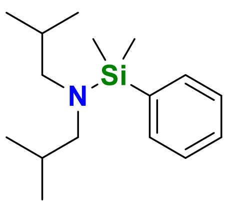

***N*,*N*-diisobutyl-1,1-dimethyl-1-phenylsilanamine (6g).** ^{1}H NMR (300 MHz, 298 K, C_6D_6): δ 7.59 (m, 2H, Si-*o*-(C_6H_5)), 7.28 - 7.20 (overlapping signals, 3H, Si-*m,p*-(C_6H_5)), 2.57 (d, $^3J_{HH}$ = 7.3 Hz, 4H, 2 x N-CH_2), 1.75 (m, 2H, 2 x C*H*-CH_2), 0.79 (d, $^3J_{HH}$ = 6.6 Hz, 12H, 2 x ($CH_3)_2$-CH), 0.36 (s, 6H, Si-($CH_3)_2$). ^{13}C{^{1}H} NMR (75 MHz, 298 K, C_6D_6): δ 140.4 (s, C^{ipso}), 134.4 - 128.0 (5C, *C*H-Ar), 55.3 (s, 2C, N-CH_2), 26.9 (s, 2C, *C*H-CH_2), 20.7 (s, 4C, ($CH_3)_2$-CH), –0.6 (s, 2C, Si-($CH_3)_2$). ^{29}Si from the ^{1}H-^{29}Si HMQC NMR (60 MHz, 298 K, C_6D_6): δ –1.7 (s). HRMS (ESI$^+$, *m/z*): calcd. for $C_{16}H_{30}NSi$, $[M+H]^+$ = 264.2148; found = 264.2131. **6g** was obtained as a light and air sensitive pale yellow oil with a conversion of 88% (130.1 mg, 0.49 mmol).

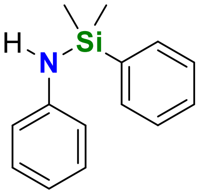

1,1-dimethyl-*N*,1-diphenylsilanamine (7a). ^{1}H NMR (300 MHz, 298 K, C_6D_6): δ 7.69 (m, 2H, Si-*o*-(C_6H_5)), 7.33 (overlapping signals, 3H, Si-*m,p*-(C_6H_5)), 7.16 (m, 2H, N-*m*-(C_6H_5)), 6.83 (m, 1H, N-*p*-(C_6H_5)), 6.70 (m, 2H, N-*o*-(C_6H_5)), 3.38 (s, 1H, N-*H*), 0.46 (s, 6H, Si-($CH_3)_2$). ^{13}C{^{1}H} NMR (75 MHz, 298 K, C_6D_6): δ 147.3 (s, C^{ipso}), 138.5 (s, C^{ipso}), 134.0 - 117.0 (10C, *C*H-Ar), –1.3 (s, 2C, Si-($CH_3)_2$). ^{29}Si from the ^{1}H-^{29}Si HMQC NMR (60 MHz, 298 K, C_6D_6): δ –5.2 (s). HRMS (ESI$^+$, *m/z*): calcd. for $C_{14}H_{18}NSi$, $[M+H]^+$ = 228.1209; found = 228.1204. **7a** was obtained as a light yellow oil in 88% isolated yield (106.4 mg, 0.45 mmol).

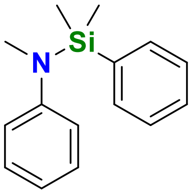

***N*,1,1-trimethyl-*N*,1-diphenylsilanamine (7b)**. ^{1}H NMR (300 MHz, 298 K, C_6D_6): δ 7.48 (m, 2H, Si-*o*-(C_6H_5)), 7.20 - 6.76 (overlapping signals, 8H, N-(C_6H_5) and Si-(C_6H_5)), 2.75 (s, 3H, N-CH_3), 0.36 (s, 6H, Si-($CH_3)_2$). ^{13}C{^{1}H} NMR (75 MHz, 298 K, C_6D_6): δ 151.0 (s, C^{ipso}), 139.4 (s, C^{ipso}), 133.9 - 118.9 (10C, *C*H-Ar), 35.9 (s, N-CH_3), –0.4 (s, 2C, Si-($CH_3)_2$). ^{29}Si from the ^{1}H-^{29}Si HMQC NMR (60 MHz, 298 K, C_6D_6): δ –1.1 (s). HRMS (ESI$^+$, *m/z*): calcd. for $C_{15}H_{20}NSi$, $[M+H]^+$ = 242.1365; found = 242.1360. **7b** was obtained as an orange oil in 93% isolated yield (0.90 g, 3.7 mmol).

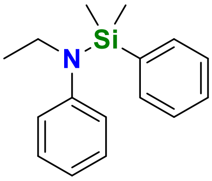

***N*-ethyl-1,1-dimethyl-*N*,1-diphenylsilanamine (7c)**. ^{1}H NMR (300 MHz, 298 K, C_6D_6): δ 7.56 (m, 2H, Si-*o*-(C_6H_5)), 7.21 - 6.77 (overlapping signals, 8H, N-(C_6H_5) and Si-(C_6H_5)), 3.25 (q, $^{3}J_{HH}$ = 7.0 Hz, 2H, CH_2-CH_3), 0.99 (t, $^{3}J_{HH}$ = 7.0 Hz, 3H, CH_2-CH_3), 0.37 (s, 6H, Si-($CH_3)_2$). ^{13}C{^{1}H} NMR (75 MHz, 298 K, C_6D_6): δ 148.8 (s, C^{ipso}), 139.7(s, C^{ipso}), 134.0 - 120.6 (10C, *C*H-Ar), 42.6 (s, CH_2-CH_3), 15.7 (s, CH_2-CH_3), –0.3 (s, 2C, Si-($CH_3)_2$). ^{29}Si from the ^{1}H-^{29}Si HMQC NMR (60 MHz, 298 K, C_6D_6): δ –1.1 (s). HRMS (MALDI, *m/z*): calcd. for $C_{13}H_{13}NNaSi$, $[M-3CH_3+Na]^+$ = 234.071; found = 234.133; calcd. for $C_{14}H_{15}NNaSi$, $[M-2CH_3+Na]^+$ = 248.087; found = 248.155. **7c** was obtained as a light and air sensitive pale yellow oil in a 93% isolated yield (119.4 mg, 0.47 mmol).

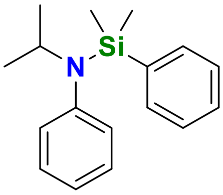

***N*-Isopropyl-1,1-dimethyl-*N*,1-diphenylsilanamine (7d)**. ^{1}H NMR (400 MHz, 298 K, C_6D_6): δ 7.64 (m, 2H, Si-*o*-(C_6H_5)), 7.25 - 6.98 (overlapping signals, 8H, N-(C_6H_5) and Si-(C_6H_5)), 3.47 (hept, $^{3}J_{HH}$ = 6.6 Hz, 1H, C*H*-N), 0.93 (d, $^{3}J_{HH}$ = 6.6 Hz, 6H,

(CH_3)$_2$-CH), 0.26 (s, 6H, Si-(C*H*$_3$)$_2$). ^{13}C{^{1}H} NMR (75 MHz, 298 K, C_6D_6): δ 144.7 (s, *C*^{ipso}), 140.4 (s, *C*^{ipso}), 134.2 - 125.1 (10C, *C*H-Ar), 49.2 (s, C*H*-N), 23.7 (s, 2C, (*C*H$_3$)$_2$-CH), –0.6 (s, 2C, Si-(*C*H$_3$)$_2$). ^{29}Si from the ^{1}H-^{29}Si HMQC NMR (60 MHz, 298 K, C_6D_6): δ –3.9 (s). HRMS (ESI$^+$, *m/z*): calcd. for $C_{18}H_{25}N_2Si$, [M-CH_3+H+CH_3CN+H]$^+$ = 297.1787; found = 297.2341; calcd. for $C_{28}H_{41}N_3NaOSi$, [M+Na+H_2O+CH_3CN+amine]$^+$ = 486.2917; found = 486.3223. **7d** was obtained as a light and air sensitive yellow oil with a conversion of 91% (129.9 mg, 0.48 mmol).

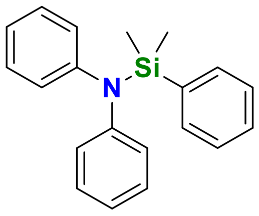

1,1-dimethyl-*N*,*N*,1-triphenylsilanamine (7e). ^{1}H NMR (400 MHz, 298 K, C_6D_6): δ 7.61 (m, 2H, Si-*o*-(C_6*H*$_5$)), 7.17 - 6.77 (overlapping signals, 13H, N-(C_6*H*$_5$) and Si-(C_6*H*$_5$)), 0.28 (s, 6H, Si-(C*H*$_3$)$_2$). ^{13}C{^{1}H} NMR (75 MHz, 298 K, C_6D_6): δ 148.6 (s, *C*^{ipso}), 143.6 (s, *C*^{ipso}), 139.1 (s, *C*^{ipso}), 136.2 - 118.2 (15C, *C*H-Ar), 0.15 (s, 2C, Si-(*C*H$_3$)$_2$). ^{29}Si from the ^{1}H-^{29}Si HMQC NMR (60 MHz, 298 K, C_6D_6): δ –2.0 (s). HRMS (ESI$^+$, *m/z*): calcd. for $C_{21}H_{23}N_2NaOSi$, [M-CH_3+Na+H_2O+CH_3CN]$^+$ = 370.1477; found = 370.1577; calcd. for $C_{32}H_{34}N_2NaOSi$, [M+Na+H_2O+amine]$^+$ = 513.2338; found = 513.2078. **7e** was obtained as a colourless oil in 70% isolated yield (106.2 mg, 0.35 mmol).

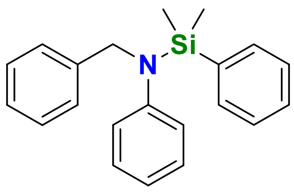

***N*-benzyl-1,1-dimethyl-*N*,1-diphenylsilanamine (7f)**. ^{1}H NMR (400 MHz, 298 K, C_6D_6): δ 7.53 (m, 2H, Si-*o*-(C_6*H*$_5$)), 7.17 - 6.92 (overlapping signals, 12H, CH_2-(C_6*H*$_5$), N-(C_6*H*$_5$) and Si-(C_6*H*$_5$)), 6.66 (m, 1H, C*H*-Ar), 4.47 (s, 2H, N-C*H*$_2$), 0.37 (s, 6H, Si-(C*H*$_3$)$_2$). ^{13}C{^{1}H} NMR (75 MHz, 298 K, C_6D_6): δ 149.8 (s, *C*^{ipso}), 141.5 (s, *C*^{ipso}), 139.1(s, *C*^{ipso}), 134.0 - 119.8 (15C, *C*H-Ar), 52.8 (s, N-*C*H$_2$), –0.3 (s, 2C, Si-(*C*H$_3$)$_2$). ^{29}Si from the ^{1}H-^{29}Si HMQC NMR (60 MHz, 298 K, C_6D_6): δ 0.7 (s). HRMS (ESI$^+$, *m/z*): calcd. for $C_{21}H_{24}NSi$, [M+H]$^+$ = 318.1678; found = 318.1673. **7f** was obtainted as a light yellow oil in 83% isolated yield (131.0mg, 0.41 mmol).

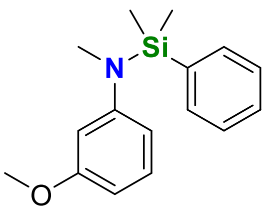

***N*-(3-methoxyphenyl)-*N*,1,1-trimethyl-*N*,1-diphenylsilan amine (7g)**. ^{1}H NMR (400 MHz, 298 K, C_6D_6): δ 7.48 (m, 2H, Si-*o*-(C_6*H*$_5$)), 7.22 - 7.16 (overlapping signals, 3H, Si-*m*,*p*-(C_6*H*$_5$)), 7.00 (t, $^3J_{HH}$ = 8.1 Hz, 1H, *H*^{5}), 6.64 (m, 1H, *H*^{2}), 6.57 (ddd, $^3J_{HH}$ = 8.1 Hz, $^4J_{HH}$ = 3.1 Hz, $^4J_{HH}$ = 2.3 Hz, 1H, *H*^{4}), 6.37 (ddd, $^3J_{HH}$ = 8.1 Hz, $^4J_{HH}$ = 3.1 Hz, $^4J_{HH}$ = 2.3 Hz, 1H, *H*^{6}), 3.32 (s, 3H, O-C*H*$_3$), 2.76 (s, 3H, N-C*H*$_3$), 0.38 (s, 6H,

Si-(CH_3)$_2$). $^{13}C\{^1H\}$ NMR (75 MHz, 298 K, C_6D_6): δ 161.0 (s, C^{ipso}), 152.4 (s, C^{ipso}), 139.4 (s, C^{ipso}), 133.9 (s, 2C, Si-*o*-(C_6H_5)), 130.1 (s, C^5), 129.6 (3C, Si-*m*,*p*-(C_6H_5)), 111.5 (s, C^4), 105.1 (s, C^2), 104.7 (s, C^6), 54.6 (s, O-CH_3), 35.8 (s, N-CH_3), –0.4 (s, 2C, Si-(CH_3)$_2$). ^{29}Si from the ^{1}H-^{29}Si HMQC NMR (60 MHz, 298 K, C_6D_6): δ 0.6 (s). HRMS (ESI$^+$, *m/z*): calcd. for $C_{16}H_{22}NOSi$, $[M+H]^+$ = 272.1471; found = 272.1472. **7g** was obtained as a light sensitive orange oil with a conversion of 84% (119.8 mg, 0.44 mmol).

10. References

S1. Hellwich, K.-H; Hartshorn, R. M.; Yerin, A.; Damhus, T; Hutton, A. T. *Pure Appl. Chem.* **2020**, *92*, 527–539.

S2. Van Der Ent, A.; Onderdelinden, A. L.; Schunn, R. A. *Inorg. Synth.* **1990**, *28*, 90–92.

S3. Kozuch, S.; Martin, J. M. L. *ACS Catal.* **2012**, *2*, 2787–279.

S4. The reactions were carried out in a Man on the Moon™ X102 microreactor, https://manonthemoontech.com/, and monitored by H_2 evolution during the CDC reaction.

S5. Dittmer, D. C.; Marcantonio, A. F. Kinetic Isotope Effect in the Reaction of *N*-Methylaniline-*N-d* and 3-Chloro-1-butene. *J. Am. Chem. Soc.* **1964**, *86*, 5621–5626.

S6. Gandhamsetty, N.; Park, S.; Chang, S. Selective Silylative Reduction of Pyridines Leading to Structurally Diverse Azacyclic Compounds with the Formation of sp^3 C–Si Bonds. *J. Am. Chem. Soc*. **2015**, *137*, 15176–15184.

S7. Ojeda-Amador, E A. I.; Munarriz, J.; Alamán-Valtierra, P.; Polo, V.; Puerta-Oteo, R.; Jiménez, M. V.; Fernández-Alvarez, F. J.; Pérez-Torrente, J. J. *ChemCatChem* **2019**, *11*, 5524.

S8. Kroesen, U.; Unkelbach, C.; Schildbach, D.; Strohmann, C. *Angew. Chem. Int. Ed.* **2017**, *56*, 14164–14168.

S9. Mitsudome, T.; Urayama, T.; Maeno, Z.; Mizugaki, T.; Jitsukama, K.; Kaneda, K.; *Chem. Eur. J.* **2015**, *21*, 3202–3205.

S10. Königs, C. D. F.; Müller, M. F.; Aiguabella, N.; Klare, H. F. T.; Oestreich, M.; *Chem. Commun*. **2013**, *49*, 1506–1508.

S11. Fasano, V.; LaFortune, J. H. W.; Bayne, J. M.; Ingleson, M. J.; Stephan, D. W. *Chem. Commun.* **2018**, *54*, 662–665.

11. NMR spectra

11.1. NMR spectra of proligands and Ir-complexes

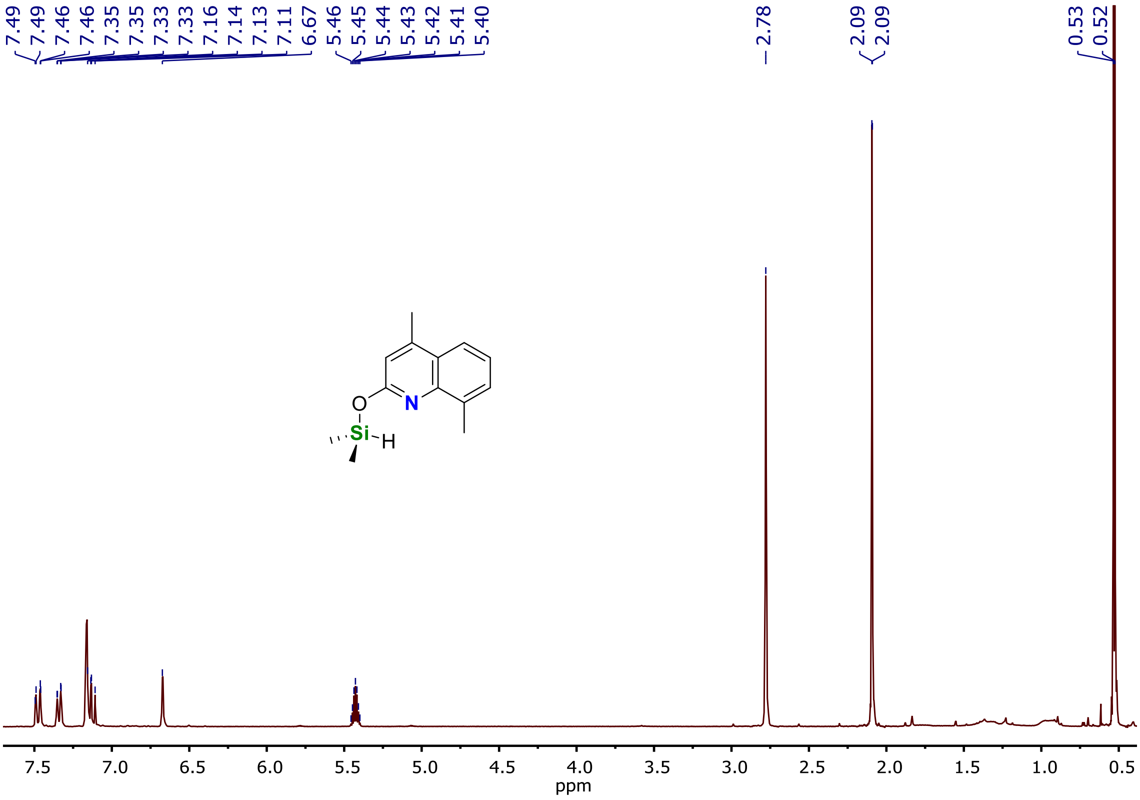

Figure S15. ^{1}H NMR spectrum of **1** in C_6D_6 (300 MHz, 298K).

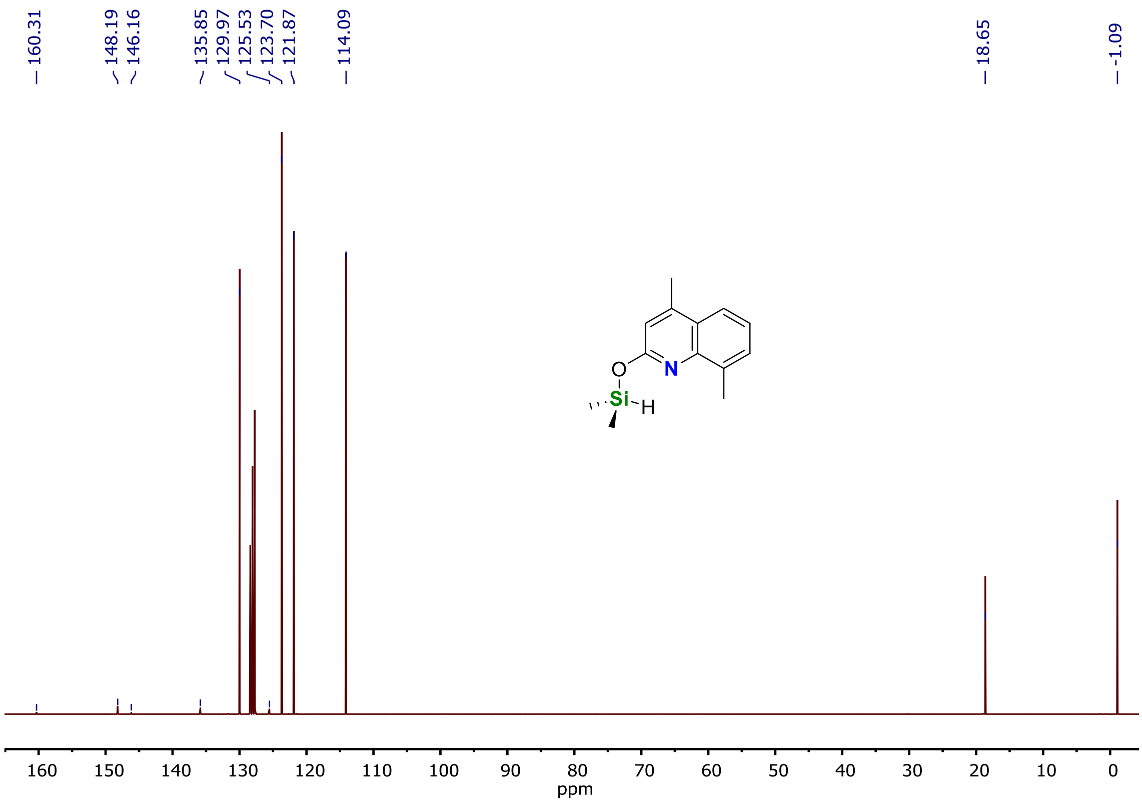

Figure 6 $^{13}C\{^{1}H\}$ NMR spectrum of **1** in C_6D_6 (75 MHz, 298K).

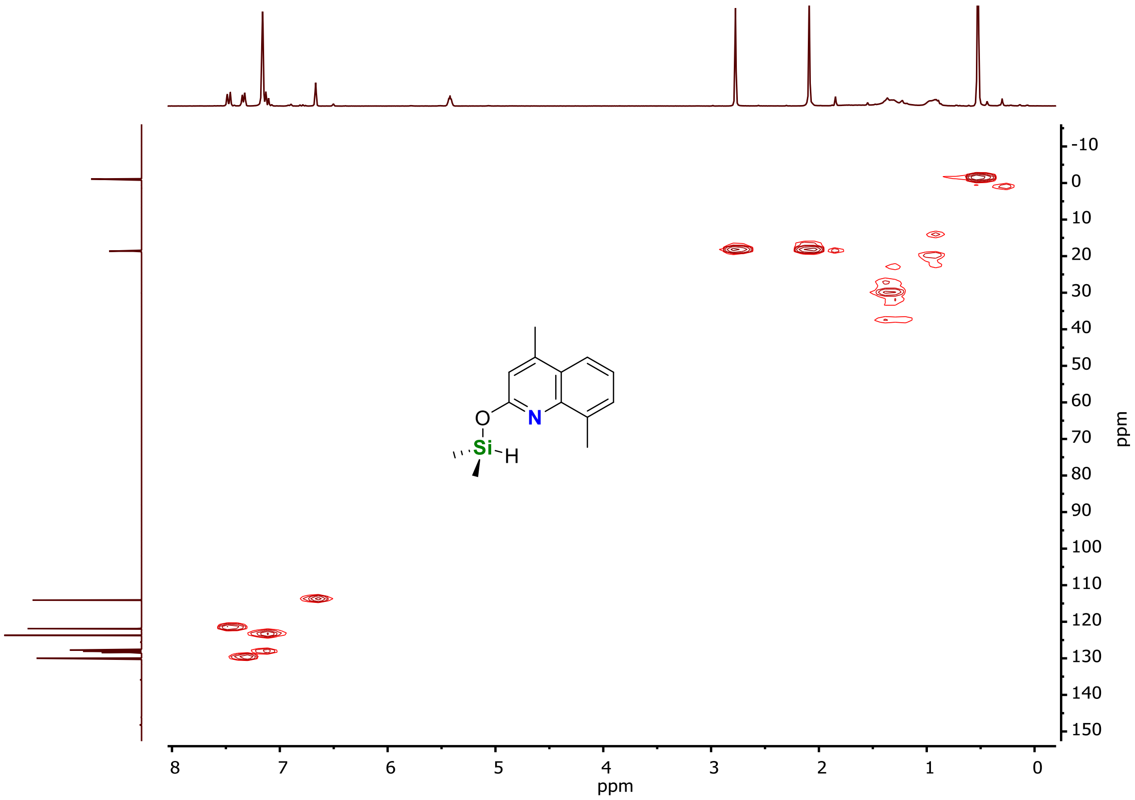

Figure S17. ^{1}H-^{13}C HSQC NMR spectrum of **1** in C_6D_6 (298K).

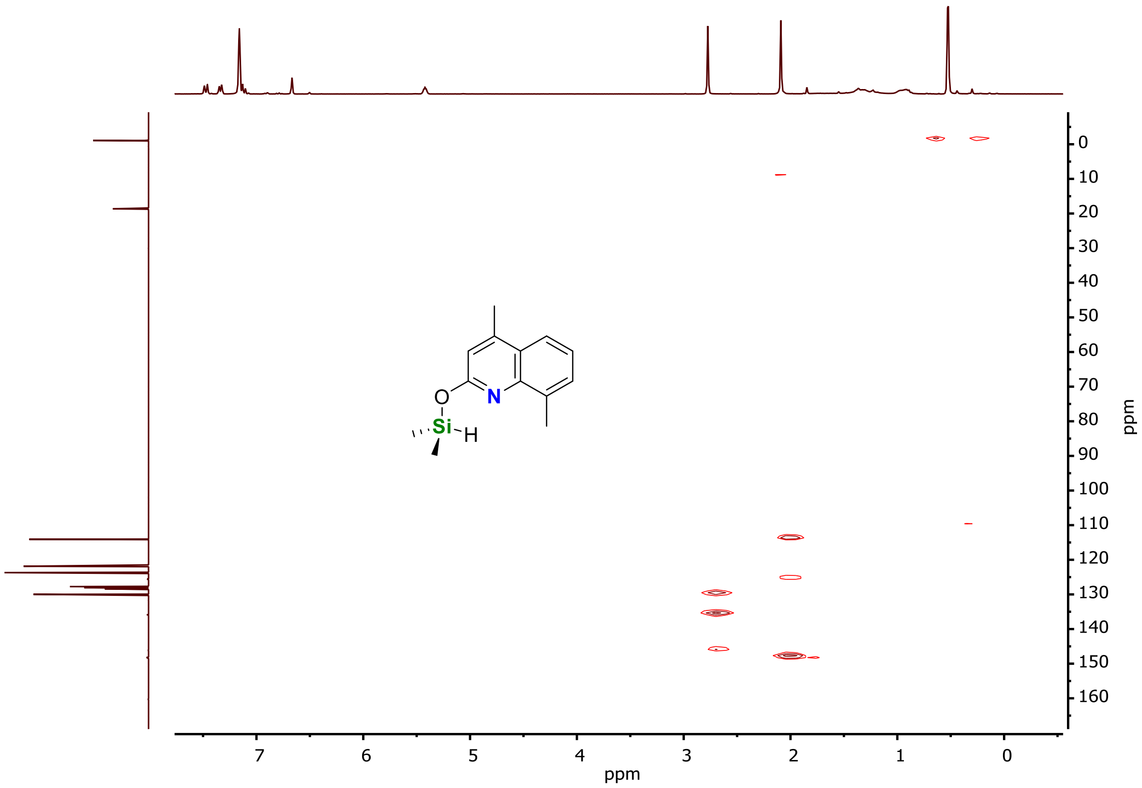

Figure S18. ^{1}H-^{13}C HMBC NMR spectrum of **1** in C_6D_6 (298K).

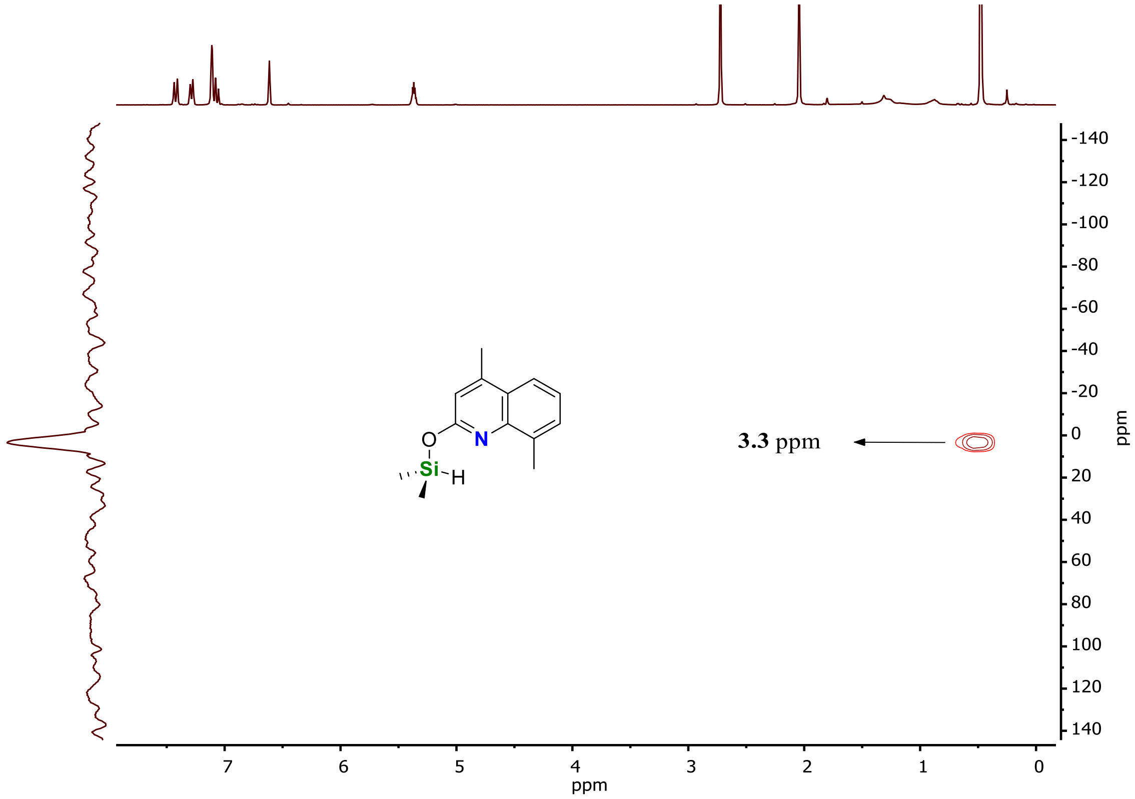

Figure S19. ^{1}H-^{29}Si HMBC NMR spectrum of **1** in C_6D_6 (298K).

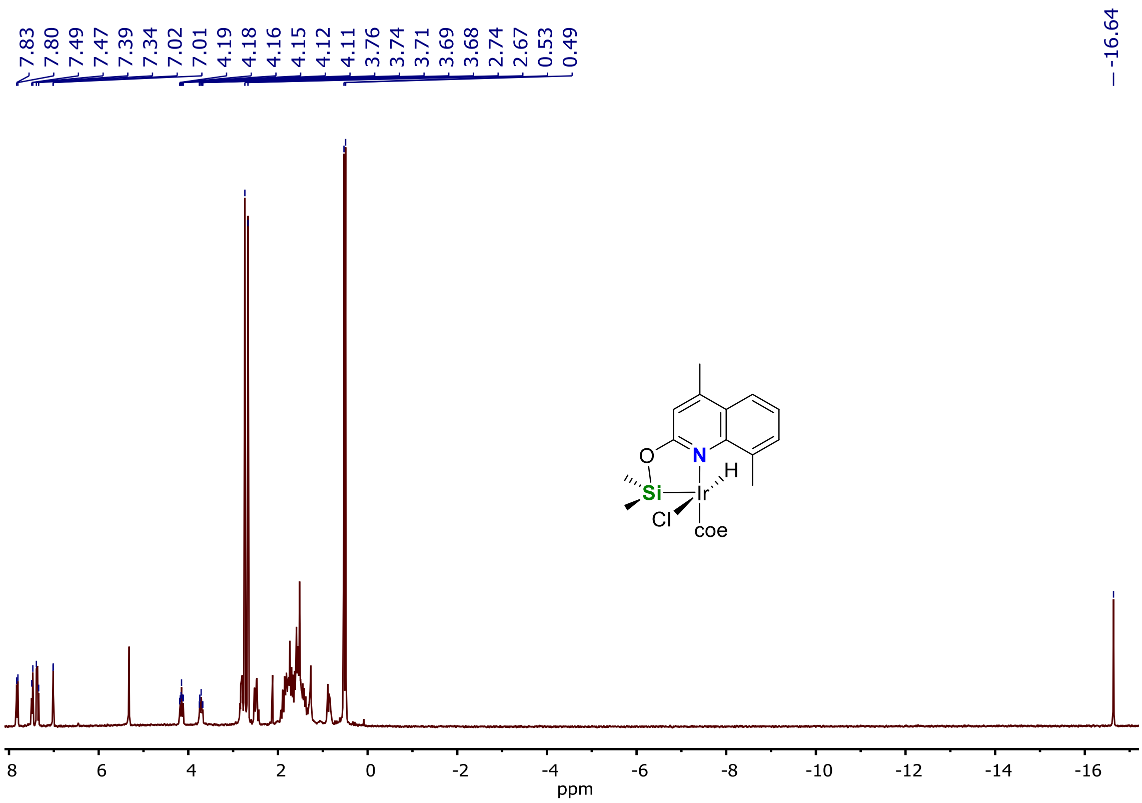

Figure S20. ^{1}H NMR spectrum of **2** in CD_2Cl_2 (300 MHz, 298K).

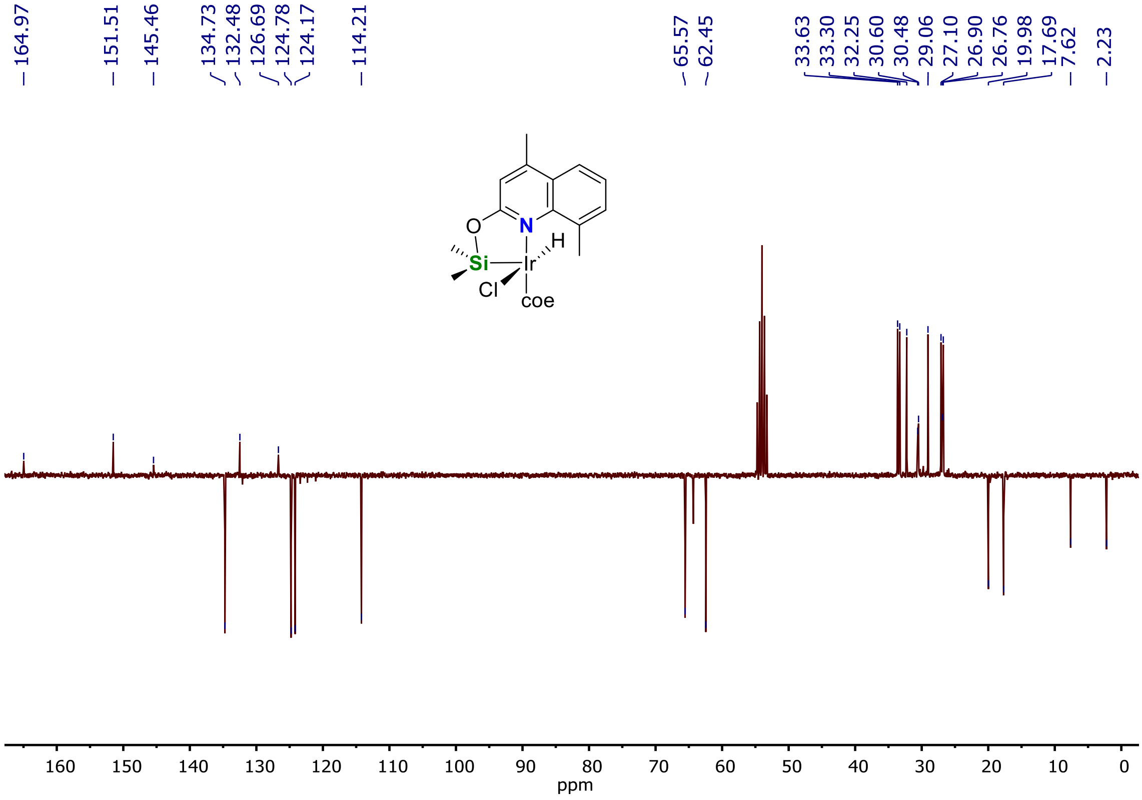

Figure S21. ^{13}C APT NMR spectrum of **2** in CD_2Cl_2 (75 MHz, 298K).

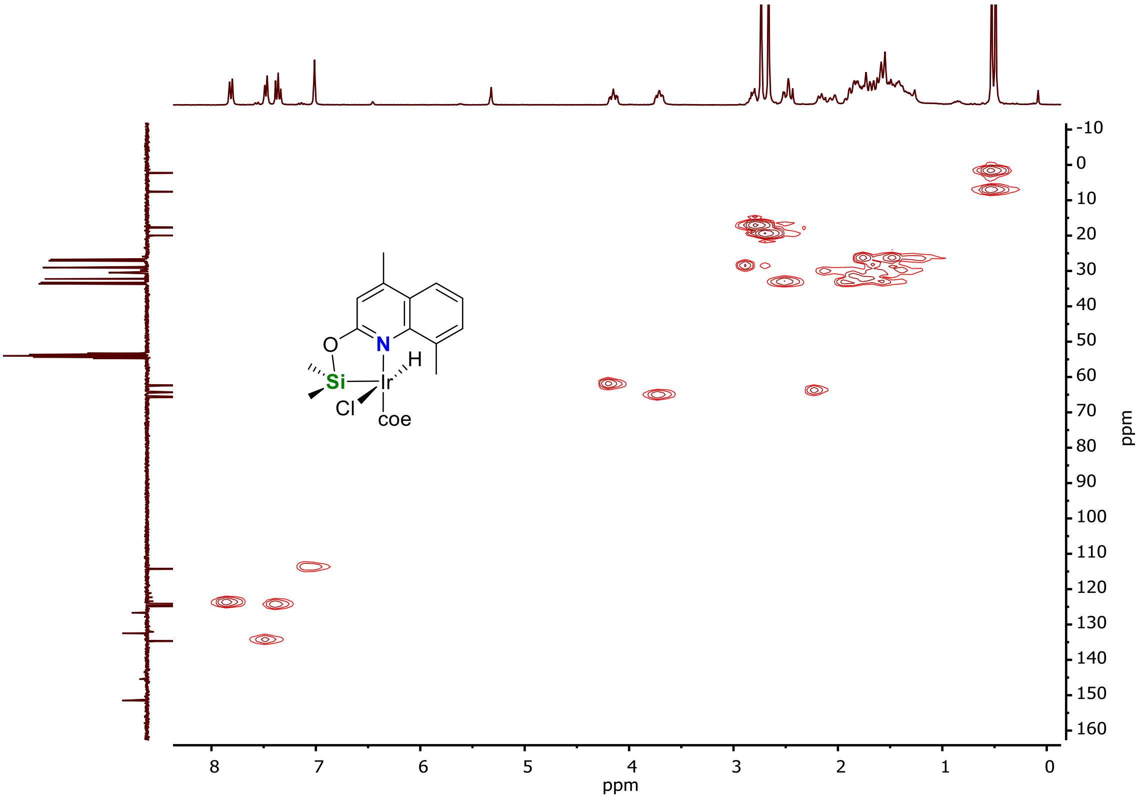

Figure S22. ^{1}H-^{13}C HSQC NMR spectrum of **2** in CD_2Cl_2 (298K).

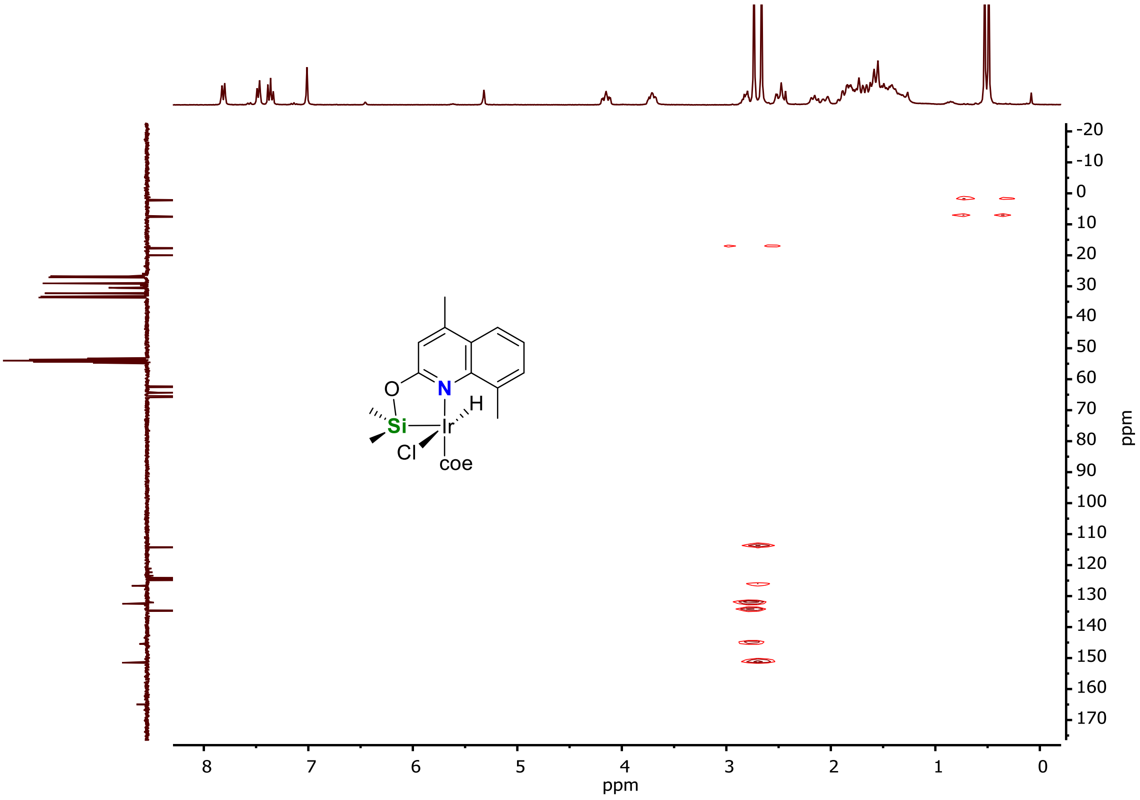

Figure S23. ^{1}H-^{13}C HMBC NMR spectrum of **2** in CD_2Cl_2 (298K).

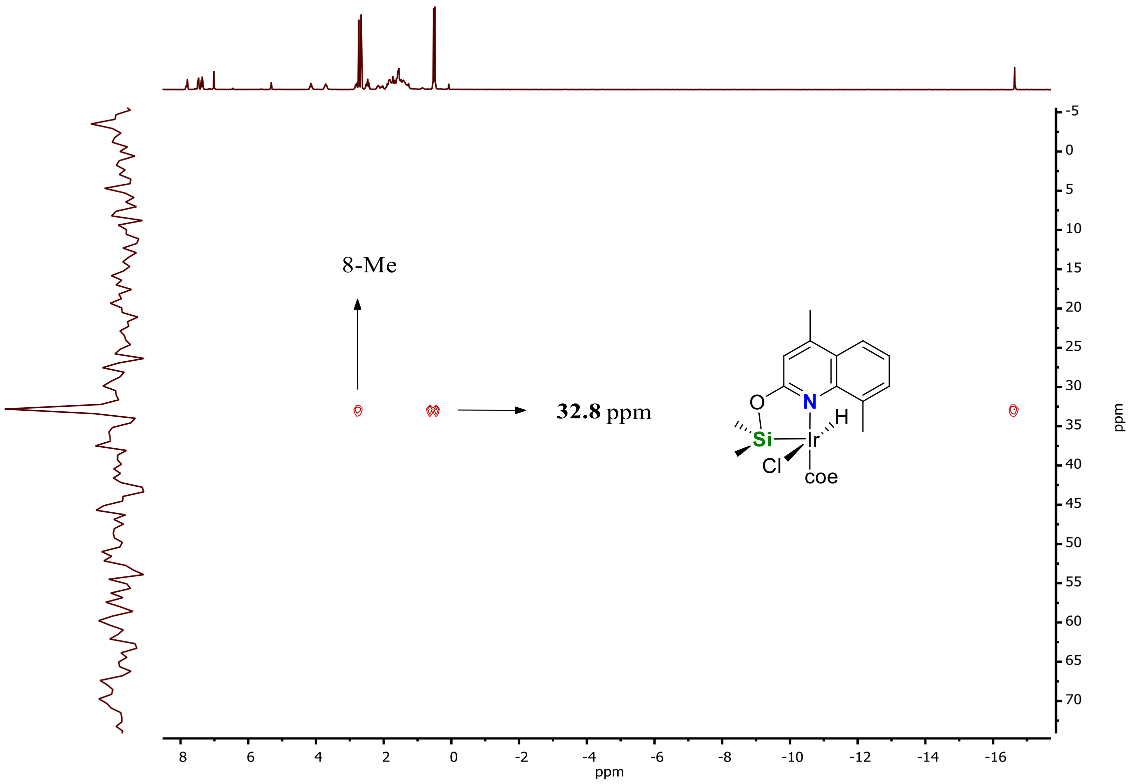

Figure S24. ^{1}H-^{29}Si HMBC NMR spectrum of **2** in CD_2Cl_2 (298K).

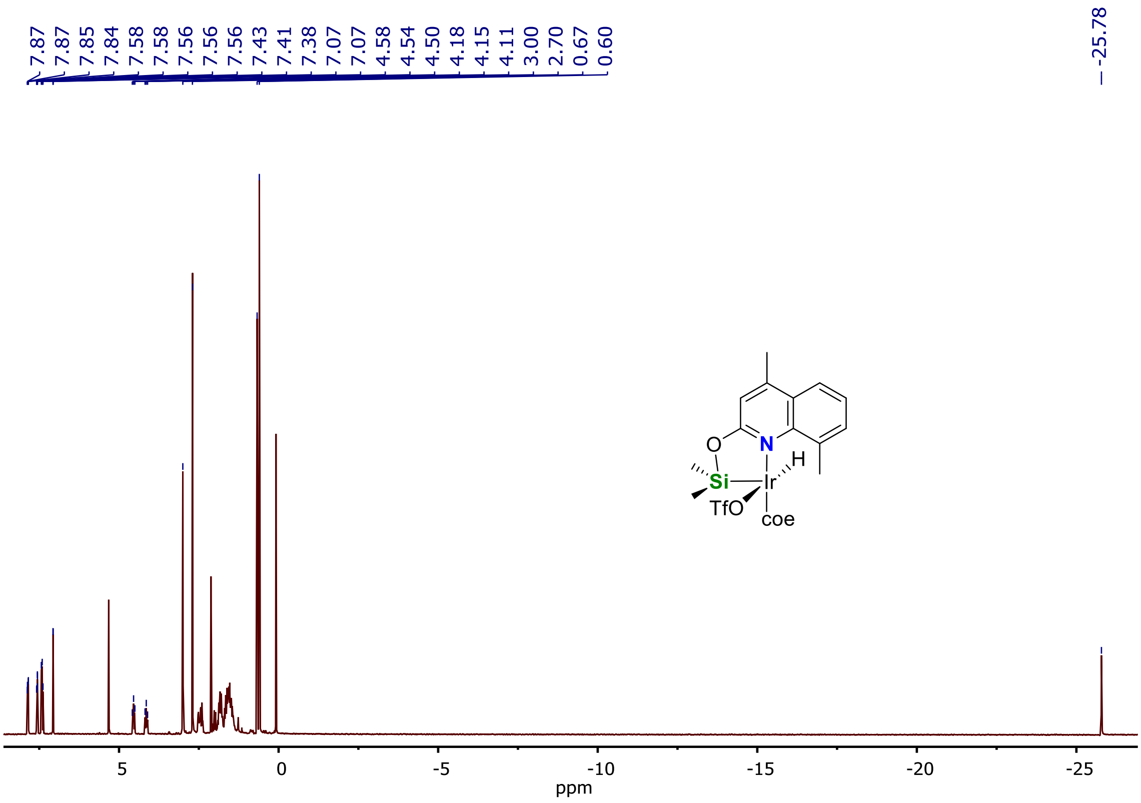

Figure S25. ^{1}H NMR spectrum of **3** in CD_2Cl_2 (300 MHz, 298K).

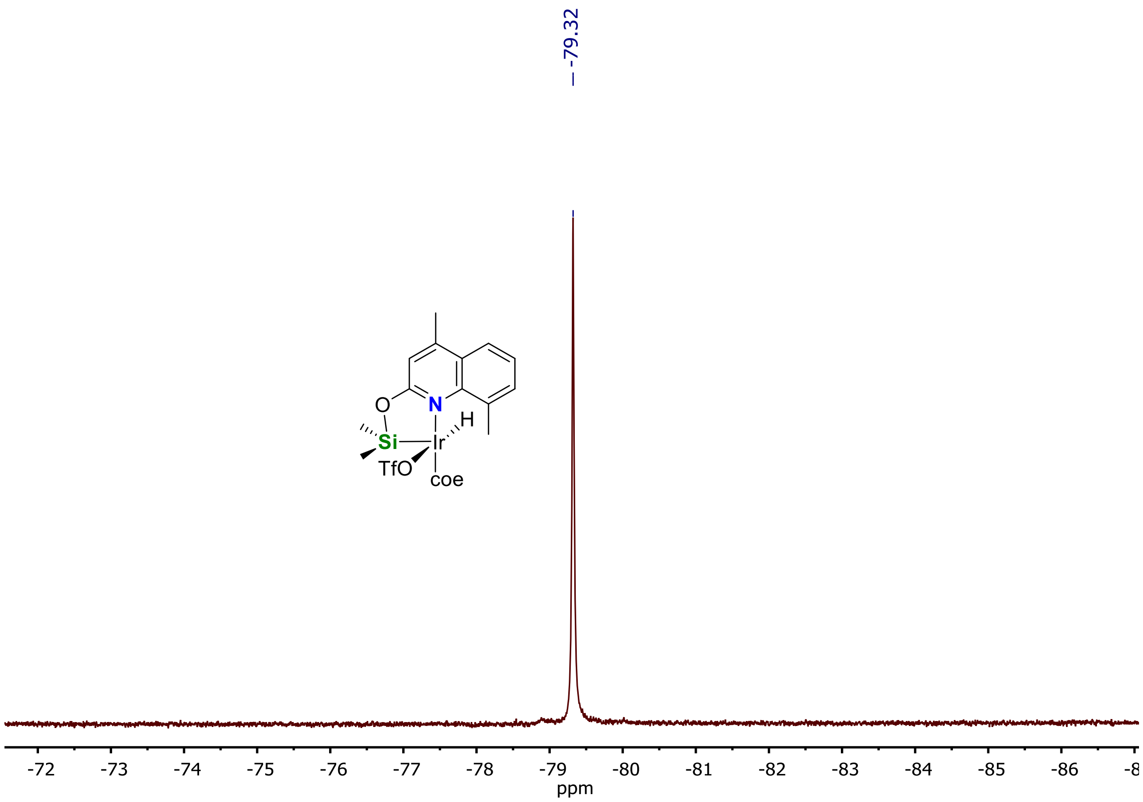

Figure S26. ^{19}F NMR spectrum of **3** in CD_2Cl_2 (282 MHz, 298K).

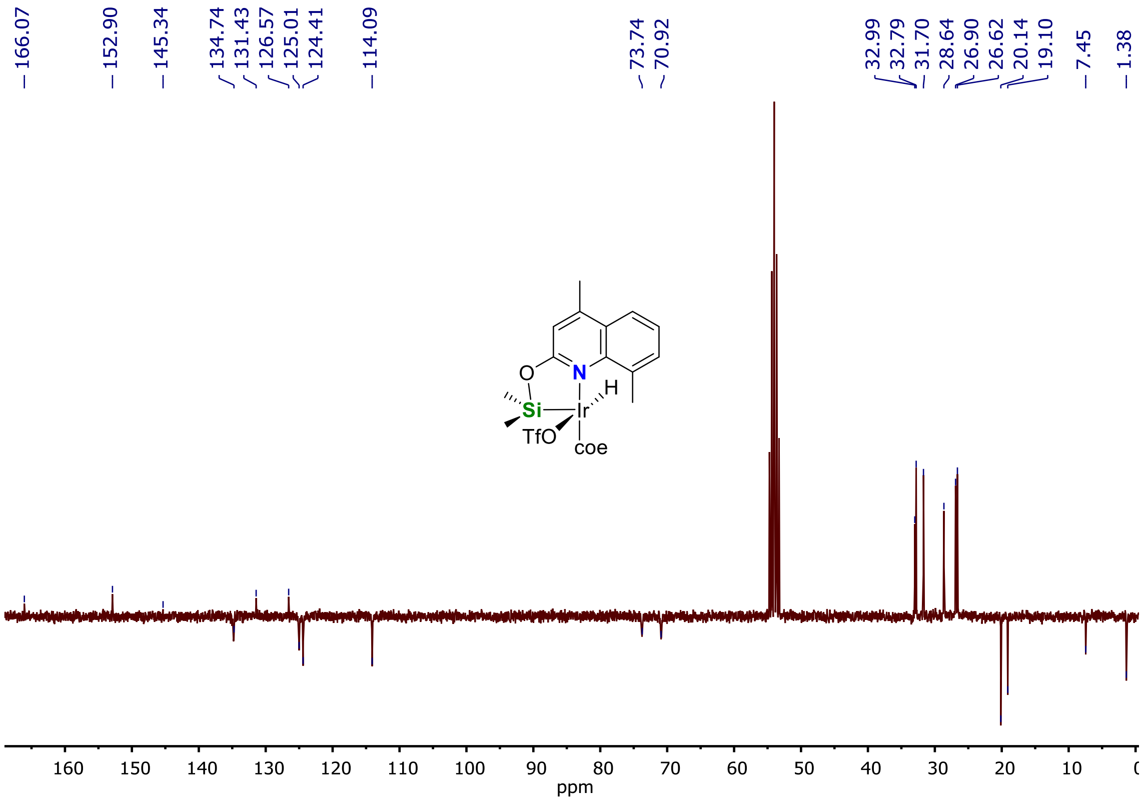

Figure S27. ^{13}C APT NMR spectrum of **3** in CD_2Cl_2 (75 MHz, 298K).

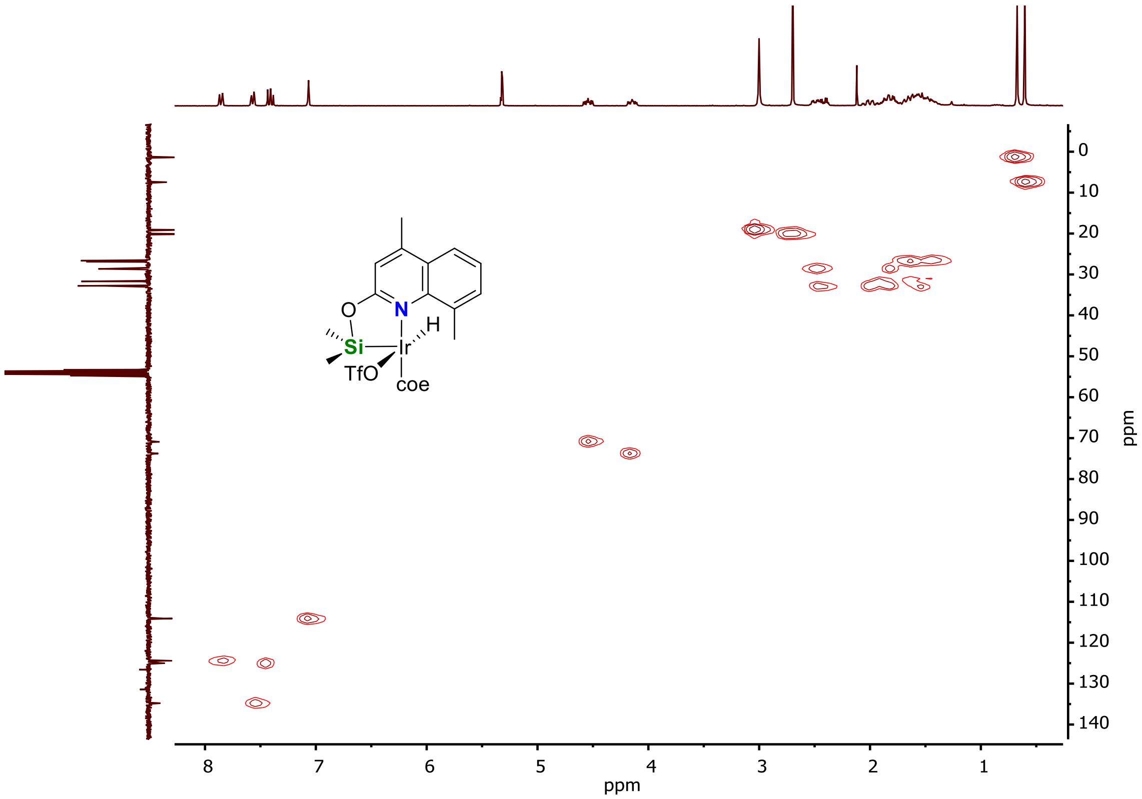

Figure S28. ¹H-¹³C HSQC NMR spectrum of **3** in CD_2Cl_2 (298K).

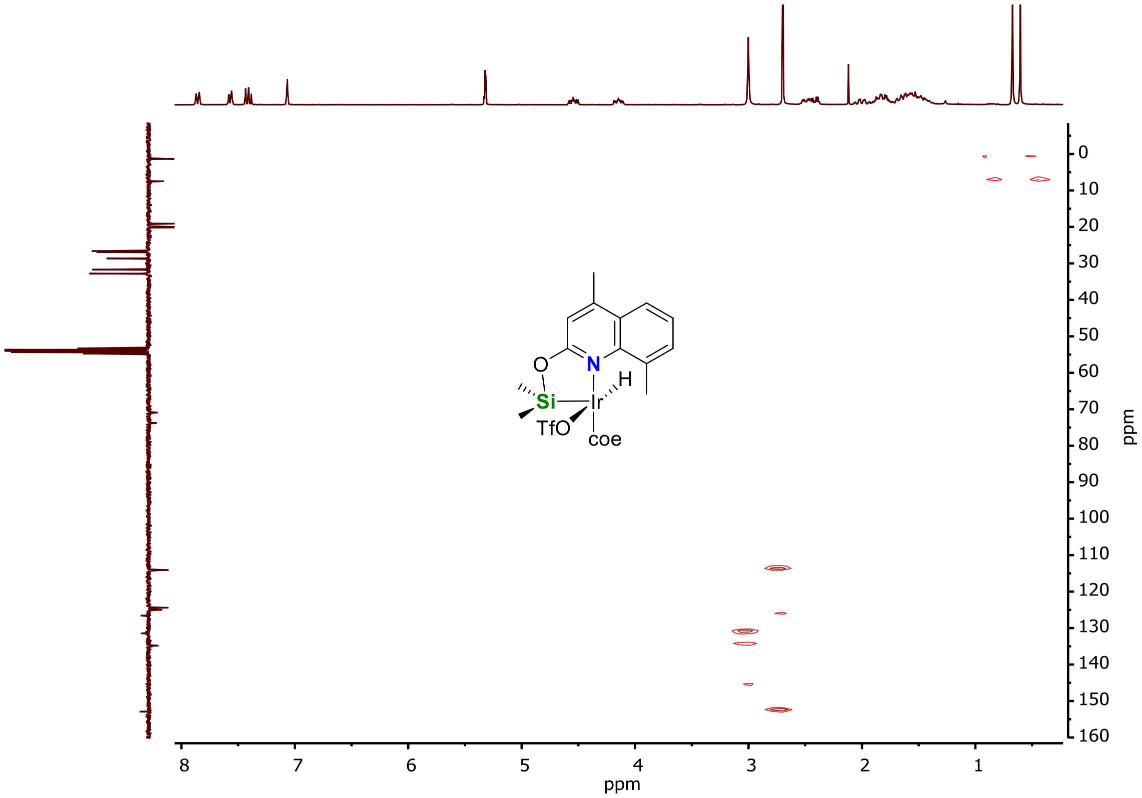

Figure S29. ¹H-¹³C HMBC NMR spectrum of **3** in CD_2Cl_2 (298K).

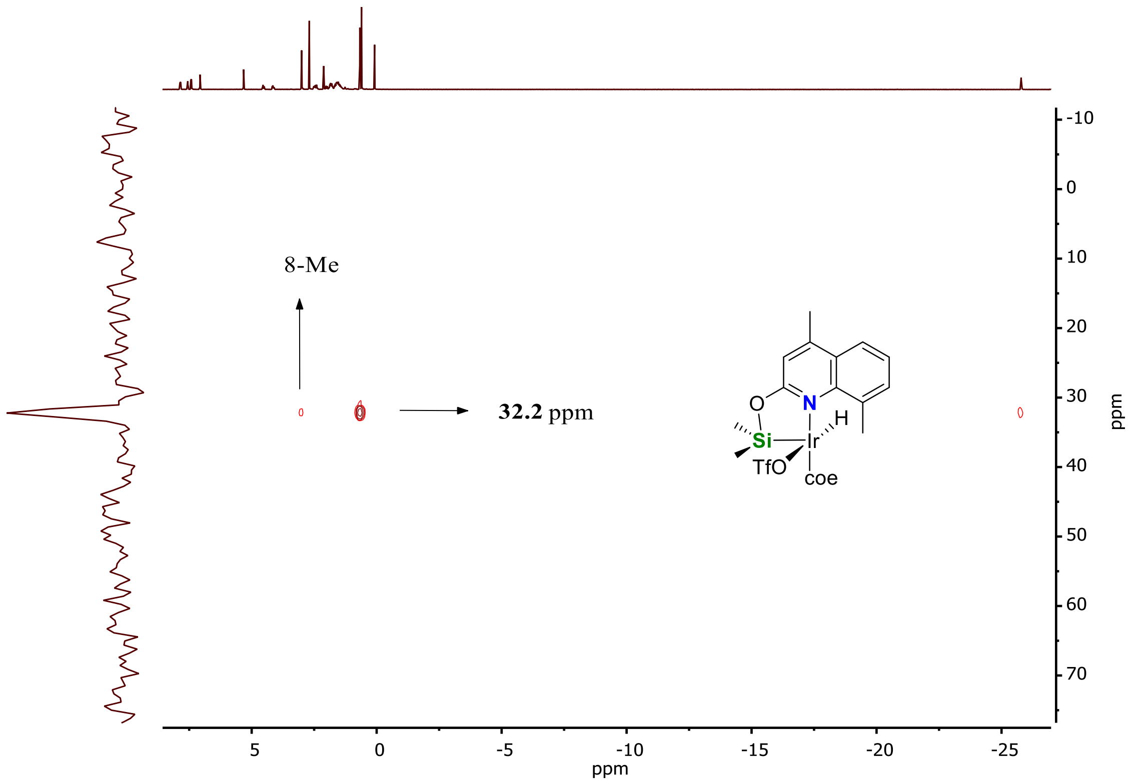

Figure S30. ^{1}H-^{29}Si HMBC NMR spectrum of **3** in CD_2Cl_2 (298K).

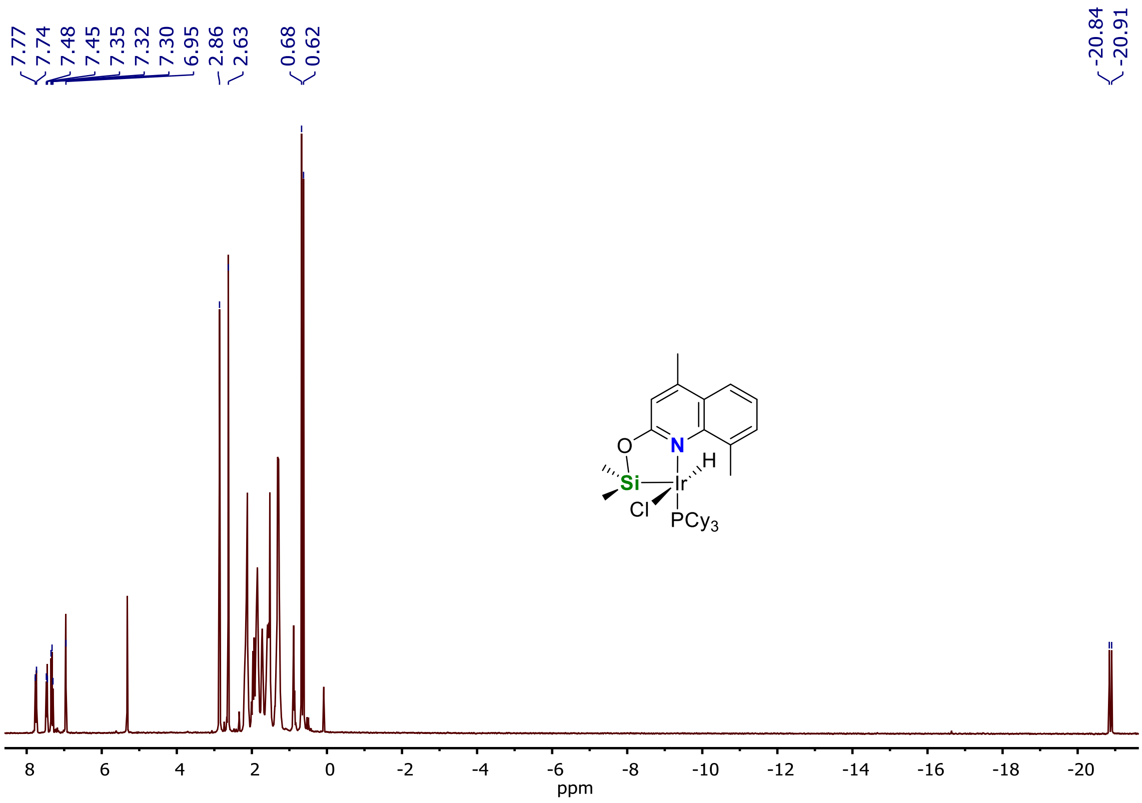

Figure S31. ^{1}H NMR spectrum of **4** in CD_2Cl_2 (300 MHz, 298K).

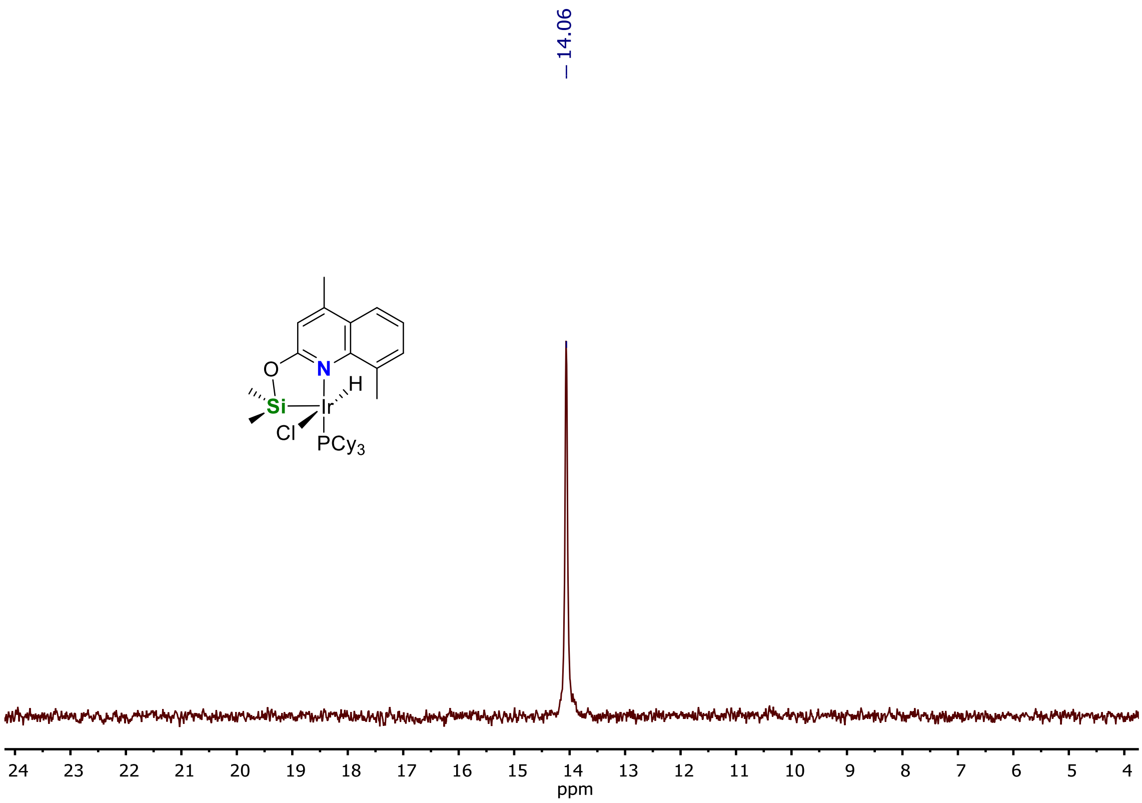

Figure S32. $^{31}P\{^{1}H\}$ NMR spectrum of **4** in CD_2Cl_2 (121 MHz, 298K).

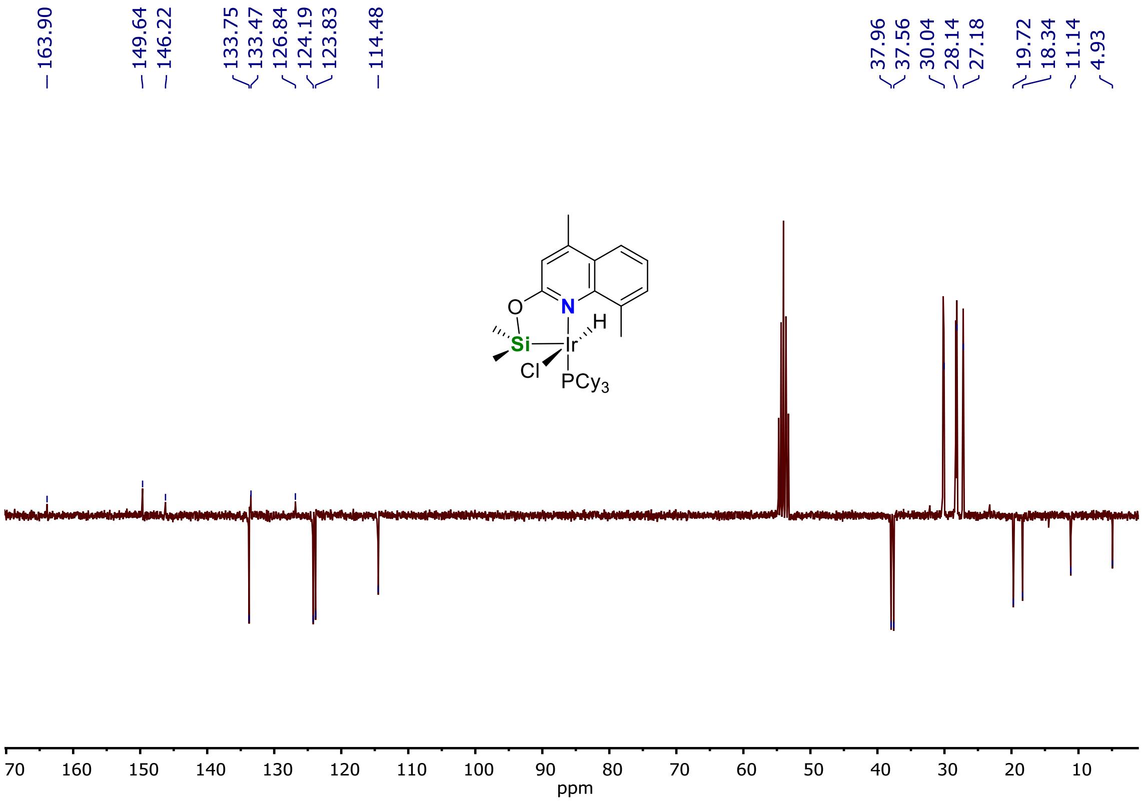

Figure S33. ^{13}C APT NMR spectrum of **4** in CD_2Cl_2 (75 MHz, 298K).

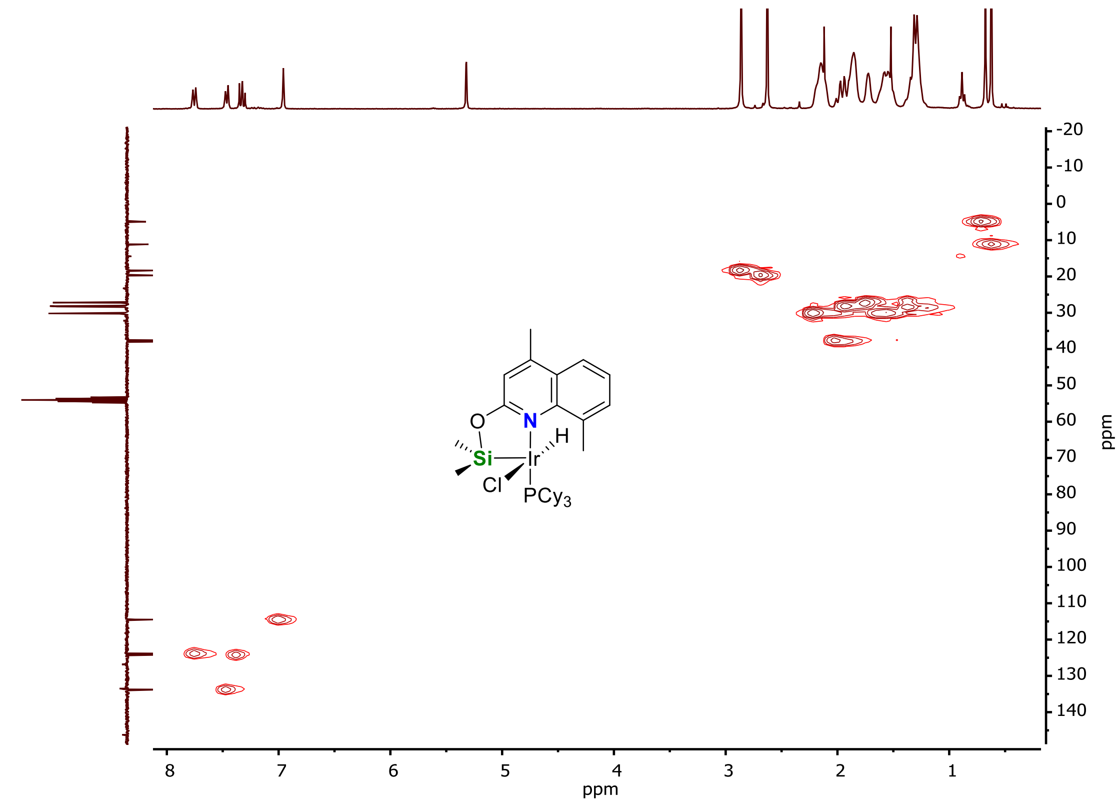

Figure S34. ^{1}H-^{13}C HSQC NMR spectrum of **4** in CD_2Cl_2 (298K).

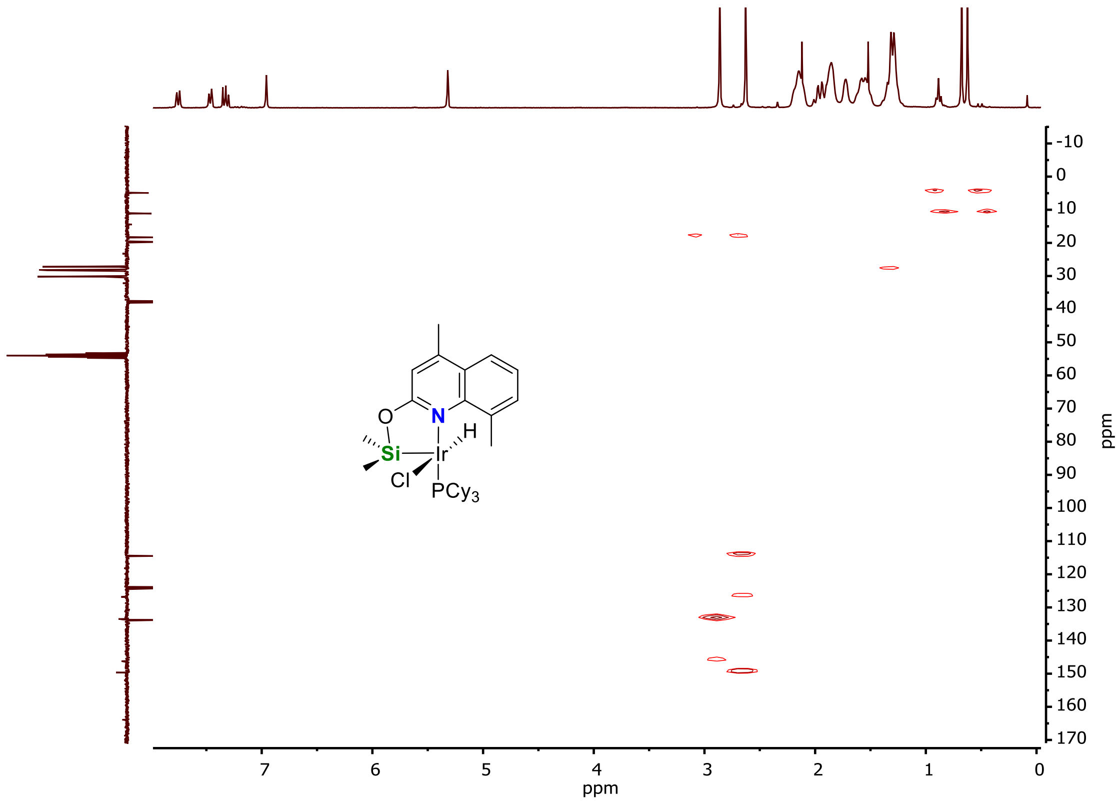

Figure S35. ^{1}H-^{13}C HMBC NMR spectrum of **4** in CD_2Cl_2 (298K).

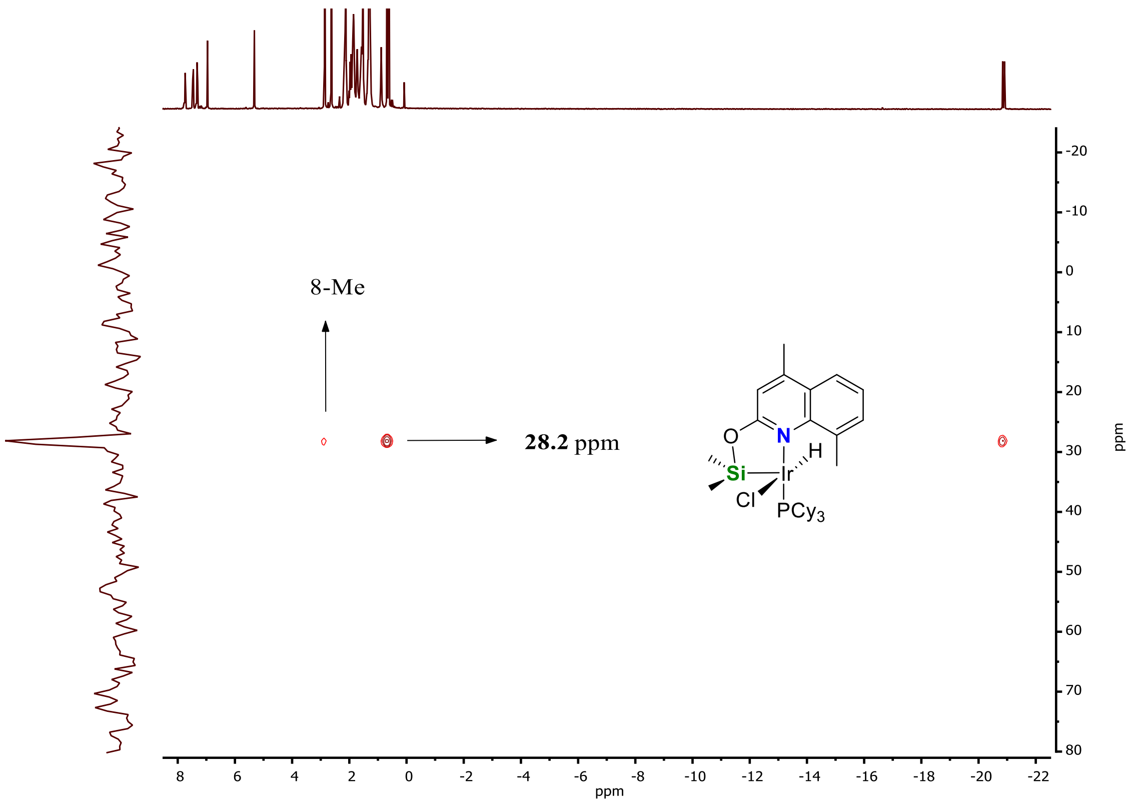

Figure S36. $^{1}H-^{29}Si$ HMBC NMR spectrum of **4** in CD_2Cl_2 (298K).

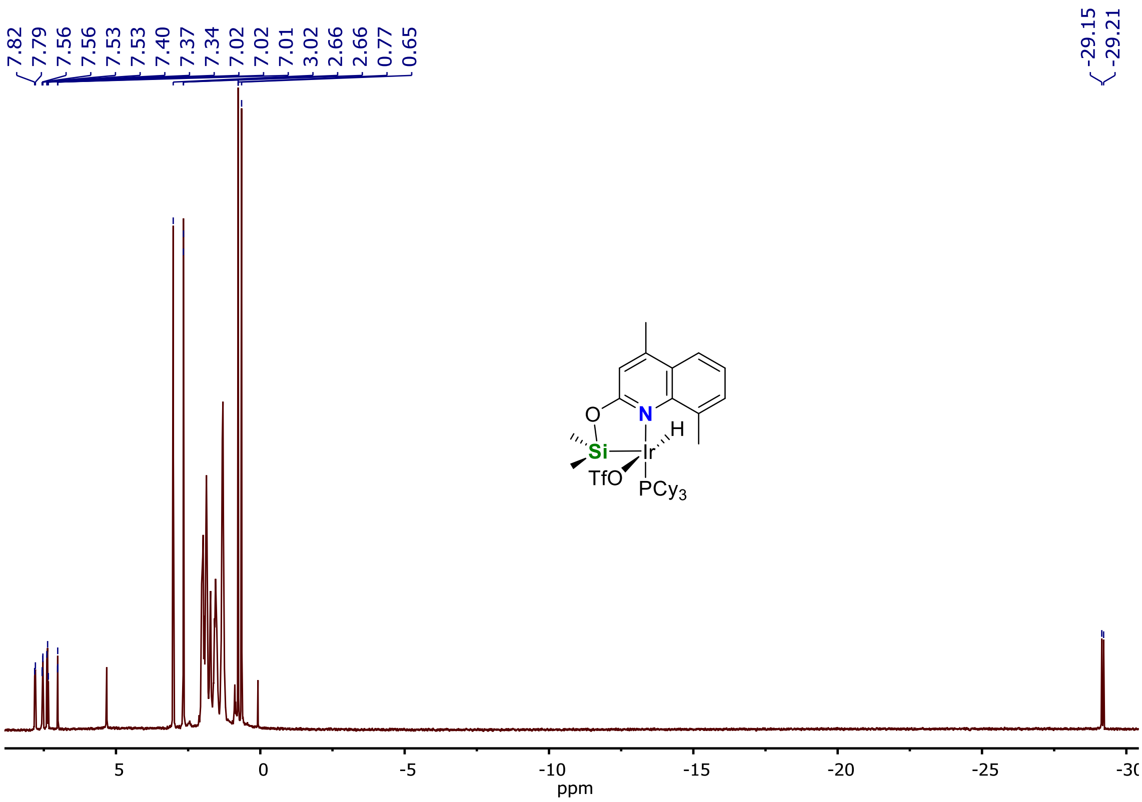

Figure S37. ^{1}H NMR spectrum of **5** in CD_2Cl_2 (300 MHz, 298K).

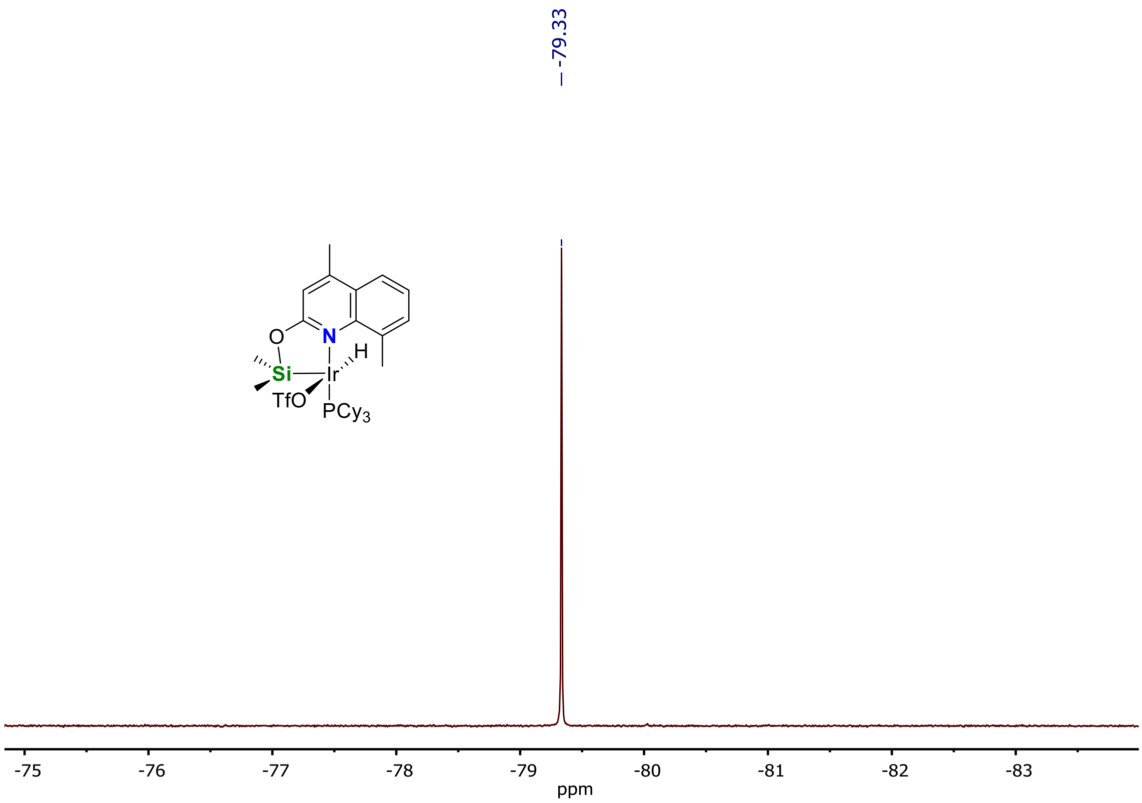

Figure S38. ^{19}F NMR spectrum of **5** in CD_2Cl_2 (282 MHz, 298K).

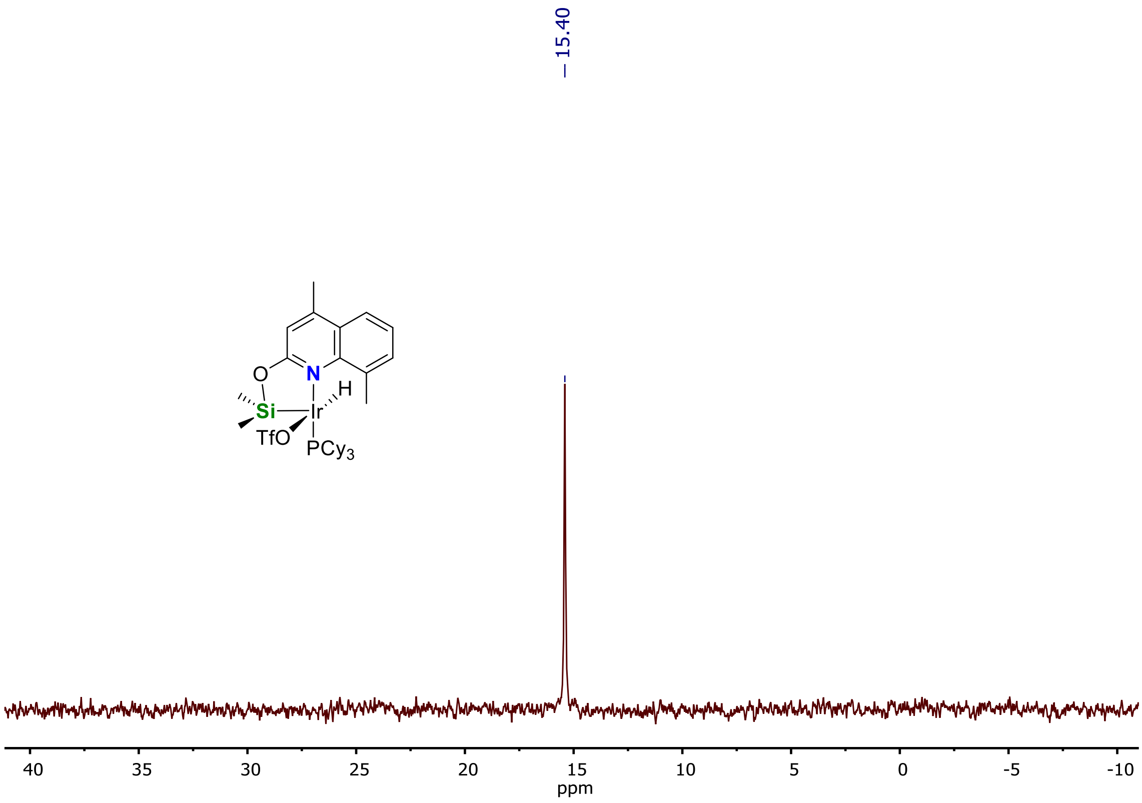

Figure S39. ^{31}P{^{1}H} NMR spectrum of **5** in CD_2Cl_2 (121 MHz, 298K).

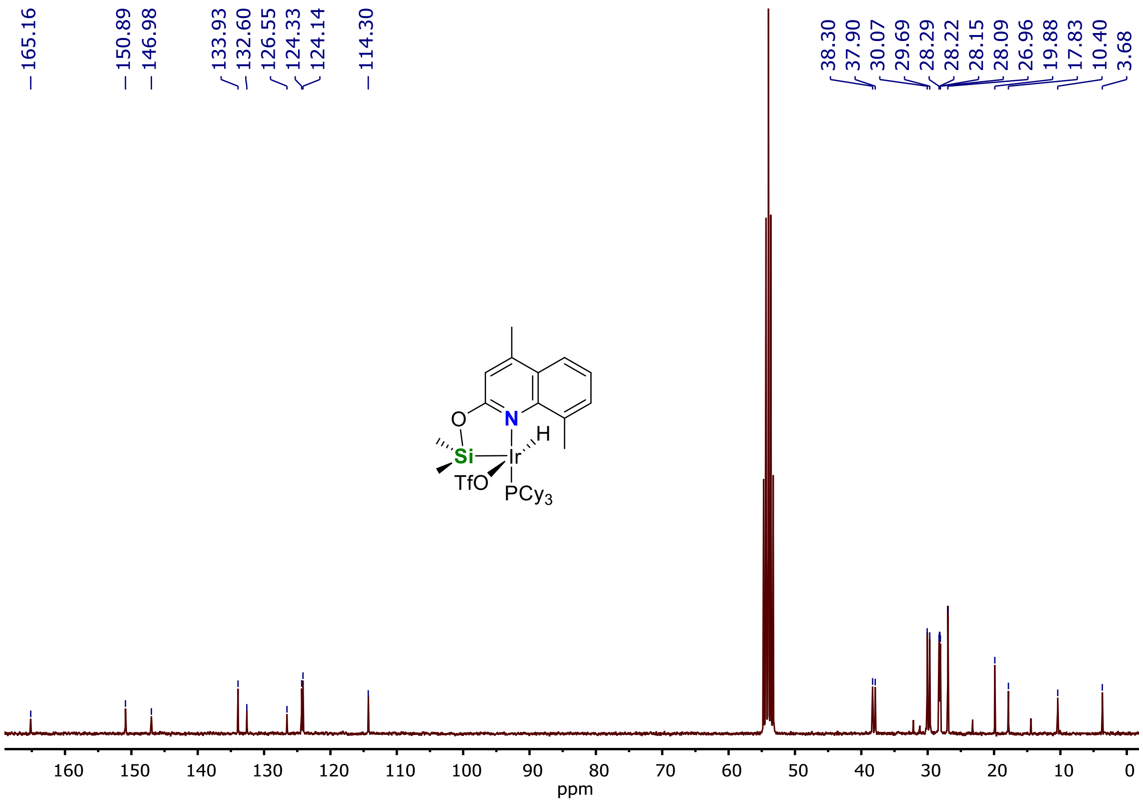

Figure S40. $^{13}C\{^{1}H\}$ NMR spectrum of **5** in CD_2Cl_2 (75 MHz, 298K).

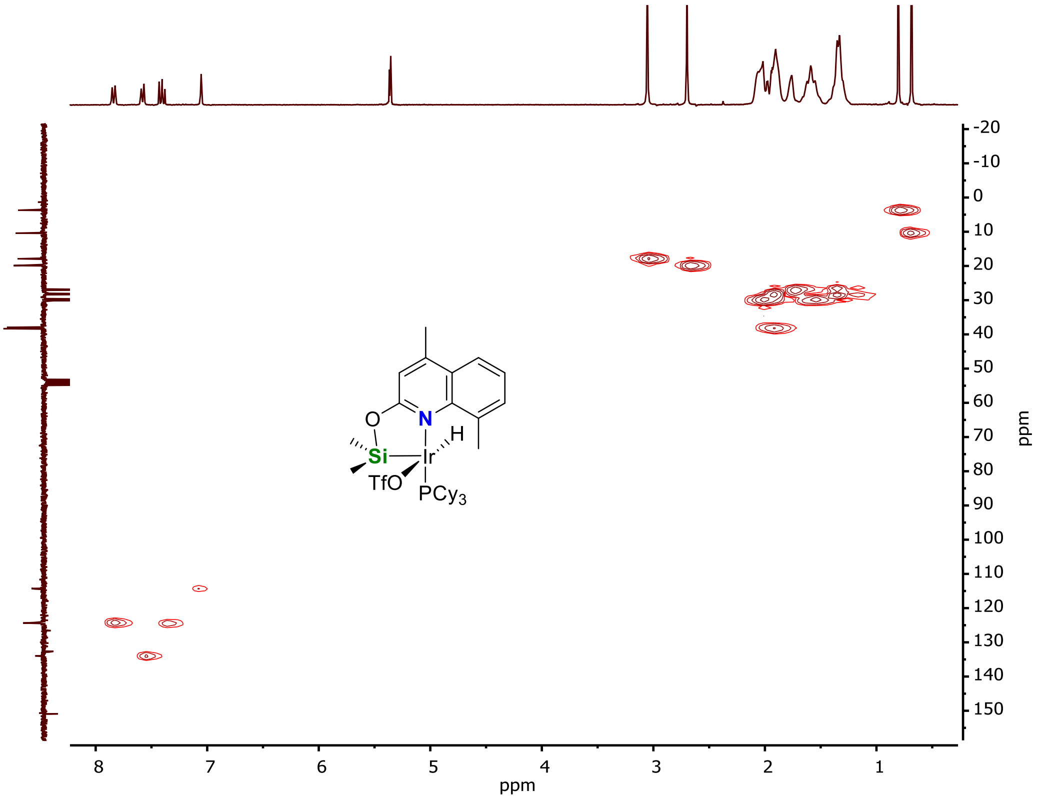

Figure S41. ^{1}H-^{13}C HSQC NMR spectrum of **5** in CD_2Cl_2 (298K).

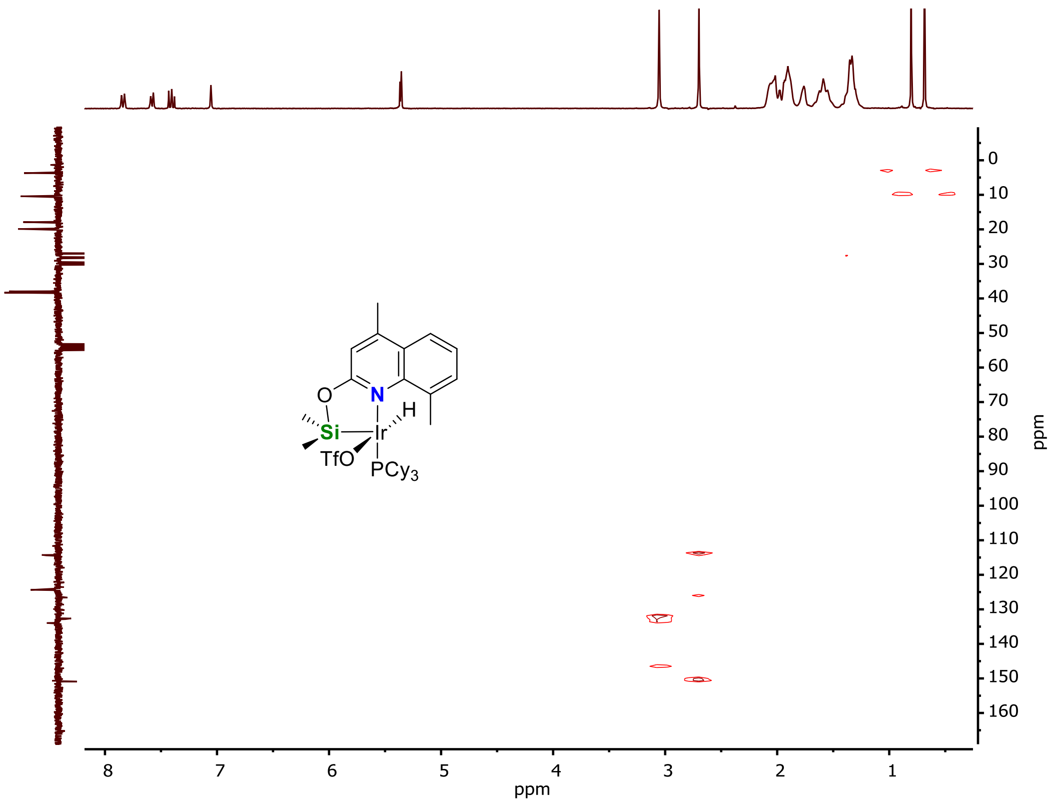

Figure S42. ^{1}H-^{13}C HMBC NMR spectrum of **5** in CD_2Cl_2 (298K).

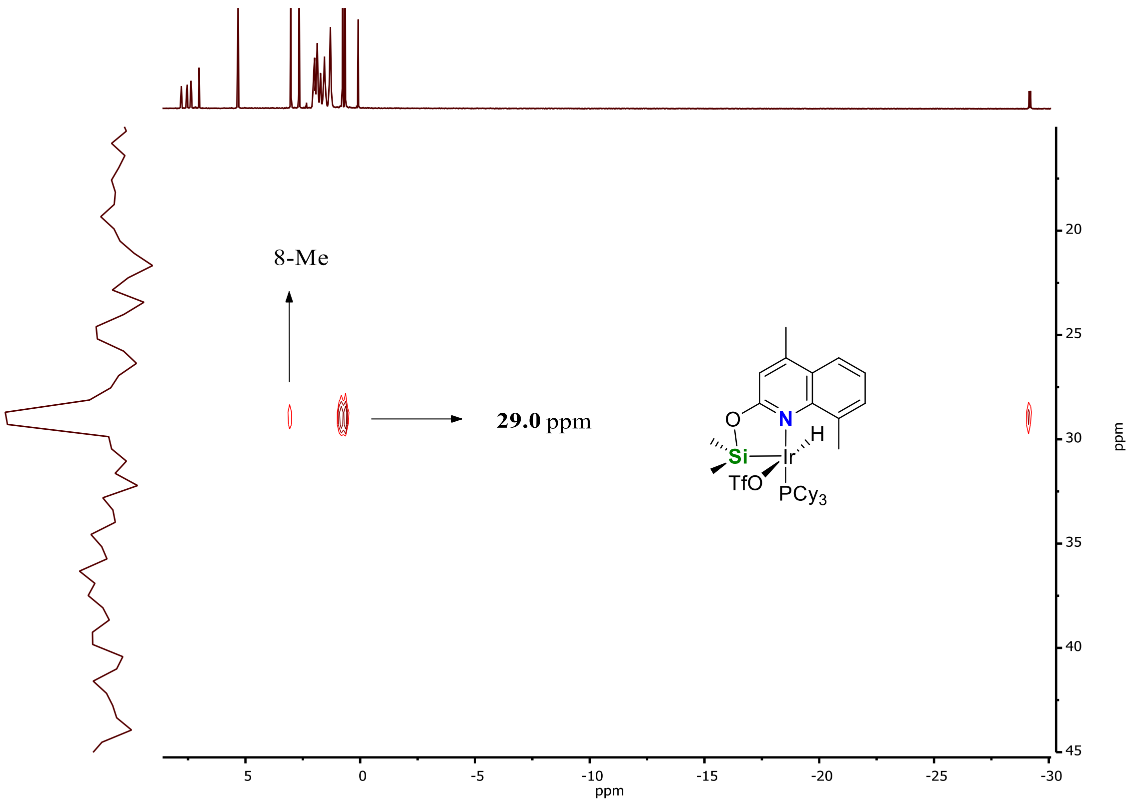

Figure S43. ^{1}H-^{29}Si HMBC NMR spectrum of **5** in CD_2Cl_2 (298K).

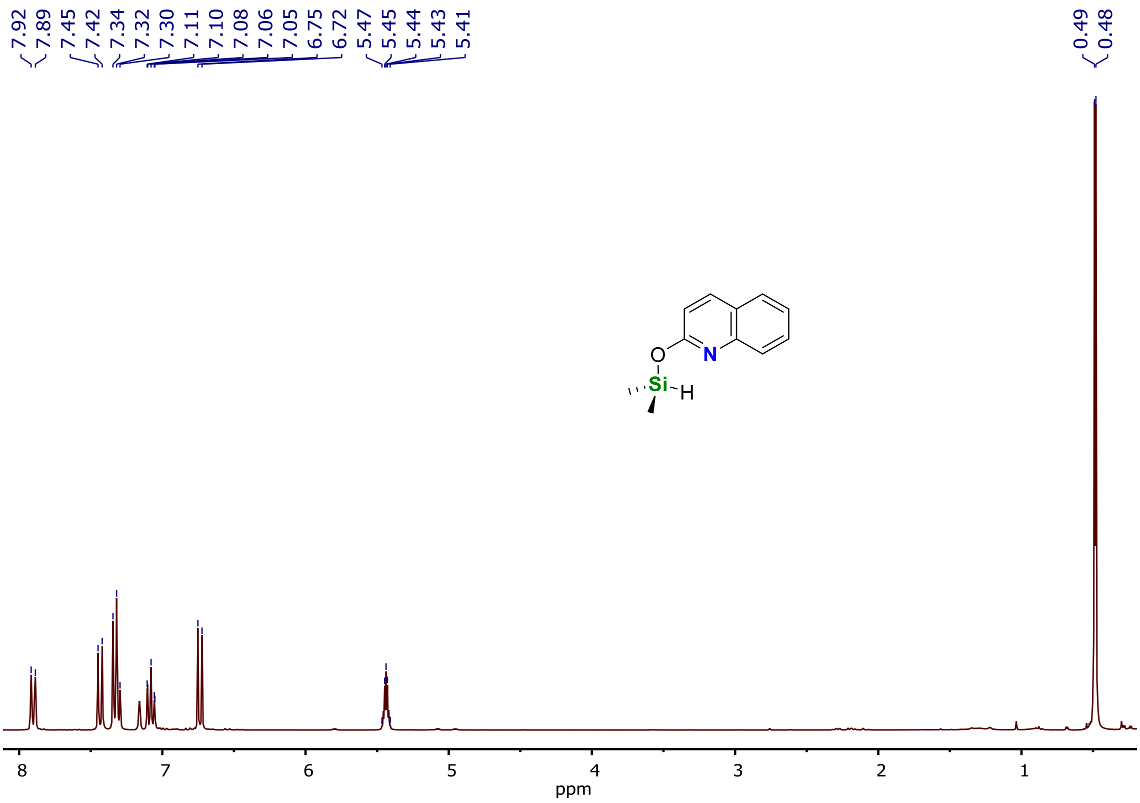

Figure S44. ^{1}H NMR spectrum of **8** in C_6D_6 (300 MHz, 298K).

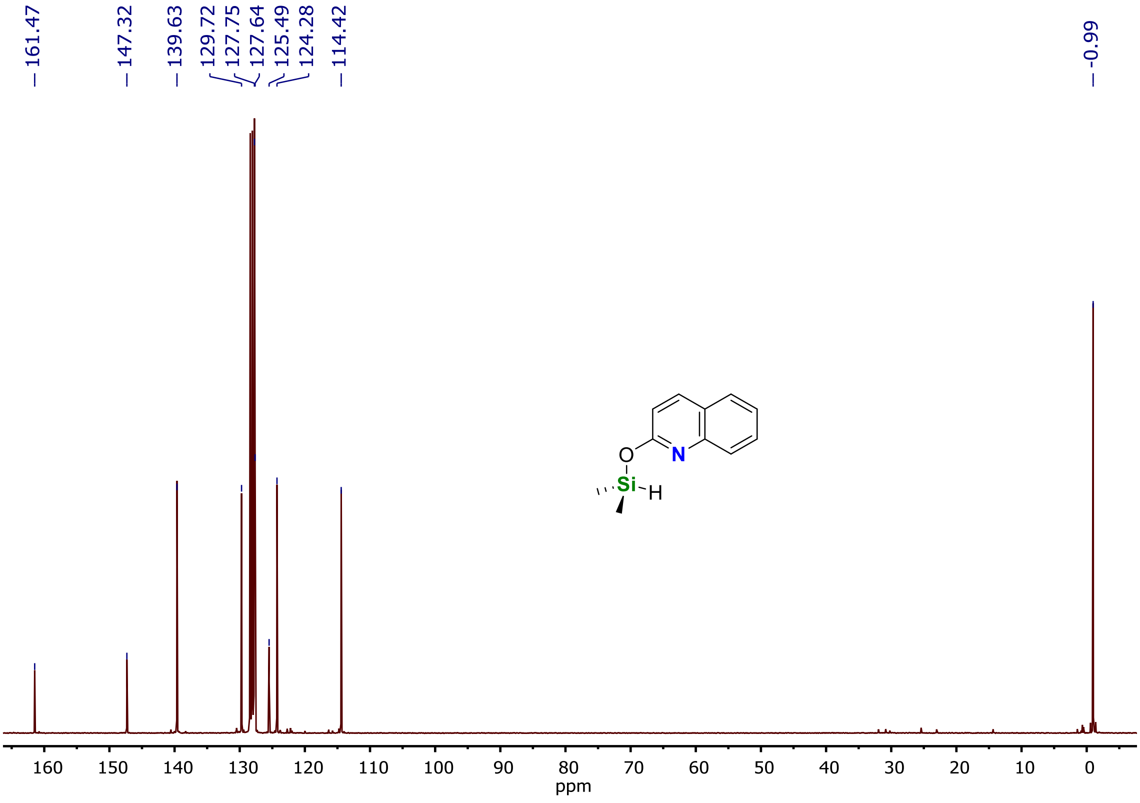

Figure S45. $^{13}C\{^{1}H\}$ NMR spectrum of **8** in C_6D_6 (75 MHz, 298K).

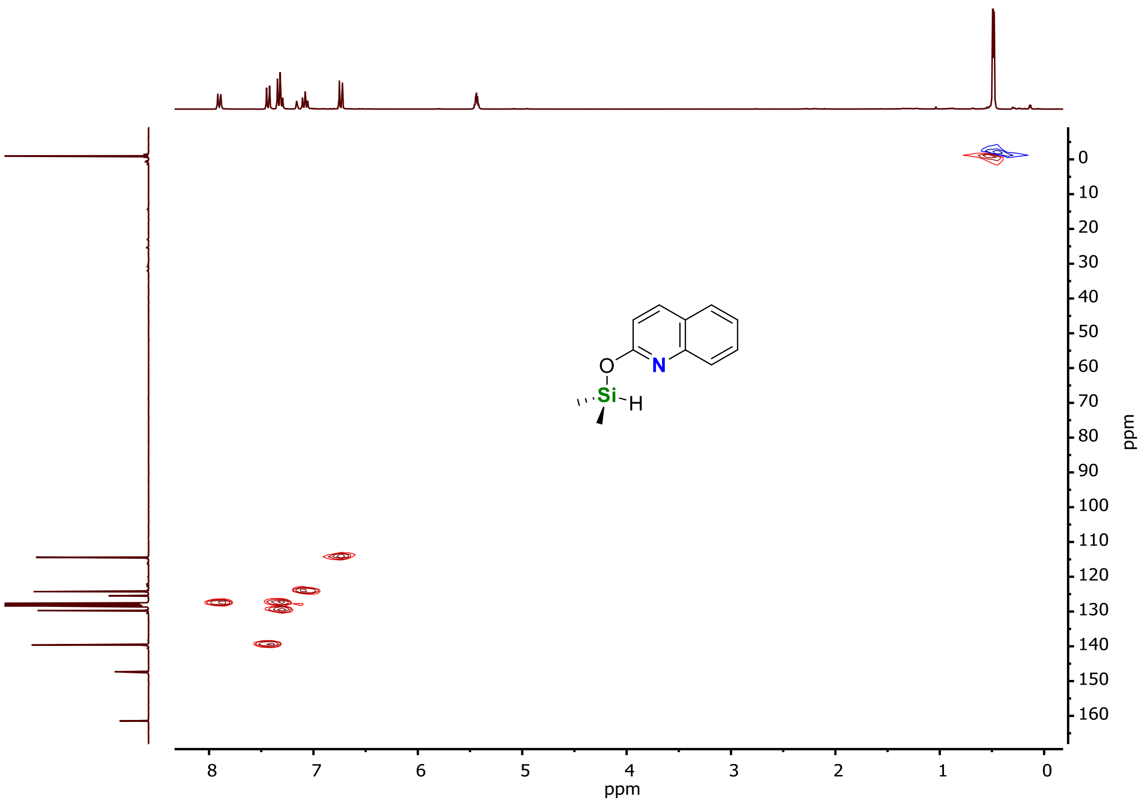

Figure S46. ^{1}H-^{13}C HSQC NMR spectrum of **8** in C_6D_6 (298K).

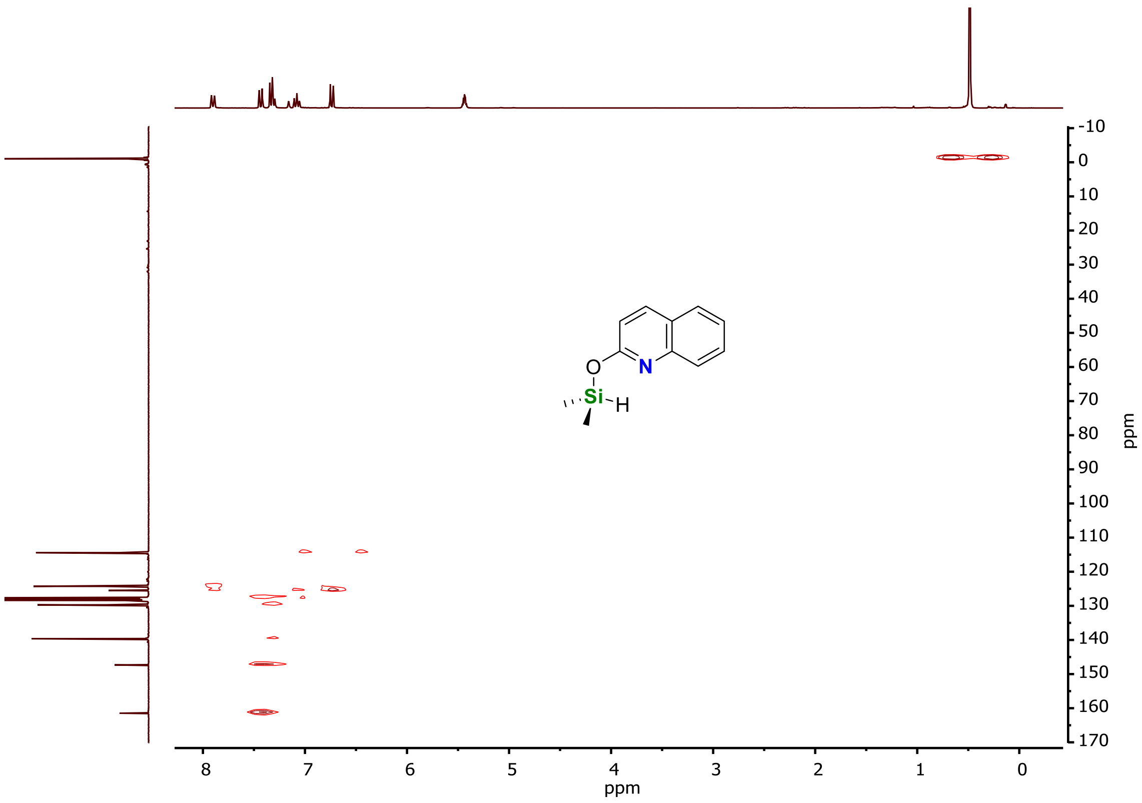

Figure S47. ^{1}H-^{13}C HMBC NMR spectrum of **8** in C_6D_6 (298K).

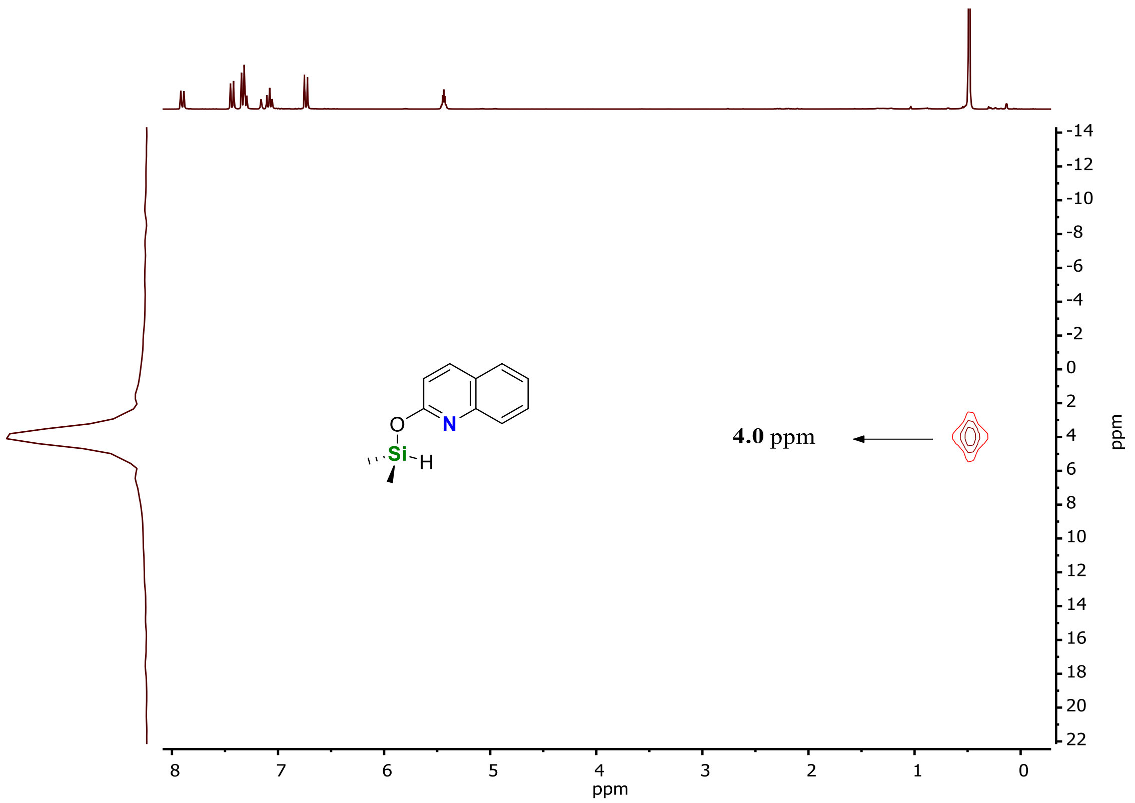

Figure S48. ^{1}H-^{29}Si HMQC NMR spectrum of **8** in C_6D_6 (298K).

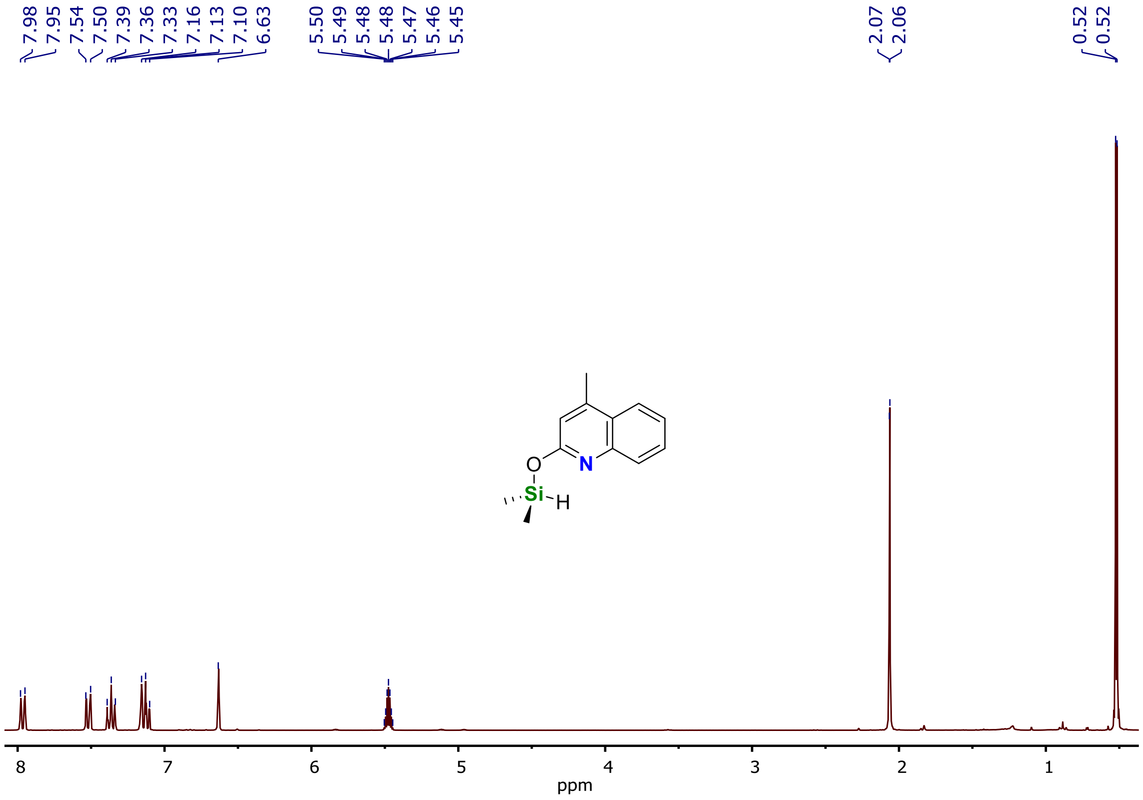

Figure S49. 1H NMR spectrum of **9** in C_6D_6 (300 MHz, 298K).

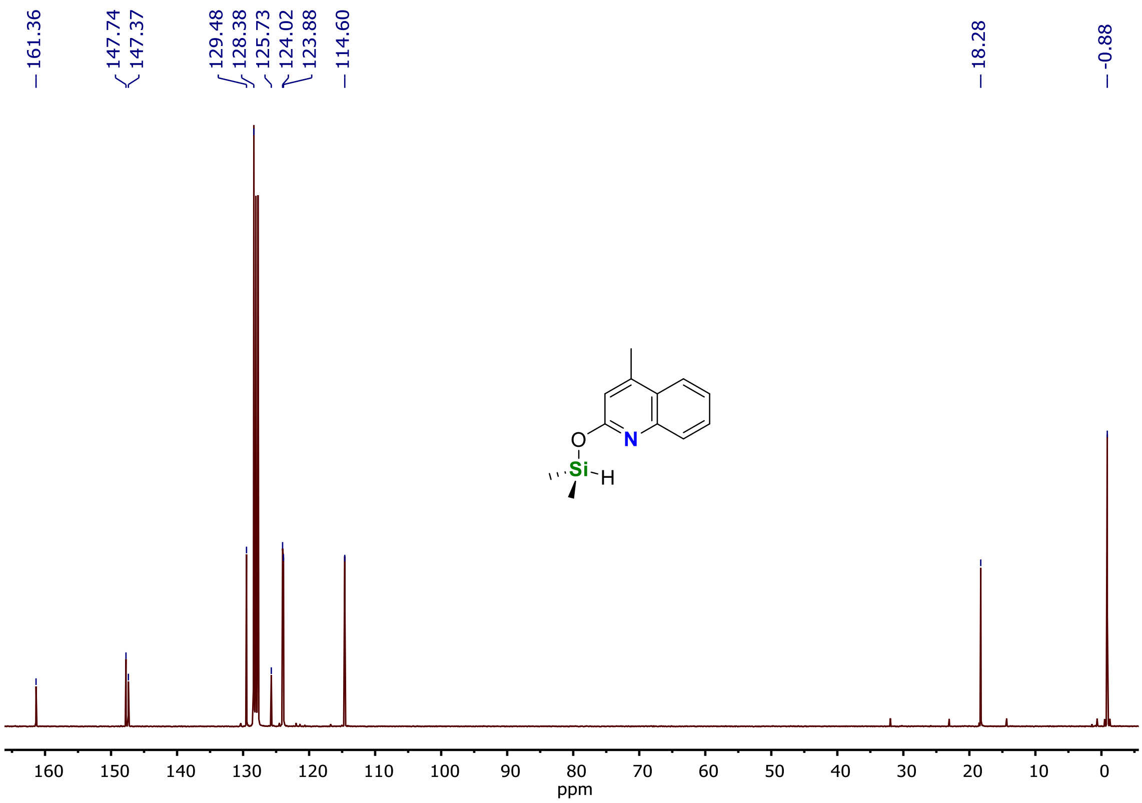

Figure S50. $^{13}C\{^{1}H\}$ NMR spectrum of **9** in C_6D_6 (75 MHz, 298K).

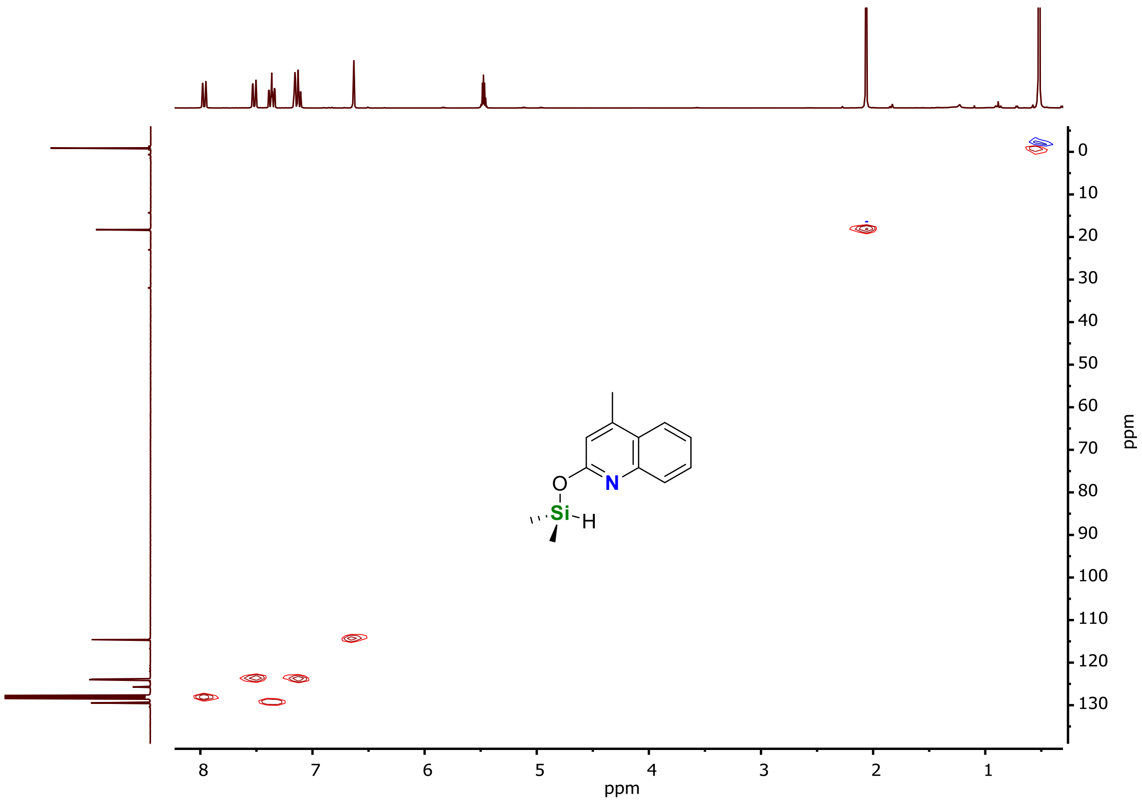

Figure S51. ^{1}H-^{13}C HSQC NMR spectrum of **9** in C_6D_6 (298K).

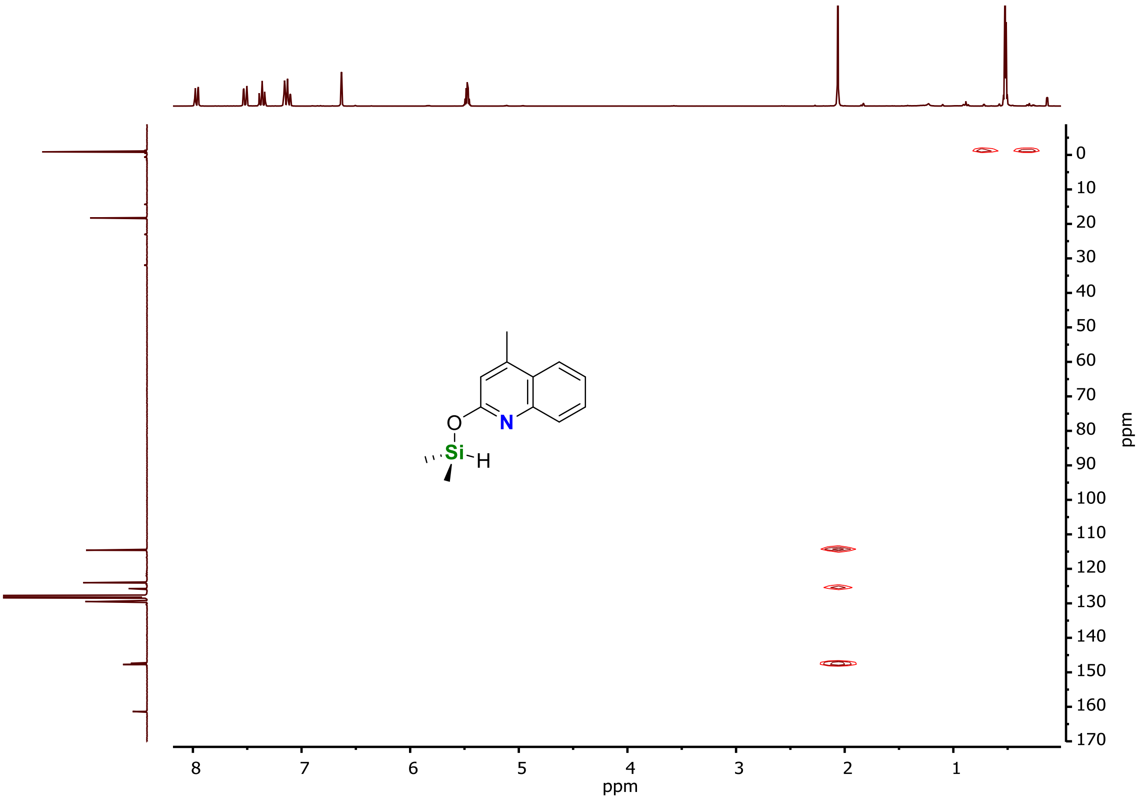

Figure S52. ^{1}H-^{13}C HMBC NMR spectrum of **9** in C_6D_6 (298K).

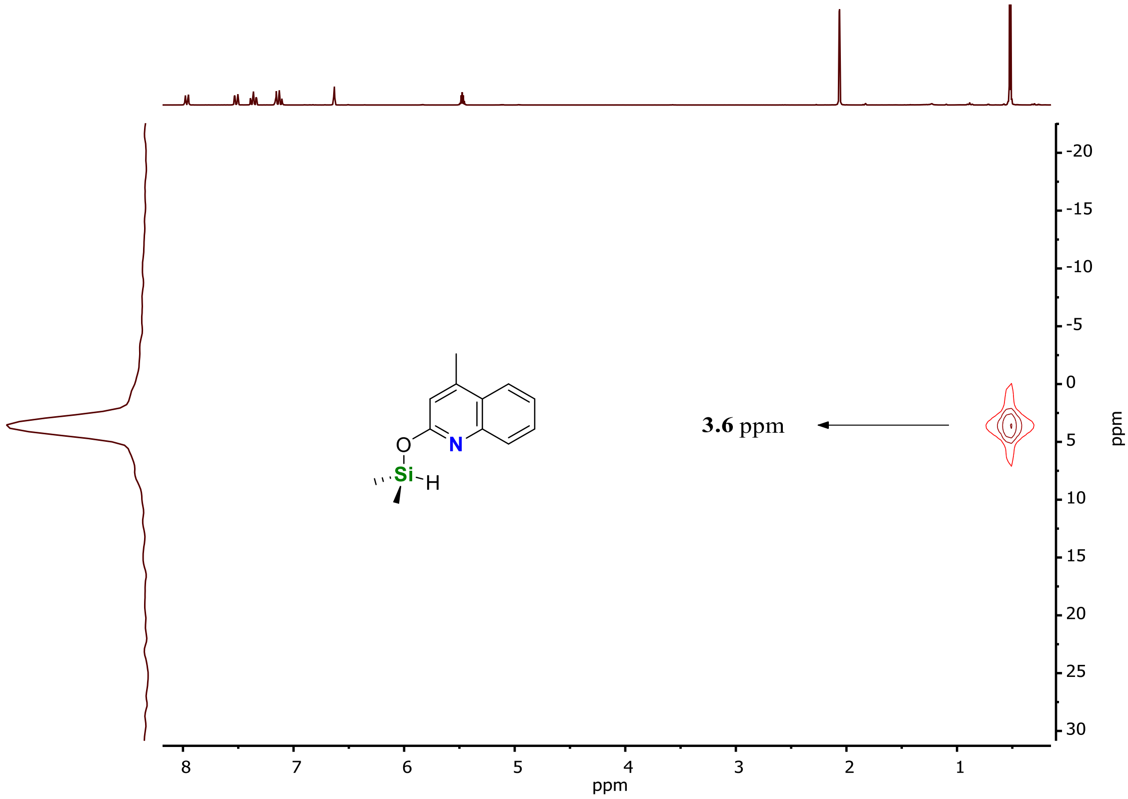

Figure S53. ^{1}H-^{29}Si HMQC NMR spectrum of **9** in C_6D_6 (298K).

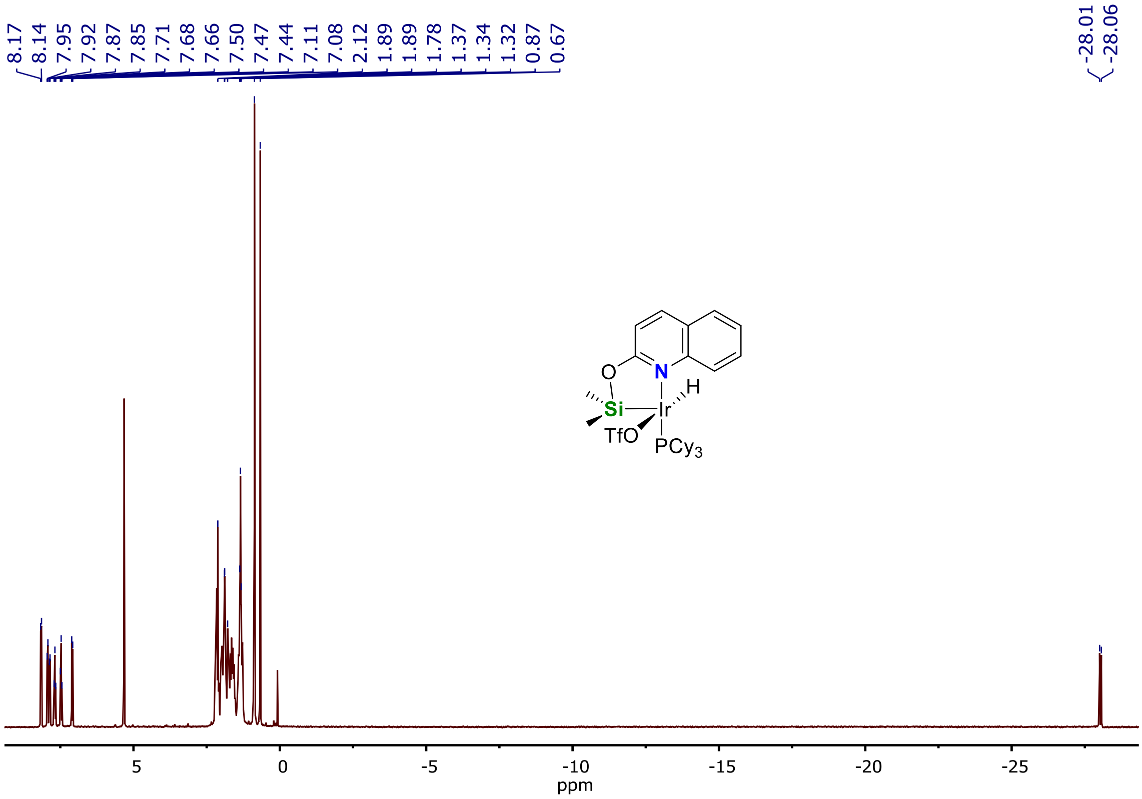

Figure S54. ^{1}H NMR spectrum of **10** in CD_2Cl_2 (300 MHz, 298K).

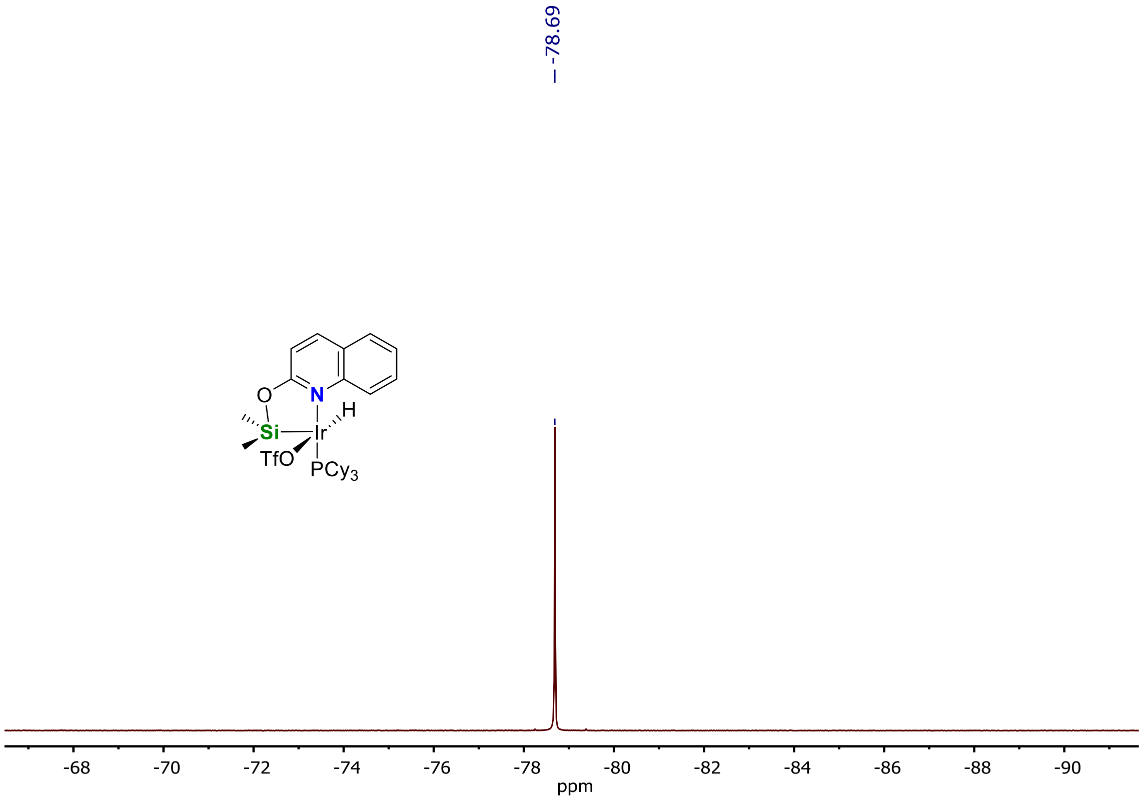

Figure S55. ^{19}F NMR spectrum of **10** in CD_2Cl_2 (282 MHz, 298K).

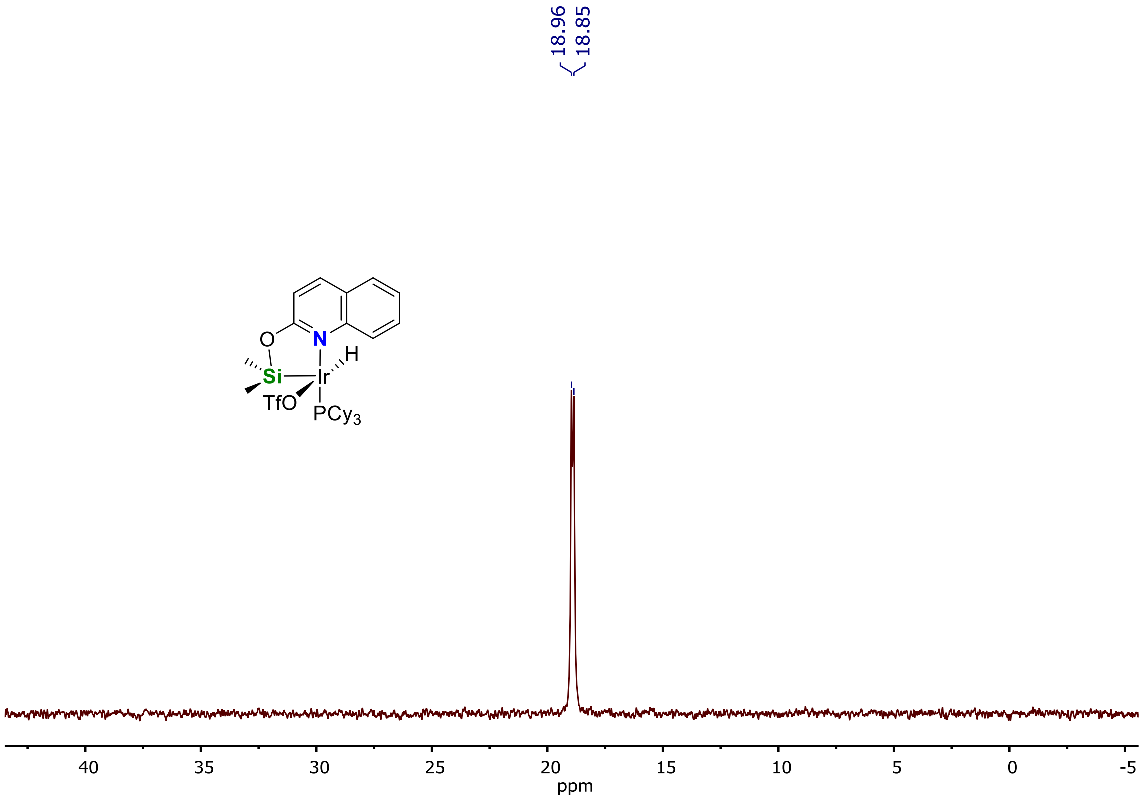

Figure S56. ^{31}P NMR spectrum of **10** in CD_2Cl_2 (121 MHz, 298K).

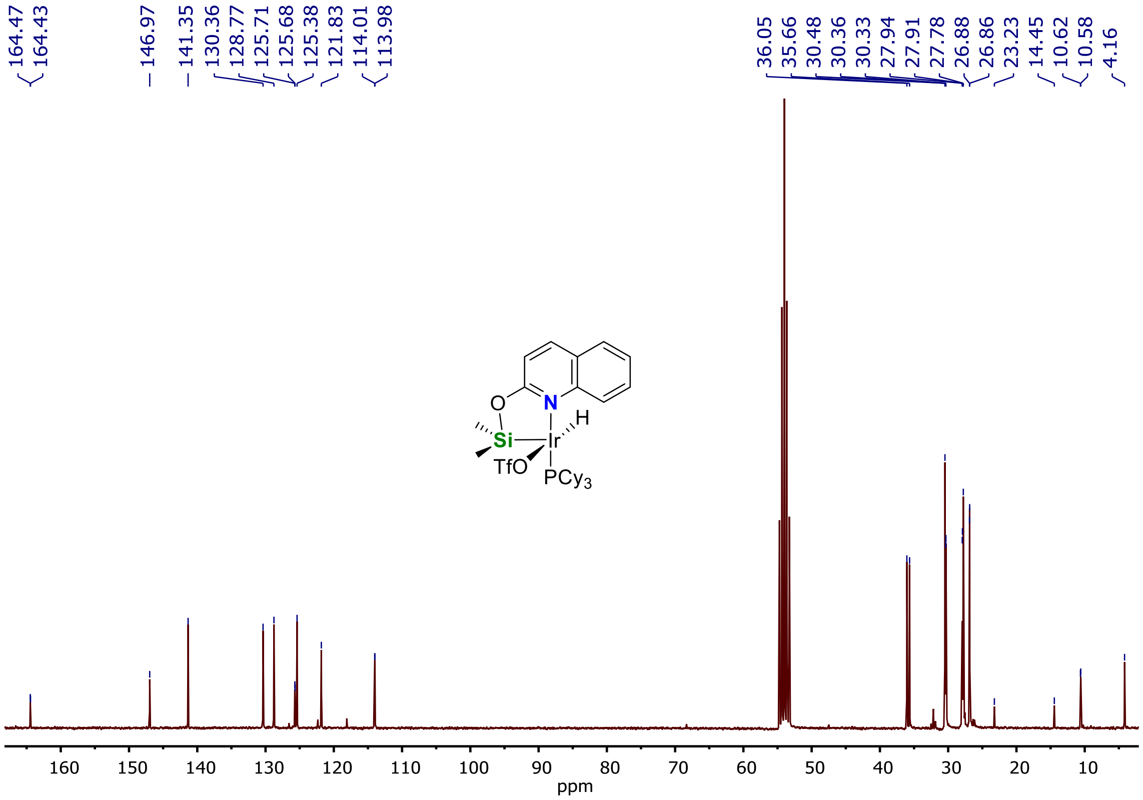

Figure S57. ^{13}C{^{1}H} NMR spectrum of **10** in CD_2Cl_2 (75 MHz, 298K).

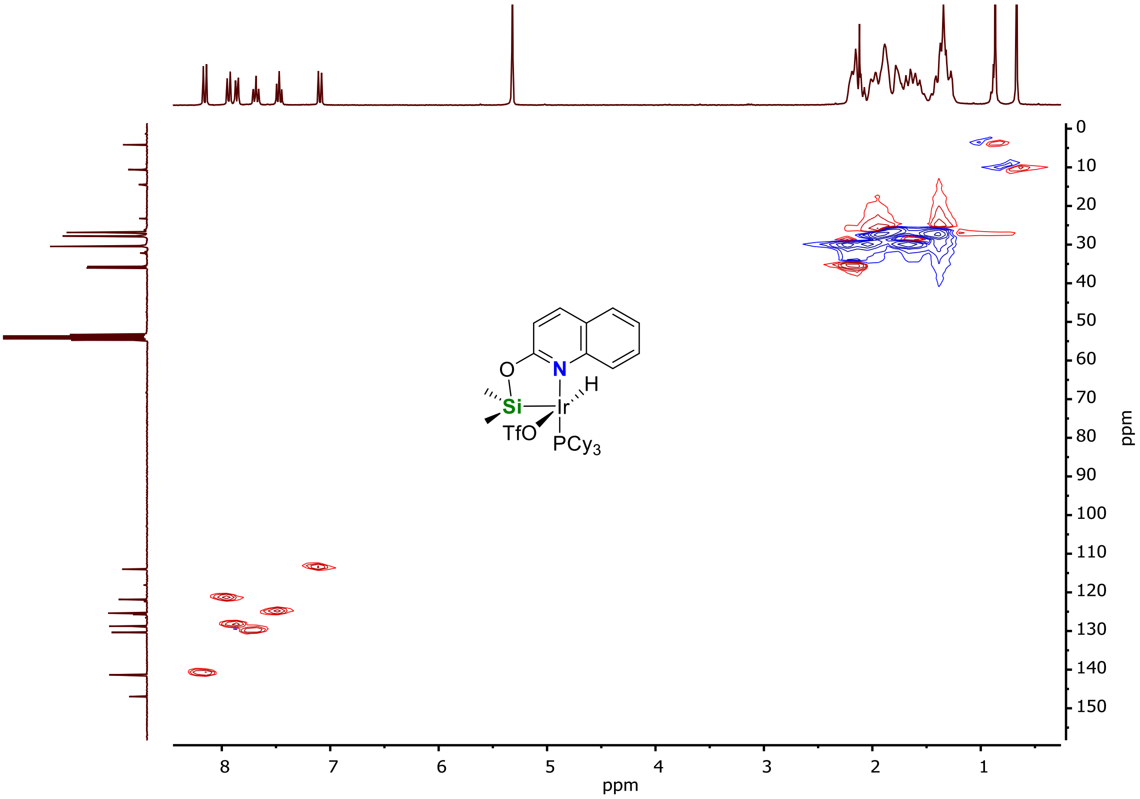

Figure S58. ^{1}H-^{13}C HSQC NMR spectrum of **10** in CD_2Cl_2 (298K).

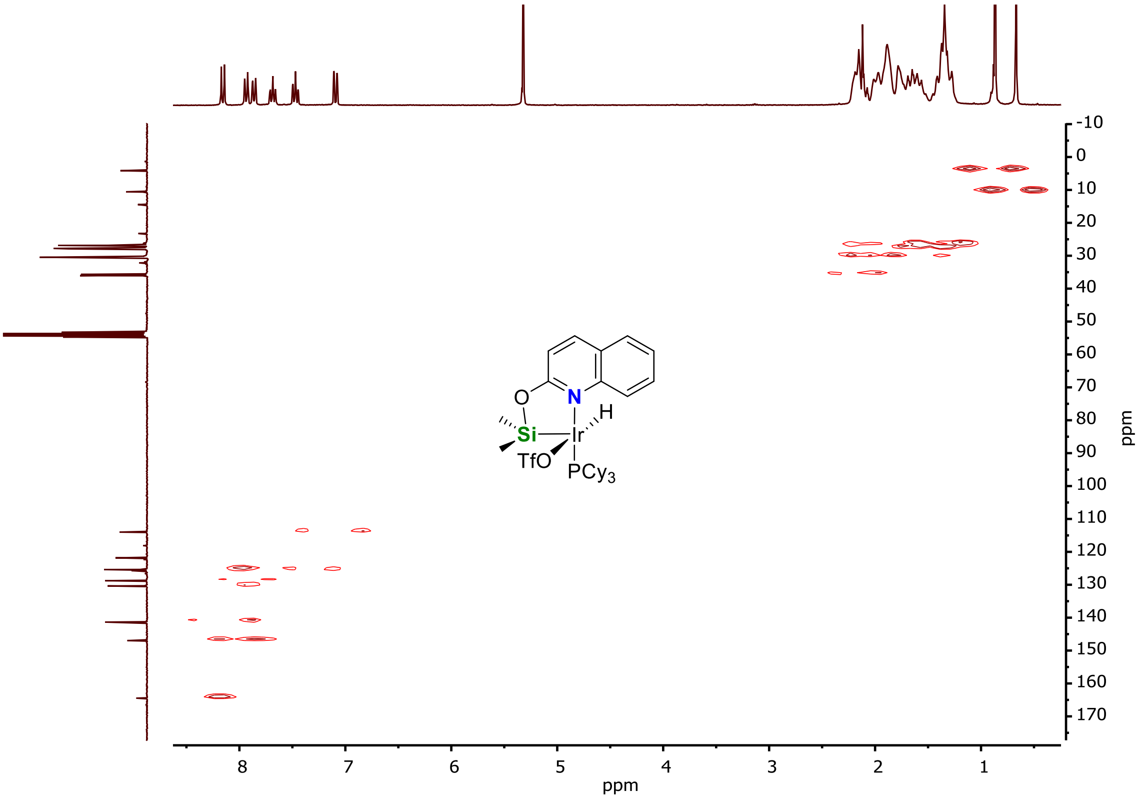

Figure S59. ^{1}H-^{13}C HMBC NMR spectrum of **10** in CD_2Cl_2 (298K).

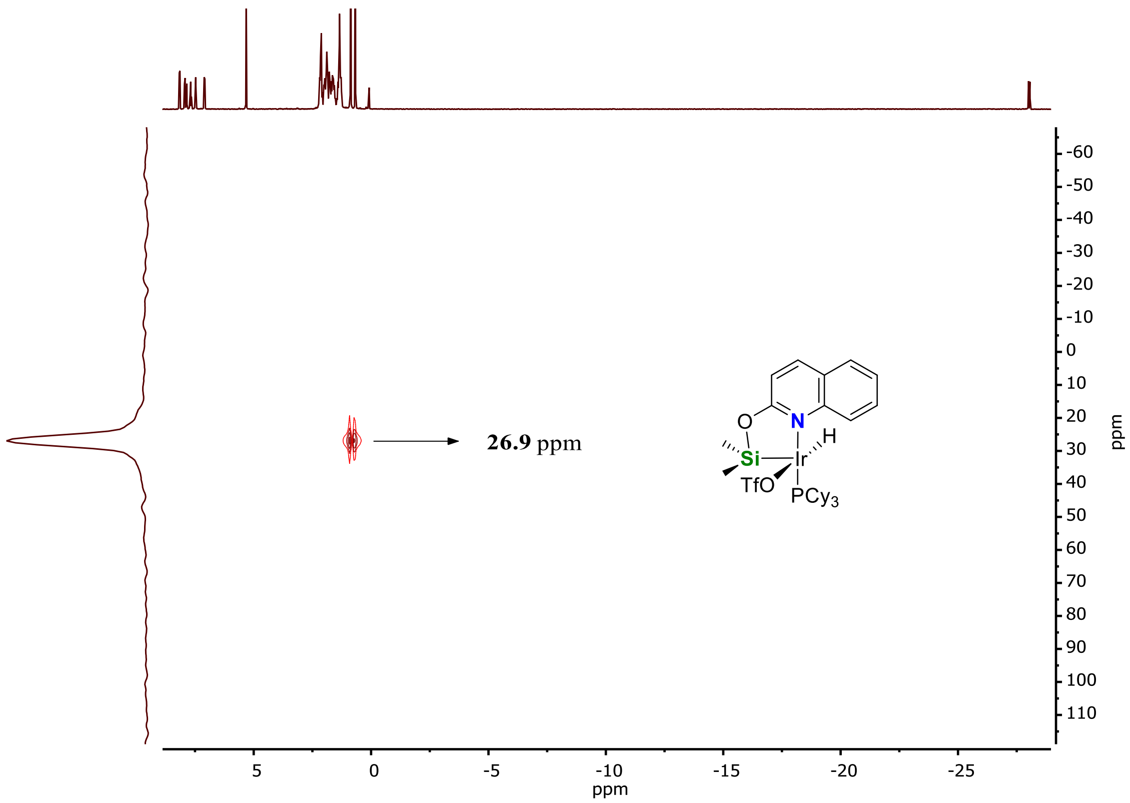

Figure S60. ^{1}H-^{29}Si HMBC NMR spectrum of **10** in CD_2Cl_2 (298K).

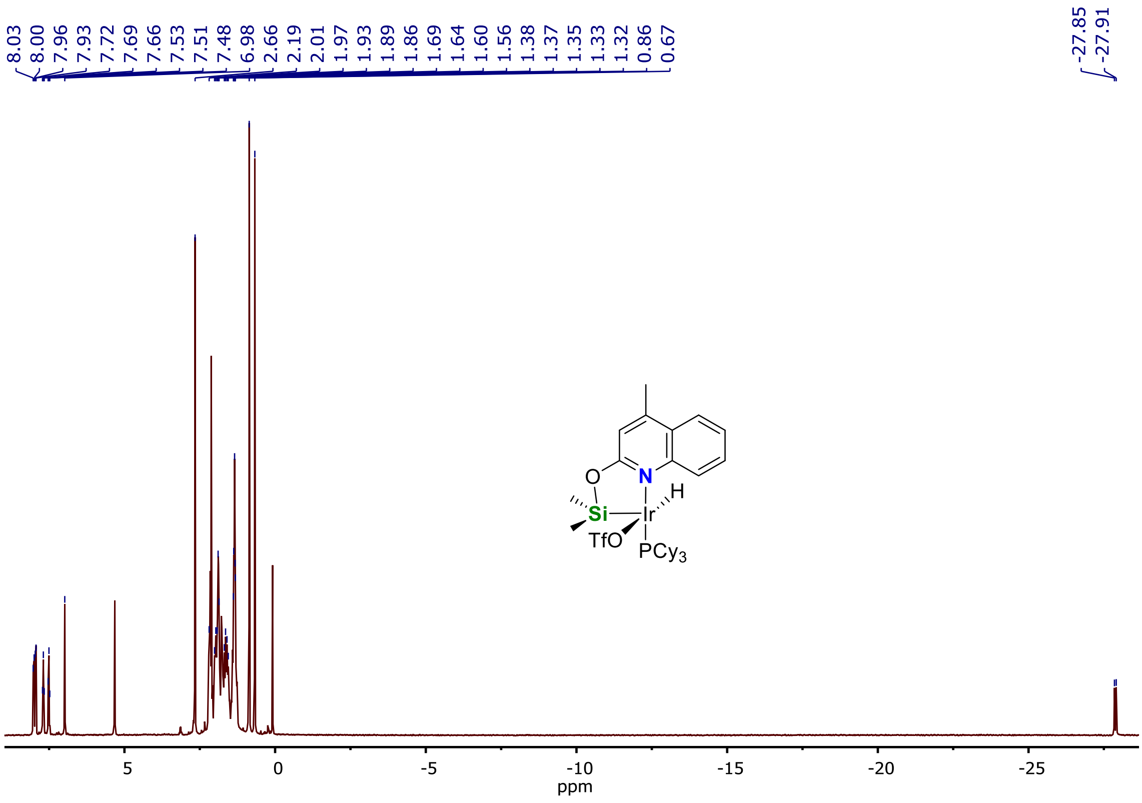

Figure S61. ^{1}H NMR spectrum of **11** in CD_2Cl_2 (300 MHz, 298K).

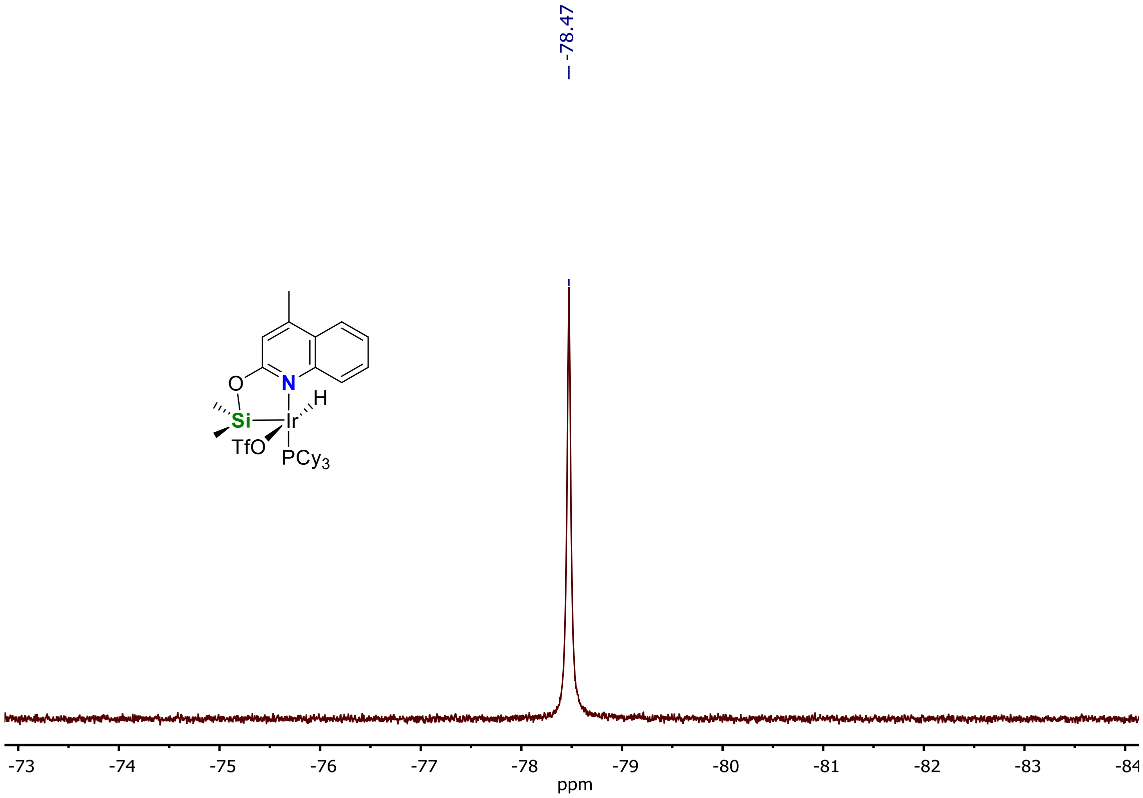

Figure S62. ^{19}F NMR spectrum of **11** in CD_2Cl_2 (282 MHz, 298K).

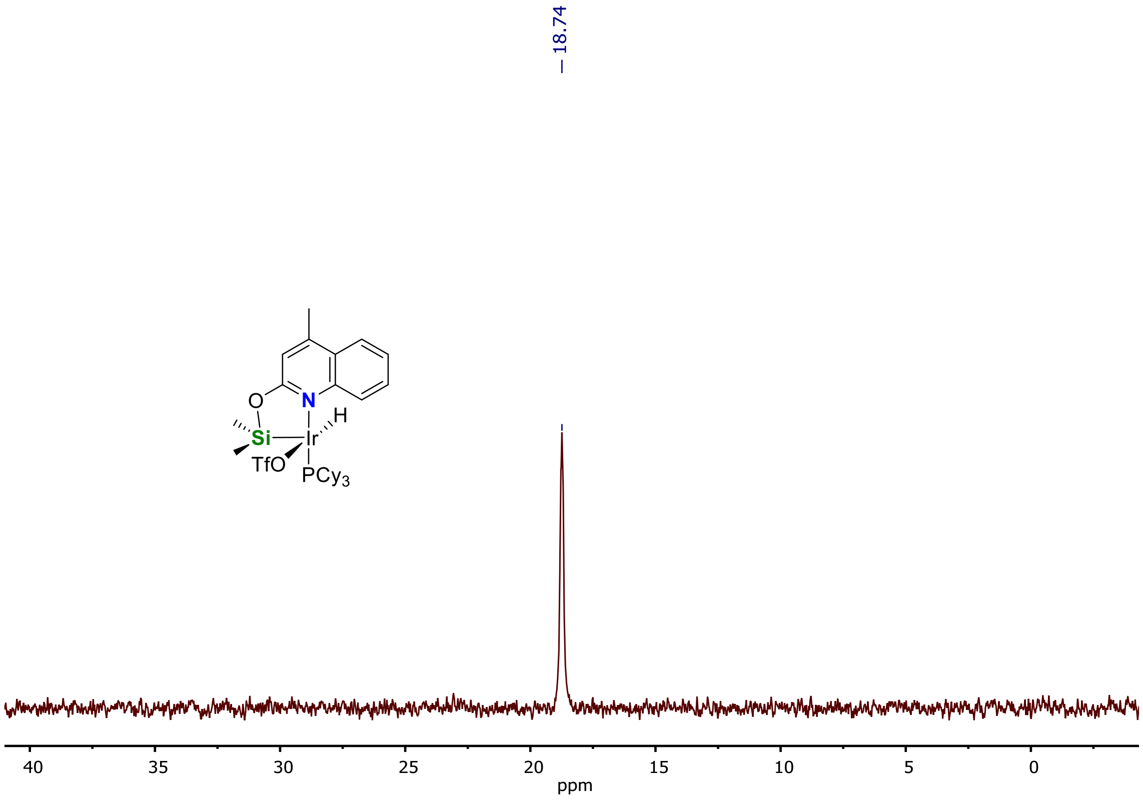

Figure S63. ^{31}P{^{1}H} NMR spectrum of **11** in CD_2Cl_2 (121 MHz, 298K).

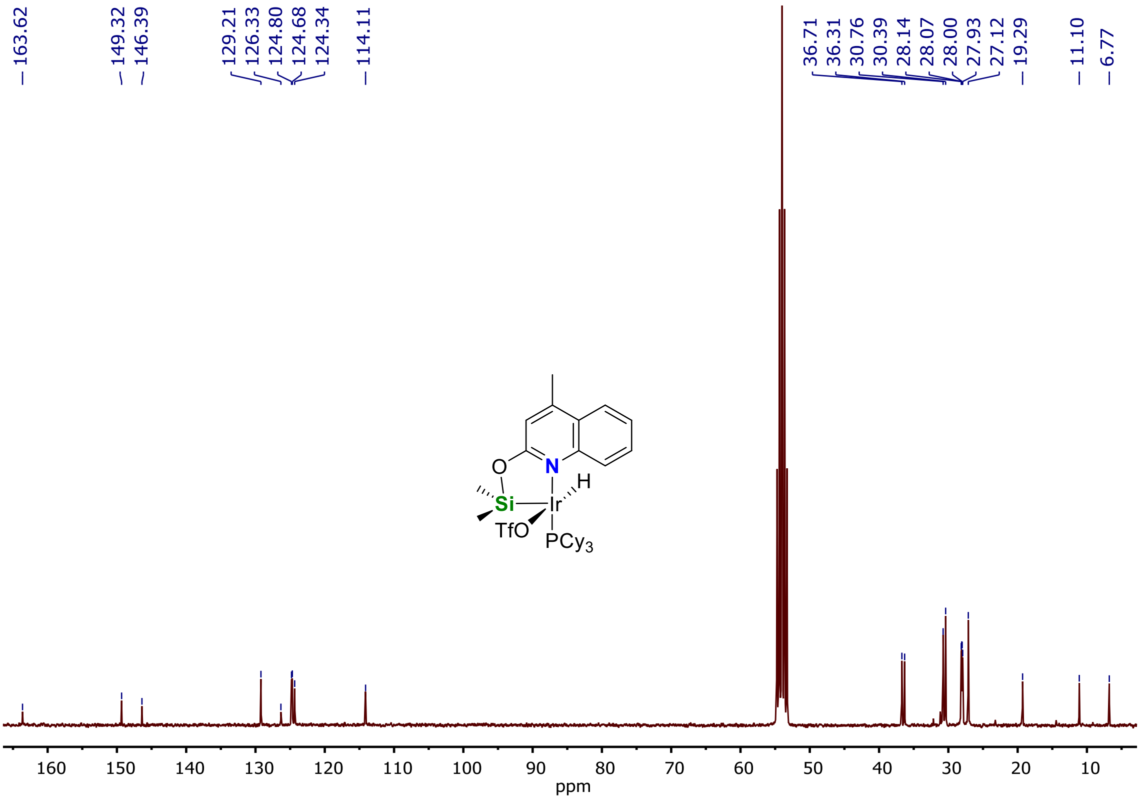

Figure S64. $^{13}C\{^{1}H\}$ NMR spectrum of **11** in CD_2Cl_2 (75 MHz, 298K).

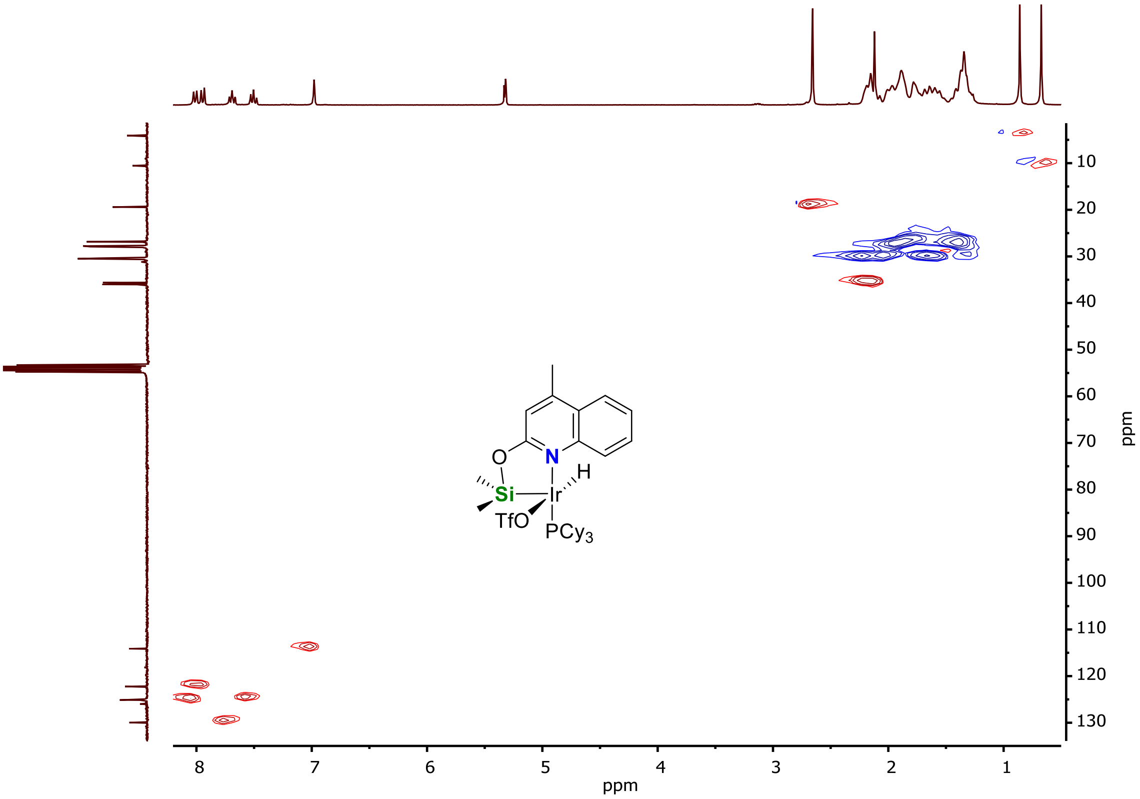

Figure S65. ^{1}H-^{13}C HSQC NMR spectrum of **11** in CD_2Cl_2 (298K).

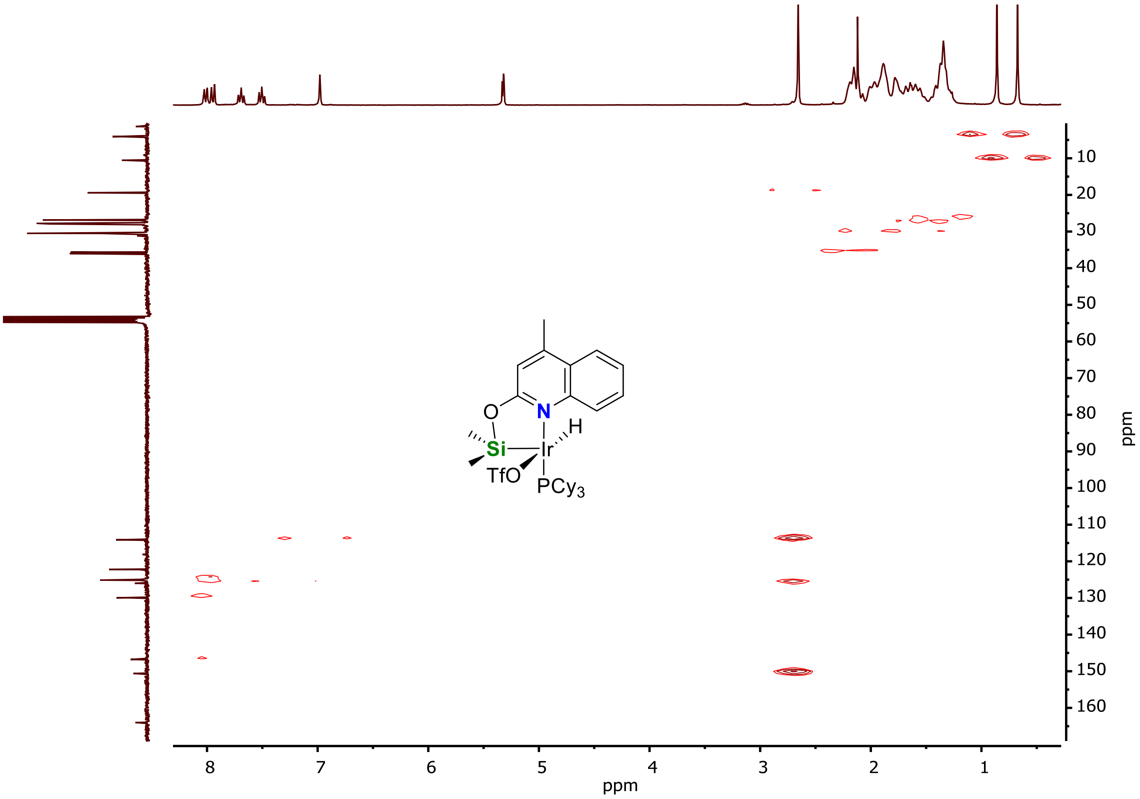

Figure S66. ^{1}H-^{13}C HMBC NMR spectrum of **11** in CD_2Cl_2 (298K).

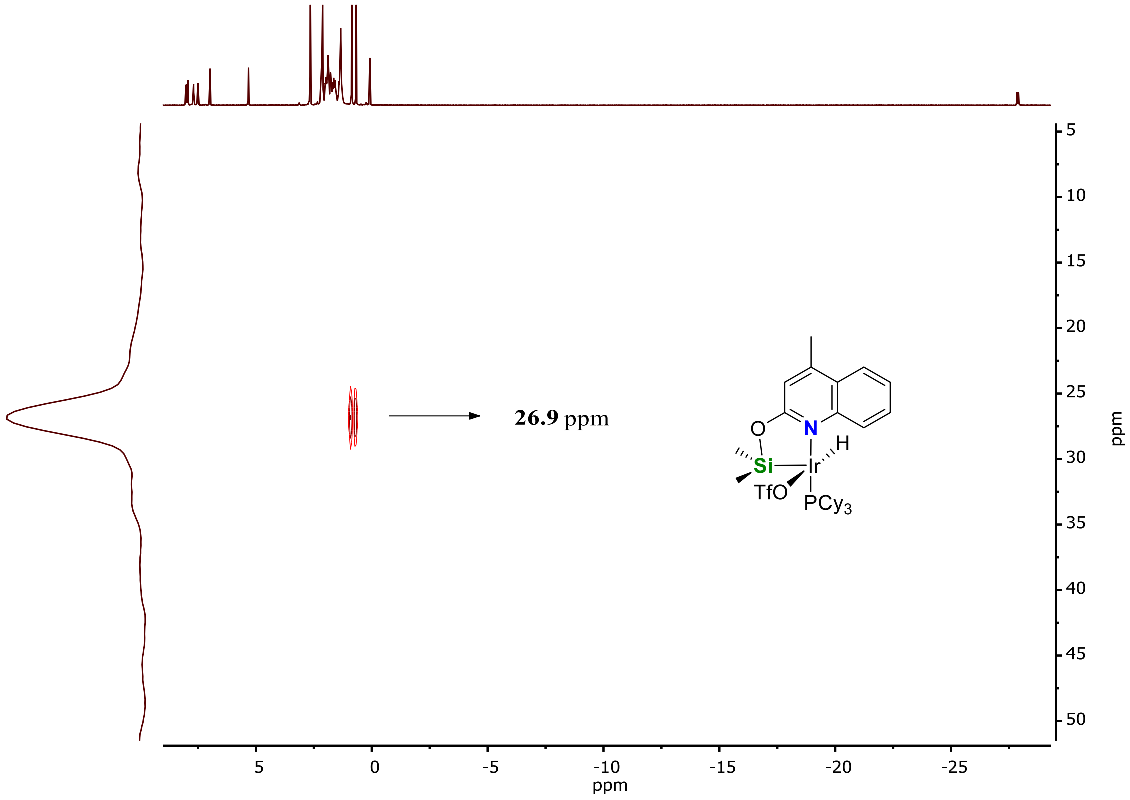

Figure S67. ^{1}H-^{29}Si HMBC NMR spectrum of **11** in CD_2Cl_2 (298K).

11.2. ^{13}C NMR spectra of **1**, **2**, **3**, **4** and **5**

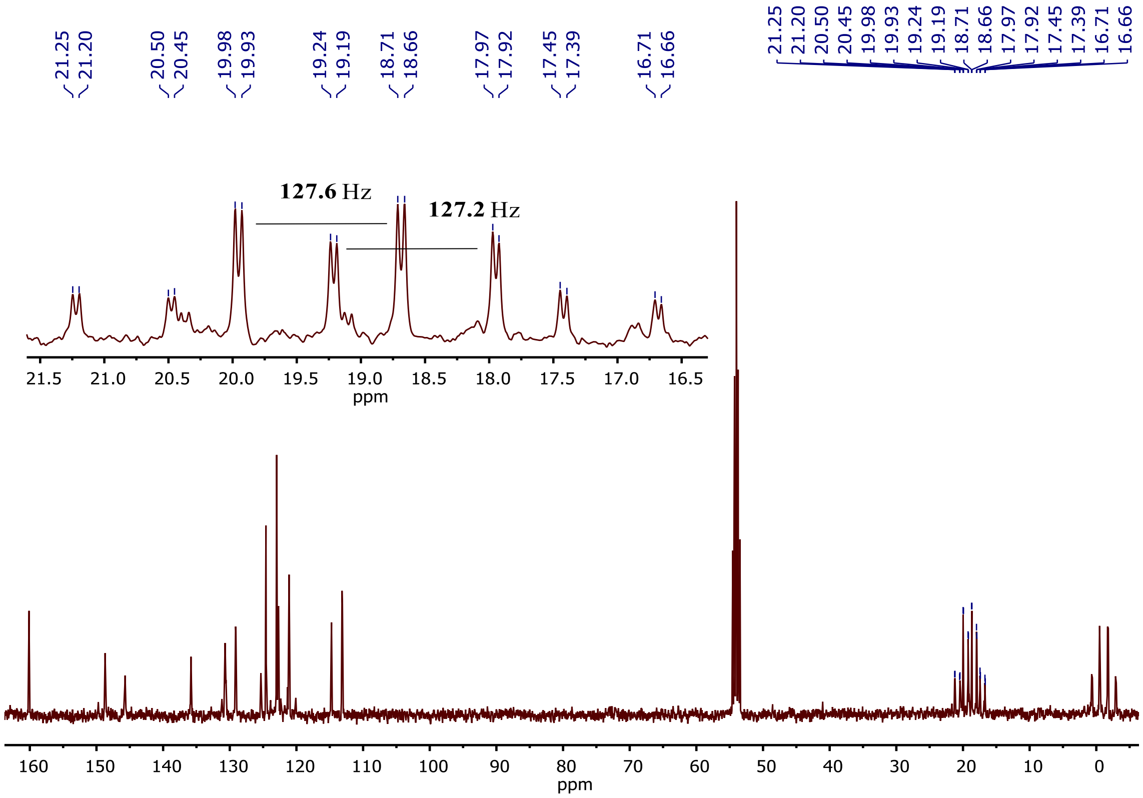

Figure S68. ^{13}C NMR spectrum of **1** in CD_2Cl_2 (101 MHz, 273K).

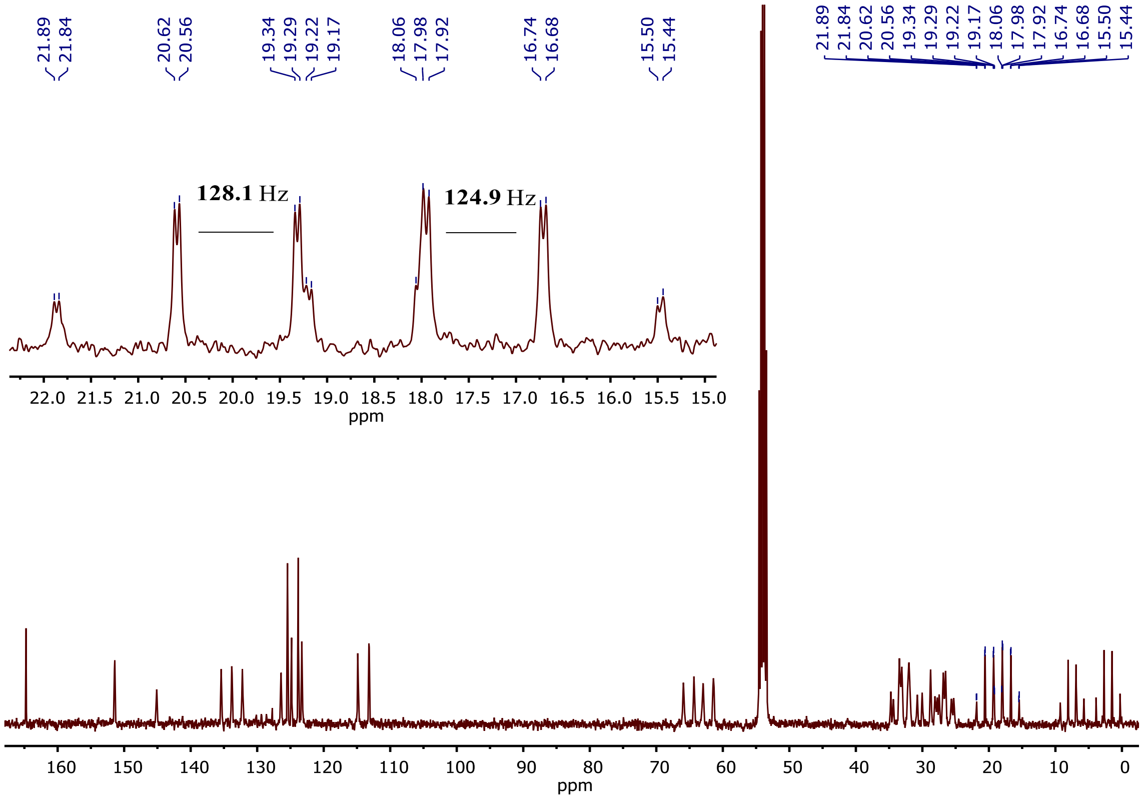

Figure S69. ^{13}C NMR spectrum of **2** in CD_2Cl_2 (101 MHz, 273K).

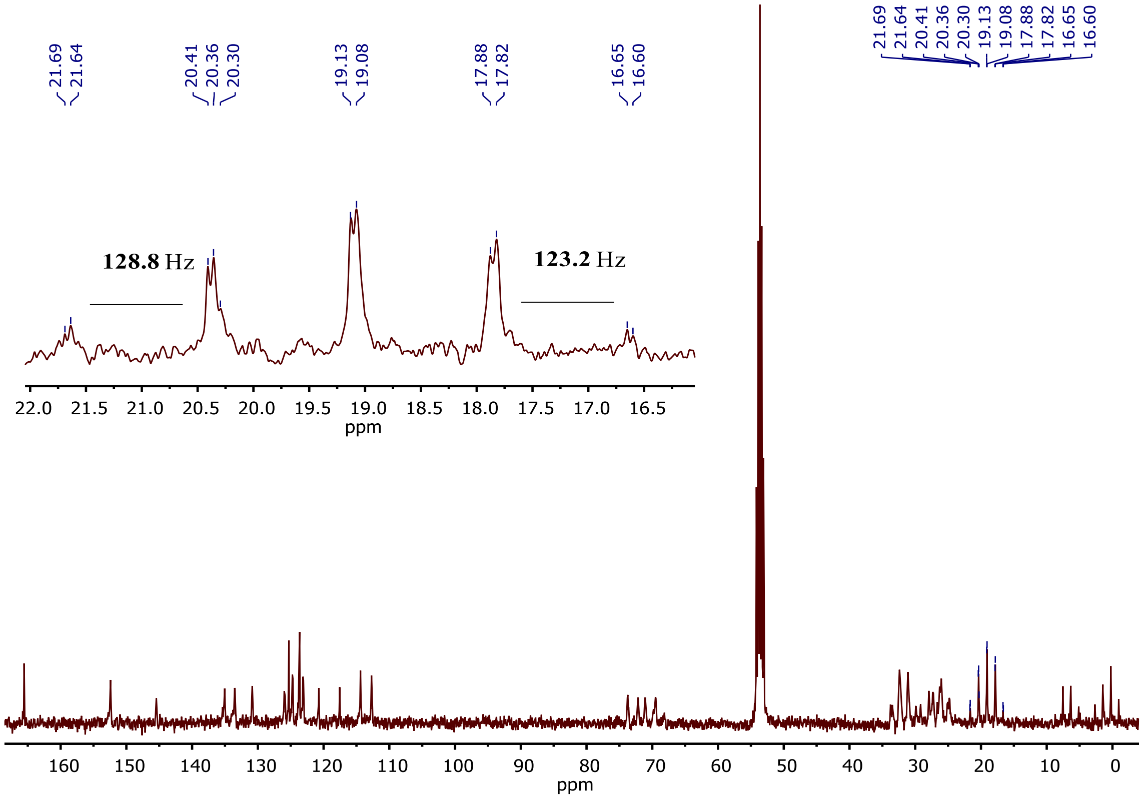

Figure S70. ^{13}C NMR spectrum of **3** in CD_2Cl_2 (101 MHz, 273K).

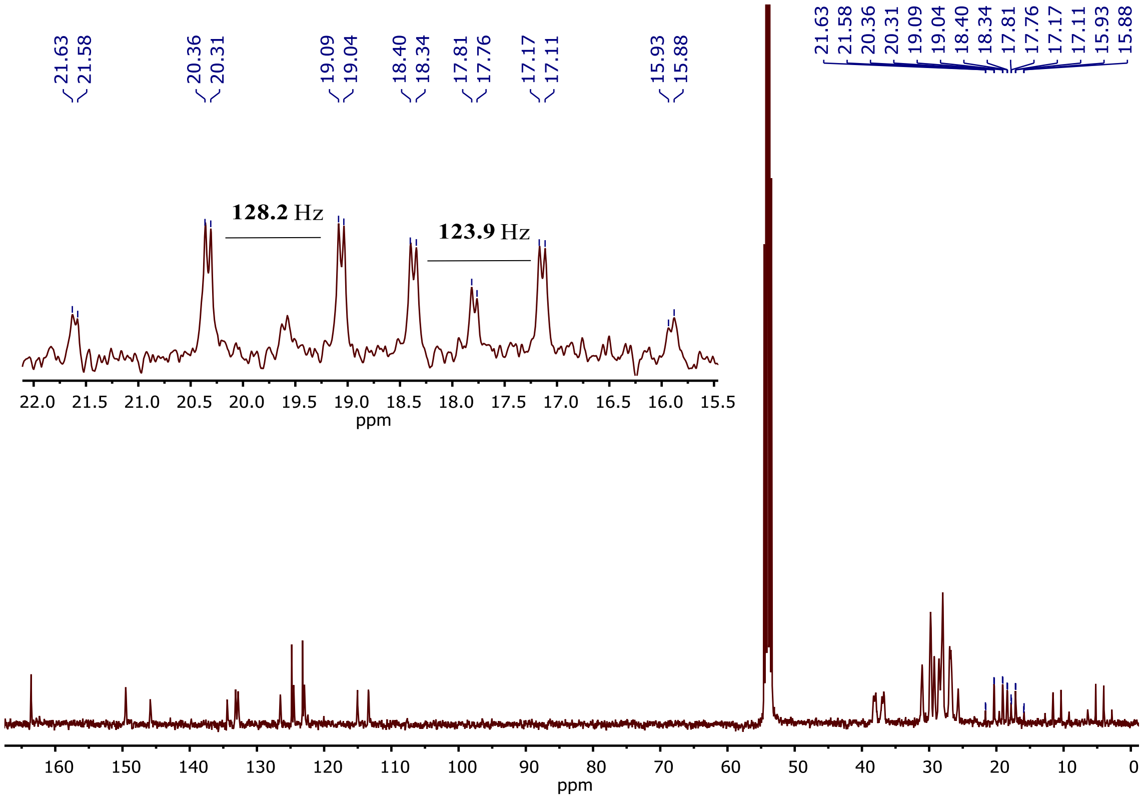

Figure S71. ^{13}C NMR spectrum of **4** in CD_2Cl_2 (101 MHz, 273K).

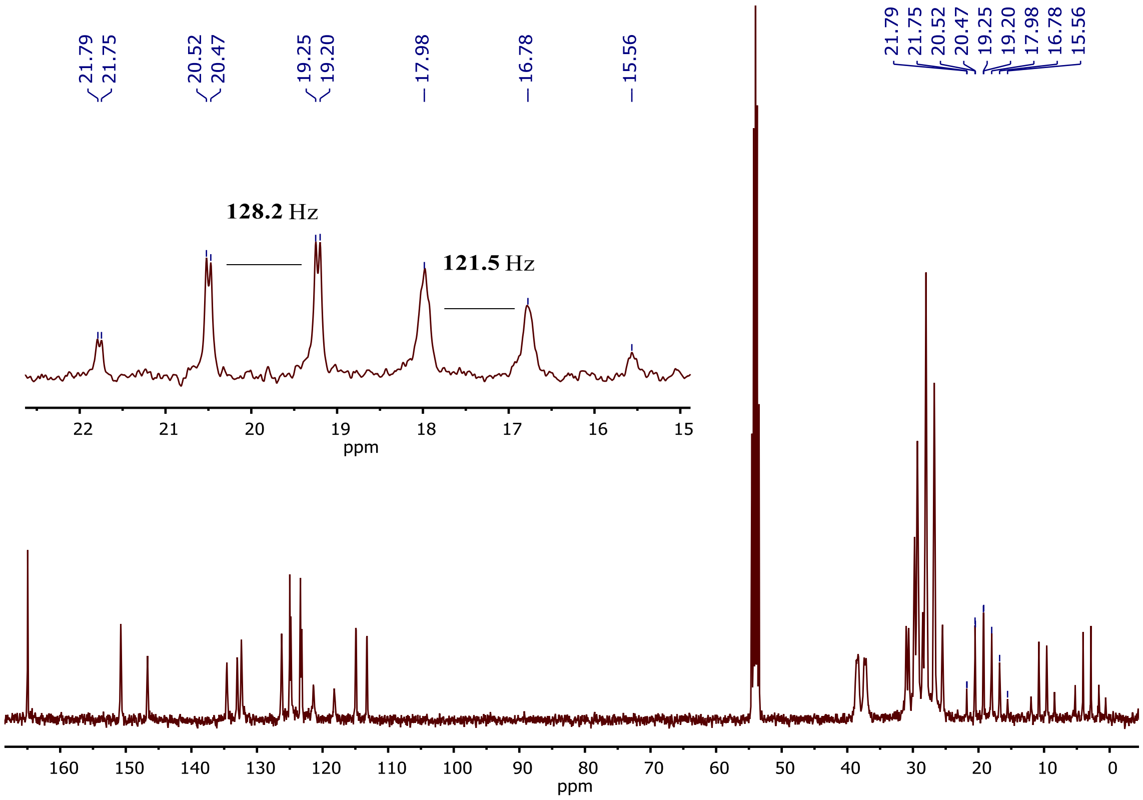

Figure S72. ^{13}C NMR spectrum of **5** in CD_2Cl_2 (101 MHz, 273K).

11.3. NMR spectra of reaction conditions optimization

NMR spectra of screening of iridium catalyst precursor

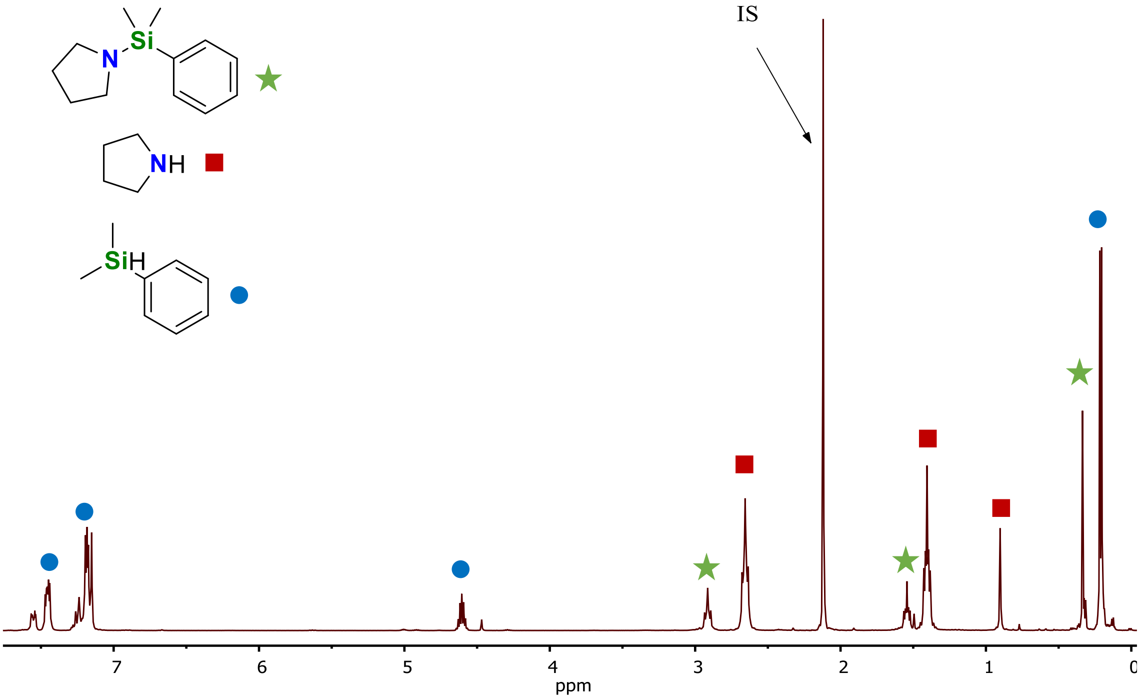

Figure S73. ^{1}H NMR spectrum of the catalytic reaction of pyrrolidine with $HSiMe_2Ph$ using **2** in C_6D_6 after 3 h at r.t. using hexamethylbenzene as IS.

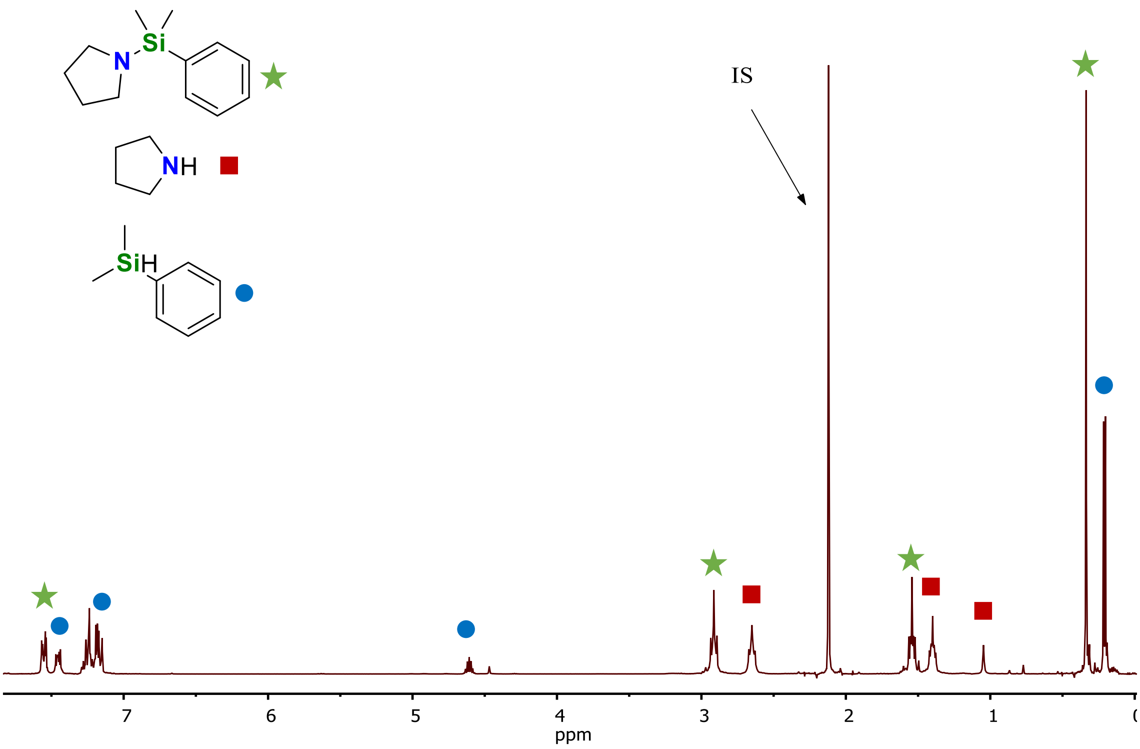

Figure S74. ^{1}H NMR spectrum of the catalytic reaction of pyrrolidine with $HSiMe_2Ph$ using **3** in C_6D_6 after 3 h at r.t. using hexamethylbenzene as IS.

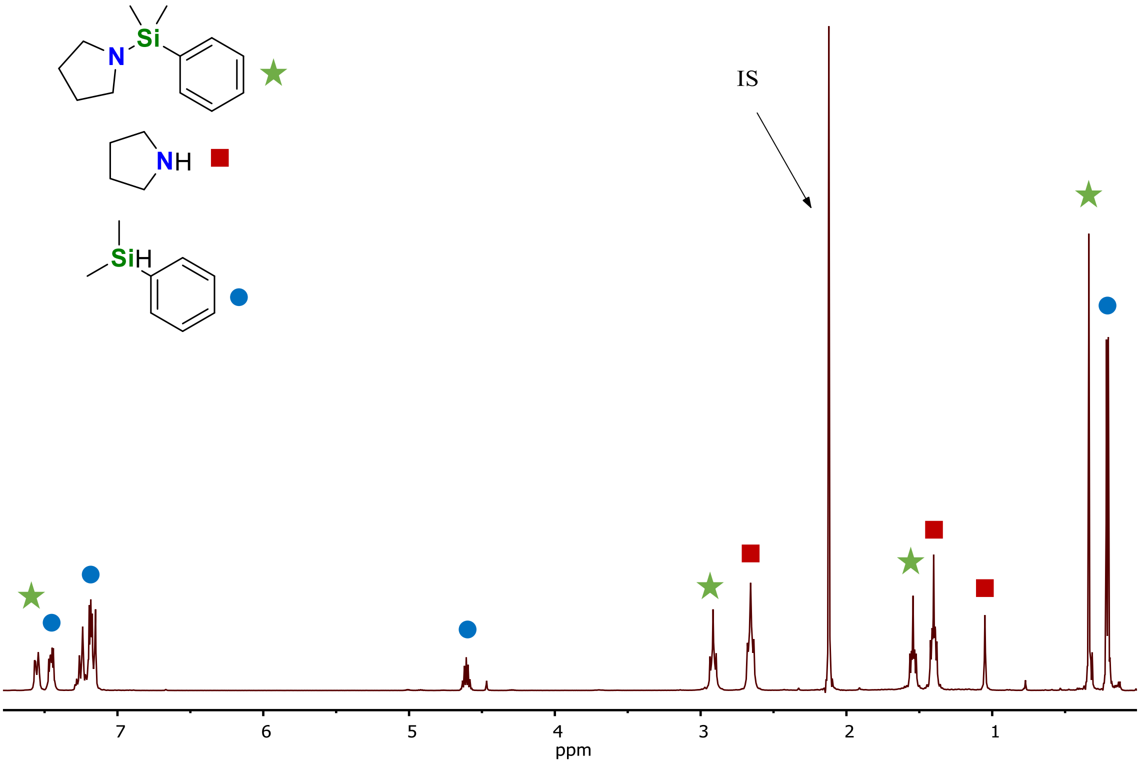

Figure S75. ^{1}H NMR spectrum of the catalytic reaction of pyrrolidine with $HSiMe_2Ph$ using **4** in C_6D_6 after 3 h at r.t. using hexamethylbenzene as IS.

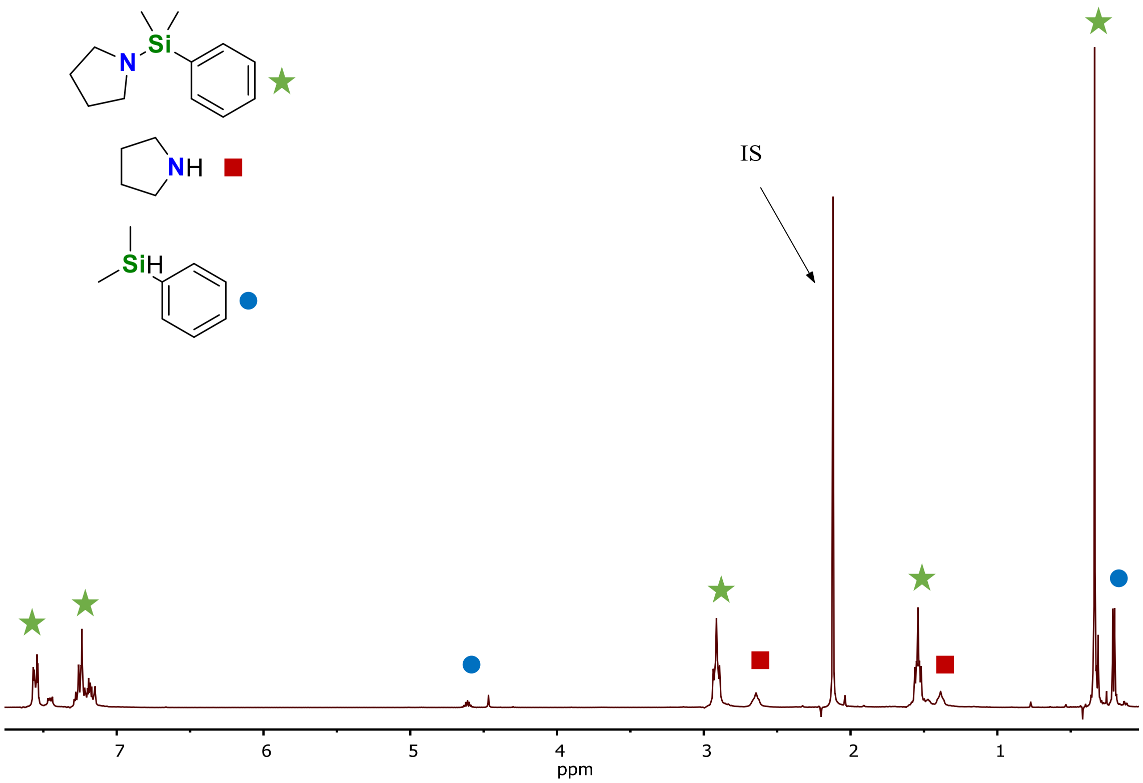

Figure S76. ^{1}H NMR spectrum of the catalytic reaction of pyrrolidine with $HSiMe_2Ph$ using **5** in C_6D_6 after 3 h at r.t. using hexamethylbenzene as IS.

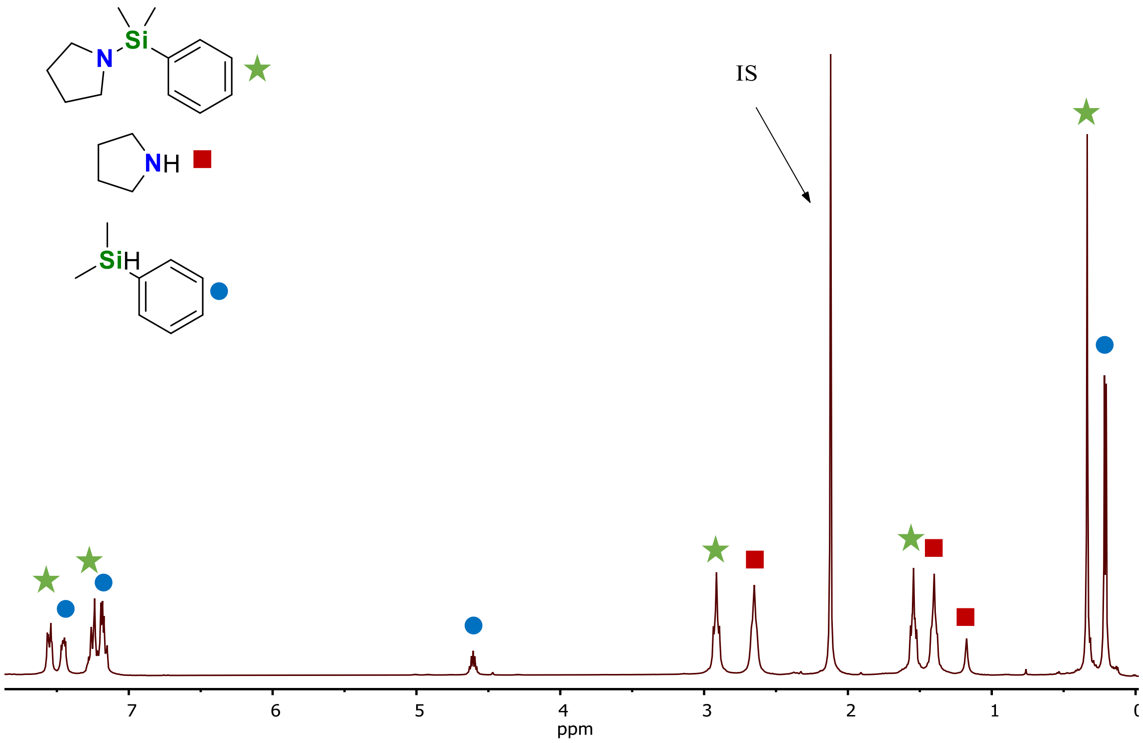

Figure S77. ^{1}H NMR spectrum of the catalytic reaction of pyrrolidine with $HSiMe_2Ph$ using **10** in C_6D_6 after 3 h at r.t. using hexamethylbenzene as IS.

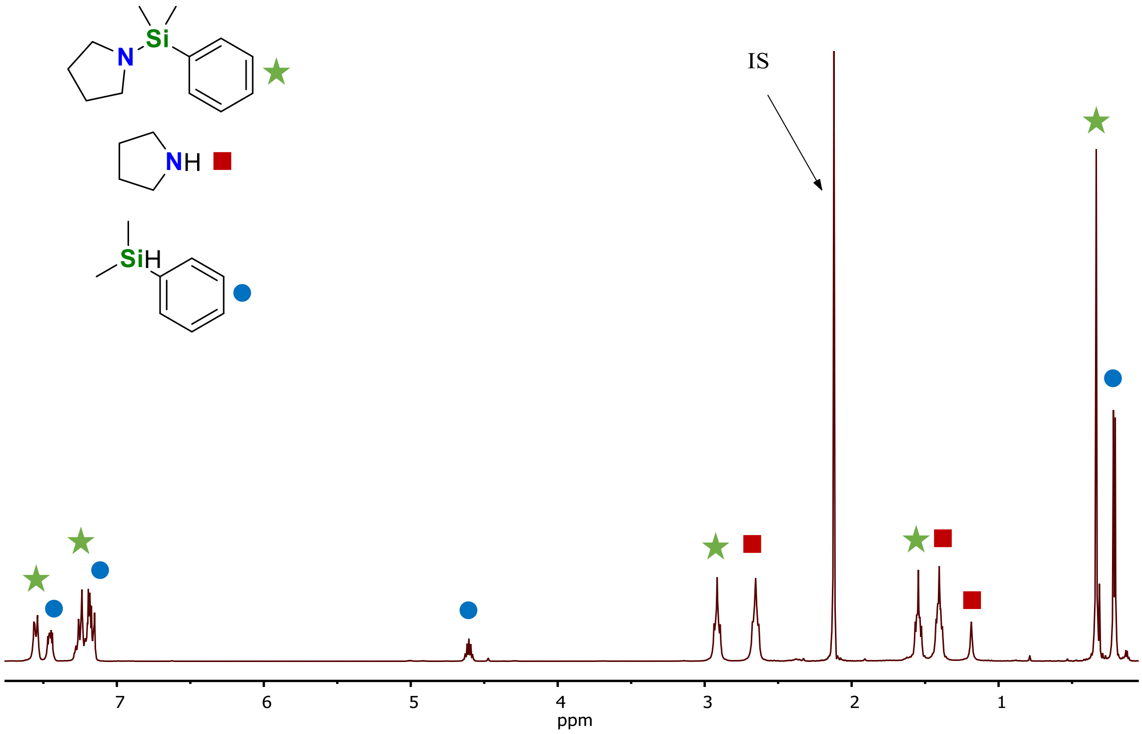

Figure S78. ^{1}H NMR spectrum of the catalytic reaction of pyrrolidine with $HSiMe_2Ph$ using **11** in C_6D_6 after 3 h at r.t. using hexamethylbenzene as IS.

NMR of screening of hydrosilanes

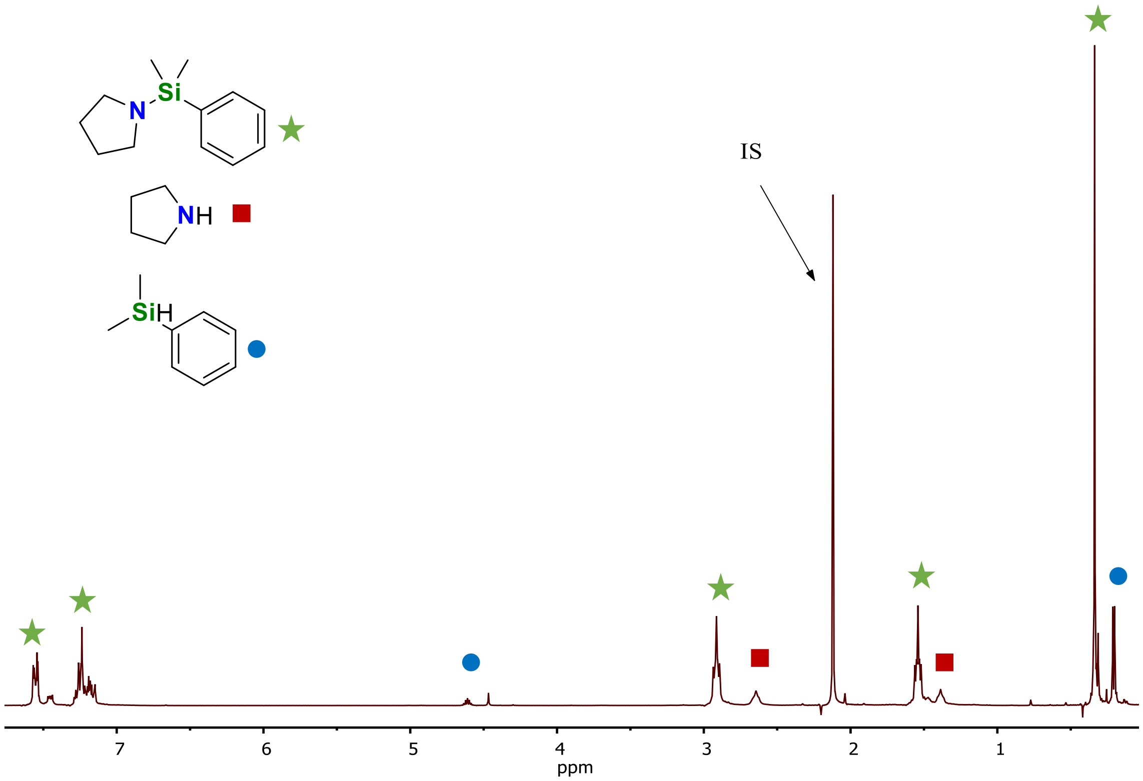

Figure S79. ^{1}H NMR spectrum of the **5**-catalyzed reaction of pyrrolidine with $HSiMe_2Ph$ in C_6D_6 after 3 h at r.t. using hexamethylbenzene as IS.

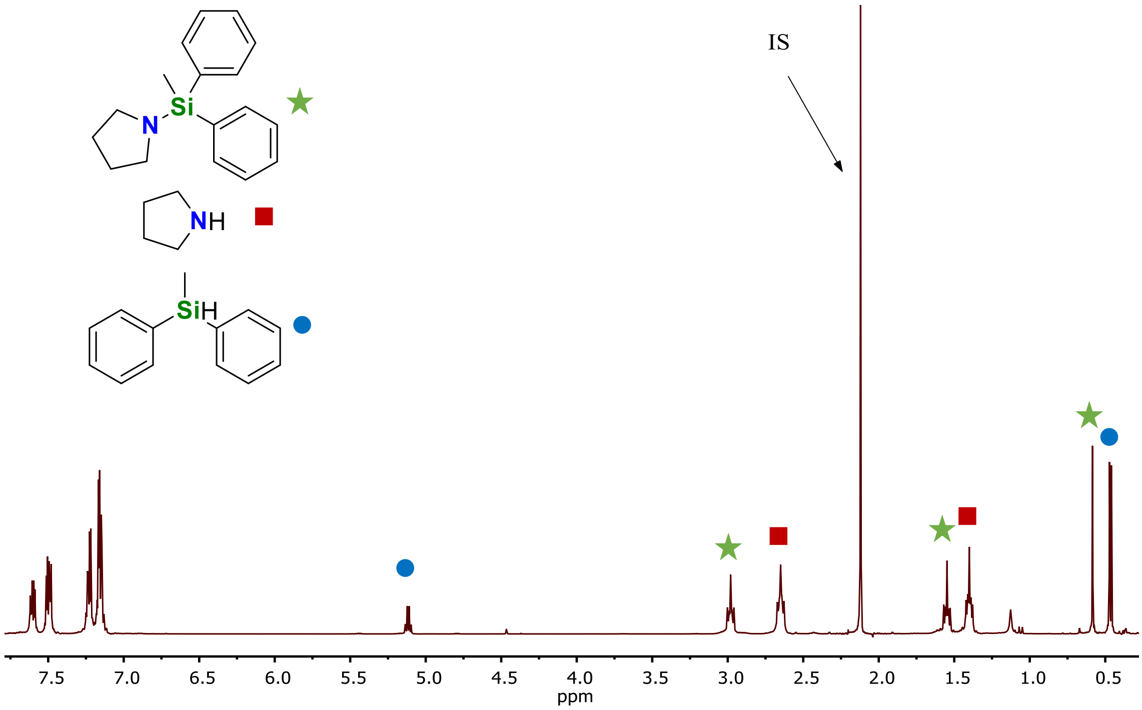

Figure S80. ^{1}H NMR spectrum of the **5**-catalyzed reaction of pyrrolidine with $HSiMePh_2$ in C_6D_6 after 3 h at r.t. using hexamethylbenzene as IS.

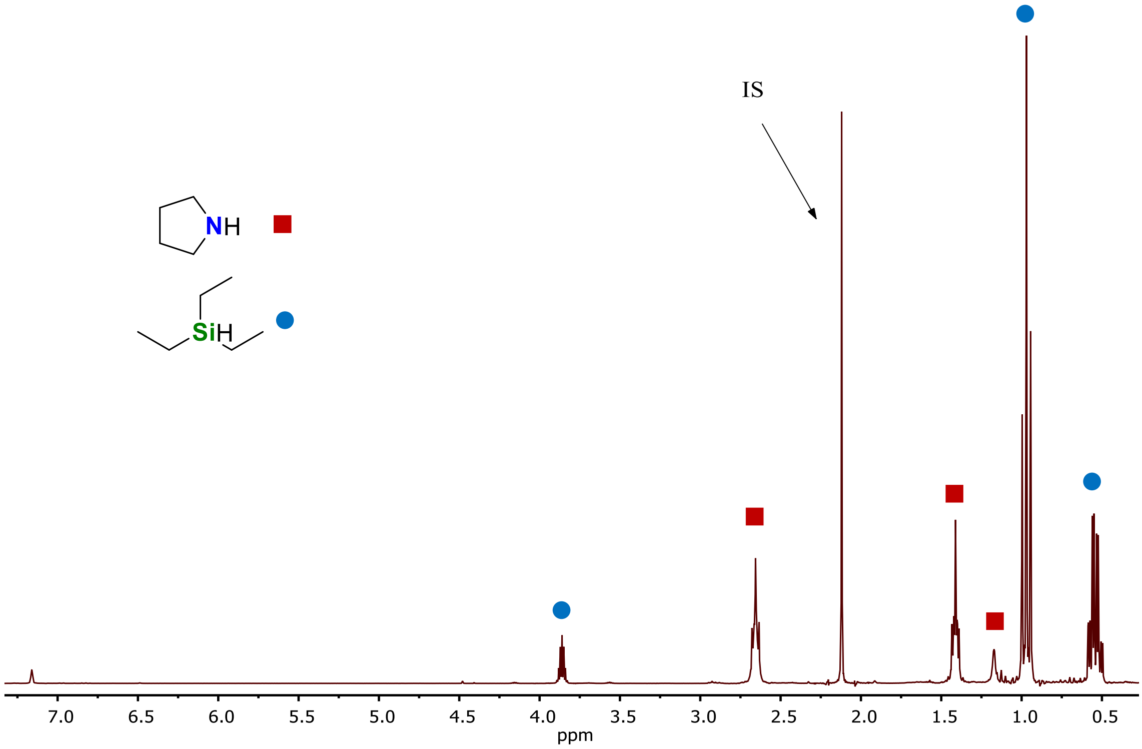

Figure S81. ^{1}H NMR spectrum of the **5**-catalyzed reaction of pyrrolidine with $HSiEt_3$ in C_6D_6 after 3 h at r.t. using hexamethylbenzene as IS.

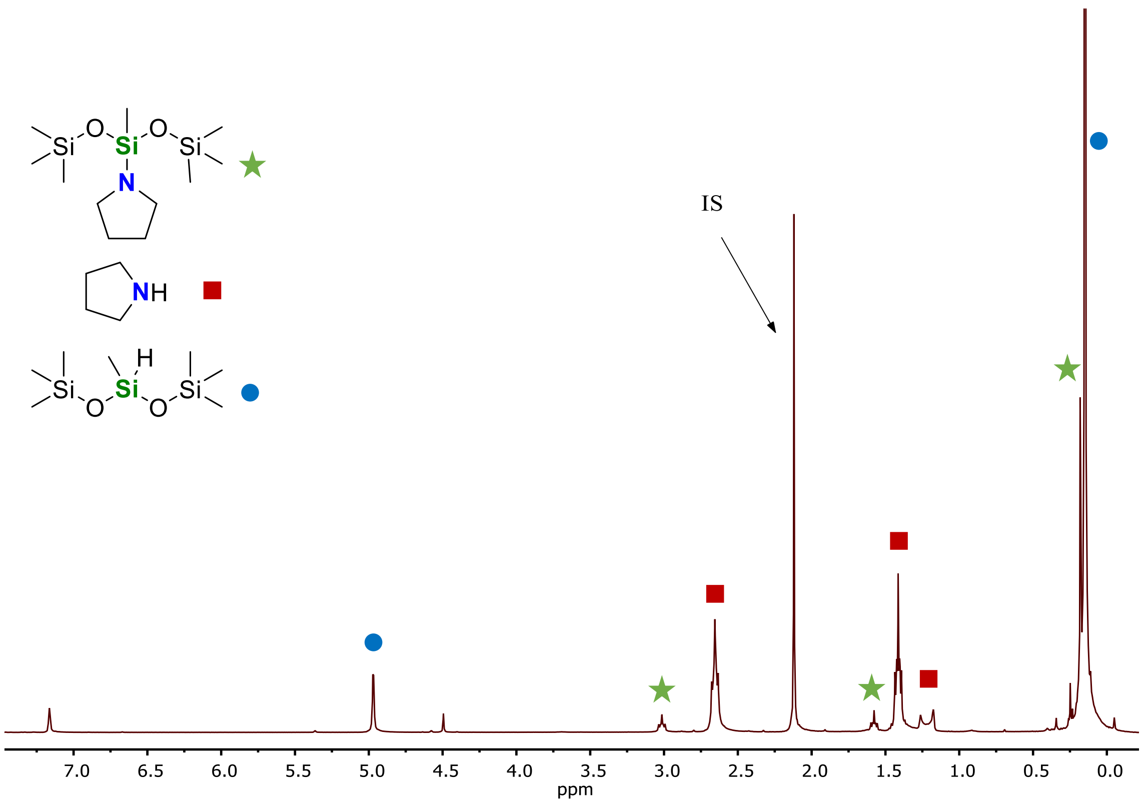

Figure S82. ^{1}H NMR spectrum of the **5**-catalyzed reaction of pyrrolidine with $HSiMe(OSiMe_3)_2$ in C_6D_6 after 3 h at r.t. using hexamethylbenzene as IS.

11.4. NMR spectra of silylamines

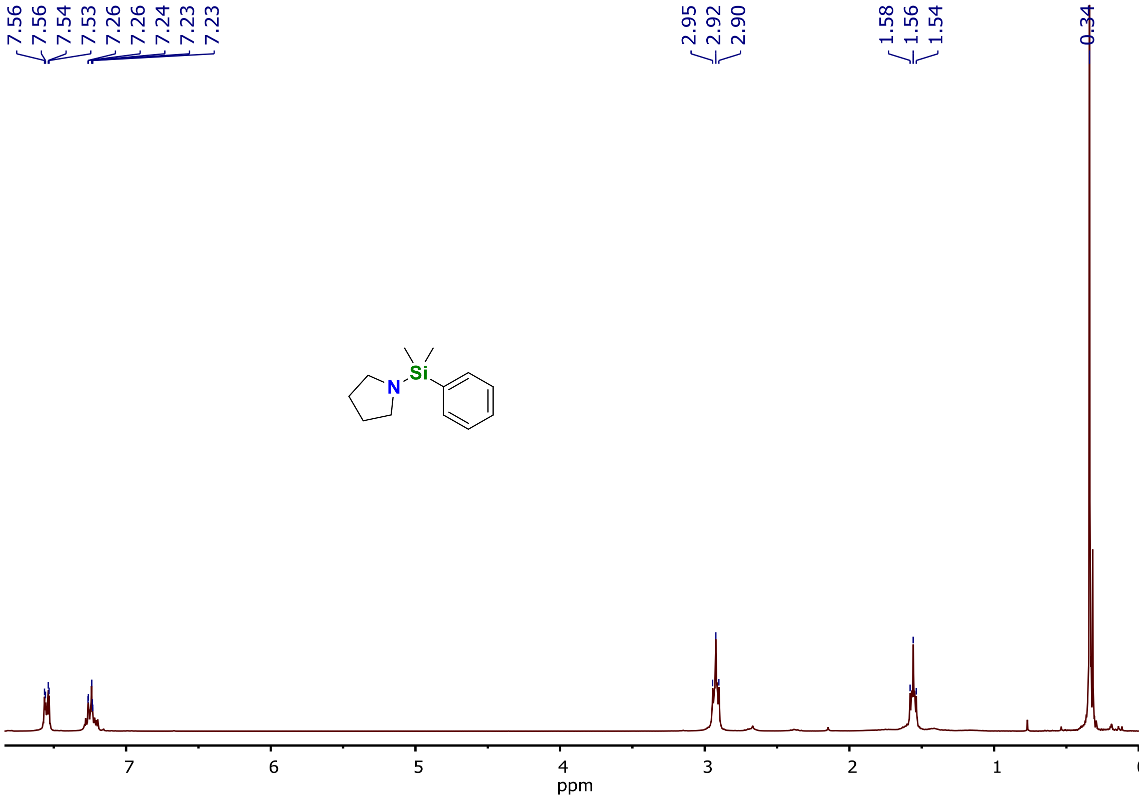

Figure S83. ^{1}H NMR spectrum of **6a** in C_6D_6 (300 MHz, 298K).

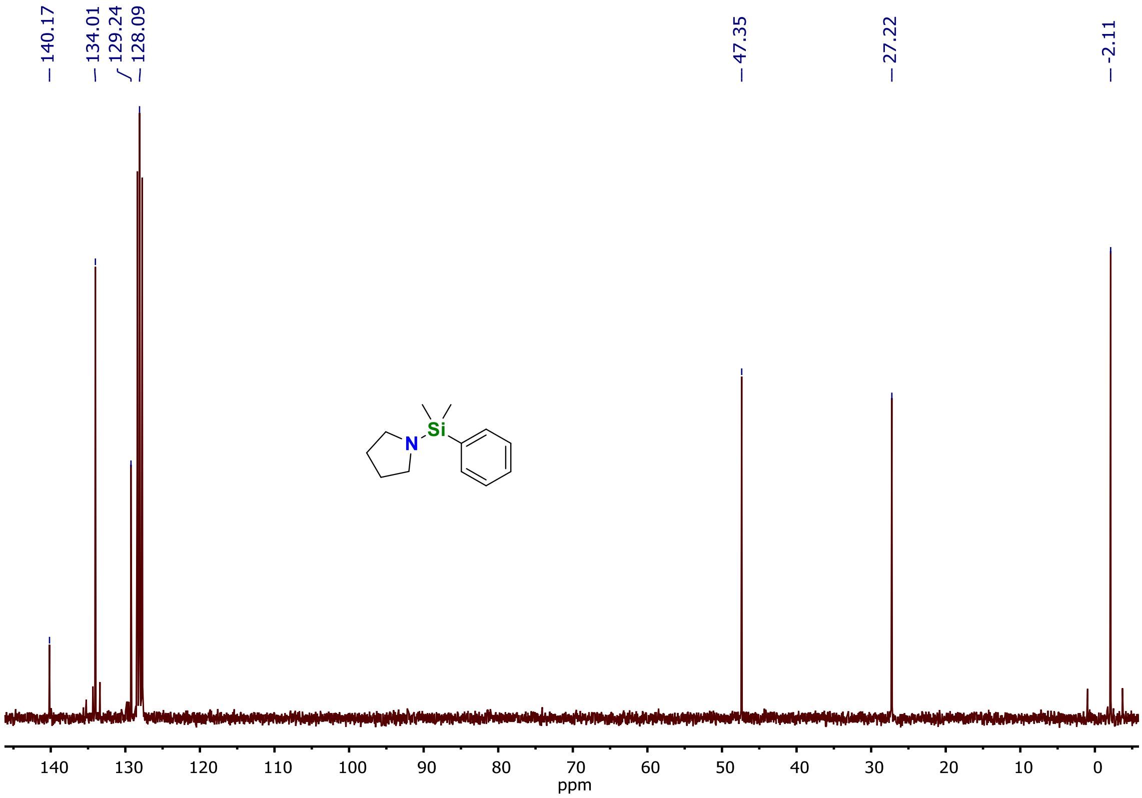

Figure S84. $^{13}C\{^{1}H\}$ NMR spectrum of **6a** in C_6D_6 (75 MHz, 298K).

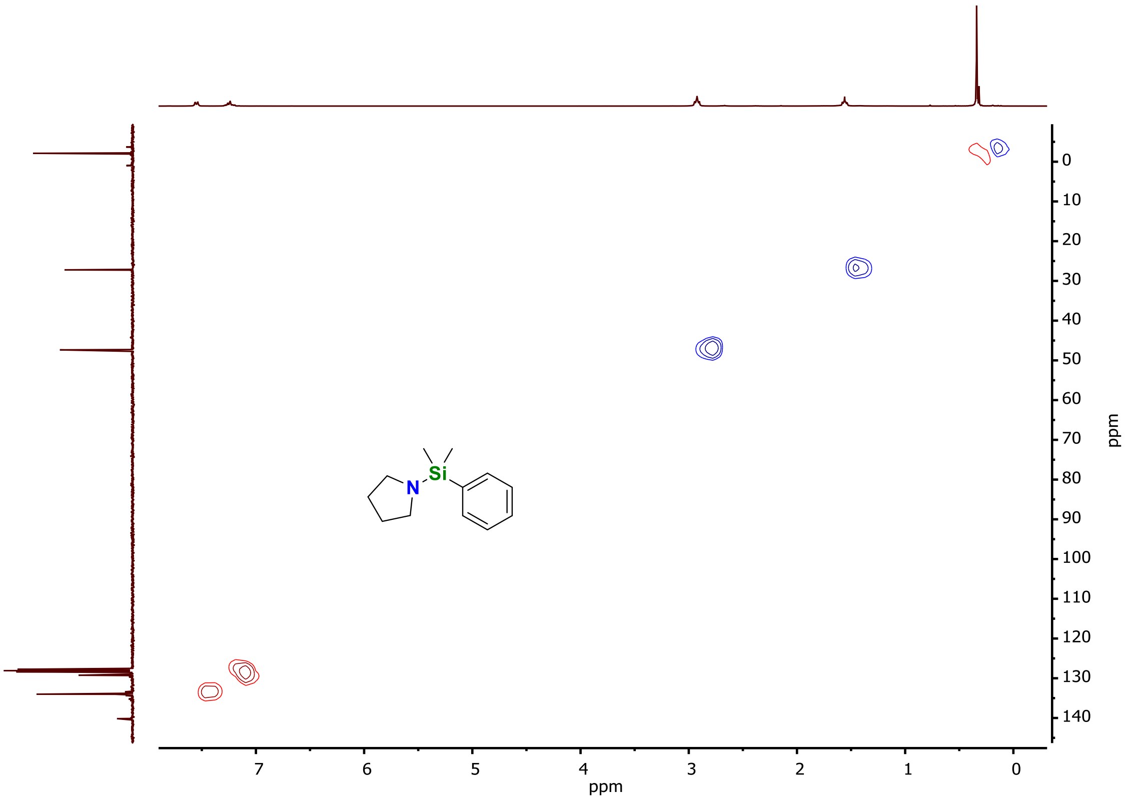

Figure S85. ^{1}H-^{13}C HSQC NMR spectrum of **6a** in C_6D_6 (298K).

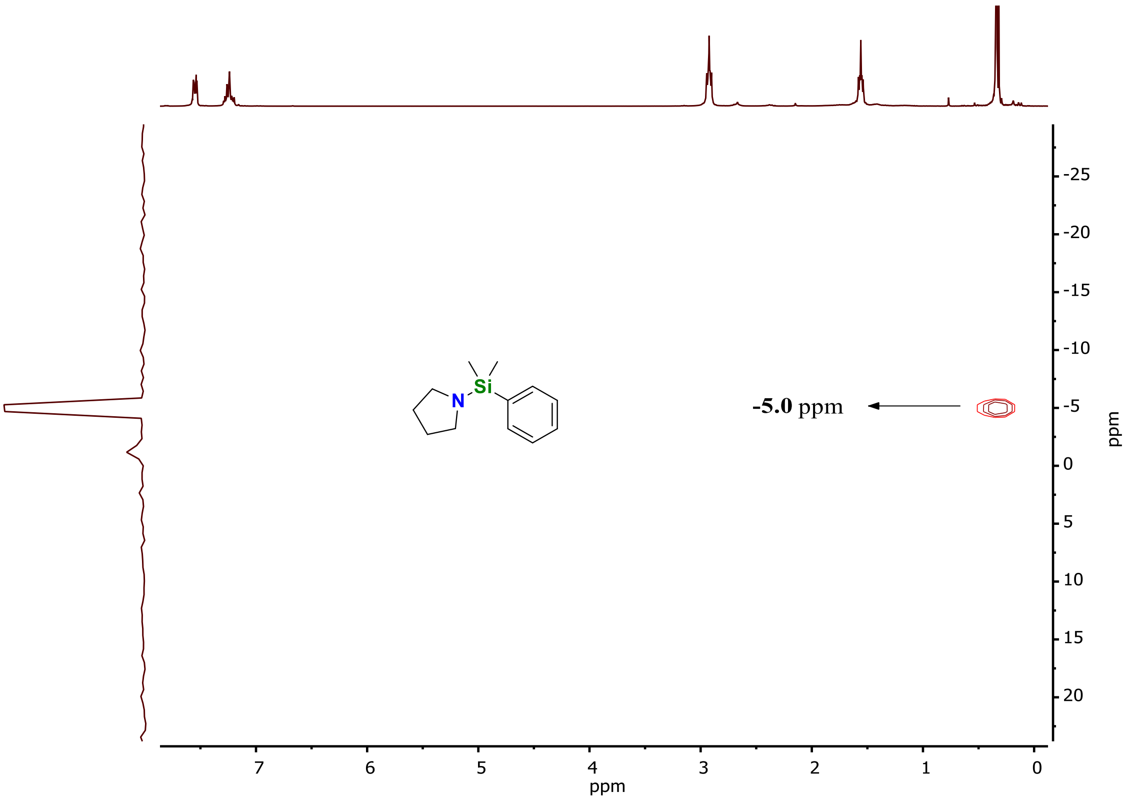

Figure S86. ^{1}H-^{29}Si HMQC NMR spectrum of **6a** in C_6D_6 (298K).

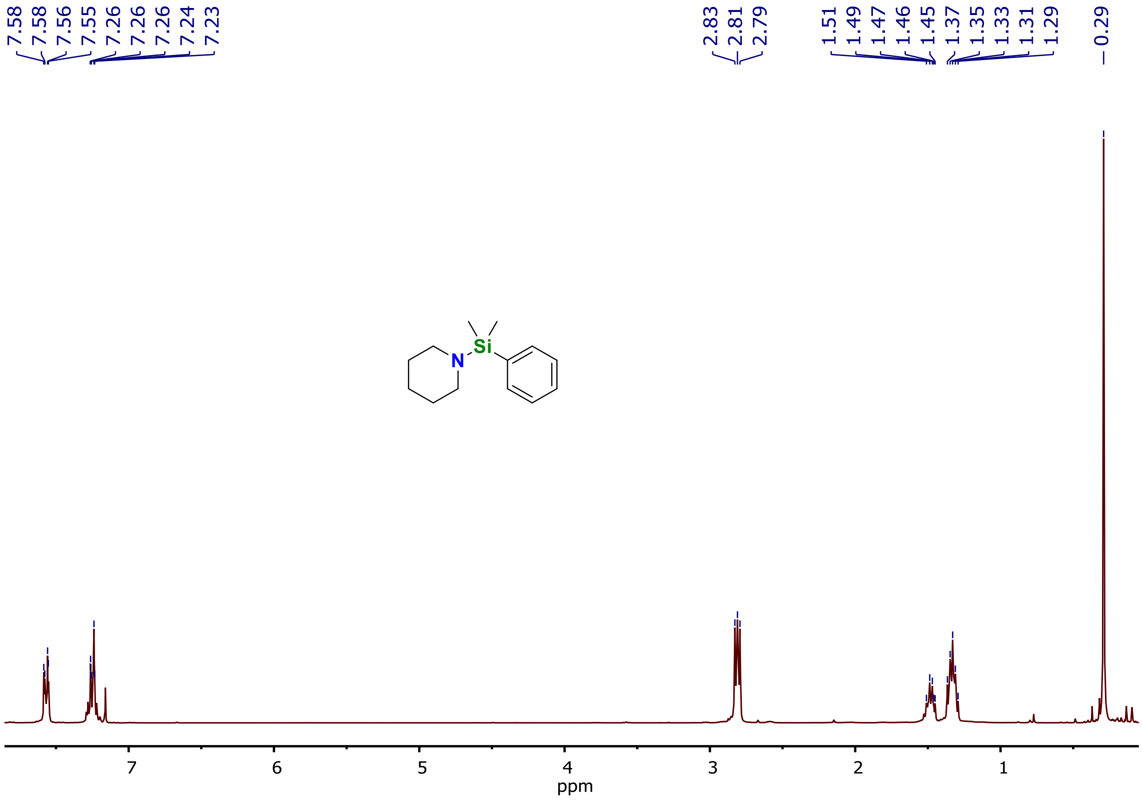

Figure S87. ^{1}H NMR spectrum of **6b** in C_6D_6 (300 MHz, 298K).

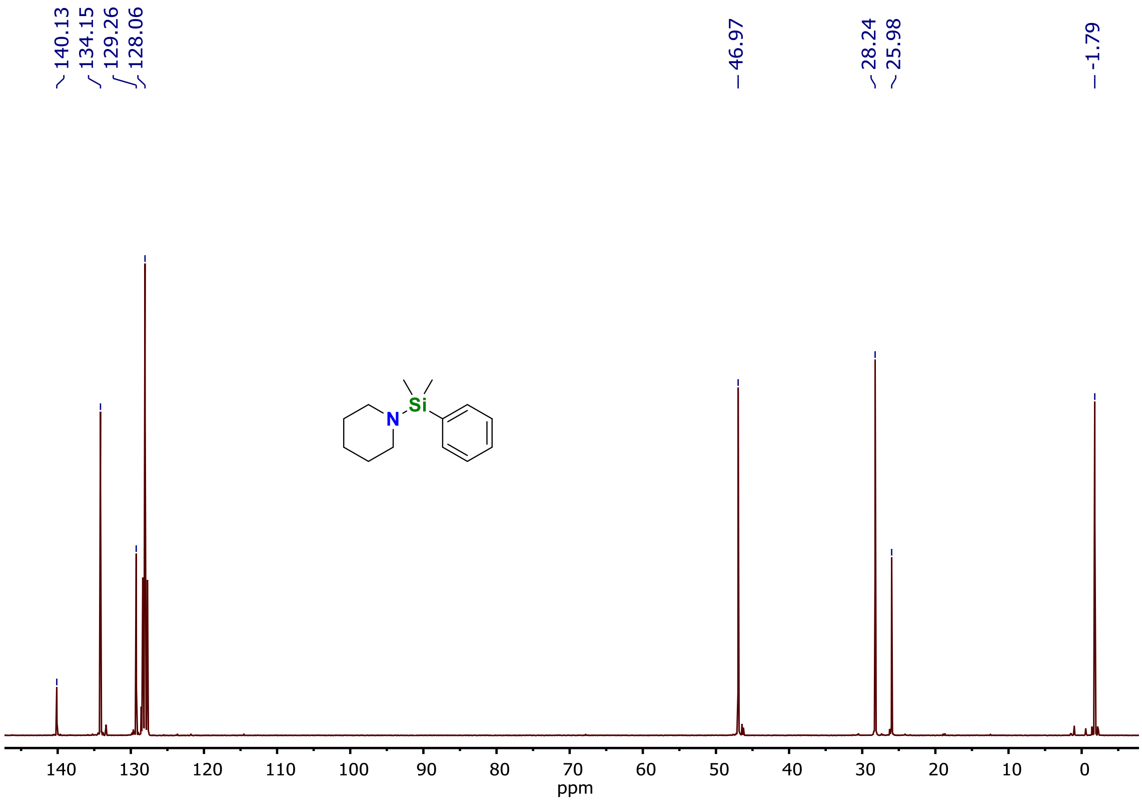

Figure S88. ^{13}C{^{1}H} NMR spectrum of **6b** in C_6D_6 (75 MHz, 298K).

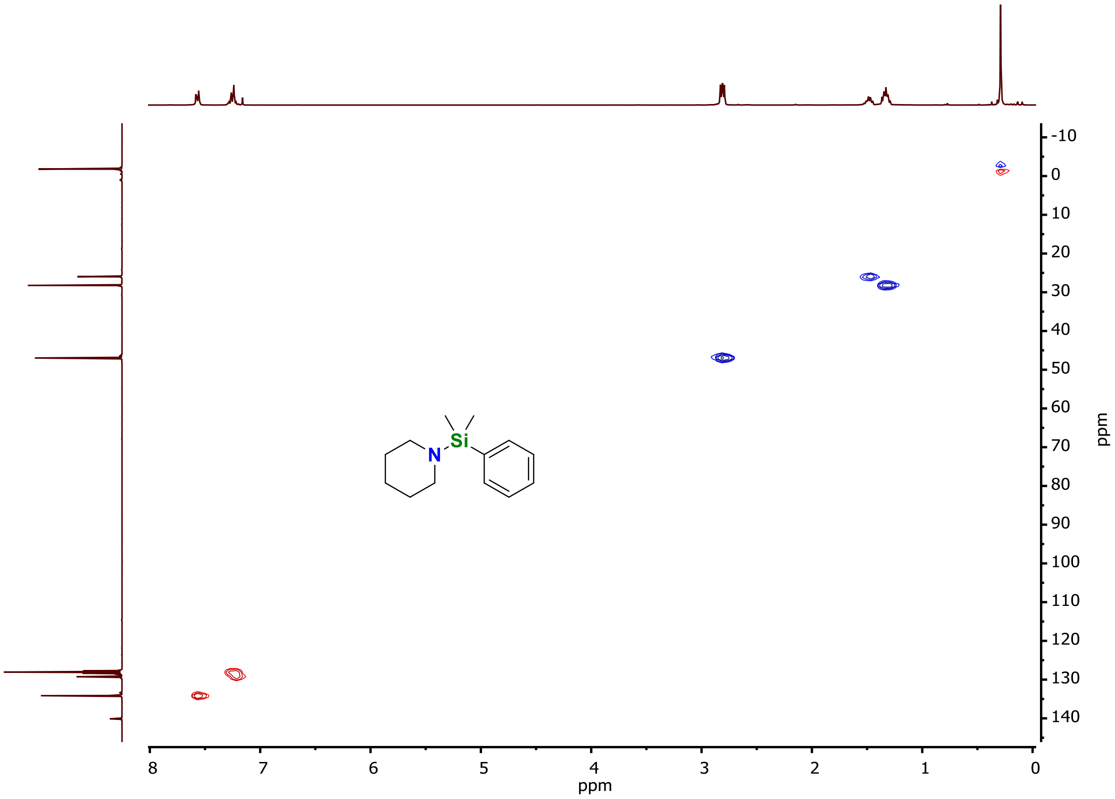

Figure S89. ^{1}H-^{13}C HSQC NMR spectrum of **6b** in C_6D_6 (298K).

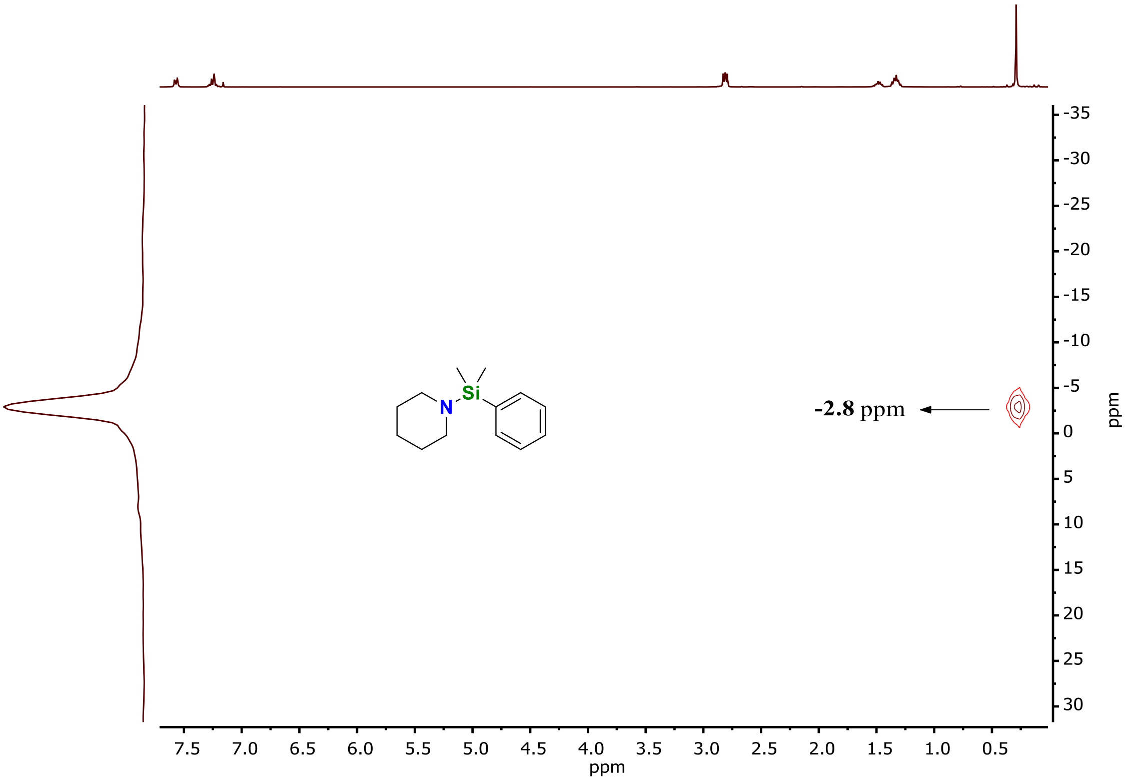

Figure S90. ^{1}H-^{29}Si HMQC NMR spectrum of **6b** in C_6D_6 (298K).

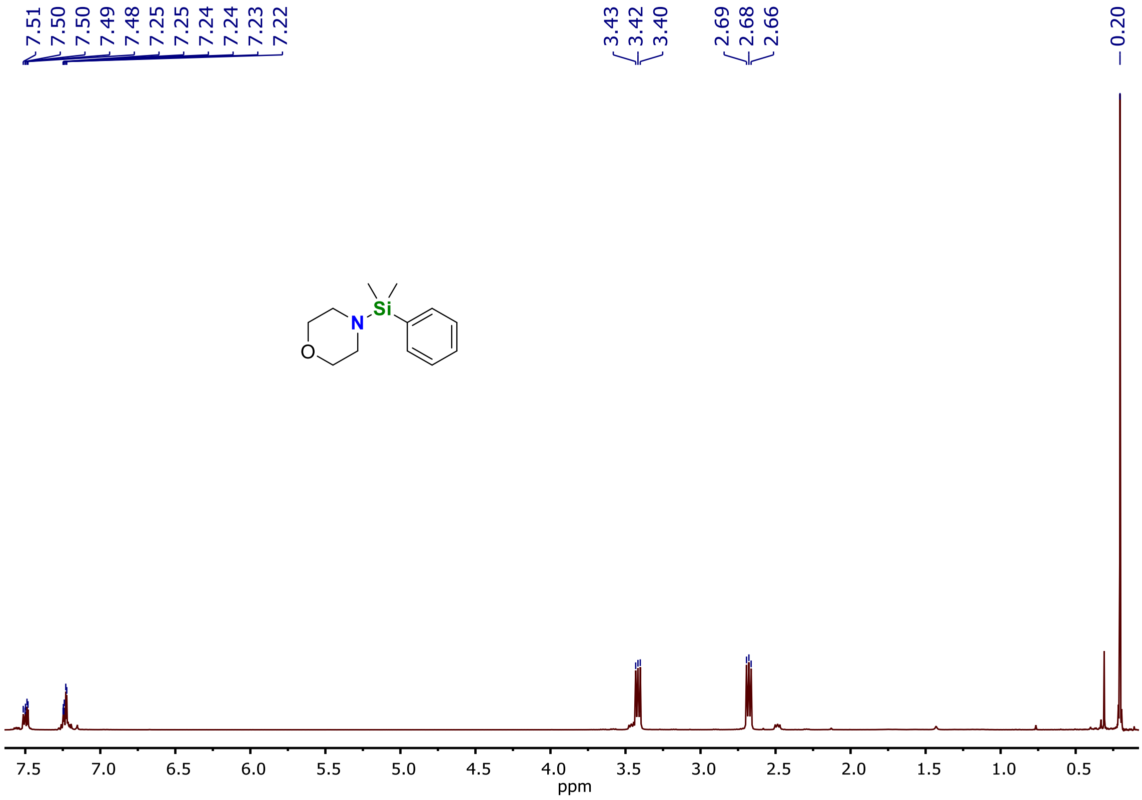

Figure S91. ^{1}H NMR spectrum of **6c** in C_6D_6 (300 MHz, 298K).

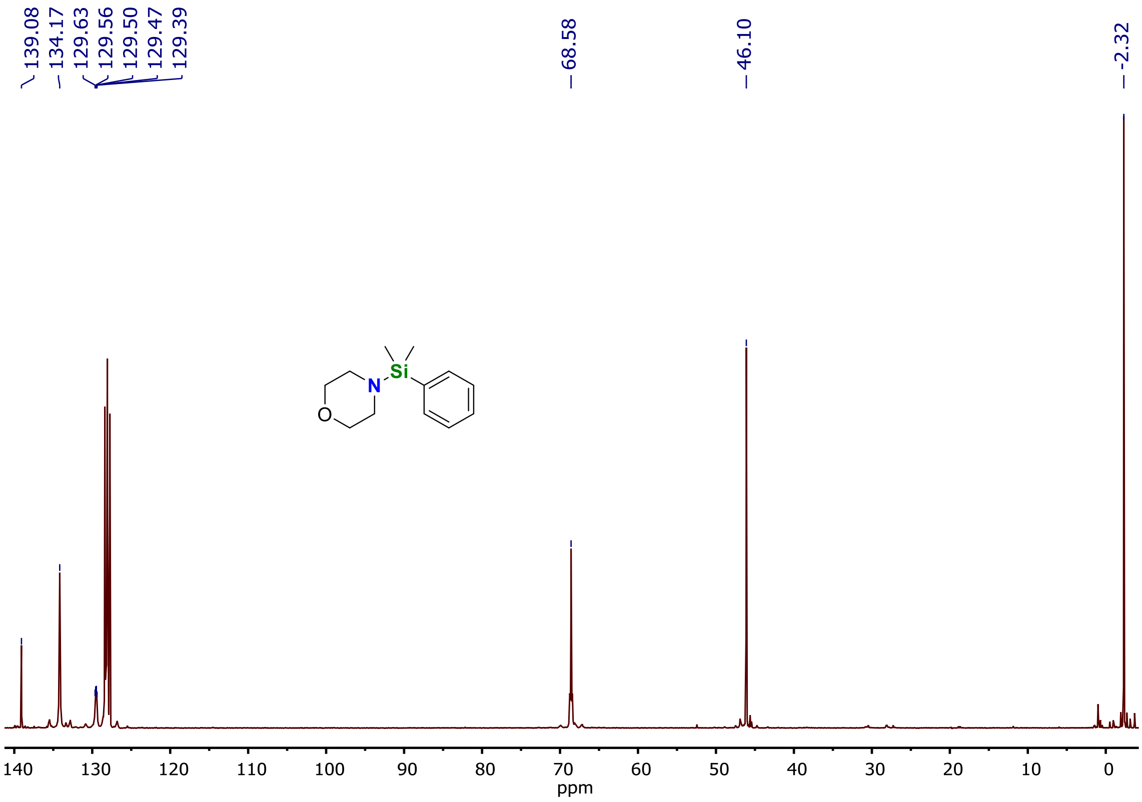

Figure S92. $^{13}C\{^1H\}$ NMR spectrum of **6c** in C_6D_6 (75 MHz, 298K).

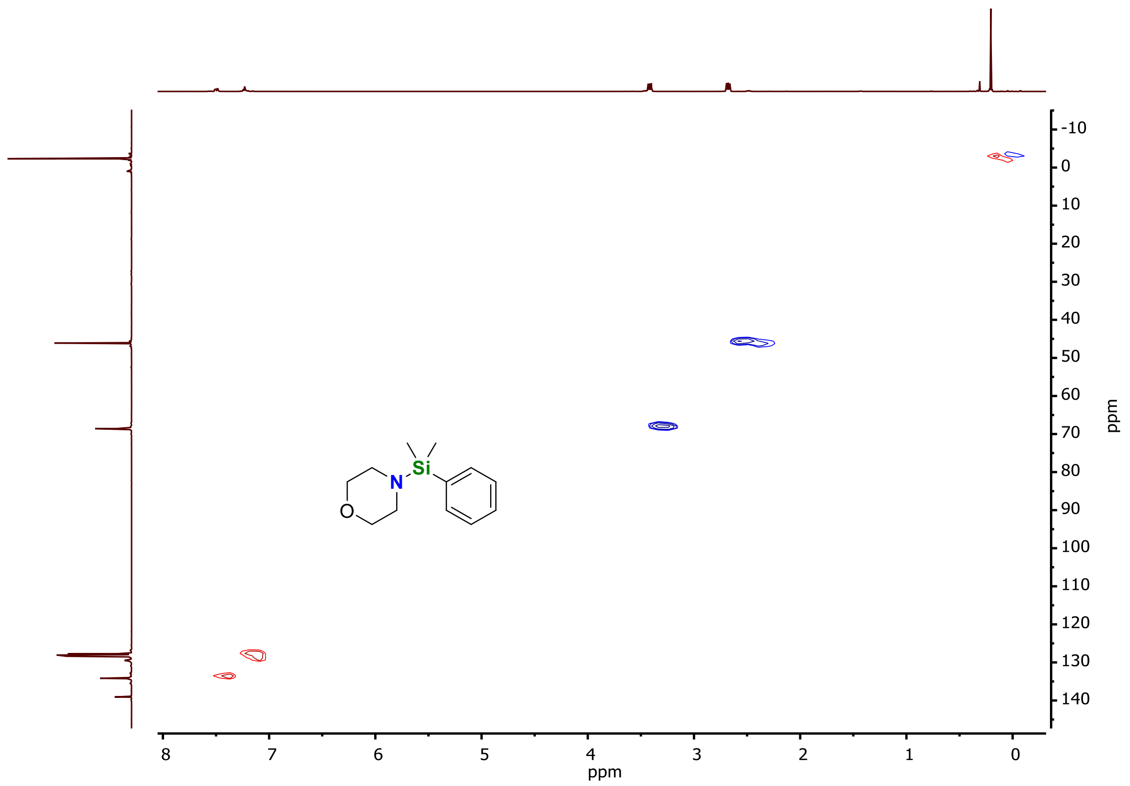

Figure S93. ^{1}H-^{13}C HSQC NMR spectrum of **6c** in C_6D_6 (298K).

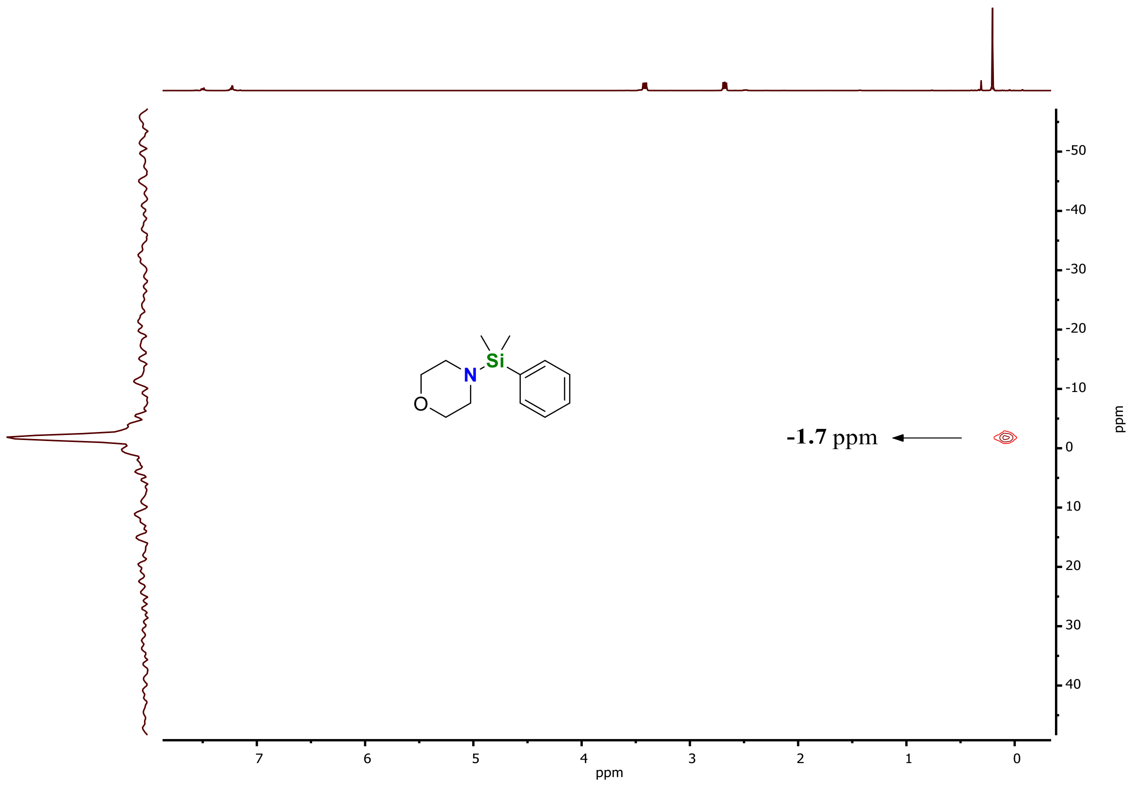

Figure S94. ^{1}H-^{29}Si HMQC NMR spectrum of **6c** in C_6D_6 (298K).

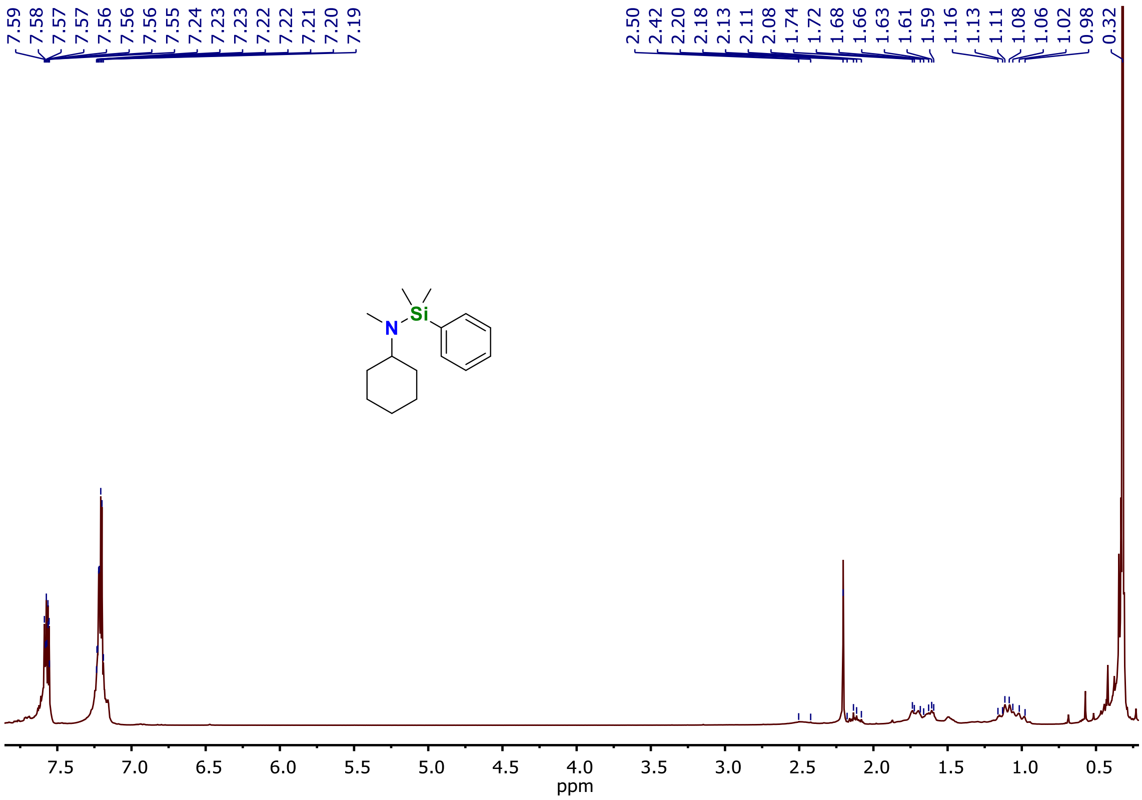

Figure S95. ^{1}H NMR spectrum of **6d** in C_6D_6 (300 MHz, 298K).

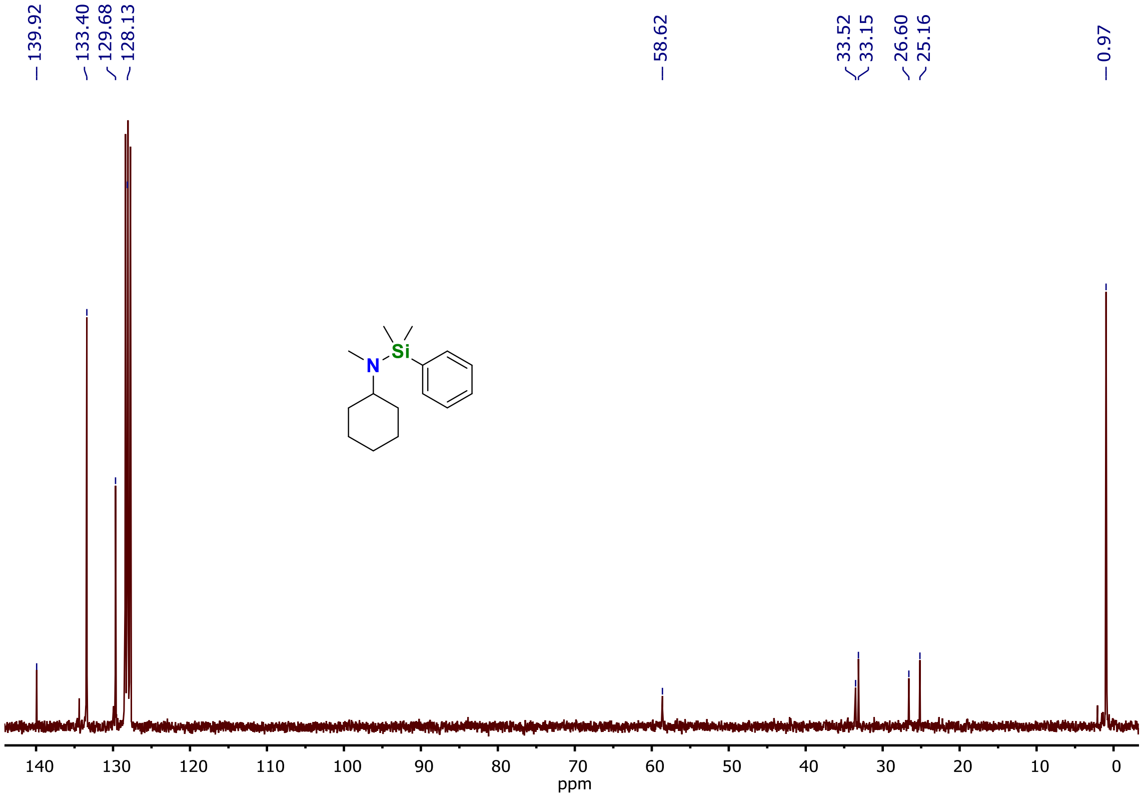

Figure S96. ^{13}C{^{1}H} NMR spectrum of **6d** in C_6D_6 (75 MHz, 298K).

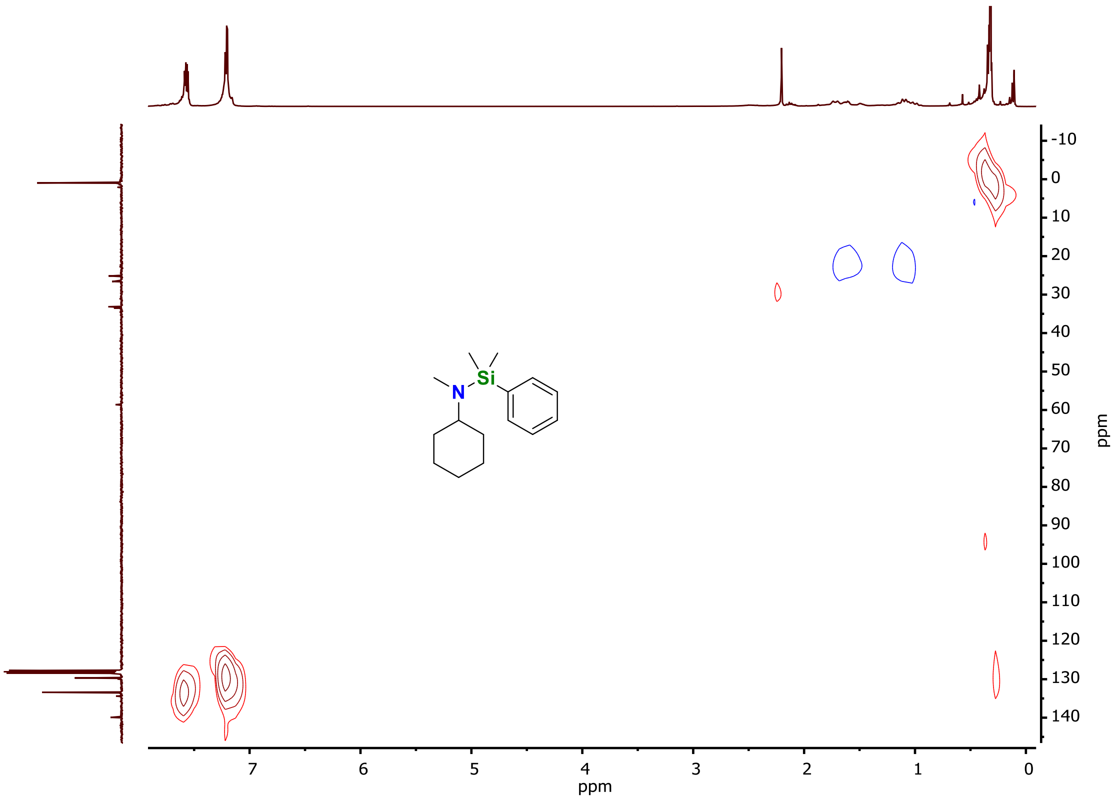

Figure S97. ^{1}H-^{13}C HSQC NMR spectrum of **6d** in C_6D_6 (298K).

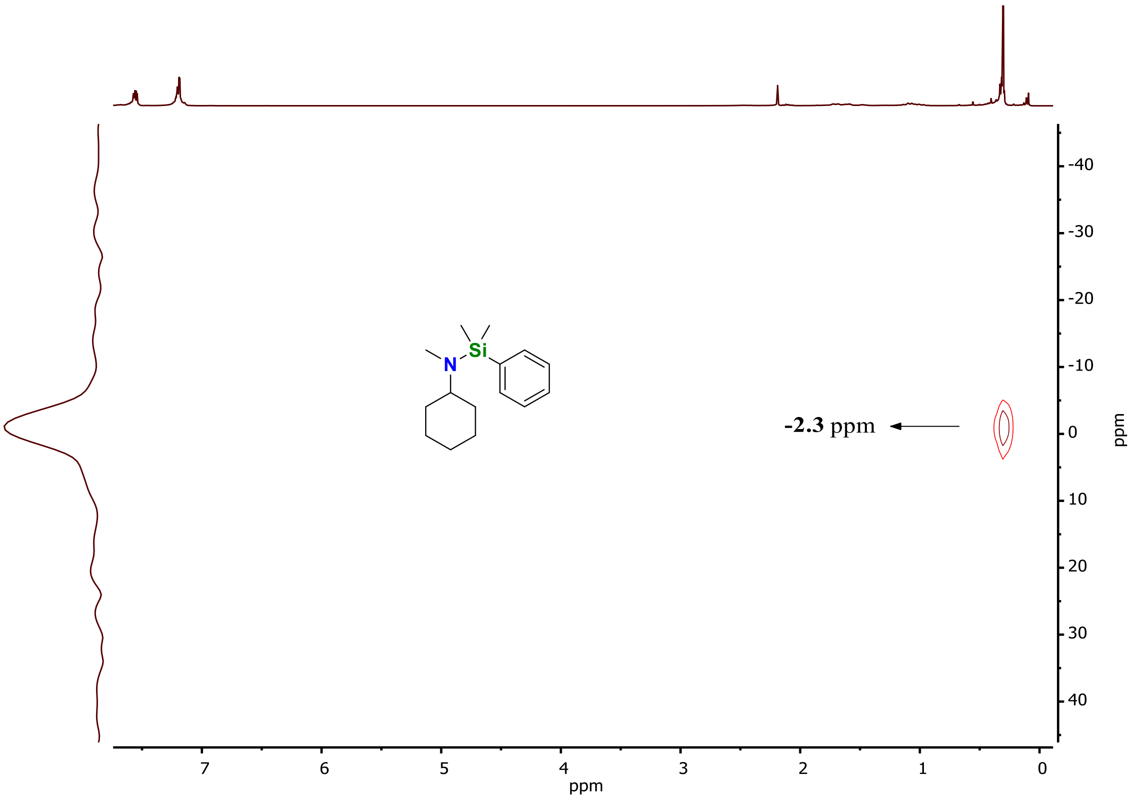

Figure S98. ^{1}H-^{29}Si HMQC NMR spectrum of **6d** in C_6D_6 (298K).

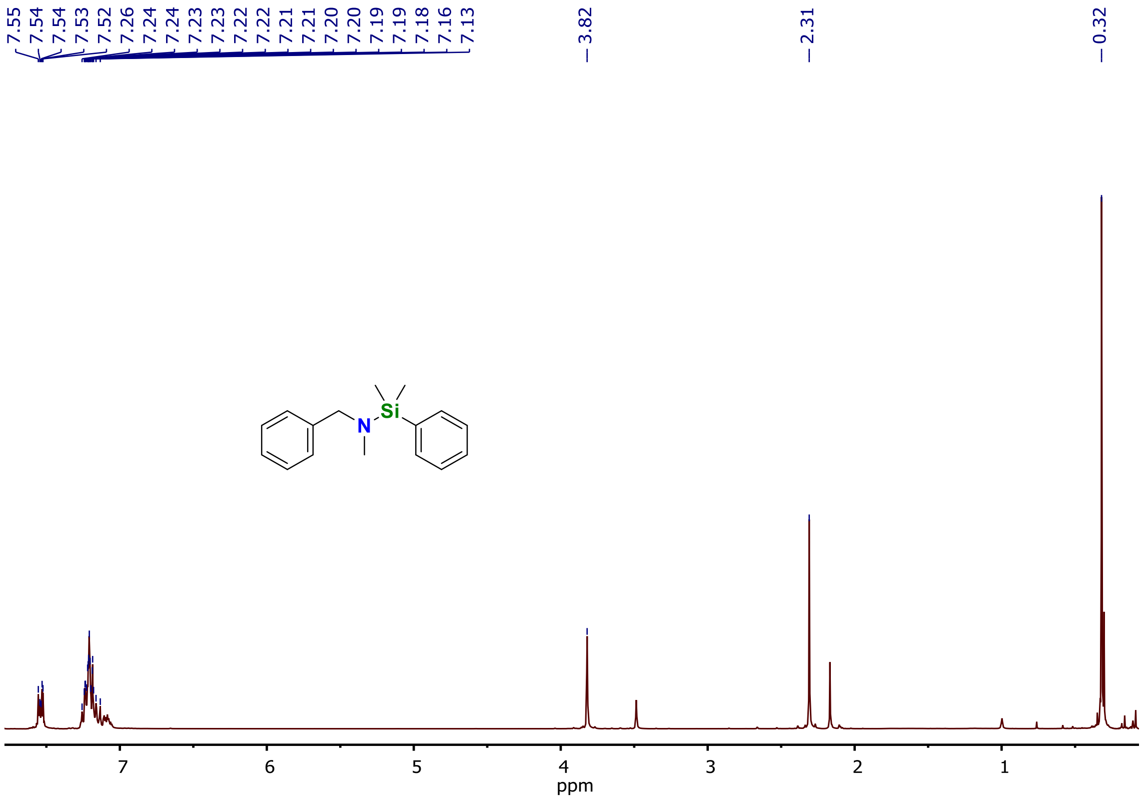

Figure S99. ^{1}H NMR spectrum of **6e** in C_6D_6 (300 MHz, 298K).

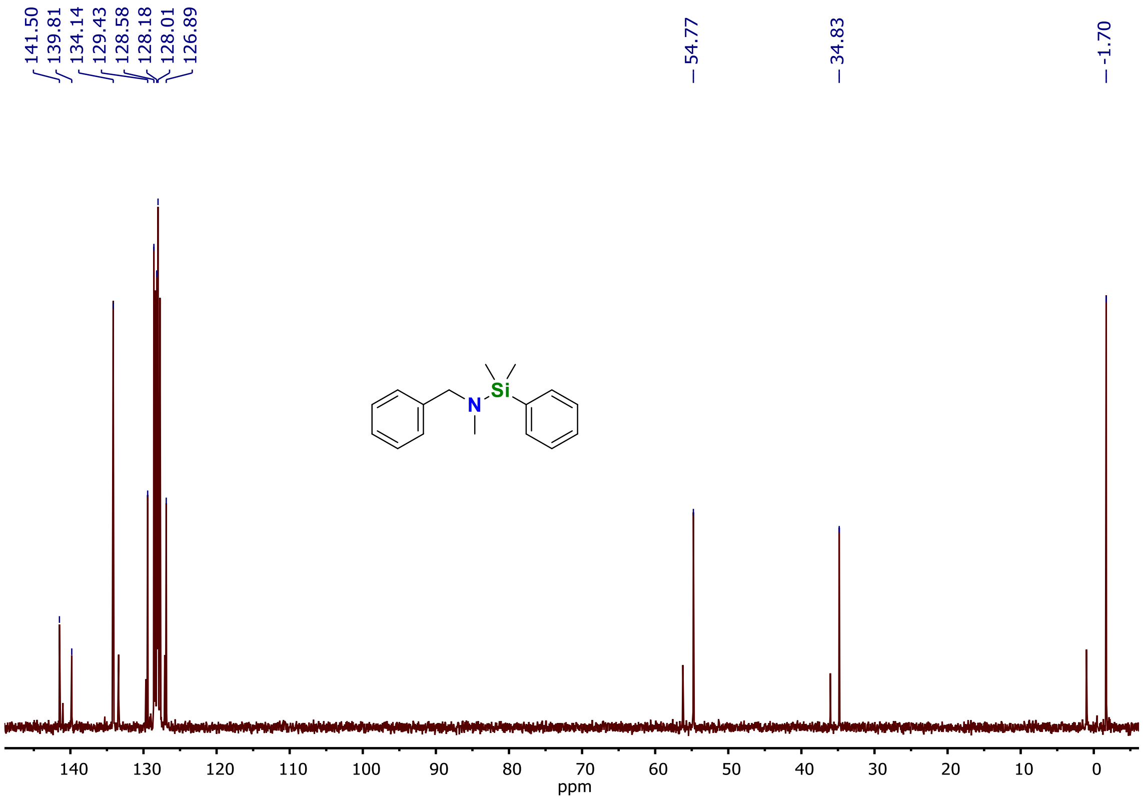

Figure S100. $^{13}C\{^{1}H\}$ NMR spectrum of **6e** in C_6D_6 (75 MHz, 298K).

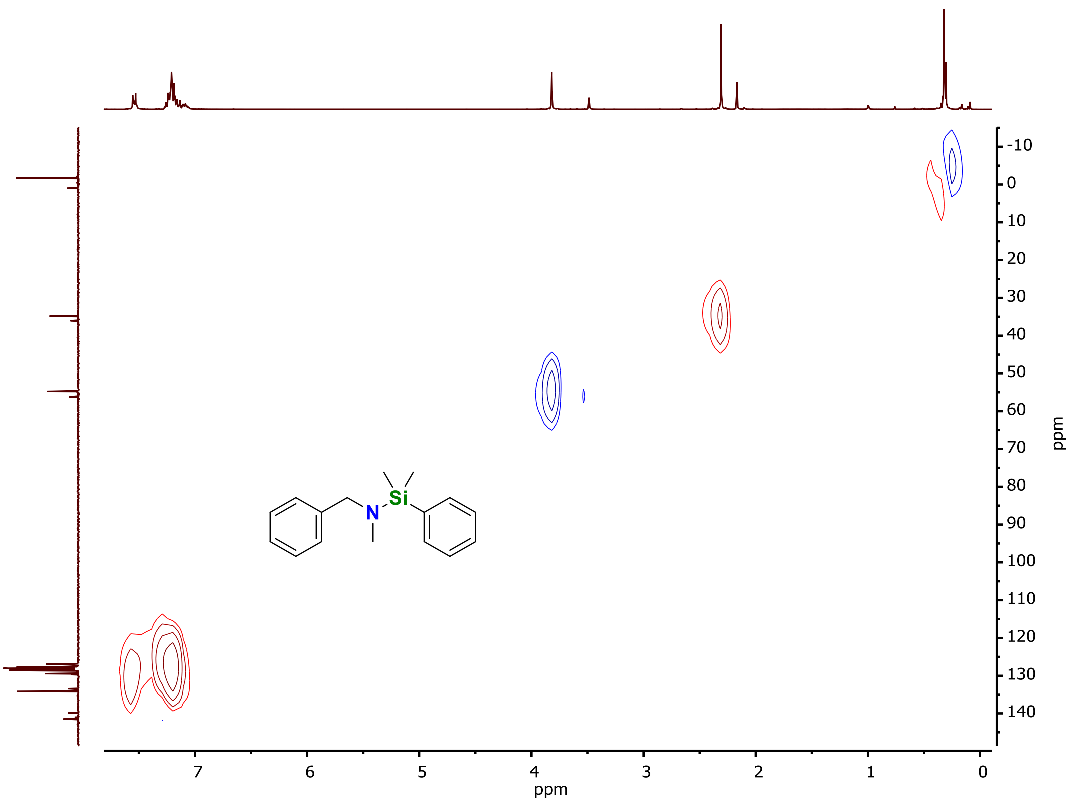

Figure S101. ^{1}H-^{13}C HSQC NMR spectrum of **6e** in C_6D_6 (298K).

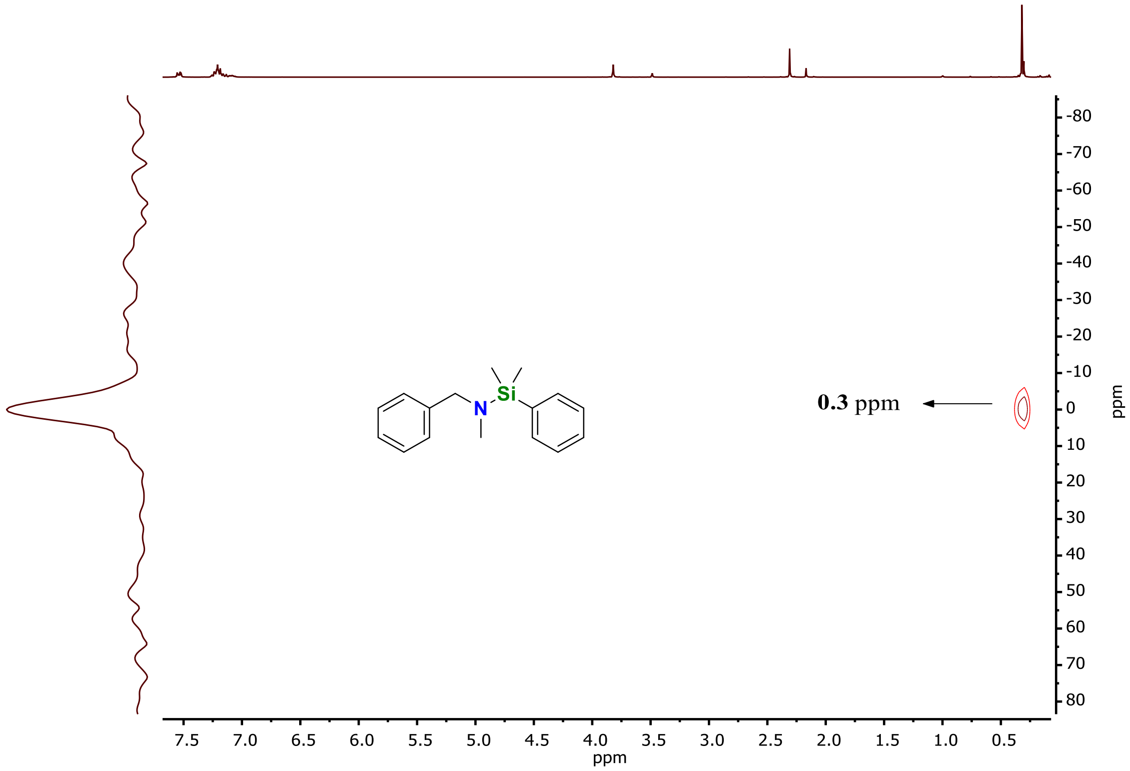

Figure S102. ^{1}H-^{29}Si HMQC NMR spectrum of **6e** in C_6D_6 (298K).

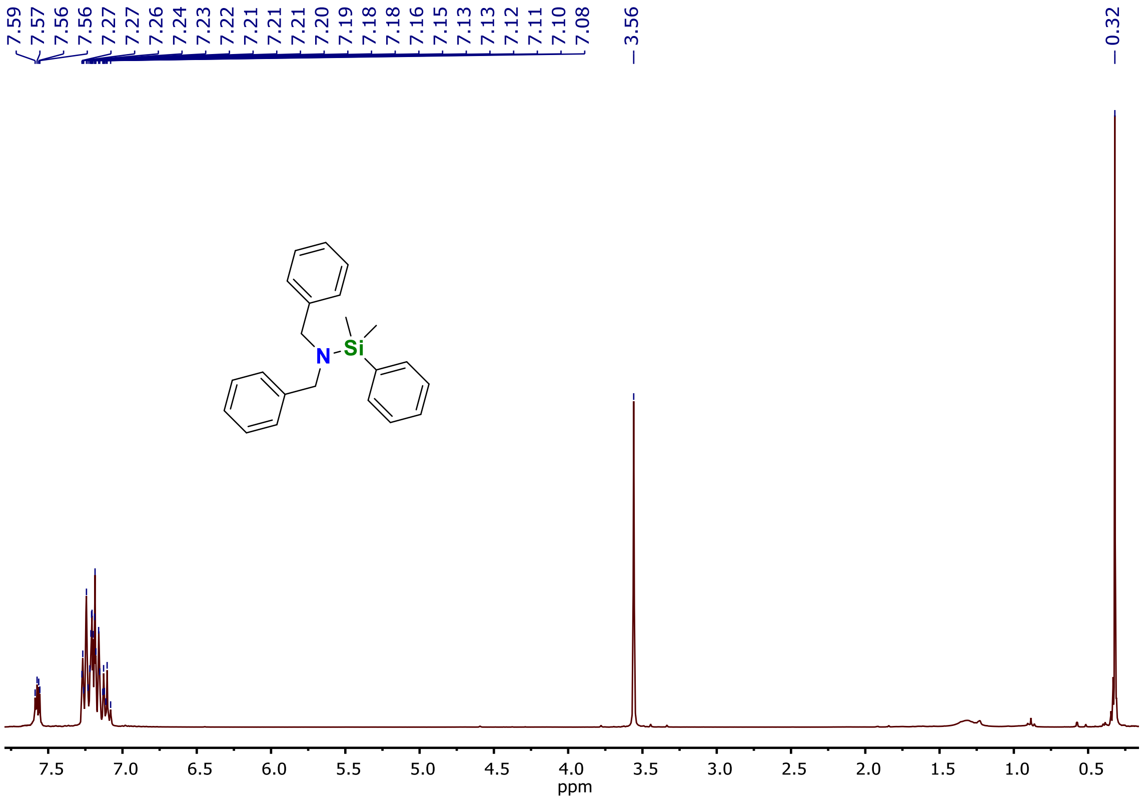

Figure S103. ^{1}H NMR spectrum of **6f** in C_6D_6 (300 MHz, 298K).

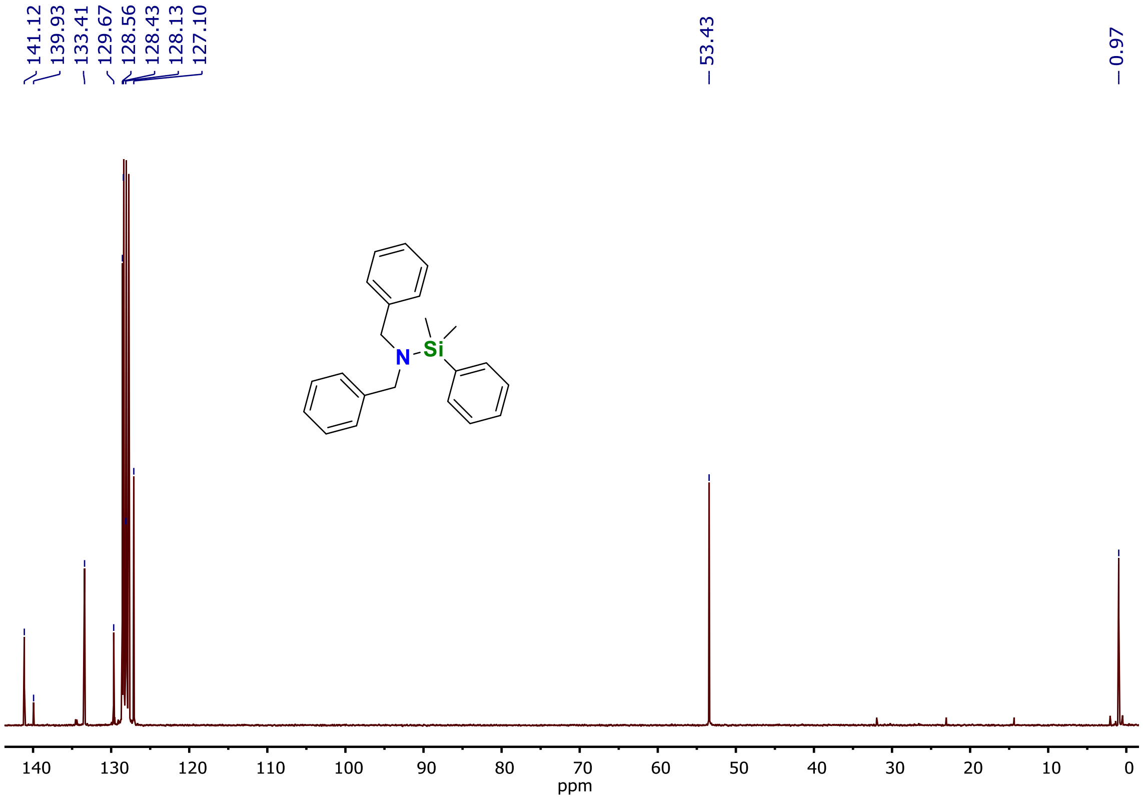

Figure S104. $^{13}C\{^{1}H\}$ NMR spectrum of **6f** in C_6D_6 (75 MHz, 298K).

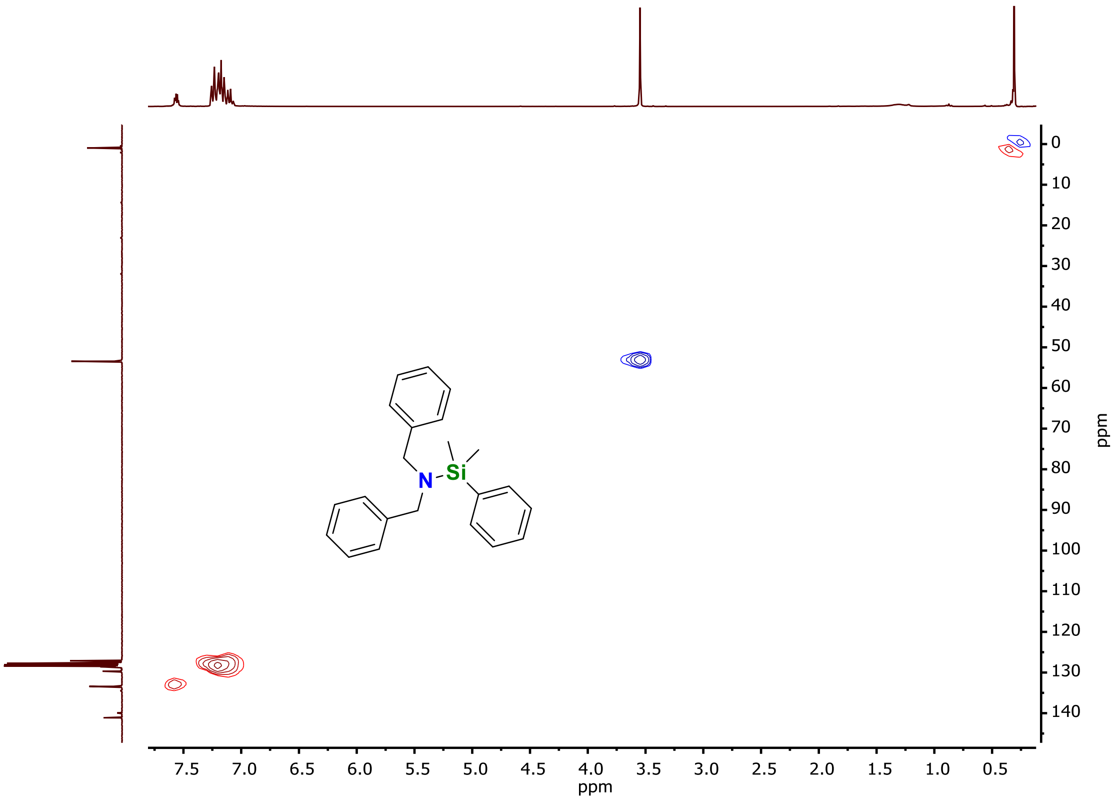

Figure S105. ^{1}H-^{13}C HSQC NMR spectrum of **6f** in C_6D_6 (298K).

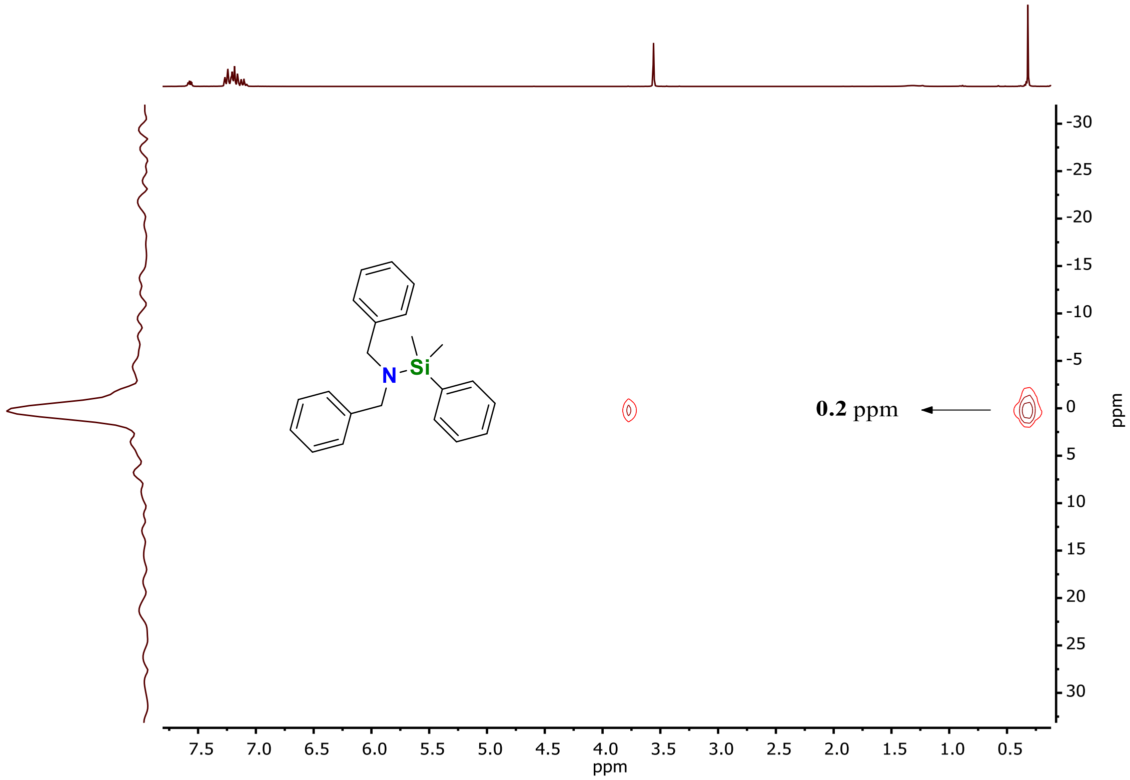

Figure S106. ^{1}H-^{29}Si HMQC NMR spectrum of **6f** in C_6D_6 (298K).

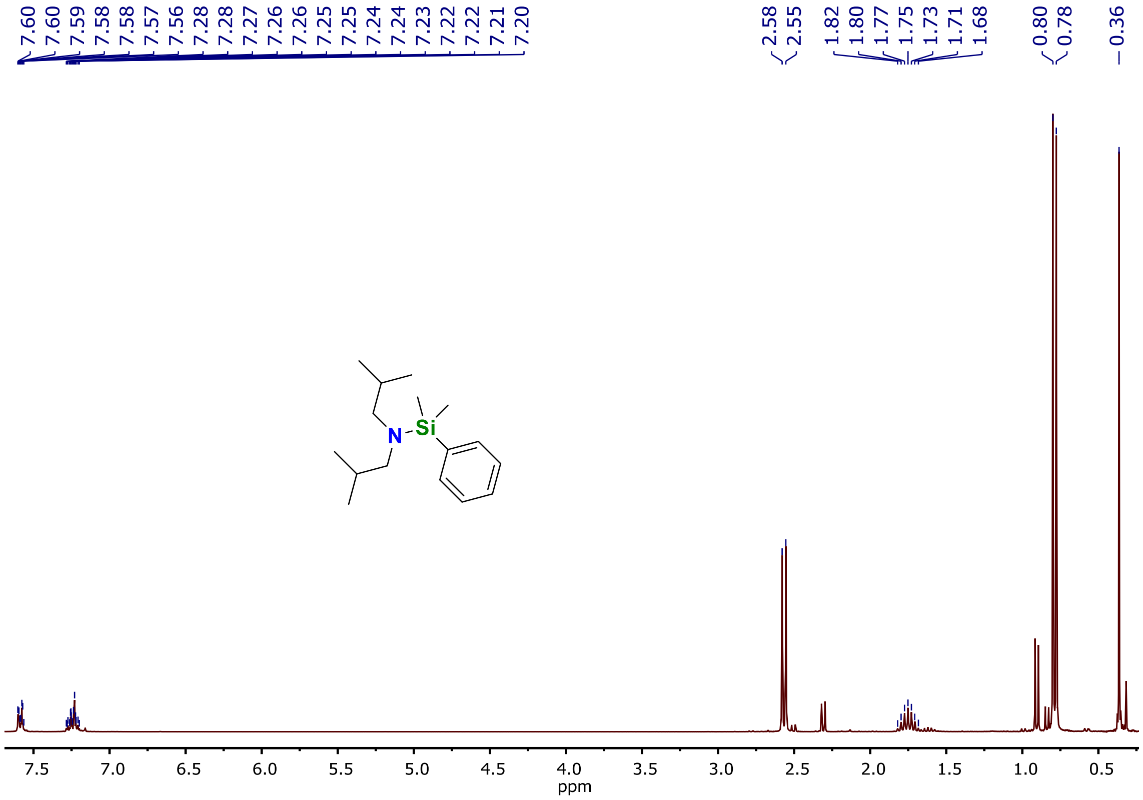

Figure S107. ^{1}H NMR spectrum of **6g** in C_6D_6 (300 MHz, 298K).

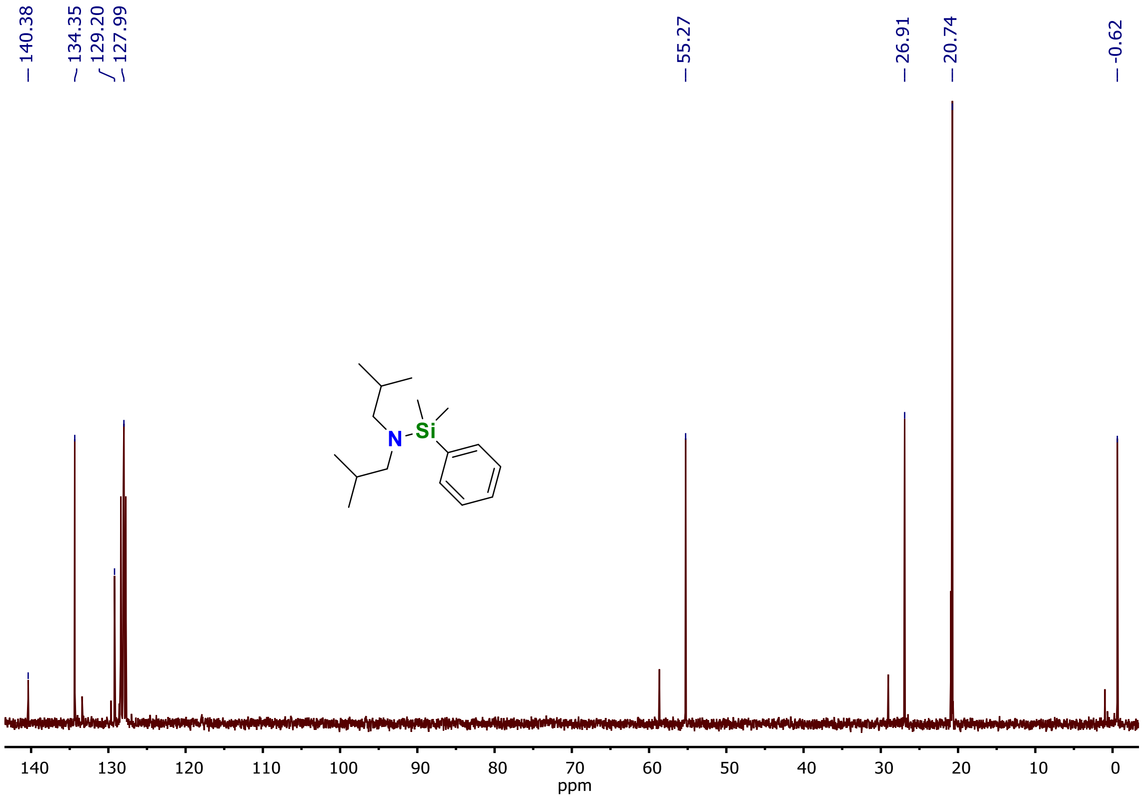

Figure S108. $^{13}C\{^{1}H\}$ NMR spectrum of **6g** in C_6D_6 (75 MHz, 298K).

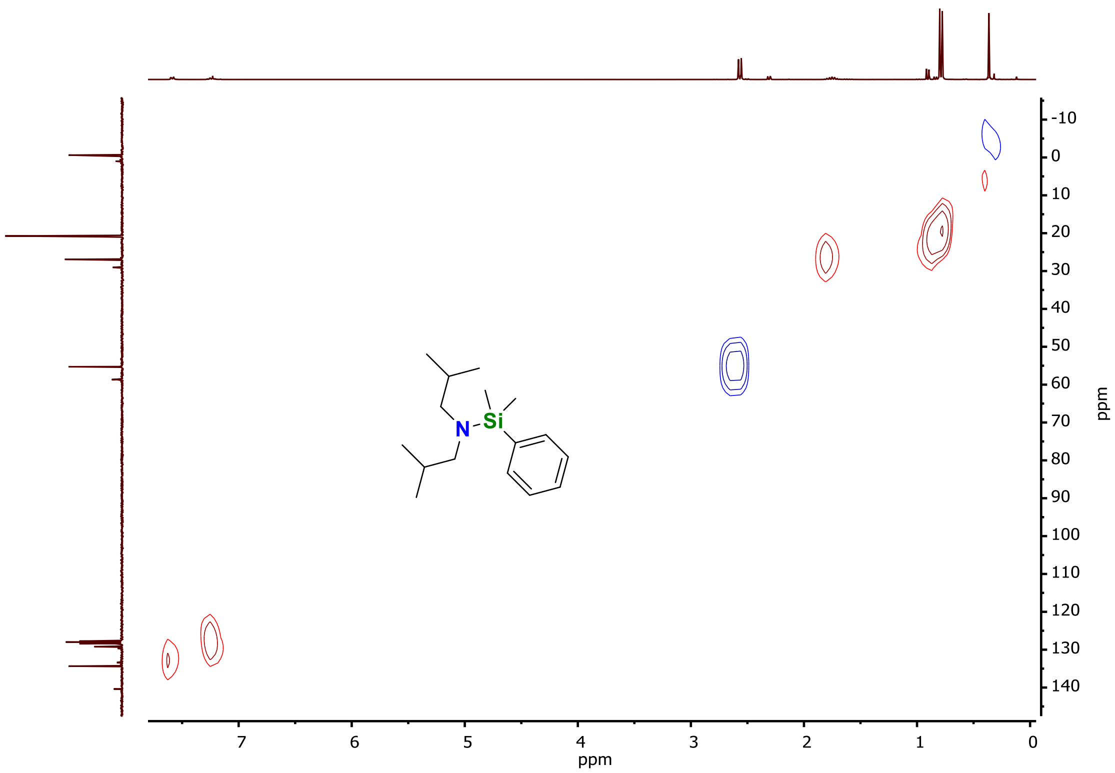

Figure S109. ^{1}H-^{13}C HSQC NMR spectrum of **6g** in C_6D_6 (298K).

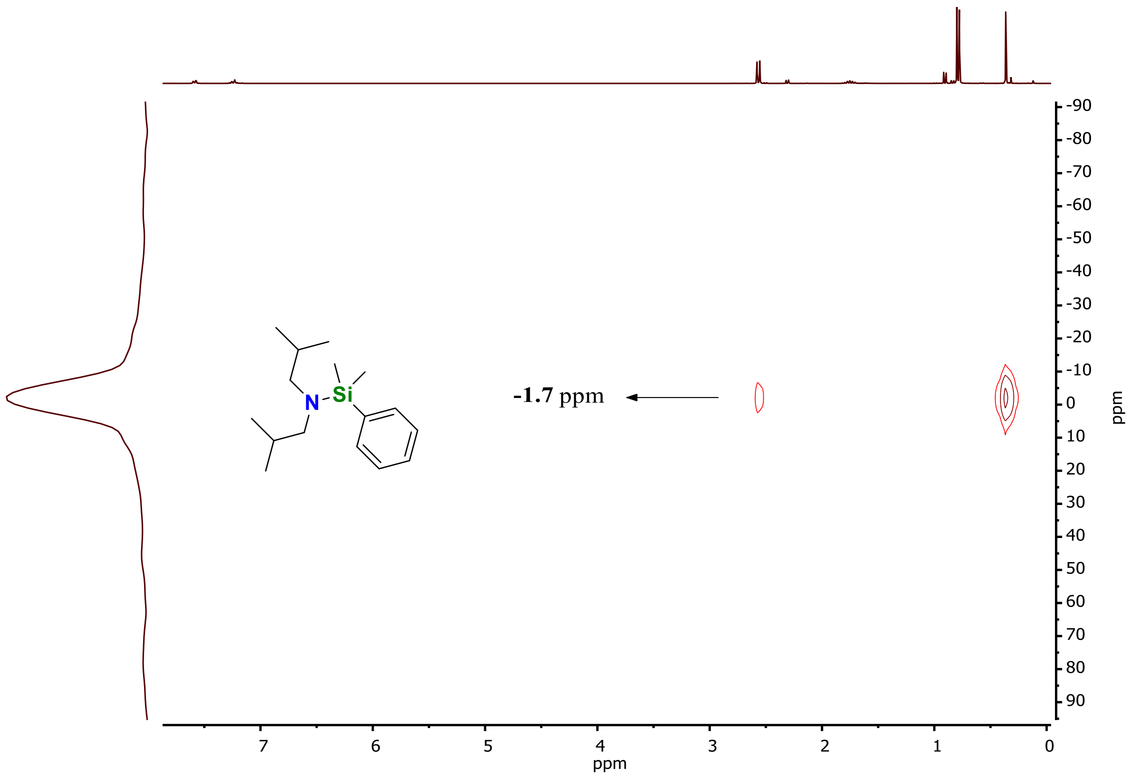

Figure S110. ^{1}H-^{29}Si HMQC NMR spectrum of **6g** in C_6D_6 (298K).

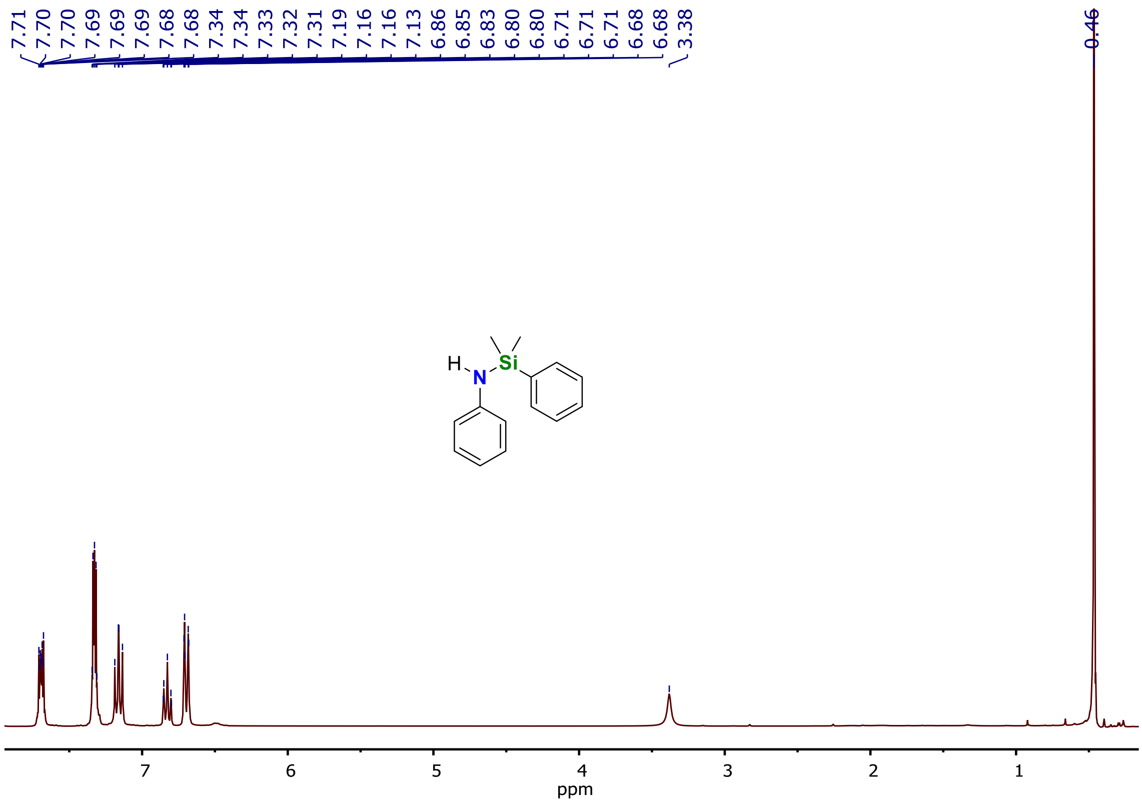

Figure S111. ^{1}H NMR spectrum of **7a** in C_6D_6 (300 MHz, 298K).

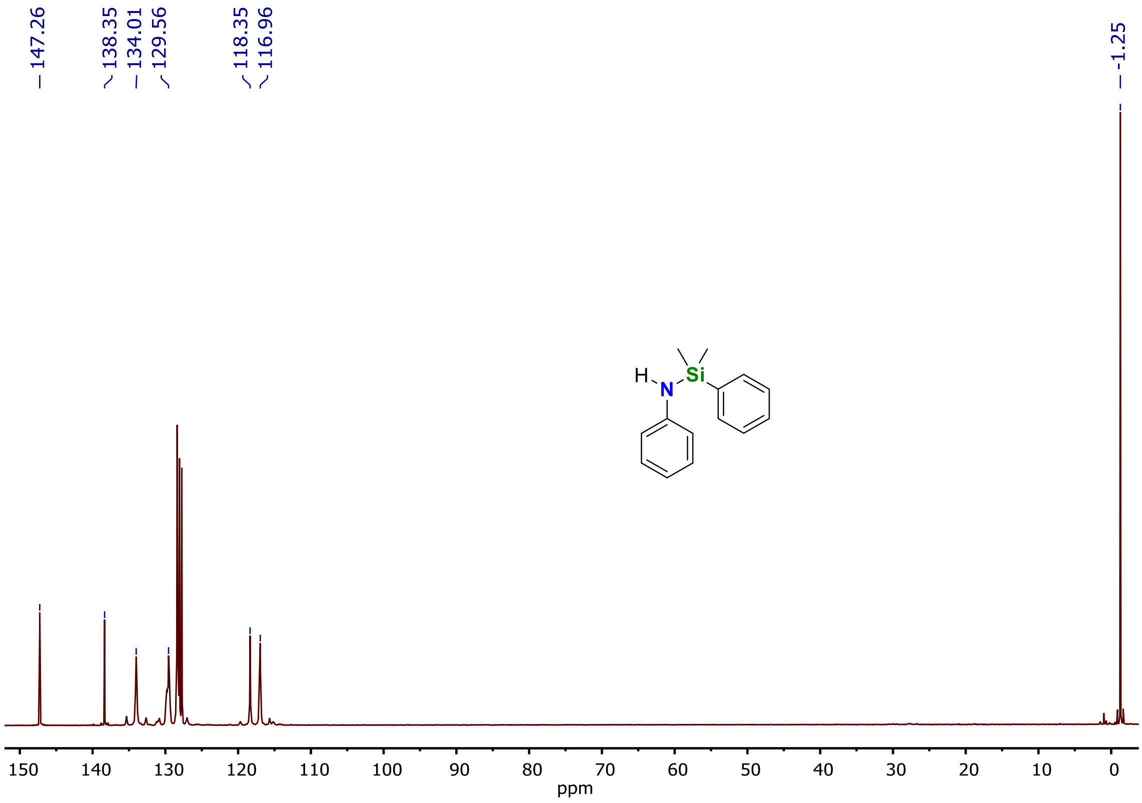

Figure S112. ^{13}C{^{1}H} NMR spectrum of **7a** in C_6D_6 (75 MHz, 298K).

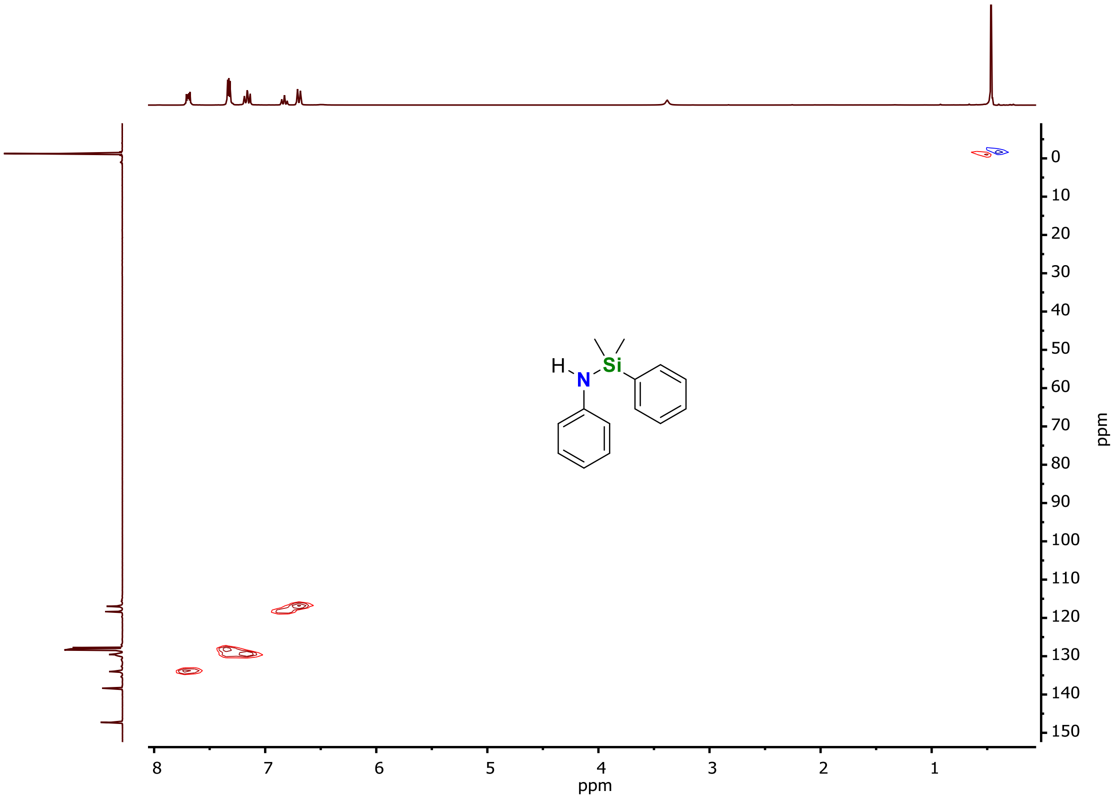

Figure S113. ^{1}H-^{13}C HSQC NMR spectrum of **7a** in C_6D_6 (298K).

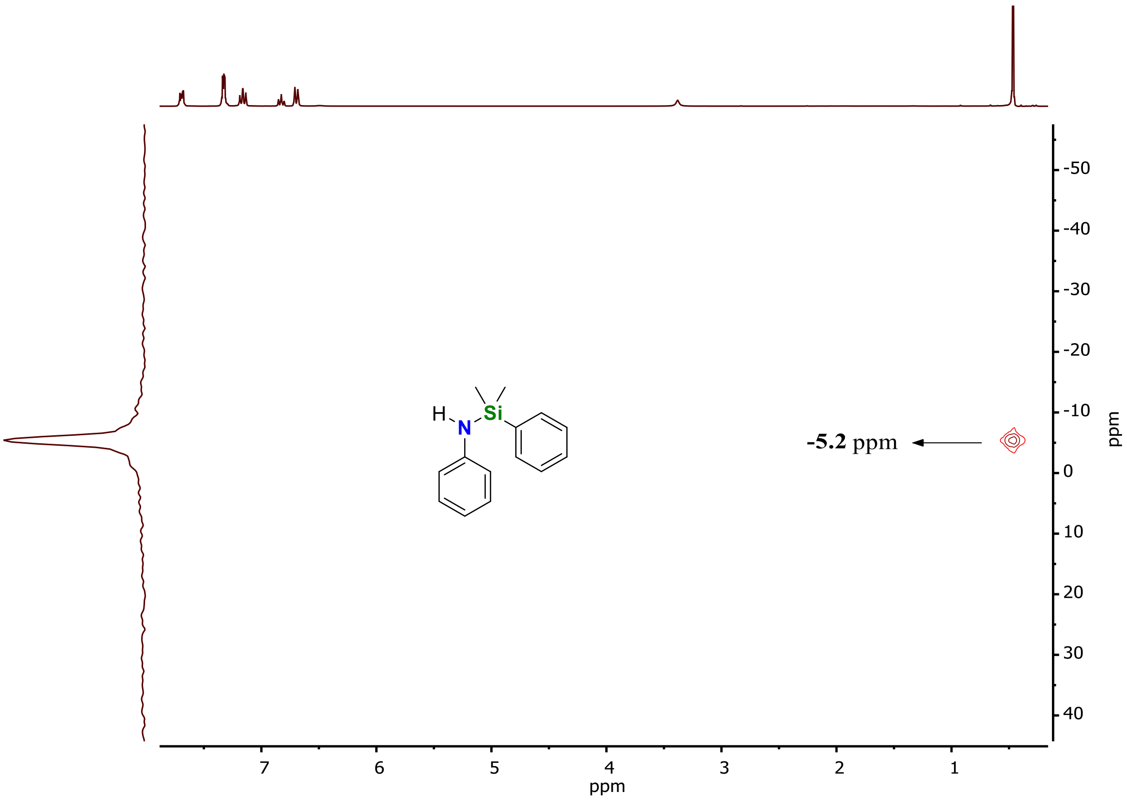

Figure S114. ^{1}H-^{29}Si HMQC NMR spectrum of **7a** in C_6D_6 (298K).

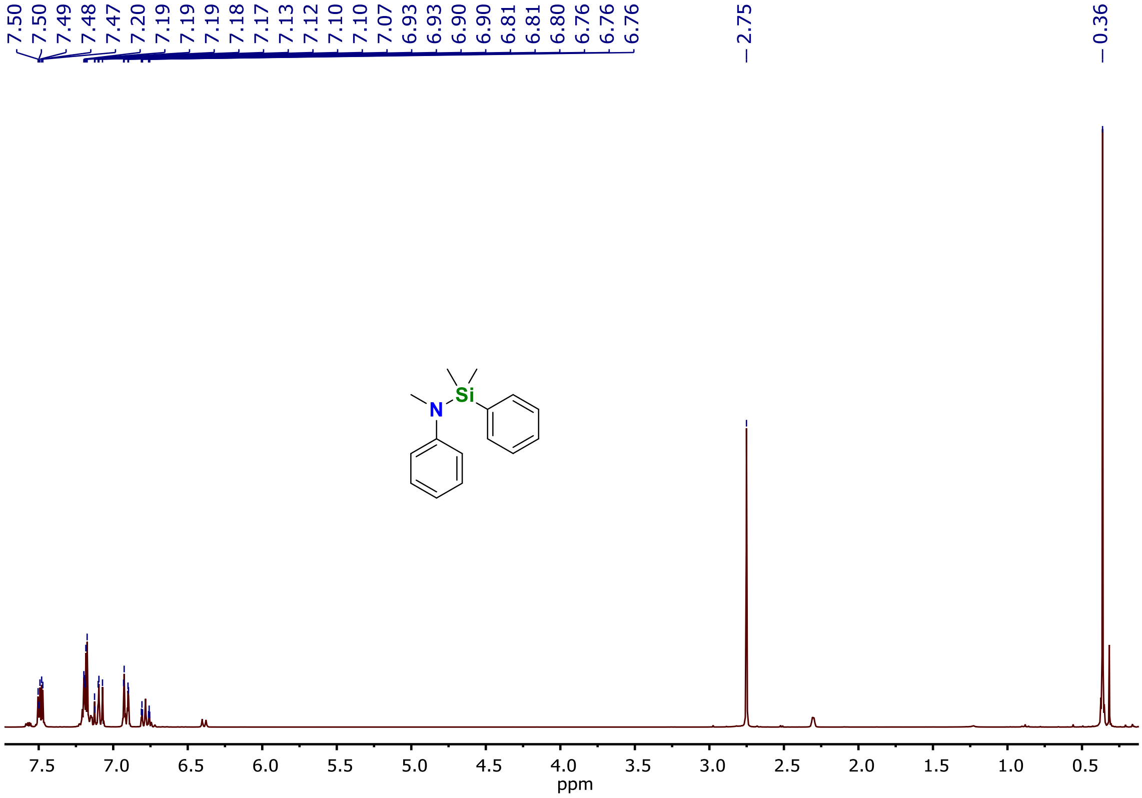

Figure S115. ^{1}H NMR spectrum of **7b** in C_6D_6 (300 MHz, 298K).

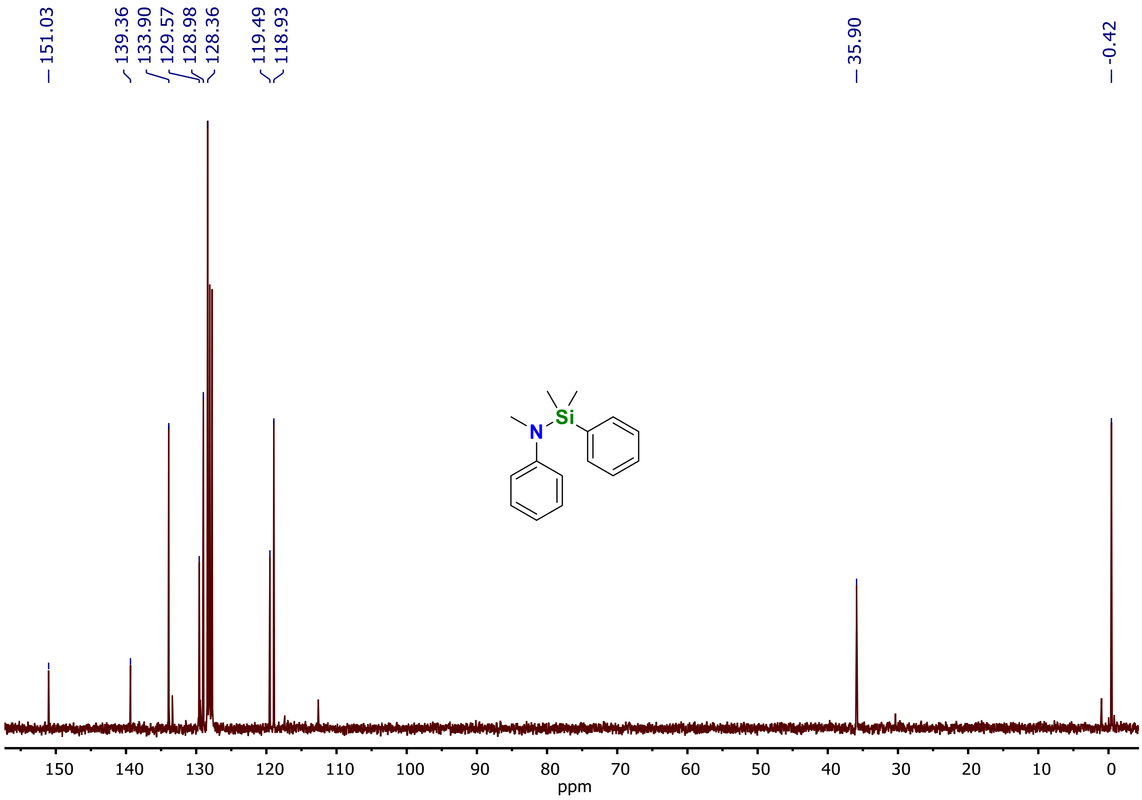

Figure S116. $^{13}C\{^{1}H\}$ NMR spectrum of **7b** in C_6D_6 (75 MHz, 298K).

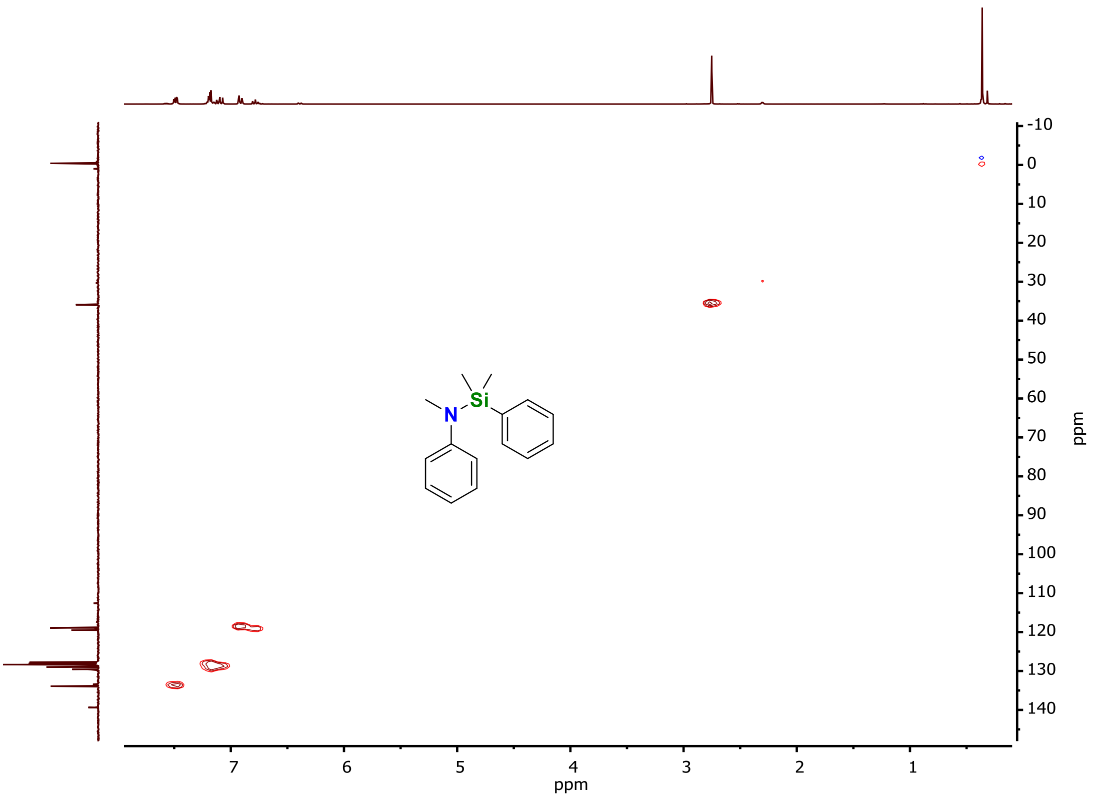

Figure S117. ^{1}H-^{13}C HSQC NMR spectrum of **7b** in C_6D_6 (298K).

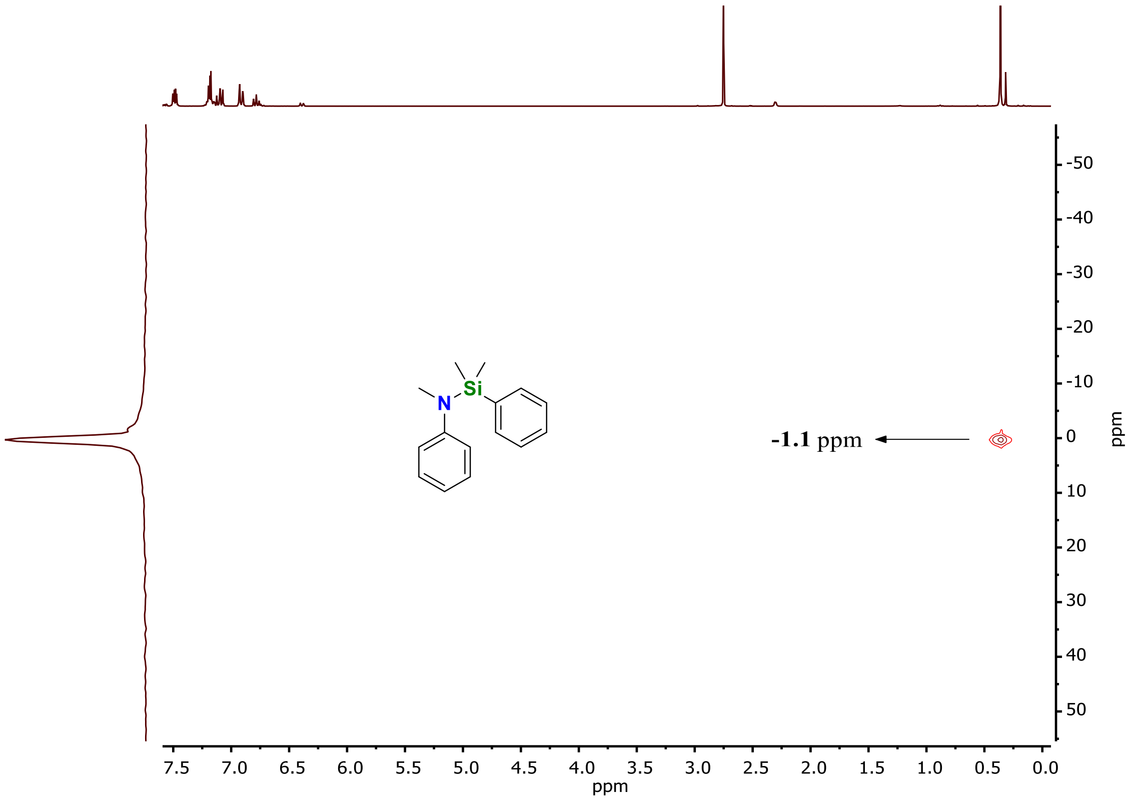

Figure S118. ^{1}H-^{29}Si HMQC NMR spectrum of **7b** in C_6D_6 (298K).

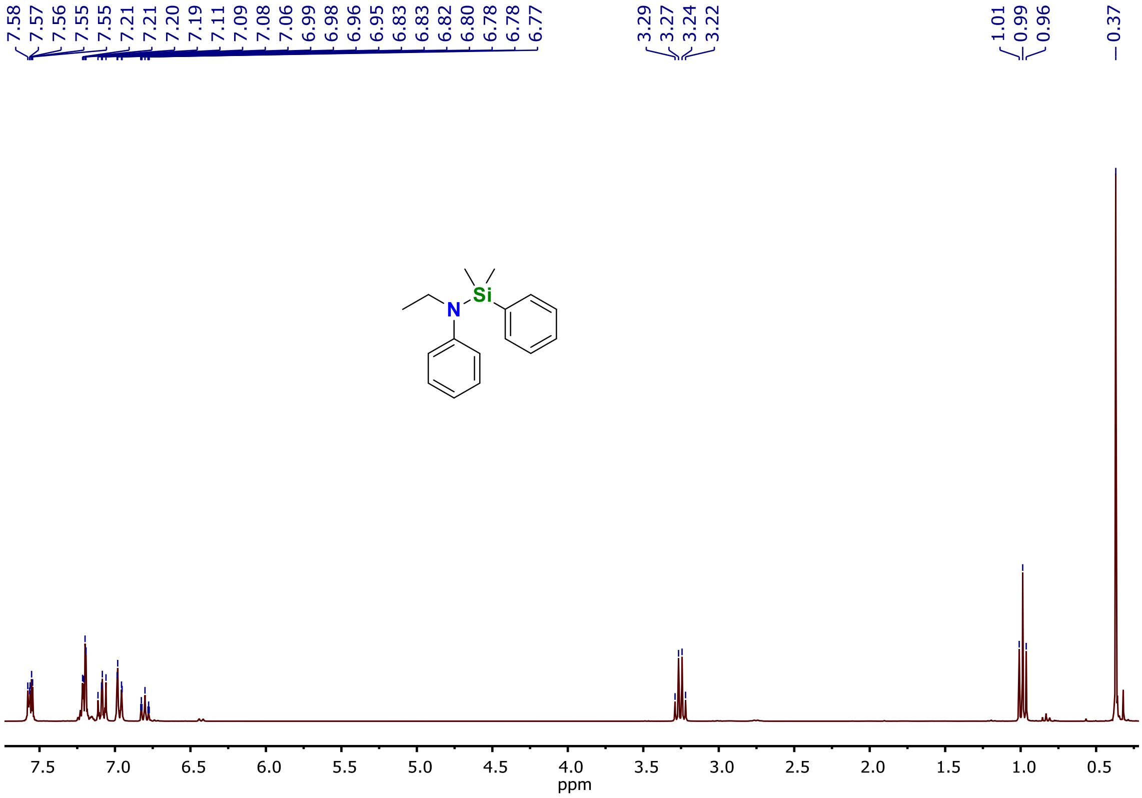

Figure S119. ^{1}H NMR spectrum of **7c** in C_6D_6 (300 MHz, 298K).

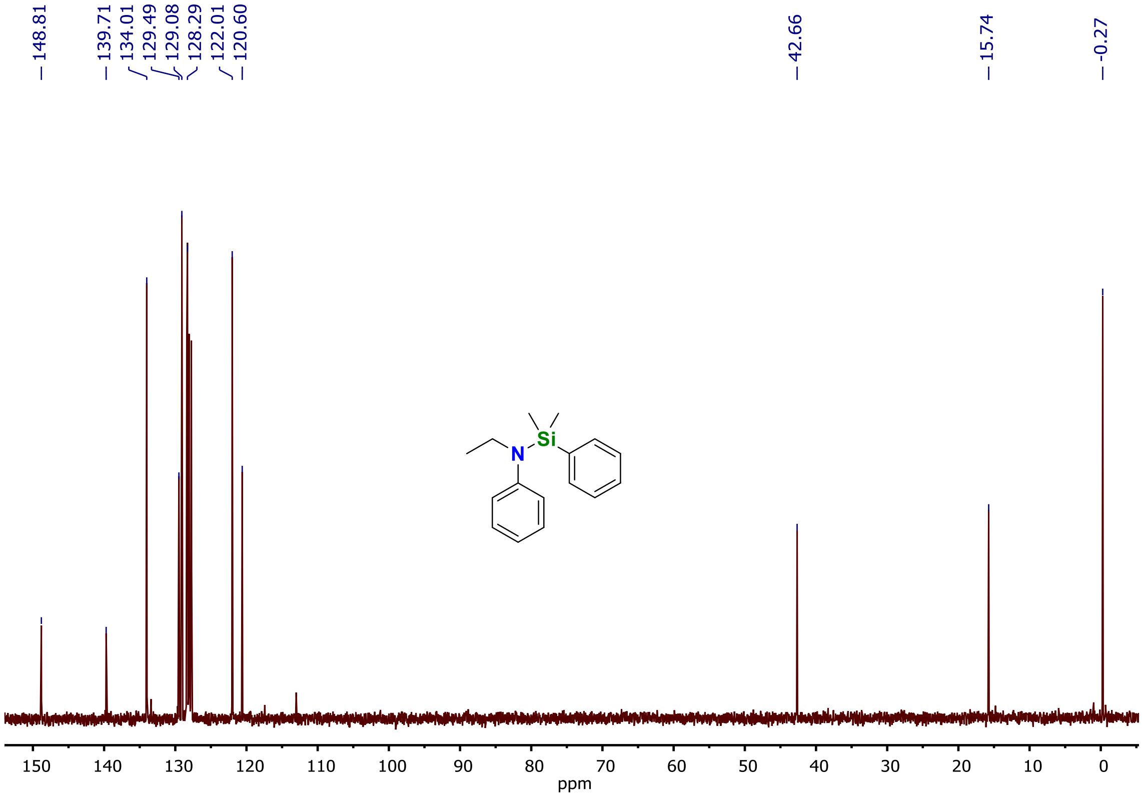

Figure S120. ^{13}C{^{1}H} NMR spectrum of **7c** in C_6D_6 (75 MHz, 298K).

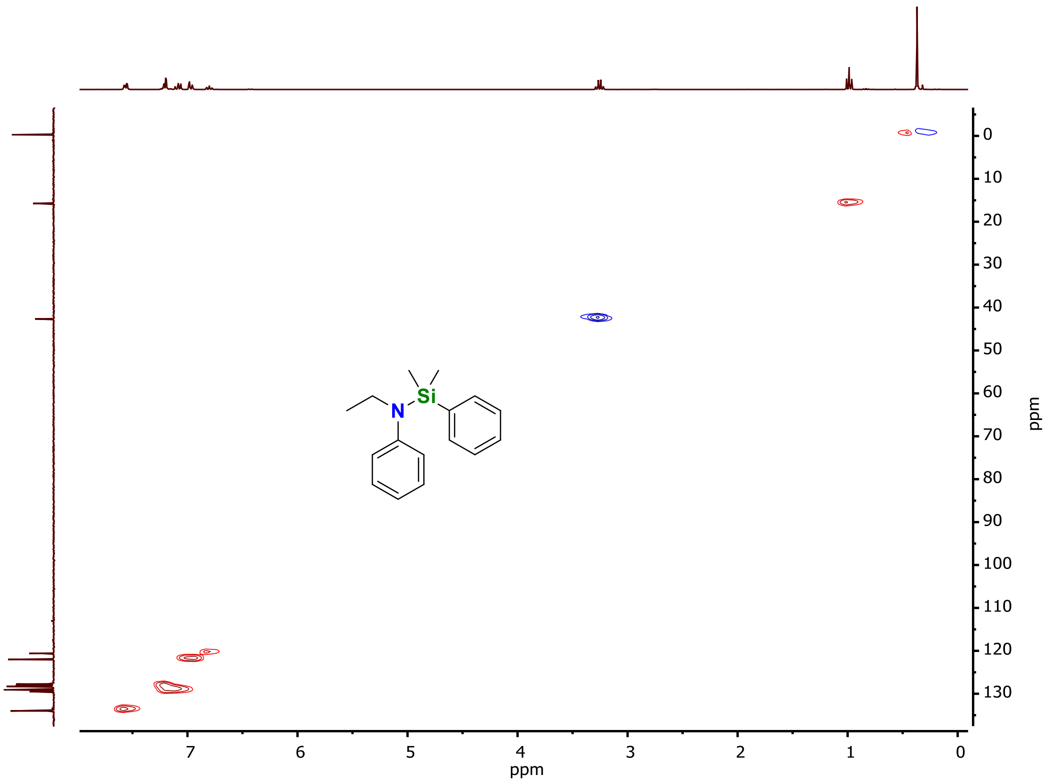

Figure S121. ^{1}H-^{13}C HSQC NMR spectrum of **7c** in C_6D_6 (298K).

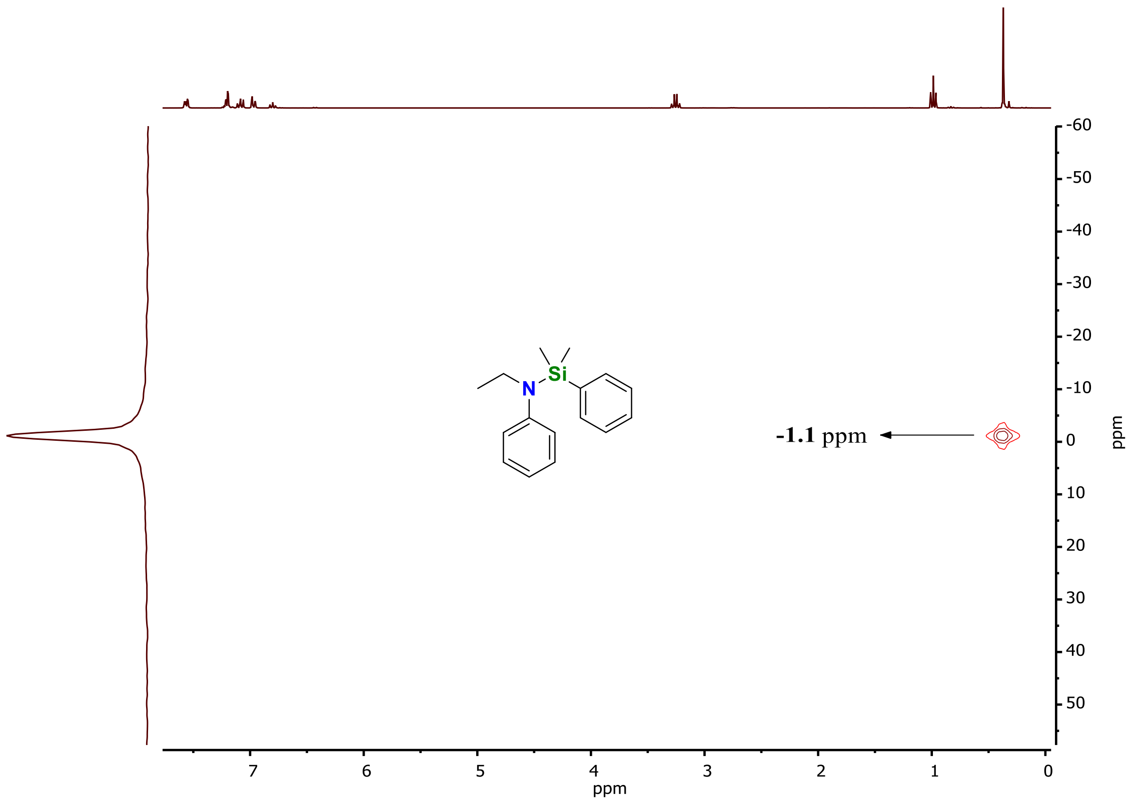

Figure S122. ^{1}H-^{29}Si HMQC NMR spectrum of **7c** in C_6D_6 (298K).

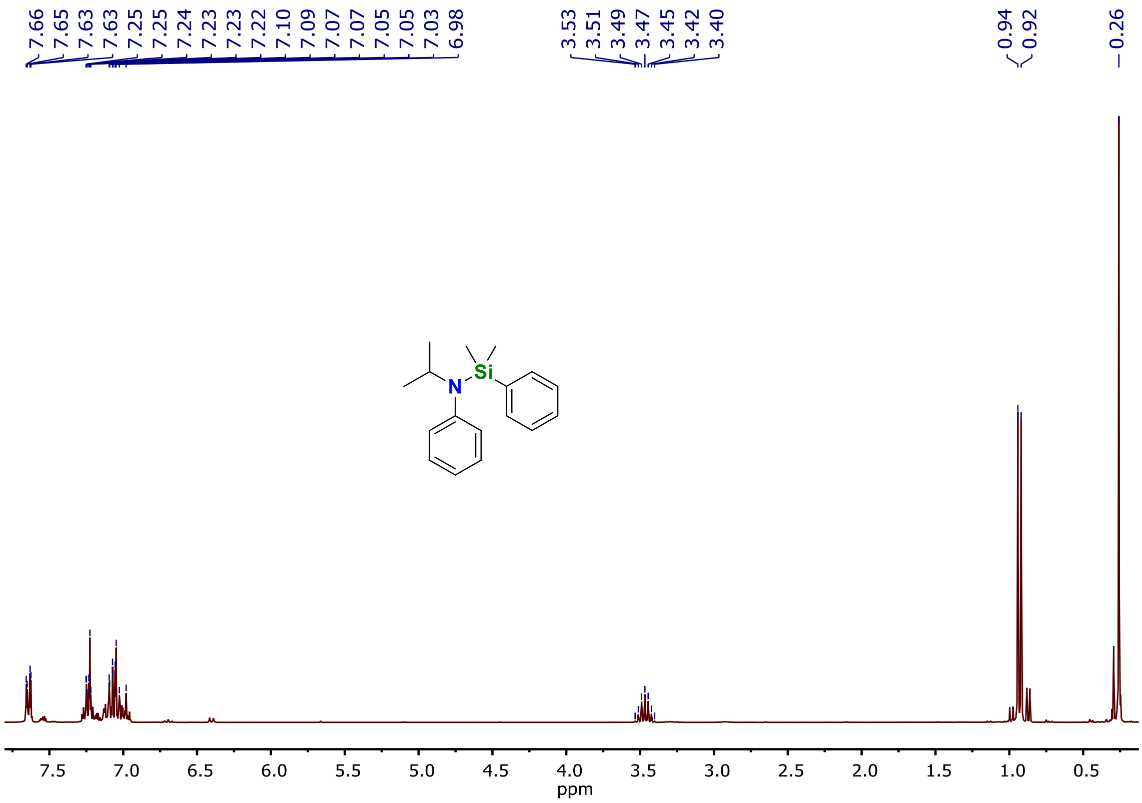

Figure S123. ^{1}H NMR spectrum of **7d** in C_6D_6 (400 MHz, 298K).

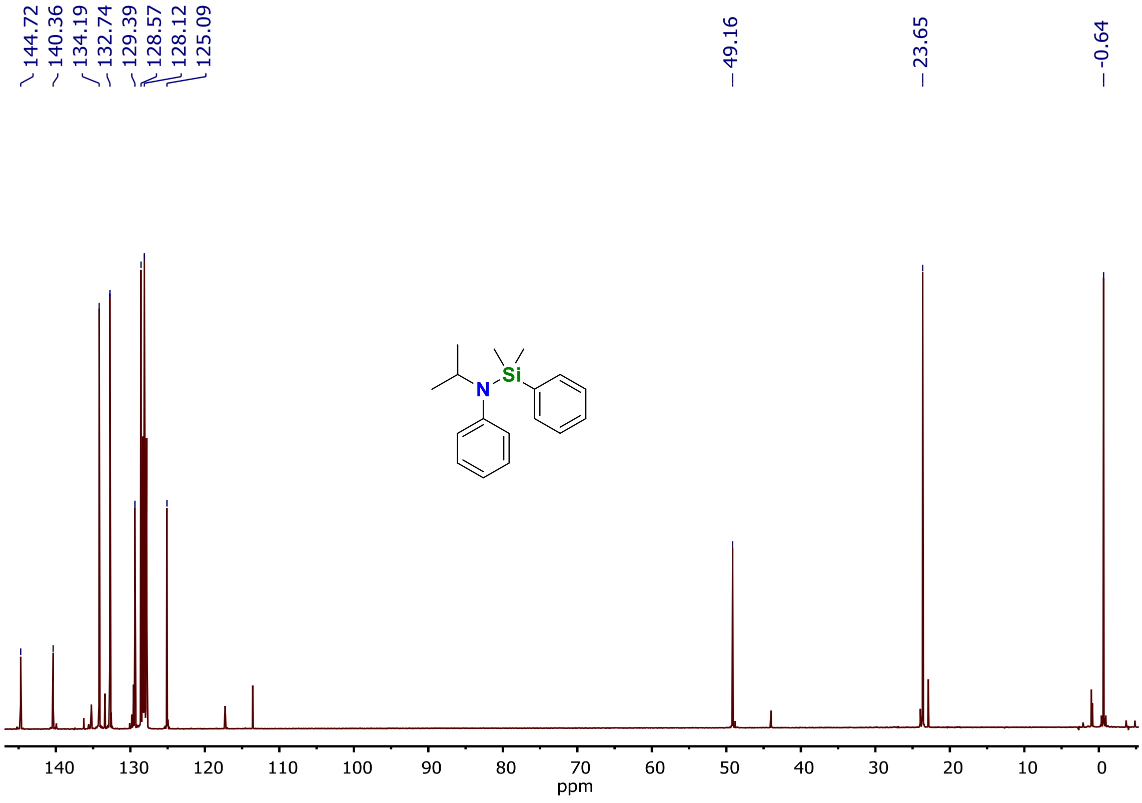

Figure S124. ^{13}C{^{1}H} NMR spectrum of **7d** in C_6D_6 (75 MHz, 298K).

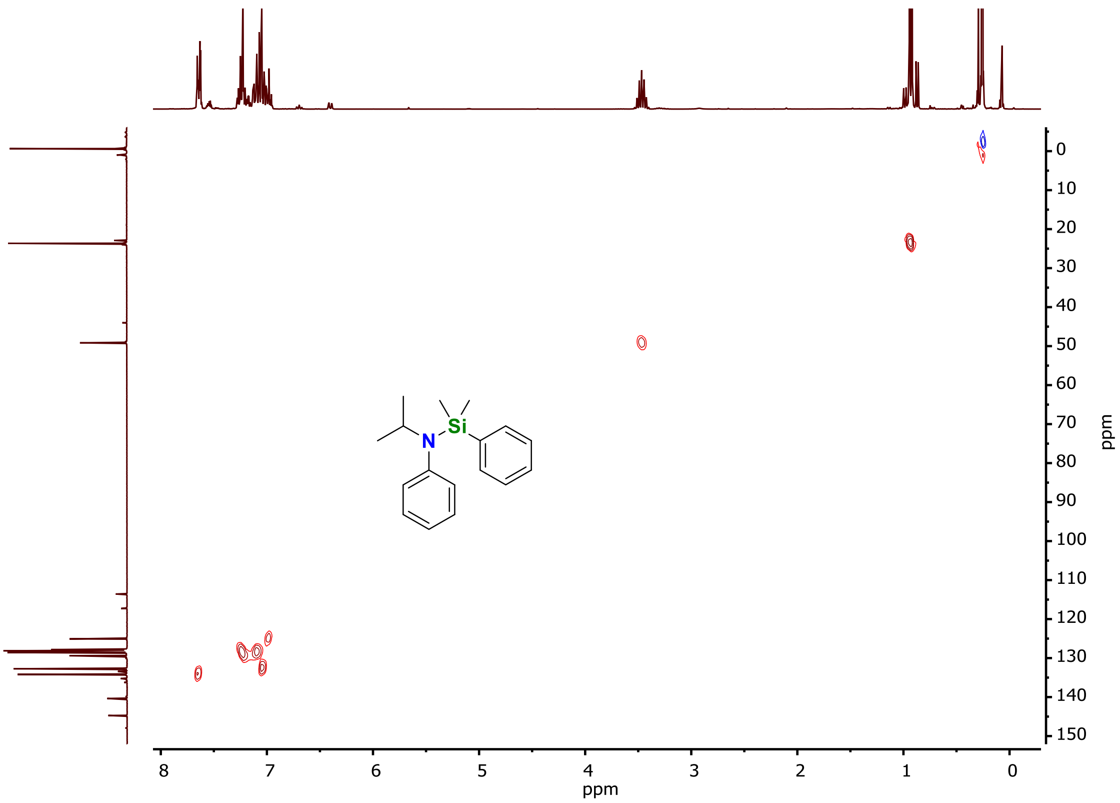

Figure S125. ^{1}H-^{13}C HSQC NMR spectrum of **7d** in C_6D_6 (298K).

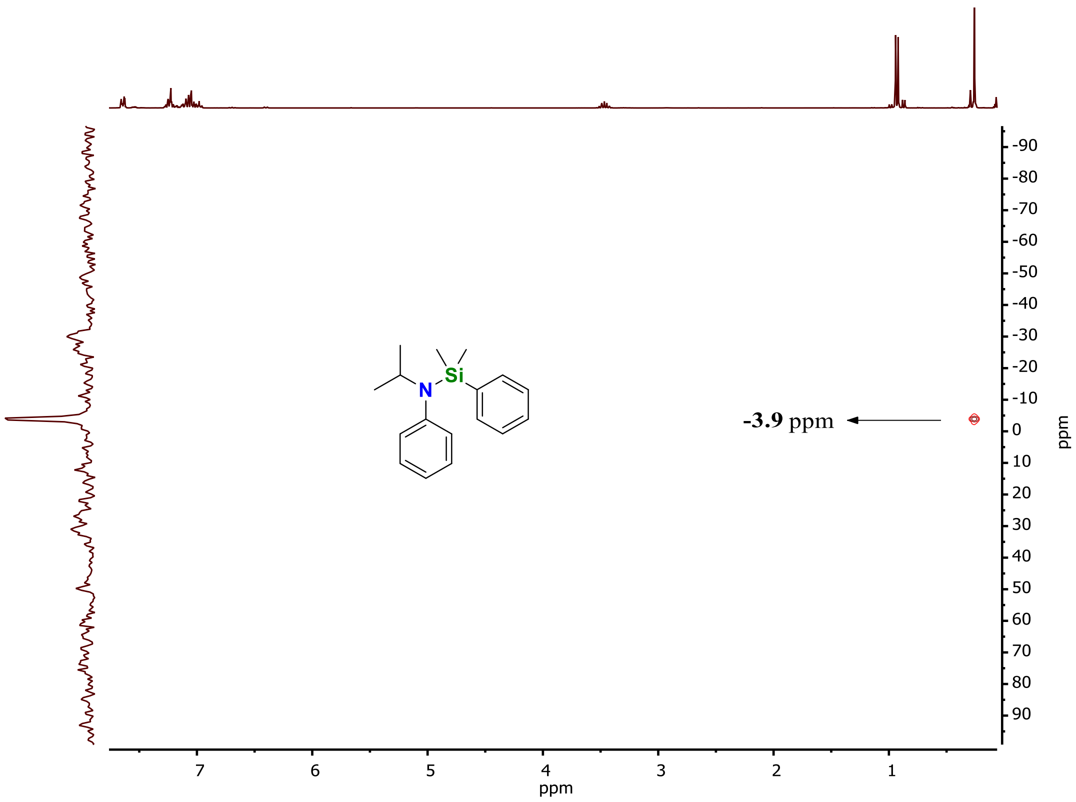

Figure S126. ^{1}H-^{29}Si HMQC NMR spectrum of **7d** in C_6D_6 (298K).

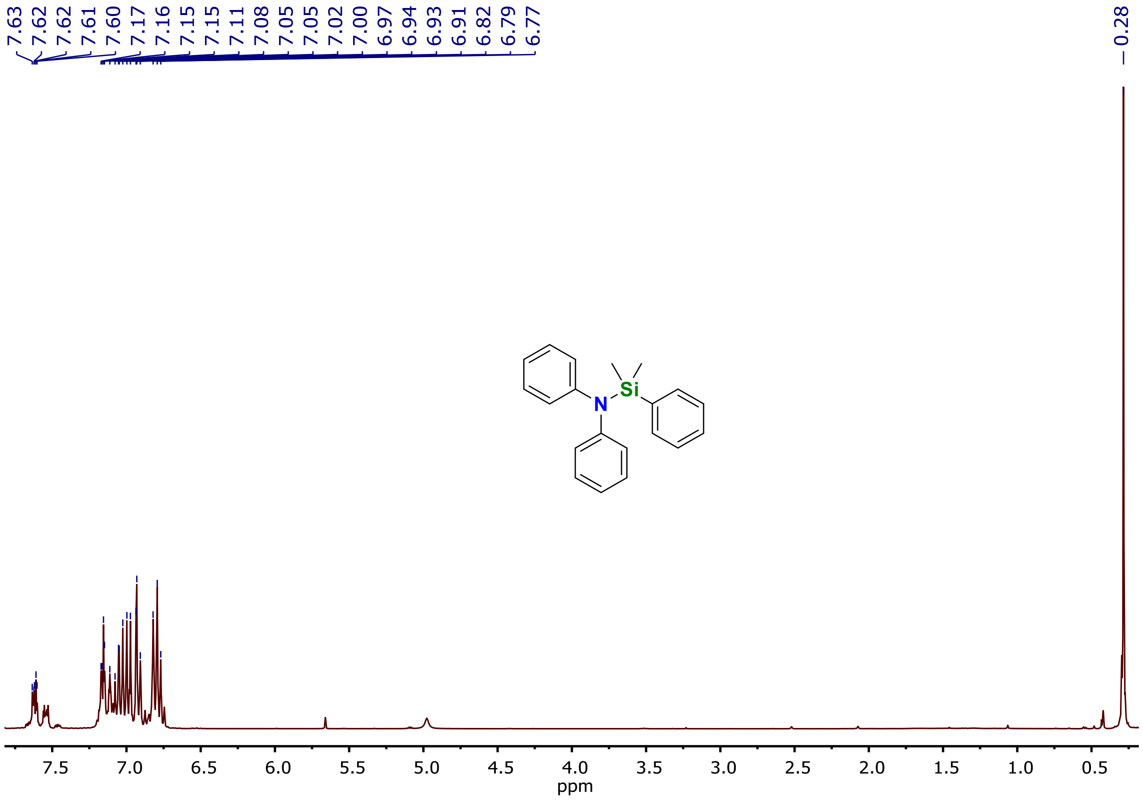

Figure S127. ^{1}H NMR spectrum of **7e** in C_6D_6 (400 MHz, 298K).

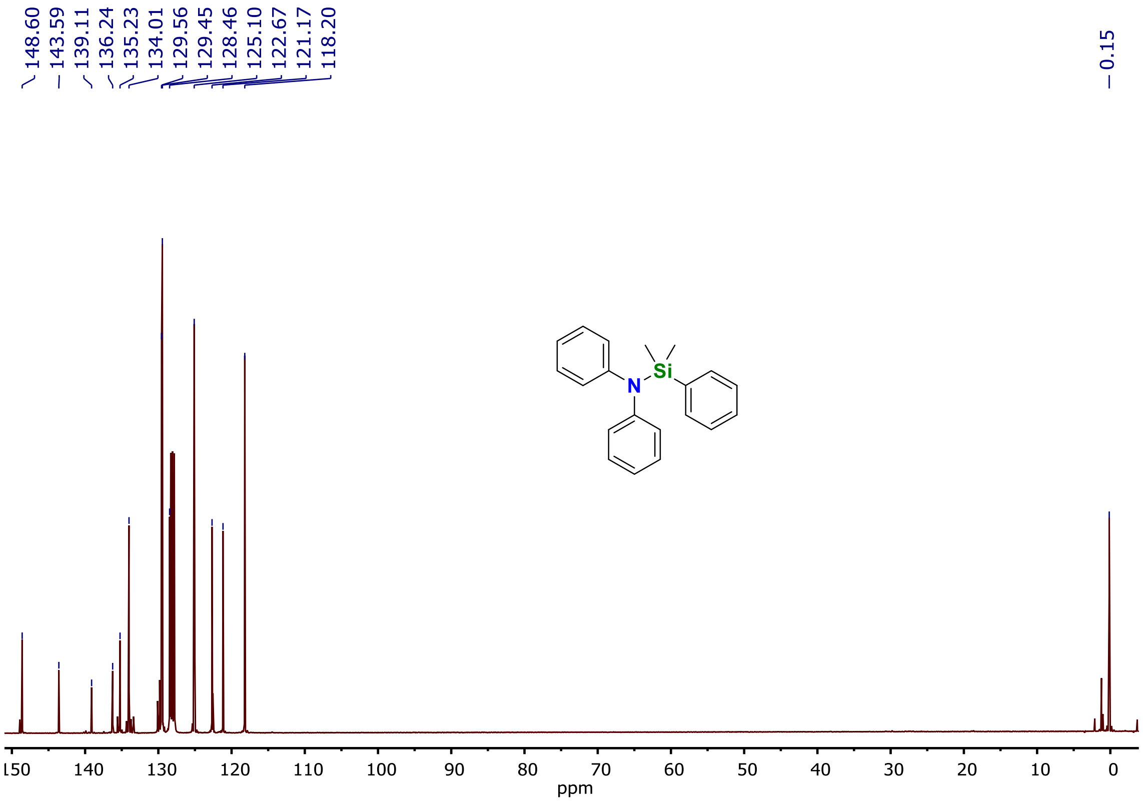

Figure S128. $^{13}C\{^{1}H\}$ NMR spectrum of **7e** in C_6D_6 (75 MHz, 298K).

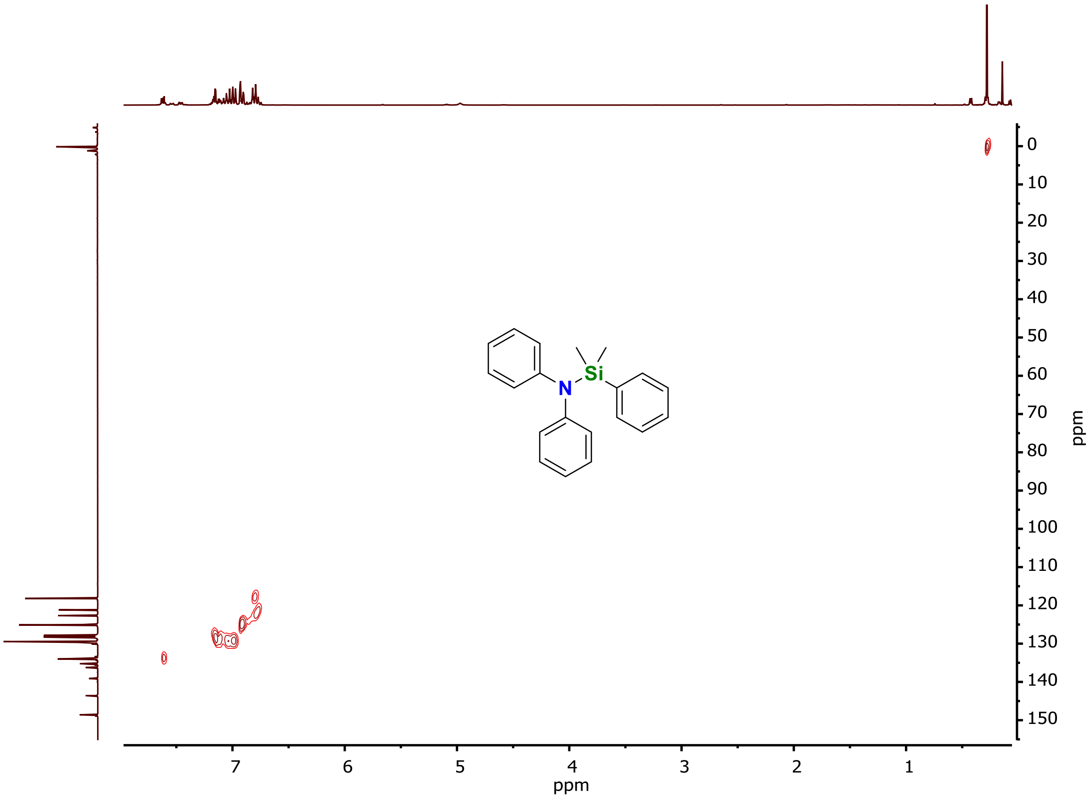

Figure S129. ^{1}H-^{13}C HSQC NMR spectrum of **7e** in C_6D_6 (298K).

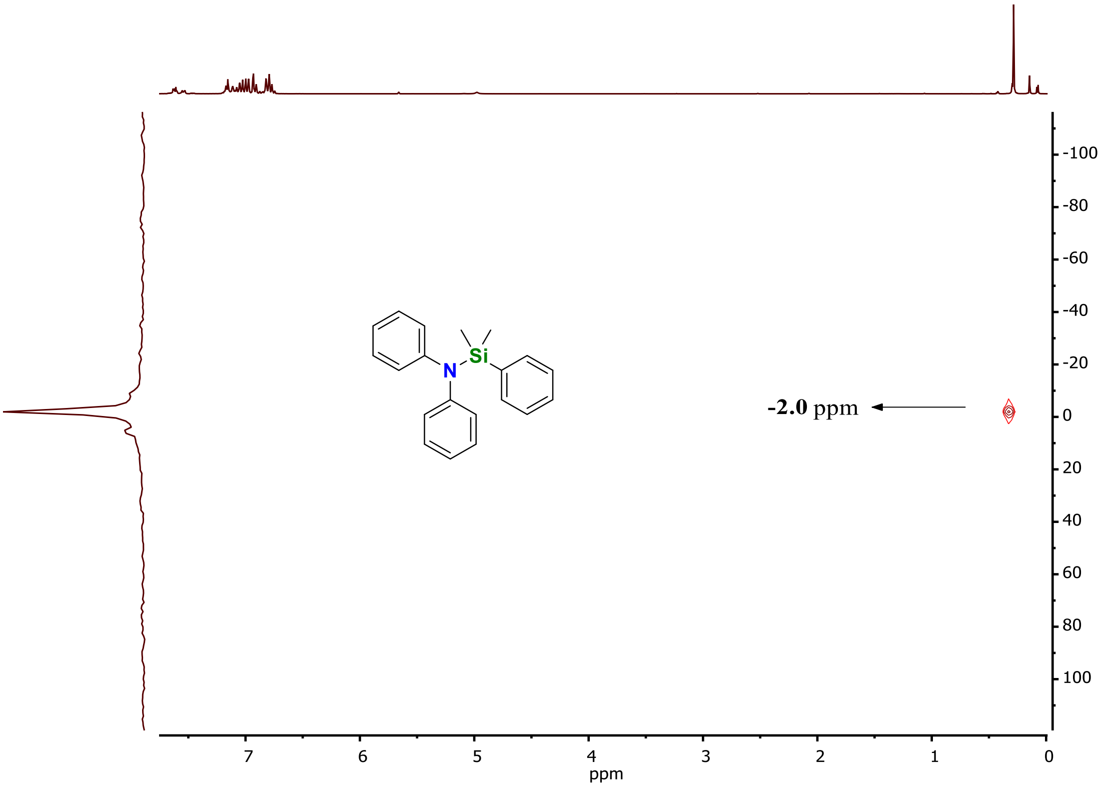

Figure S130. ^{1}H-^{29}Si HMQC NMR spectrum of **7e** in C_6D_6 (298K).

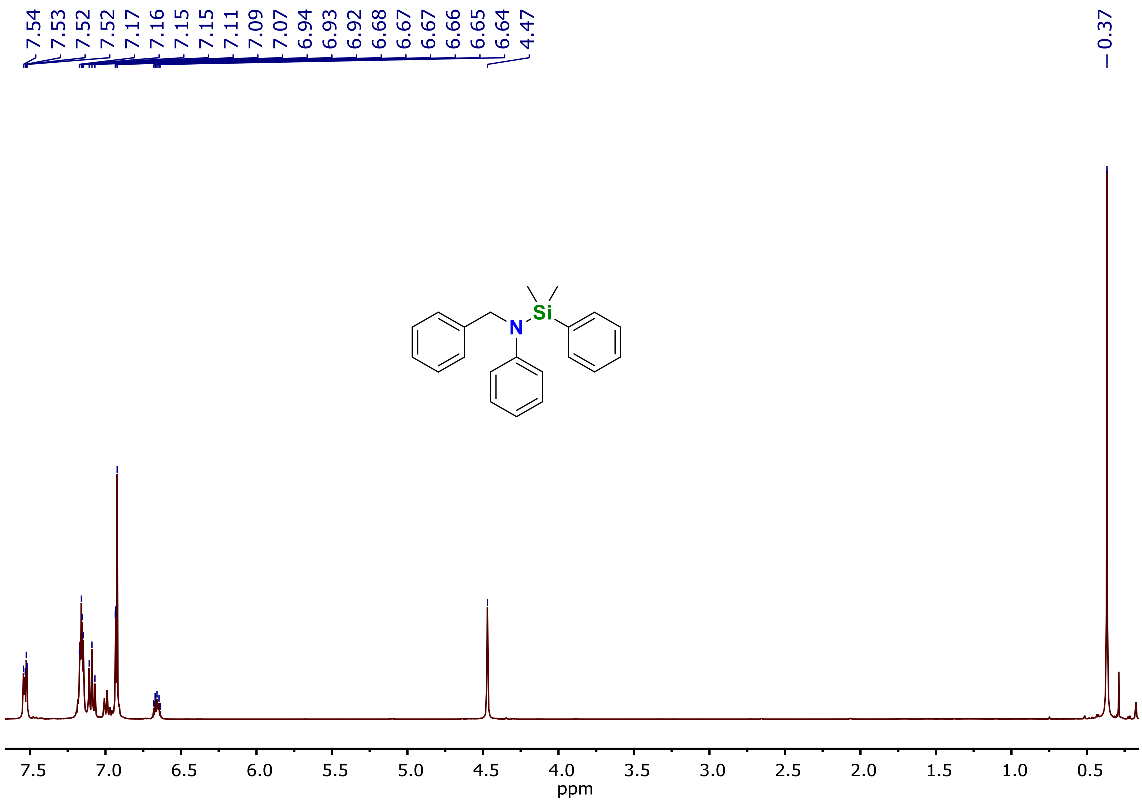

Figure S131. ^{1}H NMR spectrum of **7f** in C_6D_6 (400 MHz, 298K).

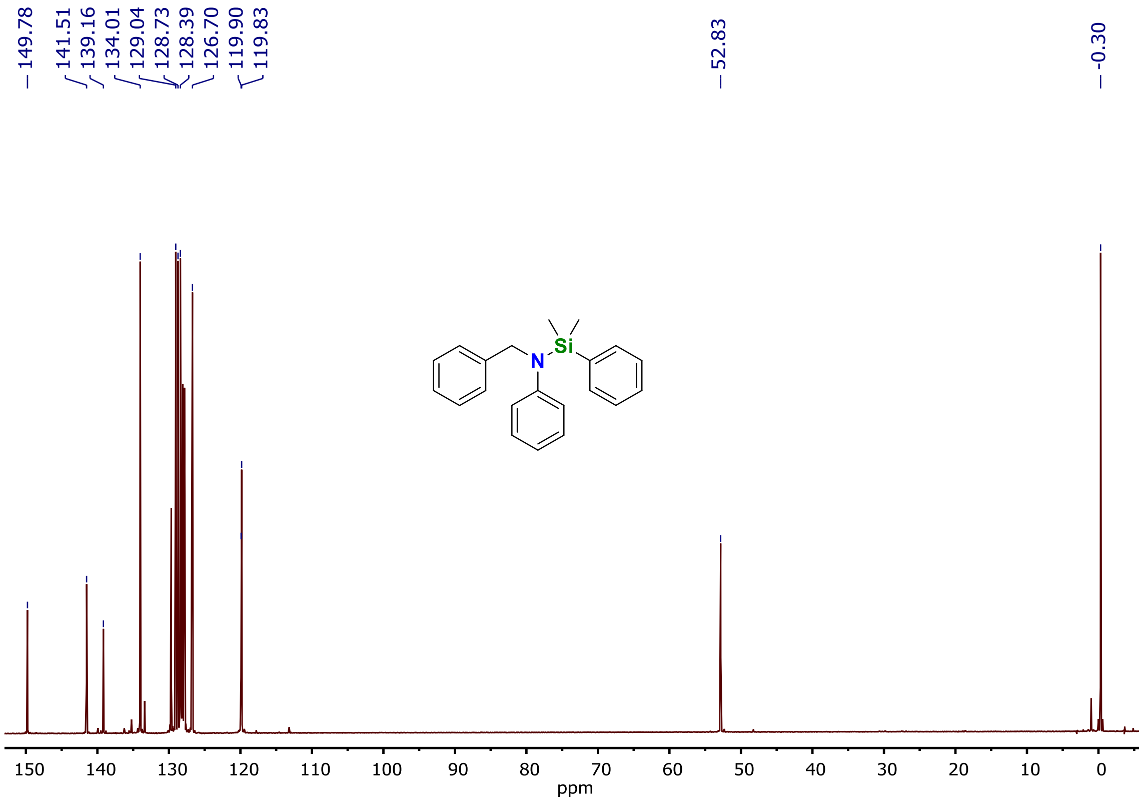

Figure S132. $^{13}C\{^{1}H\}$ NMR spectrum of **7f** in C_6D_6 (75 MHz, 298K).

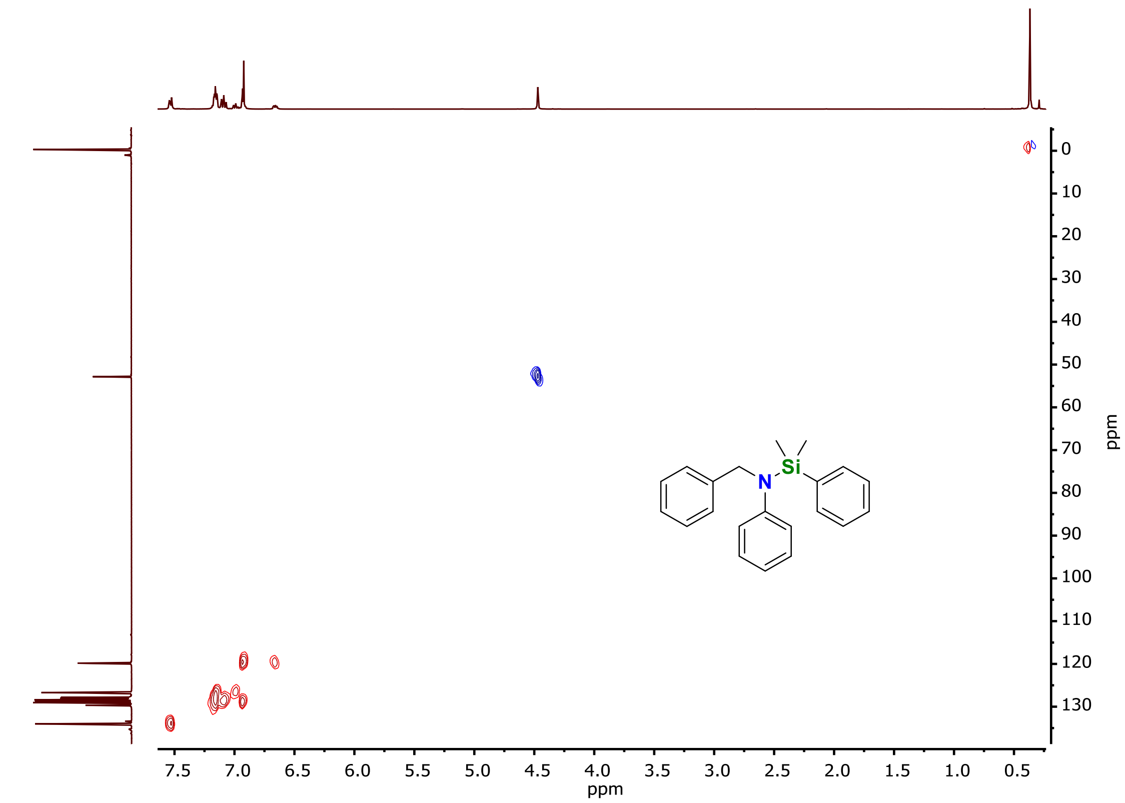

Figure S133. ^{1}H-^{13}C HSQC NMR spectrum of **7f** in C_6D_6 (298K).

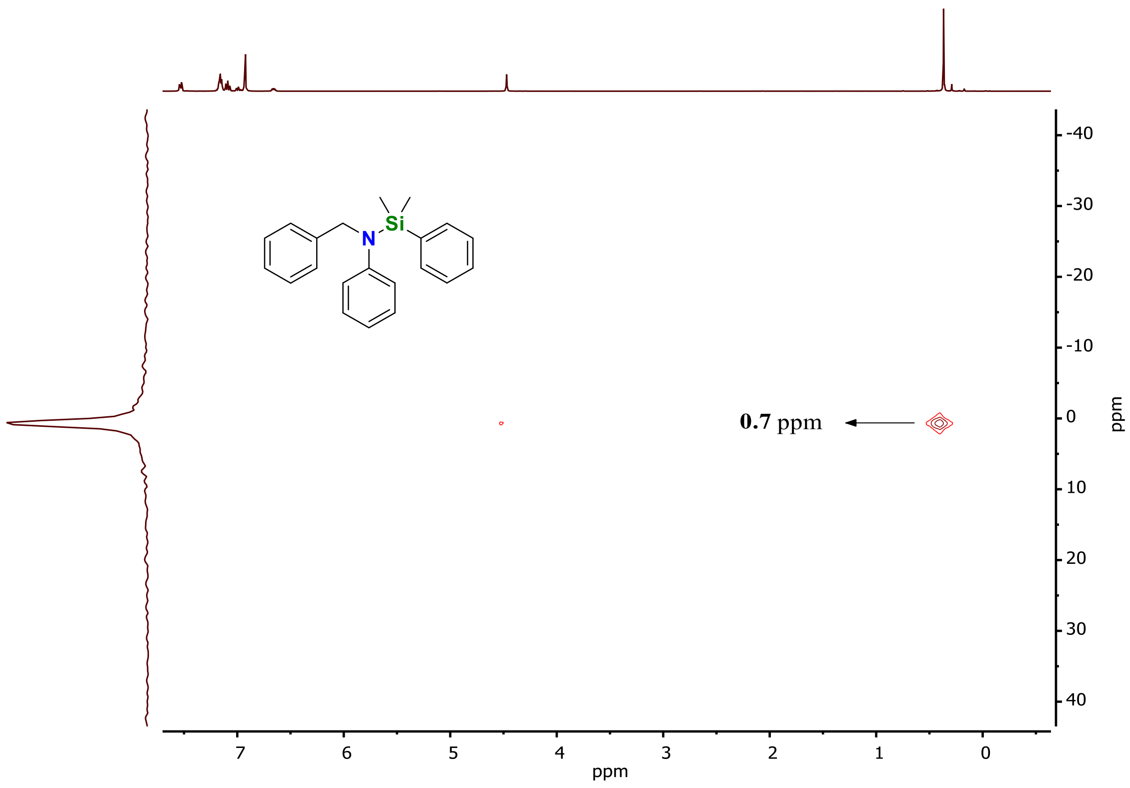

Figure S134. ^{1}H-^{29}Si HMQC NMR spectrum of **7f** in C_6D_6 (298K).

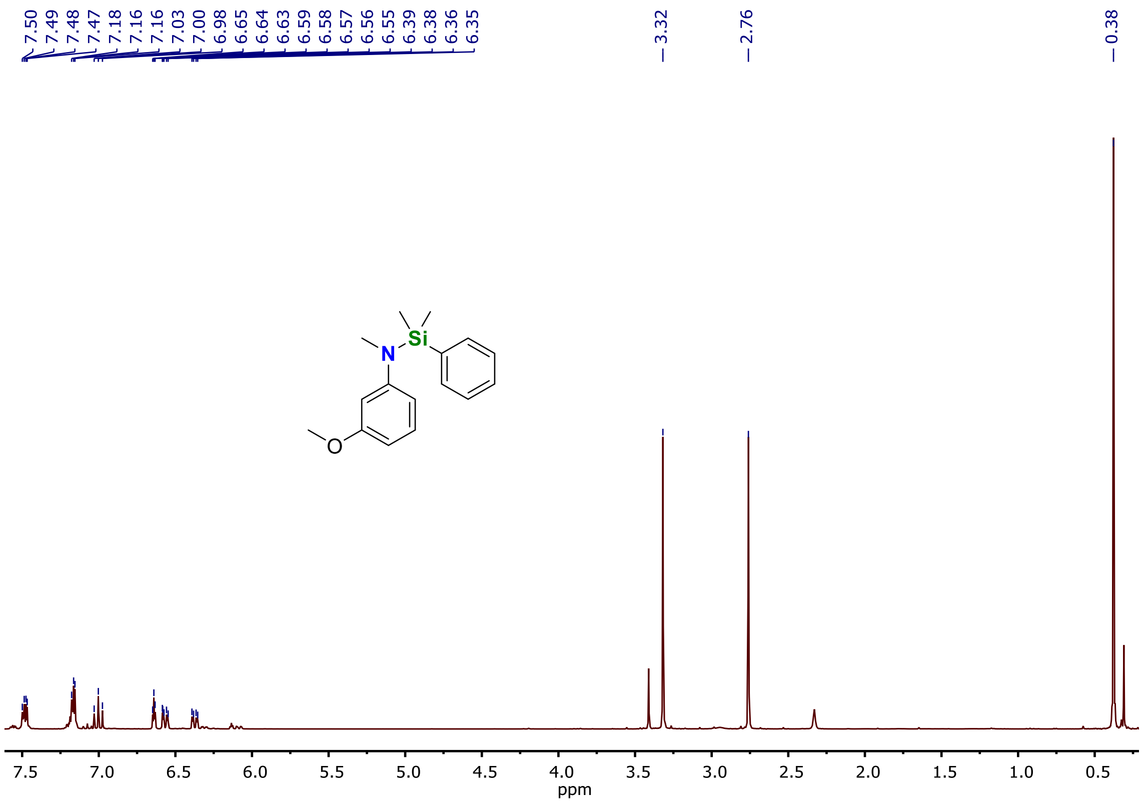

Figure S135. ^{1}H NMR spectrum of **7g** in C_6D_6 (400 MHz, 298K).

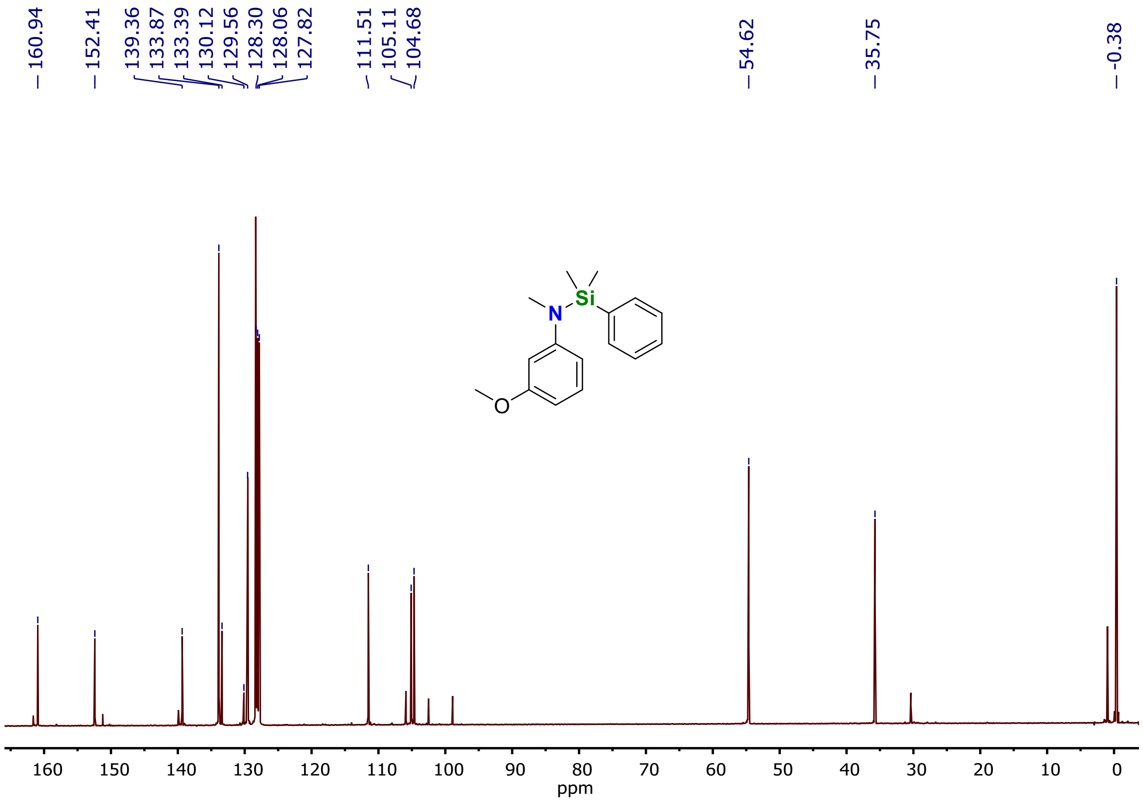

Figure S136. $^{13}C\{^{1}H\}$ NMR spectrum of **7g** in C_6D_6 (75 MHz, 298K).

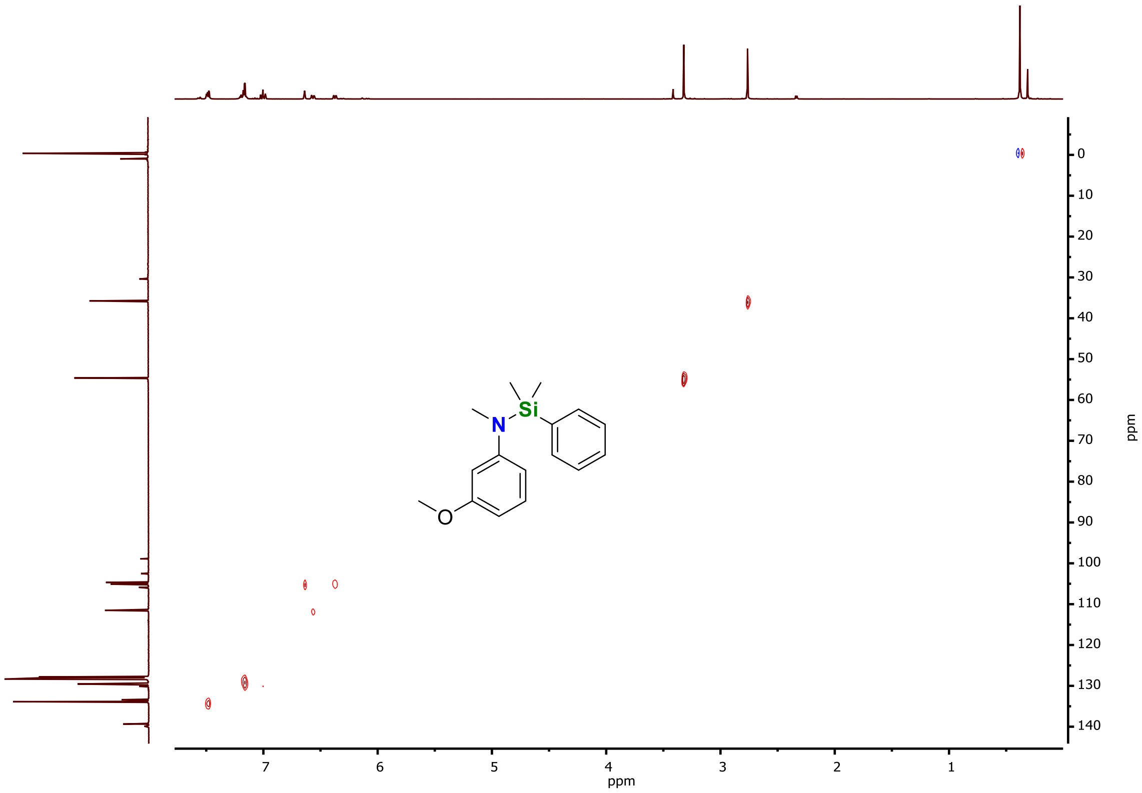

Figure S137. ^{1}H-^{13}C HSQC NMR spectrum of **7g** in C_6D_6 (298K).

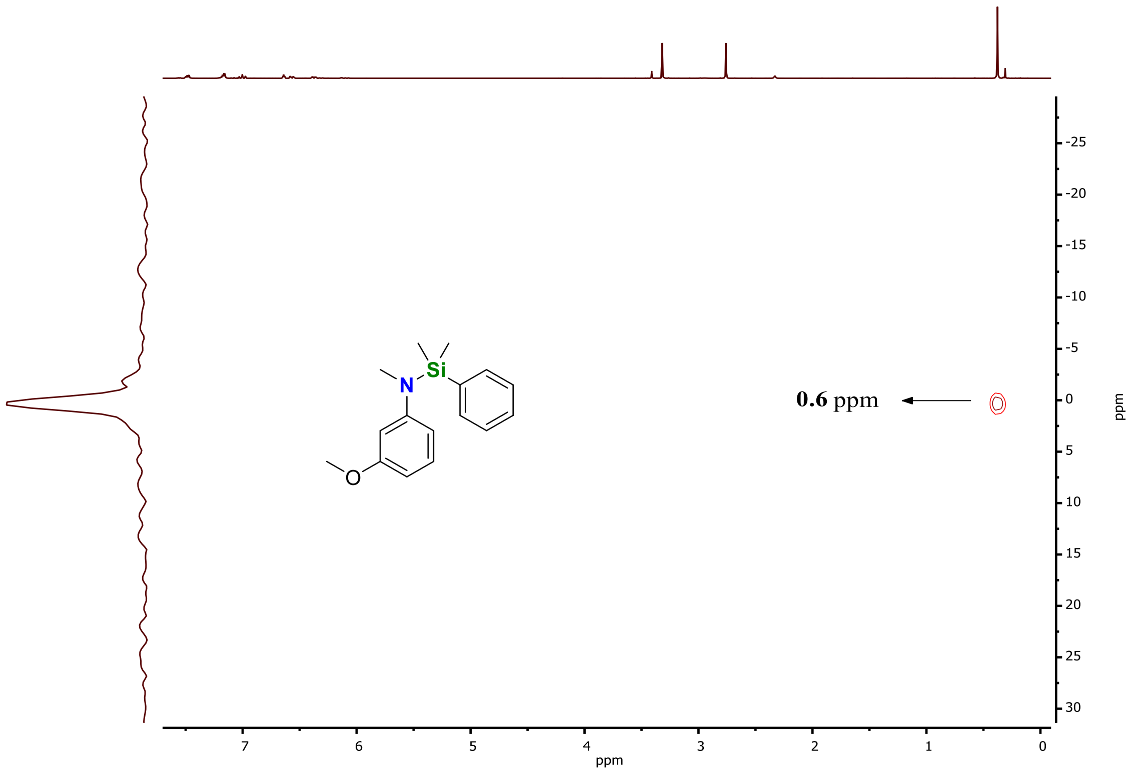

Figure S138. ^{1}H-^{29}Si HMQC NMR spectrum of **7g** in C_6D_6 (298K).

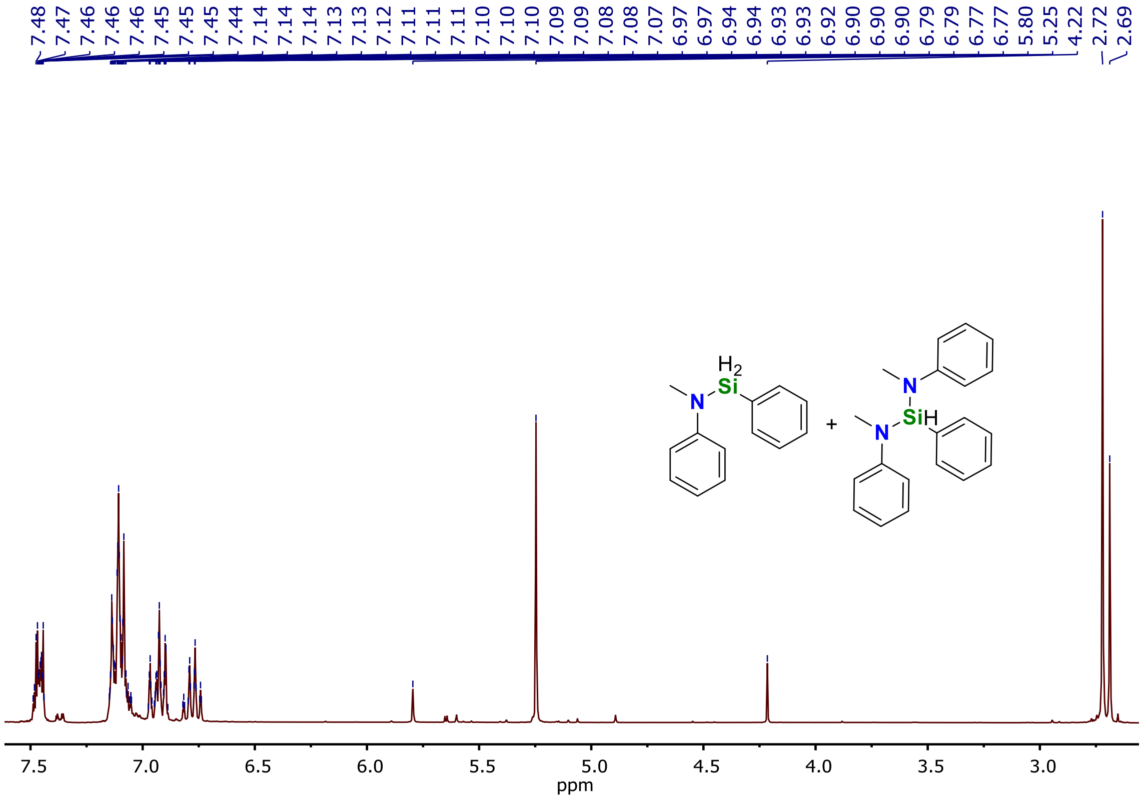

Figure S139. ^{1}H NMR spectrum of **7h-1** and **7h-2** in C_6D_6 (300 MHz, 298K).

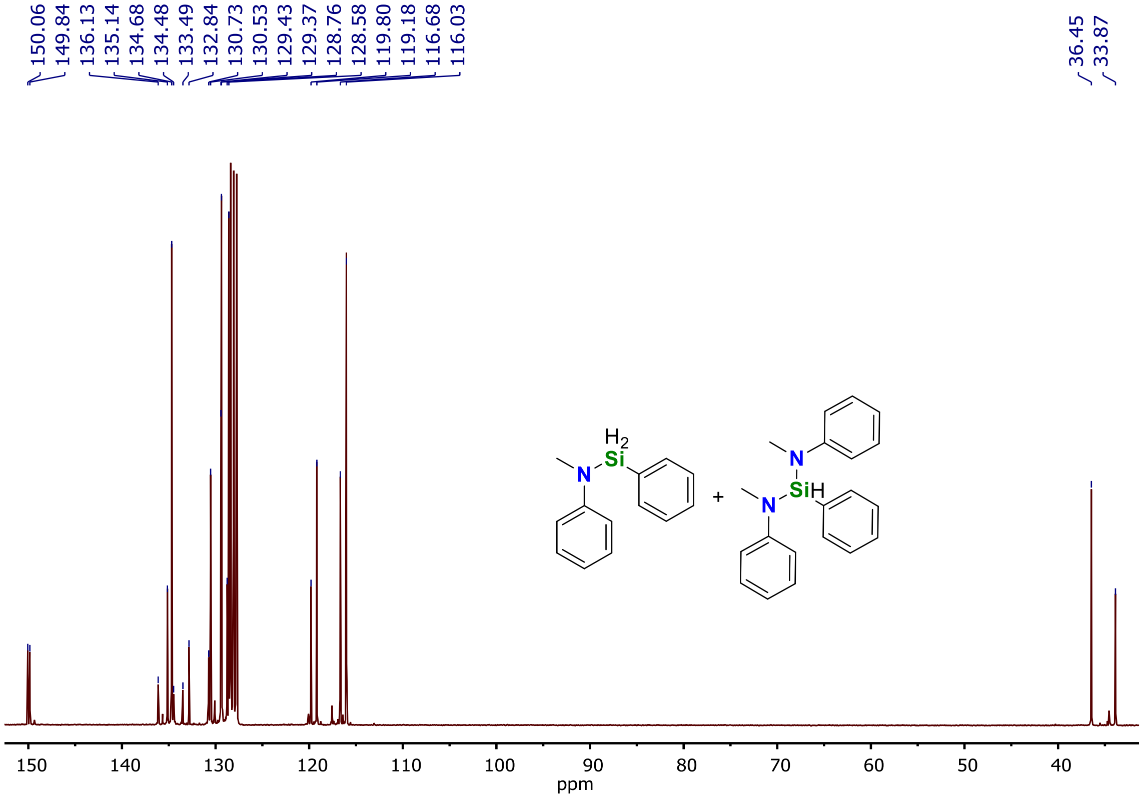

Figure S140. $^{13}C\{^{1}H\}$ NMR spectrum of **7h-1** and **7h-2** in C_6D_6 (75 MHz, 298K).

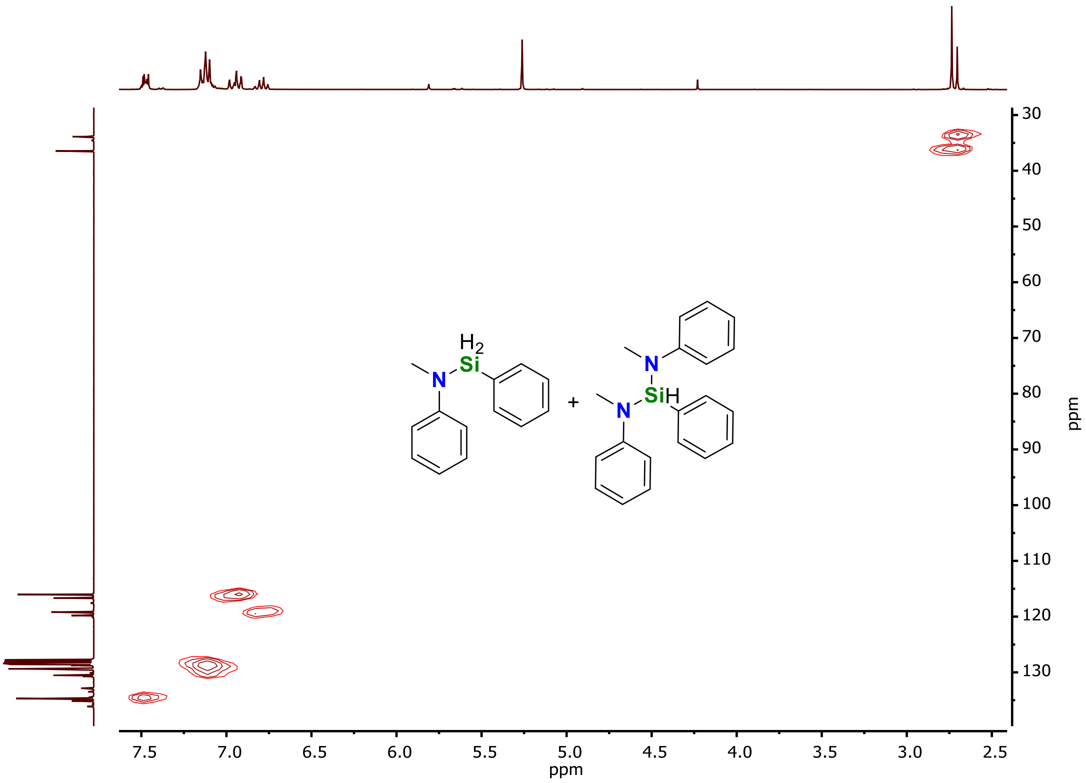

Figure S141. ^{1}H-^{13}C HSQC NMR spectrum of **7h-1** and **7h-2** in C_6D_6 (298K).

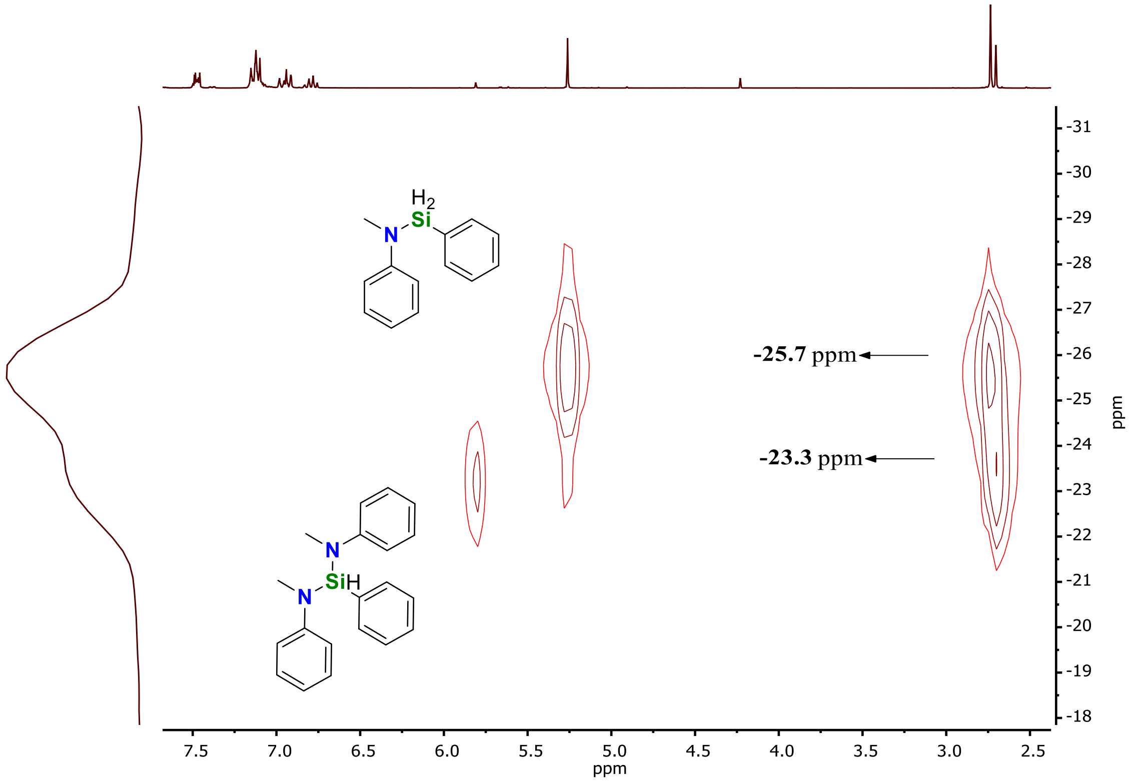

Figure S142. ^{1}H-^{29}Si HMQC NMR spectrum of **7h-1** and **7h-2** in C_6D_6 (298K).

11.5. NMR spectra of experiment at gram scale

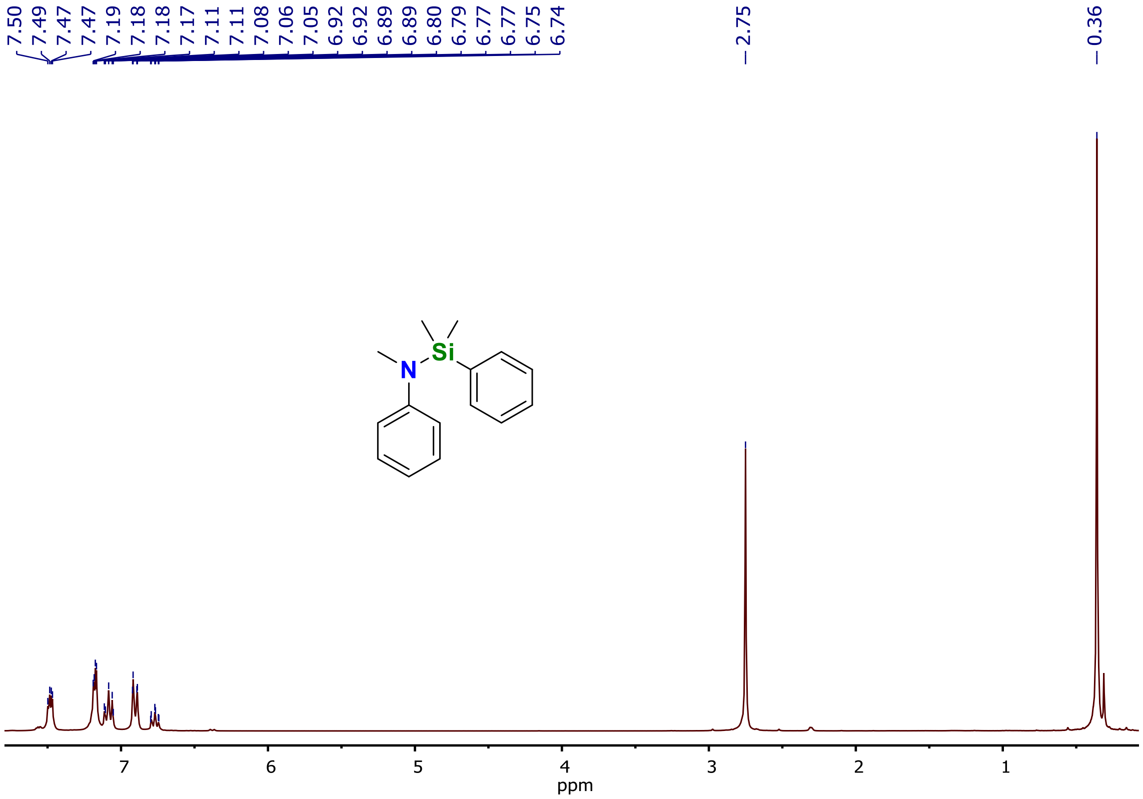

Figure S143. ^{1}H NMR spectrum of **7b** from gram scale in C_6D_6 (300 MHz, 298K).

12. Cartesian coordinates

Cartesian coordinates (in Å) and total energies (ZPVE included, in a.u.) of all the stationary points discussed in the text. All calculations have been performed at the RI-BP86-D3/def2-TZVP level.

4: E= -2537.3315783

Ir	0.536161000	-0.148246000	-0.061410000
H	0.595283000	-0.162888000	1.533518000
Cl	0.610236000	-0.034298000	-2.534777000
P	-1.715418000	0.162447000	0.101795000
Si	0.580393000	-2.403703000	0.162099000
O	2.317682000	-2.621604000	0.268148000
N	2.690691000	-0.354624000	-0.025312000
C	3.611246000	0.686144000	-0.089980000
C	3.168175000	2.038987000	-0.180194000
C	4.111068000	3.060174000	-0.192055000
H	3.756651000	4.091251000	-0.251137000
C	5.491100000	2.808855000	-0.144553000
H	6.199309000	3.637627000	-0.163014000
C	5.937505000	1.505639000	-0.073110000
H	7.006635000	1.300843000	-0.034316000
C	5.023774000	0.425860000	-0.034868000
C	5.481439000	-0.929336000	0.073395000
C	4.537745000	-1.917617000	0.158234000
H	4.807696000	-2.968274000	0.256291000
C	3.153598000	-1.606829000	0.126886000
C	1.721667000	2.399319000	-0.322873000
H	1.066865000	1.819885000	0.385886000
H	1.386167000	2.222037000	-1.352788000
H	1.540823000	3.453919000	-0.074363000
C	6.946749000	-1.263014000	0.105916000
H	7.455942000	-0.905727000	-0.801847000
H	7.099313000	-2.346557000	0.179272000
H	7.445399000	-0.788481000	0.964764000
C	-0.020001000	-3.284046000	1.713916000
H	0.347975000	-2.785430000	2.619938000
H	0.354876000	-4.319218000	1.703882000
H	-1.116731000	-3.323855000	1.765842000
C	0.165548000	-3.477136000	-1.322919000
H	-0.897516000	-3.745121000	-1.351646000
H	0.749110000	-4.408151000	-1.253831000
H	0.423979000	-2.952585000	-2.251711000
C	-2.784301000	-1.311123000	-0.367902000
H	-2.276617000	-2.145382000	0.148898000
C	-2.721212000	-1.598244000	-1.880775000
H	-3.244485000	-0.791459000	-2.421130000
H	-1.682013000	-1.579618000	-2.238053000
C	-3.413124000	-2.925255000	-2.223017000
H	-2.858637000	-3.759744000	-1.760670000
H	-3.374156000	-3.092557000	-3.310187000
C	-4.860999000	-2.956414000	-1.720533000
H	-5.452760000	-2.201028000	-2.267384000
H	-5.322758000	-3.932904000	-1.934166000
C	-4.925609000	-2.650202000	-0.219679000
H	-4.416843000	-3.454362000	0.341086000
H	-5.970267000	-2.635778000	0.128605000
C	-4.250580000	-1.308388000	0.104373000
H	-4.328517000	-1.102367000	1.181327000
H	-4.800708000	-0.507825000	-0.414825000
C	-2.256496000	0.578902000	1.837417000

H	-3.338447000	0.785823000	1.777570000
C	-2.046998000	-0.582019000	2.822949000
H	-2.550664000	-1.494596000	2.470909000
H	-0.972166000	-0.815098000	2.866224000
C	-2.552269000	-0.215347000	4.225304000
H	-3.645605000	-0.061390000	4.190473000
H	-2.374634000	-1.054154000	4.916301000
C	-1.877634000	1.059806000	4.745872000
H	-2.275654000	1.329127000	5.736534000
H	-0.798713000	0.867007000	4.879082000
C	-2.059245000	2.221000000	3.760739000
H	-1.530914000	3.117566000	4.121026000
H	-3.129691000	2.487281000	3.702969000
C	-1.555052000	1.846909000	2.360060000
H	-0.469760000	1.657265000	2.395971000
H	-1.703207000	2.689460000	1.668686000
C	-2.440632000	1.554249000	-0.950239000
H	-2.665778000	1.031846000	-1.895519000
C	-3.752157000	2.168556000	-0.418540000
H	-4.488710000	1.393745000	-0.169972000
H	-3.549046000	2.718053000	0.515439000
C	-4.357921000	3.147252000	-1.436000000
H	-4.631820000	2.594180000	-2.351726000
H	-5.290796000	3.571245000	-1.031952000
C	-3.367226000	4.260386000	-1.792168000
H	-3.169571000	4.868274000	-0.891137000
H	-3.802895000	4.939693000	-2.541418000
C	-2.050971000	3.666450000	-2.302646000
H	-1.321740000	4.463413000	-2.516522000
H	-2.233036000	3.145732000	-3.258924000
C	-1.441537000	2.665305000	-1.311449000
H	-0.556049000	2.208630000	-1.766225000
H	-1.117976000	3.196033000	-0.399910000

5: E= -3310.081571990

Ir	-0.205870000	-0.711826000	-0.162998000
S	-2.061723000	2.120989000	-0.039539000
P	2.019708000	-0.350041000	0.183386000
Si	-0.312520000	-0.749505000	-2.438090000
O	-1.994814000	-1.217724000	-2.599434000
O	-0.697302000	1.475299000	0.025668000
O	-2.787878000	2.051848000	1.220121000
N	-2.289088000	-1.119407000	-0.308338000
F	-0.733128000	4.269147000	0.859334000
F	-0.759189000	4.076947000	-1.323308000
F	-2.558943000	4.734639000	-0.259892000
C	-2.793182000	-1.266066000	-1.545753000
C	-4.173280000	-1.479355000	-1.780279000
H	-4.495231000	-1.562022000	-2.817133000
C	-5.057677000	-1.552707000	-0.736155000
C	-4.544871000	-1.422754000	0.597978000
C	-6.524956000	-1.753631000	-0.990634000
H	-6.893509000	-2.670399000	-0.506027000
H	-6.729317000	-1.828485000	-2.065348000
H	-7.111835000	-0.914373000	-0.587962000
C	-5.390325000	-1.510664000	1.728583000
H	-6.457075000	-1.678333000	1.586097000
C	-4.879791000	-1.390037000	3.004699000
H	-5.536176000	-1.462137000	3.872203000
C	-3.505536000	-1.163788000	3.180794000
H	-3.106692000	-1.052697000	4.191036000
C	-2.631601000	-1.051416000	2.107925000
C	-3.142273000	-1.195832000	0.785565000
C	-1.193349000	-0.716171000	2.381484000
H	-1.006280000	0.354380000	2.218911000
H	-0.489980000	-1.349801000	1.767230000
H	-0.917464000	-0.948349000	3.419483000
C	0.560652000	-2.047219000	-3.479615000

H	1.630578000	-1.822008000	-3.586479000
H	0.112577000	-2.048213000	-4.485208000
H	0.457189000	-3.052797000	-3.051633000
C	-0.225073000	0.888227000	-3.346175000
H	-0.775544000	1.664475000	-2.801799000
H	-0.681779000	0.764063000	-4.340133000
H	0.814374000	1.212580000	-3.486180000
C	2.898869000	0.720424000	-1.066679000
H	2.658136000	0.194814000	-2.011394000
C	4.432180000	0.799894000	-0.956077000
H	4.702687000	1.320223000	-0.023379000
H	4.882946000	-0.201777000	-0.902458000
C	5.022696000	1.567528000	-2.149576000
H	6.117314000	1.630370000	-2.046684000
H	4.825519000	0.996239000	-3.074100000
C	4.411028000	2.967720000	-2.280223000
H	4.696687000	3.569998000	-1.399615000
H	4.822899000	3.483962000	-3.161111000
C	2.881902000	2.893981000	-2.364000000
H	2.595637000	2.386259000	-3.301760000
H	2.443776000	3.902104000	-2.410955000
C	2.291791000	2.133004000	-1.170471000
H	1.197754000	2.085113000	-1.230716000
H	2.523912000	2.693265000	-0.252277000
C	2.267870000	0.333738000	1.916874000
H	1.663483000	-0.391640000	2.494661000
C	3.692008000	0.298977000	2.506113000
H	4.183920000	-0.668779000	2.336134000
H	4.311332000	1.060997000	2.006723000
C	3.647330000	0.589598000	4.015775000
H	3.096956000	-0.227136000	4.516107000
H	4.668548000	0.577207000	4.427921000
C	2.960604000	1.926672000	4.322331000
H	2.892351000	2.079373000	5.410618000
H	3.583696000	2.748759000	3.927992000
C	1.572036000	2.002788000	3.675574000
H	1.118844000	2.990781000	3.846692000
H	0.900930000	1.264317000	4.149522000
C	1.643624000	1.719702000	2.169538000
H	2.274490000	2.489561000	1.697539000
H	0.654953000	1.807616000	1.705016000
C	3.051664000	-1.903754000	0.240550000
H	4.075242000	-1.576854000	0.491979000
C	2.578892000	-2.873878000	1.339863000
H	2.572905000	-2.381433000	2.323641000
H	1.537645000	-3.168110000	1.128151000
C	3.467921000	-4.124495000	1.396926000
H	4.487583000	-3.830251000	1.702952000
H	3.093339000	-4.811179000	2.171815000
C	3.532101000	-4.831253000	0.038102000
H	4.199332000	-5.705375000	0.090396000
H	2.528416000	-5.213497000	-0.217971000
C	3.995566000	-3.866771000	-1.059630000
H	3.996263000	-4.368720000	-2.039588000
H	5.037846000	-3.560210000	-0.860895000
C	3.102413000	-2.620223000	-1.117231000
H	2.079739000	-2.915191000	-1.394555000
H	3.460990000	-1.940764000	-1.905282000
C	-1.496588000	3.908167000	-0.198658000
O	-2.785122000	1.836625000	-1.271577000
H	0.109291000	-2.249056000	-0.286439000
